# Factors Influencing the Implementation of Non‐Pharmacological Interventions for Behavioural and Psychological Symptoms of Dementia in Residential Aged‐Care Homes: A Systematic Review and Qualitative Evidence Synthesis: A systematic review

**DOI:** 10.1002/cl2.70029

**Published:** 2025-03-25

**Authors:** Hunduma Dinsa Ayeno, Gizat M. Kassie, Mustafa Atee, Tuan Nguyen

**Affiliations:** ^1^ Quality Use of Medicines and Pharmacy Research Centre, Clinical and Health Sciences University of South Australia Adelaide South Australia Australia; ^2^ Department of Pharmacy Ambo University Ambo Ethiopia; ^3^ The Dementia Centre, HammondCare Osborne Park Western Australia Australia; ^4^ Sydney Pharmacy School, Faculty of Medicine and Health The University of Sydney Sydney New South Wales Australia; ^5^ School of Nursing and Midwifery, Centre for Research in Aged Care Edith Cowan University Joondalup Western Australia Australia; ^6^ Curtin Medical School, Faculty of Health Sciences Curtin University Bentley Western Australia Australia; ^7^ School of Health Sciences Swinburne University of Technology Melbourne Victoria Australia; ^8^ National Ageing Research Institute Melbourne Victoria Australia

**Keywords:** behavioural, BPSD, dementia, factors, non‐pharmacological interventions, RACHs, residential aged care

## Abstract

**Background:**

Non‐pharmacological interventions (NPIs) are the primary approaches to the management of behavioural and psychological symptoms of dementia (BPSD), but studies have indicated that there is a suboptimal implementation. Although there are several studies on the factors influencing NPI implementation for BPSD at residential aged‐care homes (RACHs), there has not been a comprehensive qualitative systematic review on the topic.

**Objectives:**

This systematic review aimed to examine the qualitative studies that investigate the factors influencing the implementation of NPIs for managing BPSD in RACHs.

**Search Methods:**

Systematic searches were conducted up until 31 December 2023 using five databases: MEDLINE, EMCARE, EMBASE, CINAHL complete and APA PsycINFO.

**Selection Criteria:**

This systematic review included qualitative studies and qualitative data from mixed‐method studies on the implementation of NPIs for RACH residents with dementia experiencing BPSD. The research question and inclusion criteria for this review included the components of PICo: Population (aged‐care residents with dementia), Phenomenon of interest (factors influencing implementation of NPIs) and Context/setting (RACHs).

**Data Collection and Analysis:**

After screening and extracting the data, the methodological limitations were assessed using the Joanna Briggs Institute System for the Unified Management, Assessment, and Review of Information (JBI SUMARI) quality assessment tool. JBI SUMARI meta‐aggregative synthesis was used to synthesise the data. The extracted findings were categorised into the 10 Theoretical Domain Framework domains: knowledge, skills, environmental context and resources, social influences, reinforcement, emotions, intentions, beliefs about consequences, social and professional roles and beliefs about capability. Confidence in the output of qualitative research synthesis (CONQual) was used to assess the credibility and dependability of the synthesised findings.

**Main Results:**

Twenty‐four studies were included, from which factors influencing NPI implementation were extracted. Study participants included RACH managers, RACH care staff, families of aged‐care residents with dementia and volunteers. Amongst the studies specifying the gender of participants, there were 352 females (84.4%) and 46 males (15.6%). The method of data collection for the included studies consisted of eighteen interviews, five focus group discussions and one qualitative survey. All except one study had a quality assessment score of at least 60% based on the JBI SUMARI quality assessment tool. However, all studies were included regardless of the result of the quality assessment result. These studies spanned the period from 2010 to 2022 and were mostly conducted in the United Kingdom, Australia, the United States and Canada. Twenty‐four synthesised findings were identified (13 high, 7 moderate and 4 low ConQual scores). Examples of factors influencing the implementation of NPIs were collaboration amongst care staff and families of residents with dementia, belief in the efficacy of interventions, staffing, staff time constraints, funding, familiarity with the interventions, organisational support, communication amongst the care staff and with families of residents with dementia, education and training for the care staff and families of residents with dementia and familiarity with the residents with dementia.

**Authors' Conclusions:**

This systematic review highlights and synthesises factors influencing the implementation of NPIs for managing BPSD in RACHs. Key factors include collaboration amongst staff and families, organisational support, staffing, education and staff familiarity with both the interventions and residents. Strengthening these areas could enhance the care outcomes for aged‐care residents with dementia. For decision‐makers, these insights suggest the need for comprehensive strategies to improve NPI implementation. This could include ensuring appropriate staffing levels, enhancing collaboration, allocating adequate funds, providing training, strengthening organisational support and improving the quality of information exchange amongst care staff, between care staff and volunteers and families of residents with dementia. For researchers, the findings from this systematic review could provide valuable insights including the need to explore strategies to overcome barriers to NPI implementation, especially investigating innovative models for staffing and collaborative practice, examining the effectiveness of different education and training approaches, and exploring organisational policies and support mechanisms that can enhance the implementation of NPIs.

## 1 Plain Language Summary

Evidence indicates that there were various challenges in using non‐medication treatments, known as non‐pharmacological interventions (NPIs), to manage behavioural and psychological symptoms of dementia (BPSD) in residential aged‐care homes (RACHs), based on input from residential aged‐care home managers, care staff (e.g., nurses, caregivers), families of aged‐care residents with dementia and volunteers.

### 1.1 The Review in Brief

Factors such as how well care staff know the interventions and residents with dementia, education and training for care staff and families of residents with dementia, the number of staff, available funding, available time, collaboration among care staff and families of residents with dementia, communication amongst the care staff and families of resident with dementia, beliefs about the effectiveness of NPIs and organisational support can all influence how well non‐medication treatments for BPSD are implemented.

### 1.2 What Is This Review About?

NPIs are the best therapeutic option to manage BPSD. However, studies have shown that they are not being used as effectively as they could be. Even though there are many studies on what affects the use of NPIs for managing BPSD, no one has looked at all these studies together in a comprehensive way. This review looked at qualitative studies that explore why NPIs for managing BPSD are not always used well in residential aged care homes.

### 1.3 What Is the Aim of This Review?

This systematic review examines qualitative evidence on the factors influencing the implementation of NPIs for BPSD in residential aged care homes.

### 1.4 What Are the Main Findings of This Review?

#### 1.4.1 What Studies Are Included?

Twenty‐four published studies were included from which factors influencing the implementation of NPIs in residential aged care homes were extracted. Study participants included residential aged care home managers, residential aged care home staff, families of residents with dementia and volunteers. Amongst the studies specifying the gender of participants, there were 352 females (84.4%) and 46 males (15.6%). The method of data collection for the included studies consisted of 18 interviews, 5 focus group discussions and 1 qualitative survey. All except one study had a quality assessment score of at least 60%. However, all of these studies were included regardless of the result of the quality assessment. These studies spanned the period from 2010 to 2022 and were mostly conducted in the United Kingdom, Australia, the United States and Canada. Twenty‐four synthesised findings (13 high, 7 moderate and 4 low ConQual scores) were identified in this review.

#### 1.4.2 What Factors Influence the Implementation of NPIs?

Examples of factors influencing the implementation of NPIs were collaboration amongst care staff and families of residents with dementia, belief in the efficacy of interventions, staffing, funding, staff time constraints, familiarity with the interventions, organisational support, communication amongst the care staff and families of residents with dementia, education and training for care staff and families of residents with dementia and familiarity with residents with dementia.

### 1.5 What Do the Findings of This Review Mean?

For decision‐makers, these insights suggest the need for potential comprehensive strategies to improve the implementation of NPIs. This could include ensuring adequate staffing, implementing strategies to improve collaboration amongst care staff, volunteers and families, ensuring adequate available funds, providing staff training, strengthening organisational support and ensuring the quality of information exchange amongst care staff, between care staff and volunteers and families of aged care residents with dementia. For researchers, the findings from this systematic review provide valuable insights including the need for exploring strategies to overcome barriers to the implementation of NPIs especially investigating innovative models for staffing and collaborative practice, examining the effectiveness of different education and training, exploring organisational policies and support mechanisms that can enhance the implementation of NPIs.

### 1.6 How Up‐to‐Date Is This Review?

We searched five major research databases: MEDLINE, EMCARE, EMBASE, CINAHL complete and APA PsycINFO up until 31 December 2023.

## 2 Background

### 2.1 The Problem, Condition or Issue

BPSD are defined as a group of symptoms characterised by abnormal thought content (e.g., delusions), perceptual disturbances (e.g., hallucination), disturbed emotions (e.g., depression, anxiety, apathy, irritability, euphoria), abnormal motor functions (e.g., pacing, wandering, repetitive movements, physical aggression), verbal outbursts (e.g., yelling, calling out, repetitive speech, verbal aggression), disrupted circadian rhythms (e.g., sleep disturbances) and changes in appetite (e.g., either anorexia or hyperphagia) (Cerejeira et al. 2012; Kozman et al. 2006, 1). BPSD are subjective, complex and often with underlying multifactorial triggers, representing an interaction between neuropathological changes of dementia (e.g., atrophies), environmental factors (e.g., caregiver approach) and/or unmet needs (e.g., pain) (Kales et al. 2015).

BPSD are almost a universal experience with 97% of individuals experiencing at least one episode within 5 years of a dementia diagnosis (Steinberg et al. 2008). Nearly all people with Alzheimer's Disease (AD) had at least one symptom of BPSD, with apathy being the most common symptom and hallucination associated with disease severity (Cerejeira et al. 2012). Another study found that greater than 90% of people with AD and the behavioural variant of frontotemporal dementia (bvFTD) experienced BPSD, with the most common symptoms including apathy (AD, 57.4%; bvFTD, 74.6%), irritability/affective lability (AD, 50.5%; bvFTD, 52.5%) and agitation/aggression (AD, 42.3%; bvFTD, 49.7%) (Laganà et al. 2022). Another study conducted on individuals with mild‐to‐moderate to advanced dementia reported an overall BPSD prevalence of 94.6%, with apathy being the most common symptom (Castillo‐García et al. 2022).

BPSD contributed to lost days of work, high staff turnover and low job satisfaction for paid caregivers (Lyons and Champion 2022). Caregivers often report that BPSD are a difficult and challenging part of caring for someone with dementia (Tanya et al. 2016). BPSD make a substantial contribution to the total annual expenses of dementia care (Hermans et al. 2007) and around 30% of total annual dementia care cost was attributed to the direct cost of BPSD management (Beeri et al. 2002). Another study has also shown that BPSD (e.g., agitation) can increase the annual cost of care in RACHs by 44% (Burley et al. 2020).

### 2.2 The Intervention

The treatment of BPSD comprises NPIs and pharmacological interventions, with NPIs recommended as the primary choice (Magierski et al. 2020). Pharmacological intervention involves the use of medicines or drugs to prevent or treat the disease or symptoms (Bhardwaj and Misra 2018; Maciel et al. 2019). In contrast, NPIs refer to any form of treatment that does not directly involve medication and aims to enhance the healthcare needs of complex patients or improve the management of their chronic illnesses (Akintola et al. 2019; Castellano‐Tejedor 2022). NPIs are often complex interventions, and there are specific challenges in implementing such interventions (Regmi and Lwin 2021). The UK Medical Research Council (MRC) framework can help provide methodological guidance for evaluating these complex interventions (Skivington et al. 2021). We defined NPIs as any non‐medication or non‐drug intervention that is relevant to BPSD management, including but not limited to, sensory practices (e.g., aromatherapy, multisensory stimulation, massage, bright light therapy) and psychosocial practices (e.g., validation therapy, reminiscence therapy, music therapy, pet therapy, meaningful activities) (Scales et al. 2018). As per clinical guidelines to manage BPSD, NPIs are advocated as the primary treatment option instead of psychotropic medications (Dementia Centre for Research Collaboration [DCRC] and Centre for Healthy Brain Ageing [CHeBA] 2022; Guideline Adaptation Committee [GAC] 2016; Kales et al. 2015).

The individual experiencing BPSD requires a structured care approach that follows a continuous cycle of acceptance, assessment, action and reassessment. This approach should consider their physical, psychosocial and environmental needs, as well as the current and future capacity of care providers to meet these needs (New South Wales Ministry of Health [NSW MOH] 2022). At the core of the cycle is the concept of person‐centred care, a flexible process that includes the following components:

1. ACCEPT: The person and their background, along with the involvement, acknowledgement and expertise of their family and various healthcare professionals.
2. ASSESS: Assessment of the person's physical as well as psychosocial needs.
3. ACT: Creation and execution of a preliminary behavioural support plan.
4. REASSESS: Continuous evaluation of the person and immediate results, along with adjustments to the behavioural support plan (New South Wales Ministry of Health [NSW MOH] 2022).

Several studies have indicated that NPIs are more effective than pharmacological interventions in reducing BPSD and associated caregiver burden (Berg‐Weger and Stewart 2017; Plante‐Lepage et al. 2022; Sun et al. 2022; Yin et al. 2024). Despite the evidence of their effectiveness, NPIs are often not implemented appropriately in RACHs (Ervin et al. 2014; Van Der Ploeg et al. 2012).

Some challenges in implementing NPIs for BPSD in RACHs include but are not limited to, time constraints, shortage of workforce, the severity of physical and cognitive impairment (Ervin et al. 2014), staffing, staff collaboration and care time (Hussin et al. 2021).

### 2.3 Why Is It Important to Do This Review

The prevalence and severity of BPSD are higher in people with dementia living in RACHs compared to those in the community (Cerejeira et al. 2012; Olsen et al. 2016). NPIs prove effective in handling BPSD when applied correctly, and they are more commonly utilised by informed paid caregivers who have confidence in their efficacy (Lyons and Champion 2022). Several meta‐analyses revealed that NPIs such as reminiscence therapy (Li et al. 2021; Watt et al. 2021), massage therapy, music therapy, exercise therapy (Yin et al. 2024), combined massage and touch therapy, cognitive stimulation plus exercise plus social interaction (Watt et al. 2021), behavioural activation, acceptance and commitment therapy and cognitive behavioural therapy (Sun et al. 2022) are effective in the management of BPSD. Yet, there hasn't been a systematic review specifically addressing the factors influencing the implementation of NPIs for BPSD, despite the critical role implementation plays in the real‐world success of interventions. This gap exists even though various individual qualitative studies have highlighted diverse factors hindering the implementation of NPIs, such as lack of staff training, experience and confidence (Ervin et al. 2014; Hussin et al. 2021; Kolanowski et al. 2010; Sung et al. 2011), lack of familiarity with residents (Janzen et al. 2013), as well as understaffing (Ervin et al. 2014; Hussin et al. 2021; Kolanowski et al. 2010; Lewis et al. 2005) and lack of staff time (Ervin et al. 2014; Garrido et al. 2021; Hussin et al. 2021; Kolanowski et al. 2010; Miller et al. 2021; Sung et al. 2011). As this systematic review aims to explore the factors influencing the implementation of NPIs in RACHs and to pinpoint the factors that promote their successful implementation, it is crucial to focus on qualitative studies. This systematic review and synthesis of qualitative evidence will form part of a PhD project, which seeks to co‐design interventions for improving BPSD management in RACHs.

## 3 Objectives

This systematic review and qualitative evidence synthesis aimed to examine the qualitative studies that investigate the factors influencing the implementation of NPIs for managing BPSD in RACHs. The specific objectives are to (a) identify the factors that influence the implementation of NPIs for BPSD, (b) determine strategies for decision makers to improve implementation for BPSD and (c) identify future areas of research to improve implementation and overcome barriers to implementation of NPIs for BPSD.

## 4 Methods

The protocol systematic review was registered on PROSPERO (CRD42023388808, 11 February 2023) and published in Campbell Systematic Reviews (Ayeno et al. 2024).

### 4.1 Criteria for Considering Studies for This Review

The research question for this review included the components of PICo: Population (aged care residents with dementia experiencing BPSD), Phenomenon of interest (factors influencing implementation of NPIs) and Context/setting (residential aged care homes).


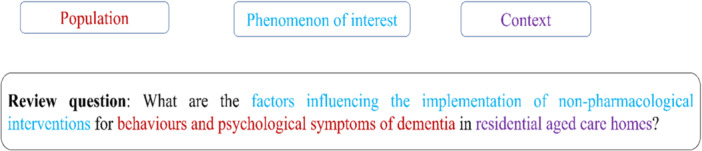


After the search was conducted, the eligibility for the study was assessed using the inclusion and exclusion criteria outlined in Table 1.

**Table 1:** Inclusion and exclusion criteria for studies in the systematic review.

Variables	Inclusion	Exclusion
Population*	The population of interest for this systematic review are people with dementia who experience BPSD, behavioural disturbances, or neuropsychiatric symptoms, or equivalent terms	People with dementia with no BPSD or related terms, general older people Findings from mixed population (BPSD plus general older people) where there are no separate results for BPSD or findings from neuropsychiatric symptoms unrelated to dementia
Phenomenon of interest	Factors or related terms such as barriers, facilitators, enablers, challenges, obstacles, or experiences of staff and/or residents with the intervention (e.g., music therapy, Montessori) or its implementation are included	NPIs relevant to BPSD with no factors or challenges or experiences being discussed or specified on their implementation or their use
Context	Residential aged‐care homes/nursing homes, long‐term care or related terms	Private homes, community and related terms
Types of studies	Qualitative studies: include but are not limited to interviews, focus group discussions, qualitative surveys and the qualitative data of the mixed method studies	Quantitative studies, non‐original qualitative data (e.g., reviews).
Language	Limited to English due to resource constraints	Not published in English
Date	From the inception of each database up to 31 December 2023	Beyond 31 December 2023

* Target groups or stakeholders such as aged‐care staff, families, or volunteers, from whom qualitative data was collected in the original studies, are not selection criteria for including or excluding studies in this systematic review, as they do not represent the population of interest. However, they served as a source of data about the phenomenon of interest concerning the population of interest (people with dementia experiencing BPSD).

#### 4.1.1 Types of Outcome Measures

The outcomes of this systematic review encompassed the perspective and experiences of various stakeholders including the RACH staff (comprising nurses, nurse aides or assistants, caregivers or personal care workers and allied health professionals like physiotherapists and occupational therapists), volunteers, families of dementia residents and the residents themselves on the factors influencing the implementation of NPIs in RACHs.

### 4.2 Search Methods for Identification of Studies

#### 4.2.1 Electronic Searches

With the guidance of an academic librarian from the University of South Australia, we created a list of search terms. Systematic searches were conducted across five databases: MEDLINE, EMCARE, EMBASE, CINAHL complete and APA PsycINFO. The OVID platform was used to search for articles in MEDLINE, EMCARE, EMBASE and APA PsycINFO, while EBSCOhost was used to search for articles in CINAHL. The development of search terms for the research question was centred around four key elements: the population (residents living with dementia), the phenomenon of interest (NPIs), the context (RACHs) and the study type (qualitative studies). Both Medical subject heading (MeSH) and keywords were used for each element as detailed in Appendix 1.

An initial search was conducted on MEDLINE to locate articles related to the subject. Keywords and index terms found in the titles, abstracts and indices of relevant articles were utilised to formulate a comprehensive search strategy for MEDLINE. A selection of articles meeting the inclusion criteria and eligible were chosen to validate the MEDLINE search strategy. This search strategy, along with all pertinent MeSH terms, was adapted from MEDLINE to other databases including EMCARE, EMBASE, CINAHL Complete and APA PsycINFO. The initial search was conducted in March 2023 and the research team decided to gather further comments before proceeding to the next step of extracting the findings. The protocol was then submitted for publication in June 2023 and published in March 2024. We updated the search to include results up to December 2023, which are annexed in Appendix 1, alongside the results of the search conducted in March 2023. The current PRISMA flow diagram includes the sum of the updated search and the results of initial research for the systematic review. A detailed list of each database searched and a line‐by‐line search process were provided in Appendix 1.

#### 4.2.2 Searching Other Resources

Additionally, searches were conducted in grey literature sources including Trove, ProQuest Dissertation & Thesis Global, abstracts of Alzheimer's Association International Conference (AAIC) and citation searching. Citation searching involved both forward and backward searching using each of the 24 included studies as seed papers.

We manually searched Google Scholar by entering the title of each seed paper, reviewing its references and examining articles that cited it. We did not seek additional studies from related systematic reviews, as we believe our search captured all relevant studies. No new eligible articles were identified after citation searching. We used the full title ‘Factors influencing the implementation of non‐pharmacological interventions for behavioural and psychological symptoms of dementia in residential aged care homes’ to search in Trove and ProQuest Dissertation & Thesis Global. The phrase ‘non‐pharmacological interventions for behavioural and psychological symptoms of dementia’ was used to search in AAIC abstracts (Appendix 2).

### 4.3 Data Collection and Analysis

#### 4.3.1 Selection of Studies

The studies identified through the searches were imported by the primary author (H. D. A.) into EndNote (version 20.2.1) to facilitate the removal of duplicates. First, the EndNote automated feature was used to remove duplicate studies and any remaining duplicates were removed manually. Following this, title and abstract screening was conducted by two reviewers (H. D. A. and G. M. K.) independently using the Joanna Briggs Institute System for the Unified Management, Assessment, and Review of Information (JBI SUMARI) software (Aromataris and Munn 2020). Then a full‐text review was undertaken by the same two reviewers (H. D. A. and G. M. K.). Any disagreements regarding eligibility were resolved by discussion between the two reviewers and if necessary, the involvement of the other authors (M. A. and T. N.), to reach a consensus. The reason for excluding articles at full‐text screening can be found in Appendix 4.

#### 4.3.2 Data Extraction and Management

Descriptive and methodological information from each paper was extracted using the JBI SUMARI data extraction form (Aromataris and Munn 2020) by (H. D. A.) and cross‐checked by the other reviewers (G. M. K., M. A., T. N.). The identified factors influencing the implementation of NPIs were then coded into different categories and matched against the Theoretical Domains Framework (TDF) domains by the primary researcher (H. D. A.) and independently checked by other authors (G. M. K., M. A., T. N.).

##### 4.3.2.1 Assessment of Methodological Limitations in Included Studies

The methodological quality of the included articles was assessed independently by the two reviewers (H. D. A. and G. M. K.), using the standardised JBI SUMARI critical appraisal checklist (Aromataris and Munn 2020). The checklist contains 10 questions, enabling rapid evaluation of studies. Questions were scored as 0 or 1, reflecting the extent to which information from the paper answered each question (0 = no or unclear; 1 = Yes). Studies were included for qualitative synthesis regardless of their quality assessment score (Table 3).

#### 4.3.3 Data Synthesis

Data synthesis was performed using JBI SUMARI meta‐aggregative synthesis, a pragmatic approach for synthesising and summarising the practicalities and usefulness of qualitative study findings (Korhonen et al. 2013). Following data extraction on JBI SUMARI, individual findings were grouped into categories based on their similarity in meaning. This was done by the primary author (H. D. A.) and reviewed by the other authors (G. M. K., M. A., T. N.) regardless of contexts such as differences in the nature and type of NPIs, resources available to each RACH, type of participants (e.g., nurses, allied health professionals, caregivers) in each study and other related factors. These categories were then matched against different TDF domains. Categories within each domain either stood alone or were combined to form a synthesised finding based on conceptual similarity. Next, these categories were subjected to synthesis to generate a comprehensive set of findings. This involved pooling the extracted data from the included studies and summarising the findings based on the overall strength of the evidence. Then, finally, the implication for practice, policy and research was generated based on the synthesised findings by the primary author (H. D. A.) and reviewed and approved by the other authors (M. A., G. M. K. and T. N.).

#### 4.3.4 Summary of Findings and Assessment of the Certainty of the Evidence

To determine the confidence in the synthesised results, the synthesised findings were evaluated using the Confidence in the Output of Qualitative Research Synthesis (ConQual) process and were summarised to provide a ConQual score for each synthesised finding (Munn et al. 2014).

Initially, all the included studies were ranked as high because they were qualitative. After this initial ranking, dependability scores were assigned to each finding based on five yes/no questions, including three on congruity, one on researcher culture and one on researcher influence, to assess the dependability of the findings. If an individual finding received four to five ‘yes’ responses, the finding remained unchanged at its current level (high). If it received two to three ‘yes’ responses, it was downgraded by one level (from high to moderate) and for zero to one ‘yes’ response, it was moved down two levels (from high to low, or moderate to very low). The synthesised finding could be downgraded depending on the overall dependability of the included findings. For instance, if most of the individual findings were rated as having ‘low’ dependability, the same rating was applied to the synthesised finding (Munn et al. 2014).

In a separate column (Table 4), a credibility scoring was assigned for each synthesised finding using the initial ranking (i.e., ‘high for all studies’ because all included studies were qualitative) as a baseline. First, individual findings were categorised as either unequivocal, where a finding is supported by a clear illustration with an unquestionable association; credible, where a finding is supported by an illustration, but its association is unclear and open to criticism; or unsupported, where a finding lacks support from or is missing an accompanying illustration (Munn et al. 2014). Then, if a synthesised finding was formulated from only unequivocal findings, the initial ranking remained unaltered. If the synthesised finding was based on a combination of unequivocal and credible findings, the initial ranking was downgraded by one level. For only credible findings, the downgrade would be by two levels and for a mixture of credible and unsupported findings, it would be downgraded by three levels (Munn et al. 2014). However, in this systematic review, there were no synthesised findings composed solely of credible findings. Unsupported findings were excluded based on the recommendation from the JBI manual, which states that unsupported findings should not be included in meta‐aggregative synthesis for JBI qualitative reviews (Aromataris and Munn 2020). Once the findings were evaluated according to the dependability and credibility rules, the cumulative score which is referred to as the ConQual score was assigned to each synthesised finding (Munn et al. 2014).

## 5 Results

### 5.1 Description of Studies

#### 5.1.1 Results of the Search

**Figure 1:**
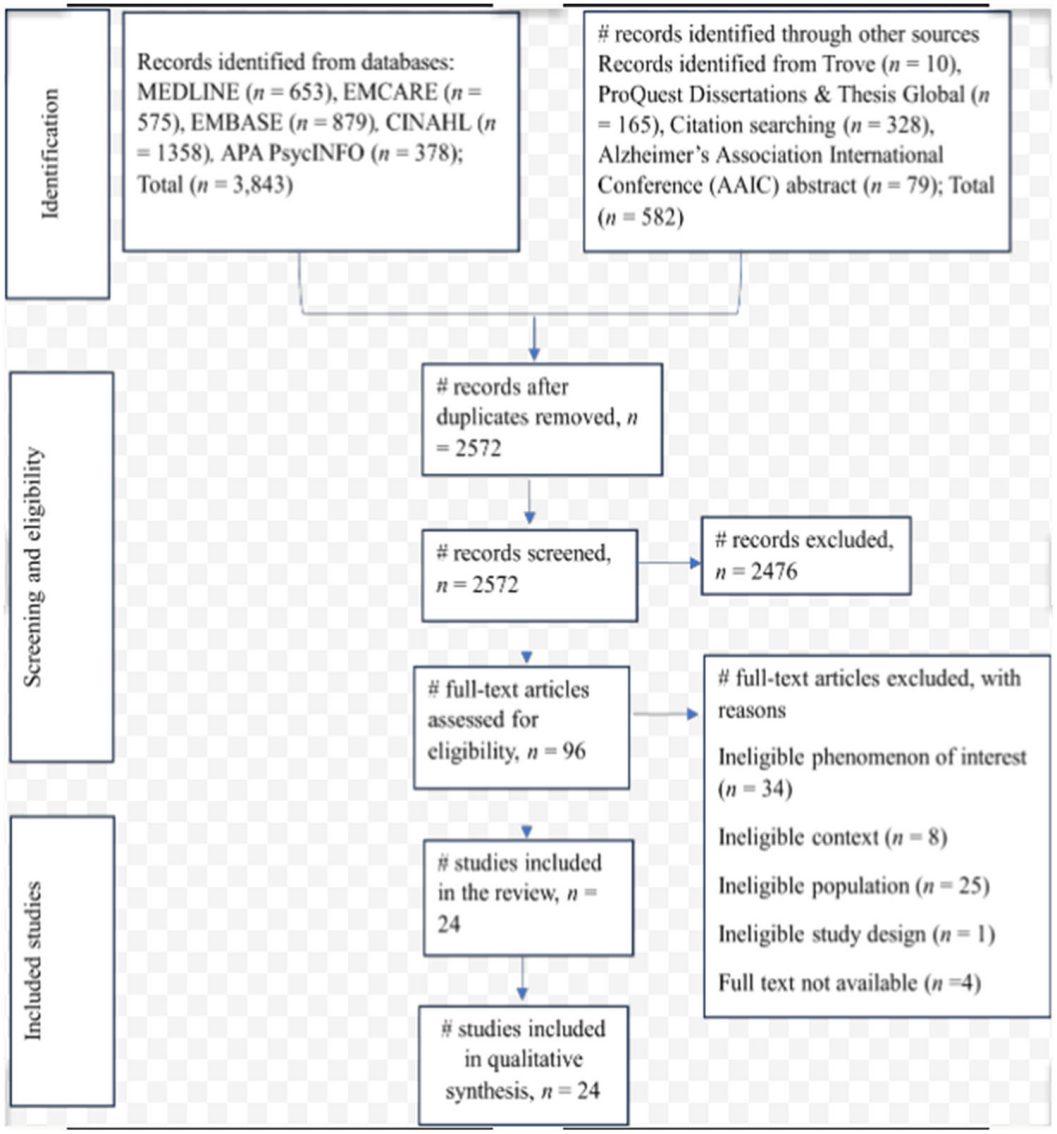
PRISMA flow chart for the study selection process.

The PRISMA flow diagram (Figure 1) includes the sum of the results of both the search conducted in March 2023 and the search updated as of 31st December 2023. Twenty‐four published studies met the inclusion criteria and were consequently included in the final qualitative synthesis (Figure 1). No eligible articles were identified through searches of other sources. Where applicable, searches such as citation searching returned articles already identified in the database search.

#### 5.1.2 Included Studies

##### 5.1.2.1 Characteristics of Included Studies

The included studies spanned the period from 2010 to 2022 were conducted across various countries, namely the United Kingdom (*n* = 6), Australia (*n* = 4), the United States (*n* = 3), Canada (*n* = 3), the Netherlands (*n* = 2), France (*n* = 1), Ireland (*n* = 1), Israel (*n* = 1), Malaysia (*n* = 1), Pakistan (*n* = 1) and South Korea (*n* = 1). Full characteristics of the included articles and samples are presented in Table 2. Amongst the studies specifying the gender of participants, there were 352 females (84.4%) and 46 males (15.6%). The type of study participants involved includes staff members/care staff from RACHs (*n* = 9) (Backhouse et al. 2016; Garrido et al. 2021; Griffiths et al. 2019; Gulliver et al. 2021; Kaasalainen et al. 2019; Kolanowski et al. 2010; McKenna et al. 2022; Nunez et al. 2018; Tasseron‐Dries et al. 2021), caregivers/care assistants/personal care attendants (*n* = 8) (Chaudhry et al. 2020; Cohen‐Mansfield and Meschiany 2022; Ervin et al. 2014; Hussin et al. 2021; Janzen et al. 2013; Kong et al. 2022; Lawrence et al. 2016; Webster et al. 2022), nurses (*n* = 7) (Clifford and Doody 2018; Cohen‐Mansfield and Meschiany 2022; Ervin et al. 2014; Janzen et al. 2013; Kong et al. 2022; Lawrence et al. 2016; Webster et al. 2022), managers of RACHs (*n* = 7) (Cohen‐Mansfield and Meschiany 2022; Griffiths et al. 2019; Janzen et al. 2013; Kwak et al. 2021; Lawrence et al. 2016; Van Der Ploeg et al. 2012; Webster et al. 2022), relatives/family of resident with dementia (*n* = 6) (Garrido et al. 2021; Griffiths et al. 2019; Gulliver et al. 2021; Kaasalainen et al. 2019; Nunez et al. 2018; Tasseron‐Dries et al. 2021), volunteers (*n* = 5) (Garrido et al. 2021; Kaasalainen et al. 2019; Kwak et al. 2021; Tasseron‐Dries et al. 2021; Van Der Ploeg et al. 2012), residents with dementia (*n* = 4) (Chaudhry et al. 2020; Garrido et al. 2021; Griffiths et al. 2019; Hussin et al. 2021), activity therapists (*n* = 3) (Cohen‐Mansfield and Meschiany 2022; Kwak et al. 2021; Lawrence et al. 2016), social workers (*n* = 2) (Cohen‐Mansfield and Meschiany 2022; Kwak et al. 2021), recreation staff (*n* = 2) (Ducak et al. 2018; Janzen et al. 2013), life enrichment coordinator(*n* = 2) (Kwak et al. 2021; Van Der Ploeg et al. 2012), activity coordinators (*n* = 2) (Ervin et al. 2014; Janzen et al. 2013), multidisciplinary consultants (*n* = 1) (Ducak et al. 2018), occupational therapists (*n* = 1) (Cohen‐Mansfield and Meschiany 2022), gerontology care assistants, medico‐psychological carers, facilitators and psychologists (*n* = 1) (Forget et al. 2021), Dementia Care Mapping mappers and expert mappers (*n* = 1) (Griffiths et al. 2019), resident assessment instrument coordinators, dietary specialists and art specialist (*n* = 1) (Janzen et al. 2013).

**Table 2:** Characteristics of included studies [ordered by study ID].

Study	Country	Setting/context/culture	Participant characteristics and sample size	Type of non‐pharmacologic interventions	Phenomena of interest	Method for data collection and analysis	Description of main results
Backhouse et al. (2016) {published data only}	UK	Care homes	Participants: 40 care home staff	Non‐pharmacologic interventions such as gardening, Jigsaws and flower arranging	Barriers to including dementia residents in activities	**Data collection**: interviews and observations and **Data analysis**: Framework approach	Physical or mental impairment, feeling ‘uneasy’ around those with BPSD, inequality in the allocation of activity provision, view that activities or NPIs are extras, residents' reluctance to take part in activities were barriers to the use of NPIs
Chaudhry et al. (2020) {published data only}	Pakistan	Residential care home	Participants (*n* = 24): 12 residents living with dementia and 12 paid caregivers (24 dyads)	Montessori interventions	Barriers to delivering the adapted Montessori intervention	**Data collection**: semi‐structured interviews and **Data analysis**: a framework analysis	Workload with the engagement of staff in routine activities and fluctuations in mood, behavioural problems and verbal outbursts of residents were barriers to Montessori interventions
Clifford and Doody (2018) {published data only}	Ireland	Both public and private long‐stay facilities	Participants: 9 nurses	*Person‐centred care*	Factors influencing person‐centred care in dementia	**Data collection**: in‐depth audio‐recorded interviews and **Data analysis**: Qualitative content analyses	Shortage of staff, lack of funding, lack of education and training, frequency and severity of resident's behaviour, lack of management support and attitude of managers towards residents' behaviour were mentioned as barriers while collaboration between nurses and activities coordinator, getting to know the person, continuing education, attitudes and being self ‐aware when caring and good communication skills were facilitators to person‐centred care
Cohen‐Mansfield and Meschiany (2022) {published data only}	Israel	Nursing homes	Participants: 41 nursing home staff (5 chief executive officers (CEOs), 5 social workers, 10 nurses, 8 occupational therapists, 4 activity) workers and 10 nursing assistants. One staff member worked in two roles)	Quality of care to improve quality of life	Barriers to providing quality care (maximising residents' engagement and pleasure and minimising disruption to residents' natural cycle of activity and inactivity, such as sleep, walking and movement, etc.) for persons with dementia	**Data collection**: interview and **Data analysis:** thematic analysis	Concerns related to resident's behavioural challenges and violence, rigid care routine, lack of regular professional and individualised activities, lack of communication skills among staff, lack of financial resources, lack of manpower, lack of training and lack of equipment and adequate facilities were barriers to providing quality of care using NPIs
Ducak et al. (2018) {published data only}	Canada	Long‐term care homes	Participants (*n* = 17): participants were classified into two groups ‘recreation staff’ (12) and ‘multidisciplinary consultants’ as LTC staff members (5)	Montessori Methods	Barriers and facilitators to implementing Montessori methods for dementia	**Data collection**: semi‐structured telephone interviews and **Data analysis**: thematic analysis	Fear of disapproval from ministry, lack of staff buy‐in, rigid routine, medicalised nature of LTC and adherence to routines, difficulty to alter a deeply ingrained medical nature of LTC, lack of interdisciplinary collaboration in LTC, limited time available for recreation staff to get resident onboard, unwillingness of nursing staff to use MMD, lack of funding, very low staff‐to‐resident ratio, lack of understanding were barriers whereas knowledge of usefulness, seeing results is believing, support from management and support from family were the facilitators to implementation of Montessori interventions
Ervin et al. (2014) {published data only}	Australia	Residential aged‐care facilities	130 staff responded to the survey (registered nurse, personal care attendant, student or an activity coordinator	Behaviour‐oriented strategies; Cognitive‐oriented strategies; Stimulation‐oriented strategies; Emotion‐oriented strategies	Barriers to implementation of interventions for BPSD	**Data collection**: a qualitative survey using open‐ended questions **Data analysis**: Collaborative analysis	The need for more education and training, the effectiveness of the strategy as low and time constraints were cited as barriers to non‐pharmacological intervention implementation
Forget et al. (2021) {published data only}	France	Nursing homes	11 participants which include: gerontology care assistants (ASG) (2), medico‐psychological carers (AMP) (1), facilitators (ANIM) (4) or psychologists (PSY) (4)	Animal‐assisted intervention	What weighed in favour of or against the implementation of an animal‐assisted intervention.	**Data collection**: Semi‐structured interviews and **Data analysis**: qualitative content analysis	Organisational constraints, concern about animal hygiene, animal phobia, budget constraint, work overload, management constraint and concern about animal's quality of life were found to be brakes whereas intervention effectiveness, improved quality of life of the elderly, improved social interaction, increased cognitive stimulation, patient motivation, physical stimulation, positive feedback from families and other caregivers were levers to the implementation of animal‐assisted therapy
Garrido et al. (2021) {published data only}	Australia	Long‐term residential aged‐care facilities	Participants, 4 males and 13 females, staff associated with five different providers of long‐term residential aged‐care facilities in Australia (n = 11), family caregivers (n = 4), one person with dementia and one volunteer musician	Music playlist	Challenges to implementing music playlist programmes	**Data collection**: focus group discussions and **Data analysis**: thematic analysis	Lack of time, not seeing the value of music, challenges with storage of ipods and difficulty of music individualisation were the main barriers to music implementation
Griffiths et al. (2019) {published data only}	UK	Residential, nursing and dementia‐care homes	A total of 83 participants were interviewed: 17 care home Managers, 25 DCM mappers, 27 staff members, 6 relatives, 2 residents and 6 expert mappers	Dementia Care Mapping (staff‐led psychosocial intervention)	Barriers and facilitators to DCM implementation	**Data collection**: Semi‐structured interviews and **Data analysis:** Framework Analysis approach	The findings demonstrated that implementing DCM in care homes is complex and many factors may facilitate or prevent successful implementation. Managers, mappers, expert mappers and staff members identified barriers and facilitators; no residents or relatives highlighted any barriers or facilitators
Gulliver et al. (2021) {published data only}	Australia	Residential aged‐care setting	Participants (*n* = 9) = 6 care home staff and 3 family and community members	Music engagement programme	Barriers to programme implementation and sustainability	**Data collection**: interviews and **Data analysis**: Thematic analysis	Insufficient time and fear of not producing a good voice were barriers while staff familiarity with music was a facilitator for implementing the music engagement programme
Janzen et al. (2013) {published data only}	Canada	Long‐term care	A total of 44 LTC staff members participated. 8 registered nurses (RNs), 13 registered practical nurses 8 personal support workers (PSWs), 6 recreation specialists or coordinators, 3 DOCs, 2‐unit coordinators, a recreation assistant, a resident assessment instrument coordinator, a dietary specialist and an art therapist	Non‐pharmacologic interventions such as pet therapy, calming music, reminiscence therapy and 17 other interventions	Facilitators and barriers to NPI implementation	**Data collection**: Focus group discussions and interviews and **Data analysis**: Content analysis	Time constraints and low staff‐to‐resident ratios were the two most interconnected barriers whereas familiarity with the resident and the degree of empathy experienced by staff are facilitators of NPI implementation
Kaasalainen et al. (2019) {published data only}	Canada	Long‐term care homes	Participants (*n* = 25): 18 staff, 5 family members and 2 volunteers	Namaste care	Barriers and facilitators to namaste care implementation	**Data collection**: interviews and **Data analysis**: content analysis	Strong support from the administration, staff burden over time, adverse events such as incidence of skin breakdown, understaffing and lack of funding were barriers to the implementation of namaste care
Kolanowski et al. (2010) {published data only}	USA	Long‐term care facilities	Participants were 35 staff members	Recreational activities, aromatherapy, music, relaxation and behavioural techniques (distraction and non‐confrontational interaction)	Barriers and facilitators to the use of non‐pharmacological interventions	**Data collection:** Focus Group Discussions and **Analysis:** standard content and thematic analysis	Lack of education, lack of skills, unfamiliarity with the residents and residents' characteristics were among the barriers to the use of NPIs whereas engaging the residents back to life was a facilitator
Kong et al. (2022) {published data only}	Republic of Korea	Nursing homes	Participants: a total of 24 staff members (nurses, nurse's aides, or care workers)	Person‐centred care	Barriers to implementing ‐person‐centred care	**Data collection**: interviews and **Data analysis**: Qualitative content analysis	Insufficient staff, lack of time, inappropriate physical environment, staff's lack of education, family's lack of education, staff's negative attitudes, staff's hurtful experiences, lack of communication among staff, residents and families, lack of trust between staff and families and conflicts among/between staff and families were barriers to person‐centred care
Kwak et al. (2021) {published data only}	USA	Nursing homes	Participants *n* = 145: (Activity Coordinator/Director/Supervisor 76, administrator/Chief Operating Officer 44, Life Enrichment Director/Coordinator 11, social worker 8 and volunteer coordinator and other 6)	Music and Memory programme	Facilitators and barriers related to the implementation of the Music and Memory programme	**Data collection**: a qualitative survey with open‐ended questions and **Data analysis**: content analysis	Lack of being valued, lack of time, lack of staff buy‐in, inadequate staffing, need to educate initial staff and new staff, use of technology, costs for buying music, lack of resident buy‐in, difficulty in identifying specific songs for the playlist, lack or inconsistency of volunteers and family support were barriers whereas using headphones, calming effect of music, support of facility personnel, seeing the positive effects of M&M, training and support for M&M, family and volunteers involvement, accessibility of equipment and funding were facilitators of implementing M&M programme
Lawrence et al. (2016) {published data only}	UK	Care homes	Participants (*n* = 119): included 53 care assistants (45%), 30 senior care assistants (25%), 13 activity therapists (11%), 6 registered nurses (6%), 5 deputy managers (4%), 2 managers (2%) and 10 other staff (8%)	Psychosocial interventions	Barriers and facilitators of person‐centred care implementation	**Data collection**: Focus group discussion and **Data analysis**: thematic analysis	Lack of recognition from society, managers and relatives, fear of criticism from the training team, dislike of the word ‘intervention’, lack of resources‐pressurised environment, time constraints likely to undermine intervention, relationships strained with relatives critical of staff, divisions between staff groups, concern about engaging all staff in intervention were barriers while mutual respect and reciprocity key to good care, collective responsibility enables staff to meet resident needs were facilitators to implementation of psychosocial interventions
McKenna et al. (2022) {published data only}	UK	Care homes, nursing homes and assisted living accommodations	Participants: 13 care staff with experience in psychological formulation	Formulation‐led care (psychological approach to managing behaviour in dementia)	Opinions regarding barriers and facilitators that affect the utility of this psychological intervention	**Data collection**: semi‐structured individual interviews and **Data analysis**: thematic analysis	Little awareness of psychologist support, intervention not meeting staff's expectation, being pessimistic, sense of frustration and powerlessness that their knowledge and experience undervalued, absence of joint working and belief about lack of originality of the psychologist's suggestions were barriers to formulation led care whereas team working, being newer staff, the benefit gained(understanding) from the intervention were facilitators
Hussin et al. (2021) {published data only}	Malaysia	Seven secondary care facilities comprising of two day care centres and five nursing homes	Participants: 12 caregivers and 11 people with dementia	Physical exercise, music therapy, reminiscence therapy and pet therapy	Barriers to person‐centred care in dementia	**Data collection**: semi‐structured interviews and **Data analysis**: Thematic analysis	Lack of Training, inadequate staff numbers, time constraints, communication barriers, less supportive family members and responsibility outside the job scope were barriers whereas creating meaningful conversations with PWD, multidisciplinary collaboration and Elderspeak were facilitators to NPI implementation
Miller et al. (2021) {published data only}	USA	Assisted living communities	Participants: care staff from three assisted living communities (*n* = 2–6 per community)	Psychosocial and environmental care	Barriers and facilitators to implementation practice	Semi‐structured group interviews and **Data analysis**: The qualitative group interview notes were synthesised by the authors; no qualitative analyses were possible or required given their succinct nature	Activity supplies disappearing, lack of time and unfavourable weather conditions were barriers while championing by the activity director, designating a single staff member responsible for filling and turning on the diffuser each day were facilitators to implementing psychosocial interventions
Nunez et al. (2018) {published data only}	UK	Care homes	Participants were family carers (*n* = 5), care staff (*n* = 12) and for informal interviews: night care staff (*n* = 19) in five care homes, including care staff, senior care staff and nurses	*Person‐centred care*	Factors influencing quality person‐centred care for sleep disturbances	**Data collection**: Focus group discussion and interviews and **Data analysis**: Thematic analysis	Insufficient staffing levels, night and day care staff working relationship, nurse burden and responsibilities, communication as a challenge and difficulty of connecting with and knowing residents were mentioned as challenges during the night‐time care for BPSD
Pieper et al. (2018) {published data only}	Netherlands	Nursing homes	Healthcare professionals at nursing homes. Details not specified	Multidisciplinary and multicomponent intervention	Barriers and facilitators to implementation of STA OP!	**Data collection**: mixed methods design (1) notes and memos (2) semi‐structured interviews and **Data analysis**: thematic analysis	Organisational changes or other innovations at the time of the implementation, staff turnover, shortage of staff and high workload and having different schedules were barriers while the presence of a person with a motivational leadership style, interdisciplinary learning and cooperation and seeing results motivated them to utilise the intervention were facilitators to the STA OP! implementation
Tasseron‐Dries et al. (2021) {published data only}	Netherlands	Nursing homes	Participants: 43 participants; 10 family caregivers, 31 staff members and 2 volunteers about their experiences with the Namaste Care Family programme	Namaste Care Family Programme	Facilitating factors and barriers that influence family caregiver involvement in the Namaste Care Family programme	**Data collection**: semi‐structured interviews and **Data analysis**: thematic analysis	Reluctance to engage in unfamiliar activities, the reluctance of family caregiver to take extra obligation, the culture of leaving everything to the nursing home, lack of time, perceived difficulty to participate, (old) age of the family caregiver, conflicts within the family and caregiver burden, feeling like a man in a woman's world, misconceptions and unclear communication, lack of skills to undertake activities, family caregivers did not feel welcome were barriers to involvement in namaste care, whereas activities they felt comfortable with, positive response from the resident to an activity, living close to a nursing home, a clear structure for Namaste are facilitators to their participation
Van Der Ploeg et al. (2012) {published data only}	Australia	Aged‐care facilities	Participants (*n* = 57); 18 staff members and 39 volunteers	Volunteers' engagement with residents with BPSD	Facilitators and difficulties of potential volunteers' involvement in non‐pharmacologic implementation	**Data collection**: semi‐structured face‐to‐face interviews and **Data analysis**: qualitative content analysis	Managing a range of personalities in group activities, being asked to perform jobs that only staff members are trained to do, residents crossing boundaries and witnessing agitation were barriers whereas staff's perceived value of volunteers, bonding between residents and volunteers, enjoyable interaction with older people perceived by volunteers and increased well‐being for resident as perceived by volunteers were facilitators of volunteers participation in the implementation of NPIs
Webster et al. (2022) {published data only}	UK	Care homes	Participants: 18 staff members	Multicomponent non‐pharmacological interventions	Barriers to managing sleep disturbances using non‐pharmacologic strategies	**Data collection**: Semi‐structured interviews and **Data analysis**: thematic analysis	Difficulty in encouraging and keeping people awake in the daytime, difficulty during the night encouraging residents to go back to their rooms, understaffing at night‐time and residents' families' complaints to staff were barriers to NPIs implementation for sleep disturbances

Abbreviations: DOCs, director of cares; LTC, long‐term care; M&M, music and memory; STA OP!, A stepwise, multidisciplinary and multicomponent intervention.

The type of NPIs were animal‐assisted intervention (Forget et al. 2021), behaviour‐oriented strategies, cognitive‐oriented strategies, stimulation‐oriented strategies, emotion‐oriented strategies (Ervin et al. 2014), Dementia Care Mapping (staff‐led, psychosocial intervention) (Griffiths et al. 2019), formulation led care (psychological approach to managing BPSD) (McKenna et al. 2022), Montessori interventions (Chaudhry et al. 2020; Ducak et al. 2018), multidisciplinary and multicomponent intervention such as food or a warm drink just before bedtime, quit and dark bedroom, reassurance through companionship, playing card games or golf (Webster et al. 2022), pet therapy (Hussin et al. 2021; Janzen et al. 2013), calming music or music therapy (Garrido et al. 2021; Hussin et al. 2021; Janzen et al. 2013; Kolanowski et al. 2010), reminiscence therapy (Hussin et al. 2021; Janzen et al. 2013) and 17 other interventions (Janzen et al. 2013), gardening, Jigsaws, flower arranging, newspaper, church service (Backhouse et al. 2016), psychosocial interventions (Lawrence et al. 2016; Miller et al. 2021; Pieper et al. 2018) and environmental treatments (Miller et al. 2021; Pieper et al. 2018), physical exercise (Hussin et al. 2021), quality of care (maximising residents' engagement and pleasure and minimising disruption to residents' natural cycle of activity and inactivity, such as sleep, walking and movement) (Cohen‐Mansfield and Meschiany 2022), recreational activities, aromatherapy and relaxation, behavioural techniques (distraction and non‐confrontational interaction) (Kolanowski et al. 2010), Music and Memory Programme (Kwak et al. 2021), Music Engagement Programme (Gulliver et al. 2021), Namaste care (gentle hand or foot massages, application of a familiar scented lotion and calming music therapy) (Kaasalainen et al. 2019; Tasseron‐Dries et al. 2021), Person‐centred care (responding to responsive behaviour in an interpersonal manner to maintain a person's dignity and personhood) (Clifford and Doody 2018 *;* Kong et al. 2022 *;* Nunez et al. 2018), volunteers engagement in provision of stimulation and company (Van Der Ploeg et al. 2012). The definition of each type of NPI identified from the 24 included studies is presented in Appendix 3. Eighteen interviews, five focus group discussions and one qualitative survey were the methods of data collection for the included studies.

#### 5.1.3 Excluded Studies

Near miss studies were excluded for different reasons including ineligible phenomenon of interest (*n* = 34), ineligible context/setting (*n* = 8), ineligible population (*n* = 25), ineligible study design (*n* = 1) and full text not available (*n* = 4) (Appendix 4).

### 5.2 Results of Methodological Limitation Assessment for the Included Studies

All except one study had a quality assessment score of at least 60% based on the JBI SUMARI quality assessment tool (Table 3). However, all of them were included regardless of the result of the quality assessment result.

**Table 3:** Critical appraisal of eligible qualitative research.

Citation	Q1	Q2	Q3	Q4	Q5	Q6	Q7	Q8	Q9	Q10	100
Griffiths et al. (2019)	U	Y	Y	Y	Y	N	U	Y	Y	Y	70
Ervin et al. (2014)	U	Y	Y	Y	Y	N	N	Y	Y	U	60
Garrido et al. (2021)	Y	Y	Y	Y	Y	N	N	Y	Y	U	70
Hussin et al. (2021)	U	Y	Y	Y	Y	N	Y	Y	Y	Y	80
Tasseron‐Dries et al. (2021)	U	Y	Y	Y	Y	N	U	Y	Y	Y	70
Lawrence et al. (2016)	U	Y	Y	Y	Y	N	N	Y	Y	Y	70
Miller et al. (2021)	U	Y	Y	N	N	N	N	N	N	Y	30
Pieper et al. (2018)	U	Y	Y	Y	Y	N	N	Y	N	Y	60
Ducak et al. (2018)	Y	Y	Y	Y	Y	N	N	Y	Y	Y	80
Kolanowski et al. (2010)	U	Y	Y	Y	Y	N	Y	Y	N	Y	70
Kaasalainen et al. (2019)	U	Y	Y	Y	Y	N	N	Y	Y	Y	70
Kwak et al. (2021)	U	Y	Y	Y	Y	N	Y	Y	Y	Y	80
Janzen et al. (2013)	U	Y	Y	Y	Y	N	Y	Y	Y	Y	80
Kong et al. (2022)	U	Y	Y	Y	Y	N	Y	Y	Y	Y	80
Gulliver et al. (2021)	U	Y	Y	Y	Y	N	Y	Y	Y	Y	80
Van Der Ploeg et al. (2012)	U	Y	Y	Y	Y	N	U	Y	Y	Y	70
Backhouse et al. (2016)	U	Y	Y	Y	Y	N	Y	Y	Y	Y	80
Forget et al. (2021)	U	Y	Y	Y	Y	N	Y	Y	N	Y	70
Clifford and Doody (2018)	U	Y	Y	Y	Y	N	Y	Y	Y	Y	80
Chaudhry et al. (2020)	U	Y	Y	Y	Y	N	U	Y	Y	Y	70
Nunez et al. (2018)	U	Y	Y	Y	Y	N	Y	Y	Y	Y	80
McKenna et al. (2022)	U	Y	Y	Y	Y	N	Y	Y	Y	Y	80
Webster et al. (2022)	U	Y	Y	Y	Y	N	N	Y	Y	Y	70
Cohen‐Mansfield and Meschiany (2022)	U	Y	Y	Y	Y	N	Y	Y	Y	Y	80
%	8.33	100.0	100.0	95.83	95.83	0.0	50.0	95.83	83.33	91.66	

*Note:* The letter denotes the rating of each study (Y = Yes, N = No, U = Unclear). JBI critical appraisal checklist for qualitative research: Q1 = Is there congruity between the stated philosophical perspective and the research methodology? Q2 = Is there congruity between the research methodology and the research question or objectives? Q3 = Is there congruity between the research methodology and the methods used to collect data? Q4 = Is there congruity between the research methodology and the representation and analysis of data? Q5 = Is there congruity between the research methodology and the interpretation of the results? Q6 = Is there a statement locating the researcher culturally or theoretically? Q7 = Is the influence of the researcher on the research and vice‐versa, addressed? Q8 = Are participants and their voices, adequately represented? Q9 = Is the research ethical according to current criteria or, for recent studies, is there evidence of ethical approval by an appropriate body? Q10 = Do the conclusions drawn in the research report flow from the analysis, or interpretation, of the data?

### 5.3 Synthesis of Results

Qualitative data from 24 published studies were extracted (Appendix 5). All identified findings were listed, regardless of whether they were supported by participants' quotes or illustrative statements. From 213 findings, 179 (84.0%) were unequivocal, 6 (3.3%) were credible and 28 (13.1%) were not supported by the illustration (Appendix 5). However, following the JBI meta‐aggregative synthesis, unsupported findings were excluded from the synthesised findings (Aromataris and Munn 2020).

#### 5.3.1 Factors Influencing the Implementation of NPIs

Various factors influencing the implementation of NPIs were identified from the included studies (Appendix 6). These include collaboration amongst care staff and families of residents with dementia, belief in the efficacy of interventions, staffing, funding, staff time constraints, familiarity with the interventions, communication among the care staff and families of residents with dementia, education and training for the care staff and families of residents with dementia, familiarity with aged‐care residents with dementia and organisational support (Appendix 6).

#### 5.3.2 Synthesised Findings

**Figure 2:**
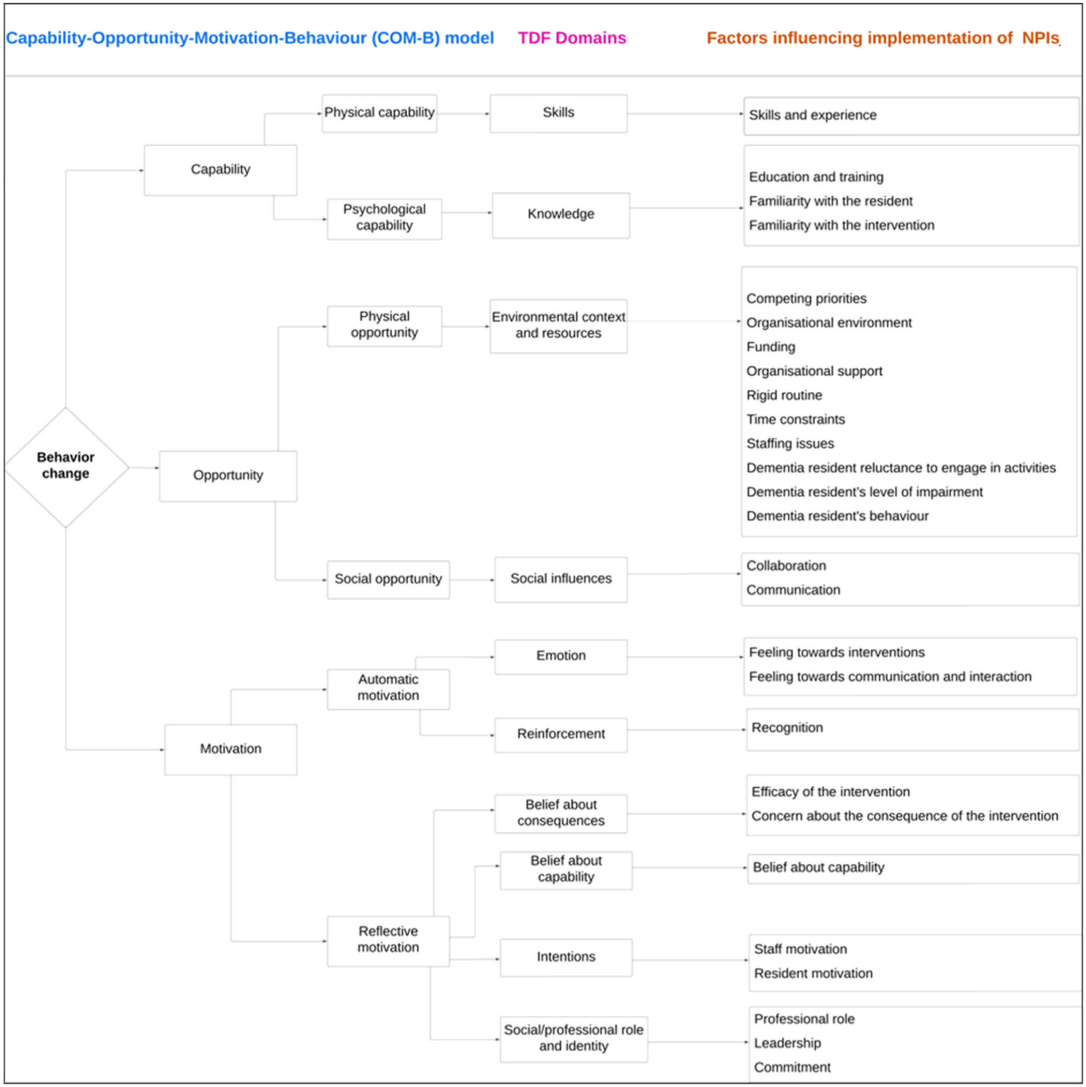
Meta‐aggregation diagram for factors influencing the implementation of non‐pharmacological interventions for managing behavioural and psychological symptoms of dementia at residential aged‐care homes.

The synthesised findings were mapped to 10 theoretical domains of the TDF: knowledge, skills, environmental context and resources, social influences, reinforcement, emotions, intentions, beliefs about consequences, social and professional roles and beliefs about capability (Appendix 6). These TDF domains were mapped to the Capability‐Opportunity‐Motivation‐Behaviour (COM‐B) model (Figure 2). The remaining TDF domains that did not capture the synthesised findings were optimism, goals, memory‐attention‐decision process and behavioural regulation (De Leo et al. 2021).

The extracted findings under each TDF category were then analysed by the primary author (H. D. A.) to produce 24 synthesised findings (13 high, 7 moderate and 4 low ConQual scores) (Table 4). Each of the 24 synthesised findings was supported by a few in‐text illustration(s) as only exemplars, and these may not be reflective of this particular finding (see Appendix 7 for more details on the quotes for each synthesised finding). In this paper, the term ‘care worker’ is equivalent to ‘caregiver’, ‘personal care worker’, ‘carer’ and these terms can be used interchangeably.

**Table 4:** CONQUal summary of synthesised findings.

TDF category	Synthesised findings	Dependability	Credibility	CONQual score	Comments
Knowledge	1. The education gap amongst the care staff and families may contribute to the slow implementation of NPI.	High	High	High	^#^Dependability: 7 findings scored 4; 1 finding scored 3. Credibility: unchanged (U = 8); hence, the ConQual is ranked as high.
	2. Familiarity of RACH care staff with aged‐care residents with dementia may facilitate the use of NPIs.	High	High	High	^#^Dependability: 9 findings scored 4; 1 study scored 3. Credibility: unchanged (U = 10); hence, the ConQual is ranked as high.
	3. Familiarity of the care staff with NPIs and effective training comprehension enhance NPI implementation.	Moderate	Moderate	Low	*Dependability: 4 findings scored 4; 5 findings scored 3. Downgraded by 1. Credibility: downgraded by 1 (U = 6, C = 3); Hence, the ConQual score is low.
Skills	4. Lack of skills and experience among the care staff may impair the implementation of NPIs.	Moderate	Moderate	Low	*Dependability: 3 findings scored 3 and 3 findings scored 4 Downgraded by 1.; Credibility: downgrade by 1 change (U = 5, C = 1). Hence, the ConQual score is ranked low.
Environmental context and resources	5. The competing demands on managers, coupled with the lack of attention from caregivers regarding the effectiveness of NPIs may impact the implementation.	Moderate	High	Moderate	*Dependability: all 3 findings scored 3. Downgraded by 1.; Credibility: Unchanged (U = 3). Hence, the ConQual score is ranked moderate.
	6. Environmental modification tailored to aged‐ care residents with dementia may enhance the implementation of NPIs.	Moderate	High	Moderate	*Dependability: 4 findings scored 2 or 3 and 2 findings scored 4 Downgraded by 1. Credibility: unchanged (U = 8). Hence, the ConQual score is ranked moderate.
	7. The lack of sufficient funds may hinder the use of NPIs.	High	High	High	^#^Dependability: 2 findings scored 3 and 6 findings scored 4; Credibility: unchanged (U = 8). Hence, the ConQual score is ranked high.
	8. The lack of an effective manager at RACHs may hinder the implementation of NPIs.	Moderate	High	Moderate	*Dependability: 5 findings scored 3 and 5 findings scored 4 Downgraded by 1.; Credibility: unchanged (U = 10). Hence, the ConQual score is ranked moderate.
	9. Lack of flexibility in daily routines could deter the use of NPIs and increase reliance on medication.	High	High	High	^#^Dependability: 2 findings scored 3 and 1 finding scored 4. Credibility: unchanged (U = 3). Hence, the ConQual score is ranked high.
	10. Lack of *adequate staff time hinders personalised NPIs*.	Moderate	Moderate	Low	*Dependability: 6 findings scored 3 and 4 findings scored 4 Downgraded by 1.; Credibility: downgrade by 1 change (U = 9, C = 1). Hence, the ConQual score is ranked low.
	11. Lack of well‐trained, qualified and stable staff, could be a barrier to the use of NPIs.	High	High	High	^#^Dependability: 5 findings scored 3 and 8 findings scored 4.; Credibility: unchanged (U = 13). Hence, the ConQual score is ranked high.
	12. Resident reluctance to engage in activities hinders NPI implementation.	Moderate	Moderate	Low	*Dependability: 3 findings scored 3 and 1 finding scored 4 Downgraded by 1.; Credibility: downgrade by 1 change (U = 3, C = 1). Hence, the ConQual score is ranked low.
	13. The severity of the resident's physical and cognitive impairment as well as comorbid conditions may slow the use of NPIs.	High	High	High	^#^Dependability: 2 findings scored 3 and 3 findings scored 4. Credibility: unchanged (U = 5). Hence, the ConQual score is ranked high.
	14. The severity of the behaviour amongst aged‐care resident with dementia could make it difficult to implement NPIs.	Moderate	High	Moderate	*Dependability: 3 findings scored 3 and 2 findings scored 4 Downgraded by 1.; Credibility: unchanged (U = 5). Hence, the ConQual score is ranked moderate.
Social influences	15. Collaboration among care staff, volunteers and families of aged‐ care residents with dementia may influence the implementation of NPIs.	High	High	High	^#^Dependability: 10 findings scored 3 and 12 findings scored 4. Credibility: unchanged (U = 22). Hence, the ConQual score is ranked high.
	16. The quality of communication among staff, between staff and aged‐care residents with dementia and between staff and families may influence the implementation of NPIs.	High	High	High	^#^Dependability: 3 findings scored 3 and 6 findings scored 4. Credibility: unchanged (U = 9). Hence, the ConQual score is ranked high.
Reinforcement	17. Lack of recognition for the work of caregivers and allied health professionals could slow the implementation of NPIs.	High	High	High	^#^Dependability: both findings scored 4 Credibility: unchanged (U = 2); Hence, the ConQual score remains high.
Emotions	18. Feeling of staff resistance, fear from families, misperceptions and feeling overwhelmed hinder NPI implementation, while resident preference alignment and ownership enhance its implementation.	High	High	High	^#^Dependability: 3 findings scored 3 and 4 findings scored 4. Credibility: unchanged (U = 7). Hence, the ConQual score is ranked moderate.
	19. Volunteers' frustration with staff communication, care staff's sense of undervalued knowledge and experience and residents' uncooperative behaviour hinder NPI implementation, while staff empathy enhances its implementation.	High	High	High	^#^Dependability: 2 findings scored 3 and 6 findings scored 4; Credibility: unchanged (U = 8). Hence, the ConQual score is ranked high.
Intentions	20. The facility manager and staff buy‐in are critical in the implementation success of NPIs.	Moderate	High	Moderate	*Dependability: 3 findings scored 3 and 2 findings scored 4 Downgraded by 1.; Credibility: unchanged (U = 5). Hence, the ConQual score is ranked moderate.
Belief about consequences	21. The care staff's belief in the benefit of NPIs and families seeing their effectiveness could enhance their implementation.	High	High	High	^#^Dependability: 6 findings scored 3 and 12 findings scored 4; Credibility: unchanged (U = 18). Hence, the ConQual score is ranked high.
	22. The care staff's concern about consequences of NPI use could impair their implementation.	High	High	High	^#^Dependability: 1 finding scored 3 and 3 findings scored 4; Credibility: unchanged (U = 4). Hence, the ConQual score is ranked high.
Social/professional role and identity	23. The lack of intervention ownership amongst the staff and role mismatching may impair the implementation of NPIs.	Moderate	High	Moderate	*Dependability: 5 findings scored 3 and 3 findings scored 4 Downgraded by 1.; Credibility: unchanged (U = 8). Hence, the ConQual score is ranked moderate.
Belief about capability	24. The lack of trust by care staff in psychologists and the loss of hope among families regarding their loved one's participation in activities could deter NPI implementation.	Moderate	High	Moderate	*Dependability: 1 finding scored 3 and 1 finding scored 4 Downgraded by 1.; Credibility: unchanged (U = 2). Hence, the ConQual score is ranked moderate.

*Note:* U: unequivocal; C: credible; N: not supported by illustration. * = Dependability downgraded by 1 if greater or equal to 50% of findings scored 2 or 3 ‘yes’ responses; # = Dependability remained unchanged, majority of findings scored 4 or 5 ‘yes’ responses. ConQual criteria for assessing confidence. The following five questions confirm the dependability of the results. (1) Is there congruity between the research methodology and the research question or objectives? (2) Is there congruity between the research methodology and the methods used to collect data? (3) Is there congruity between the research methodology and the representation and analysis of data? (4) Is there a statement locating the researcher culturally or theoretically? (5) Is the influence of the researcher on the research and vice versa, addressed?

##### 5.3.2.1 COM‐B Category: Capability – Psychological Capability

###### 5.3.2.1.1 TDF Domain 1: Knowledge

Thirty‐one findings were categorised in the knowledge domain that informed three categories (Appendix 6). These categories include education and training, familiarity with the resident and familiarity with the intervention. These three categories informed synthesised findings 1–3 (Table 4).

*Synthesised Findings 1–3 Mapped to the knowledge domain.*

*Synthesised Finding 1: The education gap amongst the care staff and families may contribute to the slow implementation of NPIs.*

The implementation of NPIs may be facilitated if annual continuing education and short‐term training for the care staff incorporated courses on the causes and management of changed behaviours in dementia. In addition, bridging the gap between theoretical knowledge and practice may be beneficial. For example, physicians, certified nursing assistants and nurses highlighted that the current education format lacks adequate training. Finally, providing families of residents with dementia with education on dementia and its management may further support effective implementation (Clifford and Doody 2018; Ervin et al. 2014; Kolanowski et al. 2010; Kong et al. 2022).

‘I don't think that new Certified nursing assistants (CNAs) come out of class knowing how to deal with combative, aggressive, or apathetic residents’. One participant stated, ‘…they don't seek to understand the behaviour; they just try to address it and I think that's when you come up on failure because you don't really understand what's causing that behaviour’. Physicians, CNAs, and nurse participants echoed that education ‘is just something that's been missing in our educational format’. (p. 5 The Educational Needs of Staff, paragraph 1)

(Kolanowski et al. 2010)

‘Much of the contents of continuing education focus on acute hospital care. I think we really need this kind of education, person‐centred dementia care, in annual continuing education (*Participant 12, Care worker*) (p. 5 Staff's lack of education, paragraph 1)’ … ‘Families seem to need education about dementia and person‐centred care. Through the education, they will be able to understand dementia of their loved one and the benefits of person‐centred care (*Participant 12, Care worker*) (p. 6 Family's lack of education, paragraph 2)’ … ‘Some educations are very theoretical. There is a gap between theory and practice (*Participant 8, Care worker*)’ (Kong et al. 2022). ‘Nurses need more training in behaviour management’. ‘The skill mix of the team is sometimes a problem’ (p. 4, Behaviour oriented strategies in dementia care, paragraph 3) (Ervin et al. 2014). ‘It definitely would pay off for anyone working in dementia care to have some training in dementia and top up session, definitely a course or some documentation that they could sit down and actually read, just to understand what might be going on’ (*P9, General nurse*), (p. 6 The impact of education on nursing practice, paragraph 3)

(Clifford and Doody 2018)

In addition, there is a sense of frustration and scepticism about the feasibility of training for caregivers at RACHs. For example, an activity coordinator reported that it is difficult to consider caregiver training due to work overload (Clifford and Doody 2018; Cohen‐Mansfield and Meschiany 2022; Hussin et al. 2021).

‘Knowledge with nursing staff is at a level where they can understand, however, for the caring staff, mainly their knowledge isn't the same, some of them have attended training, sometimes their knowledge lacks in ways that it makes it hard for them to respond to responsive behaviours or try to deal with a situation’ (*P9, Genera nurse*). (p. 7, The impact of education on nursing practice, paragraph 4) (Clifford and Doody 2018). ‘Training the caregiver team, it is naive to think that will happen. In the afternoon there is a heavy silence when there is no activity. They don't get enough compensation, so asking [staff] to do anything extra [e.g., undergoing training, conducting activities], is a joke’ (*#3, activity worker*). (p. 5 Institutional barriers, paragraph 4)

(Cohen‐Mansfield and Meschiany 2022)

*Synthesised Finding 2: Familiarity of RACH care staff with aged‐care residents with dementia may facilitate the use of NPIs.*

The use of NPIs may be easier when caregivers know the residents well and maintain accurate records of their preferences. Additionally, remaining calm, devoting adequate time and connecting with them in a way that respects their dignity and independence could help residents feel respected and receptive to the interventions. For example, one general nurse highlighted the need for caregivers to manage their behaviour, be calm and respect the personal space and unspoken desires of the individuals they care for, even when those individuals cannot clearly express what they want or do not want (Clifford and Doody 2018; Garrido et al. 2021; Janzen et al. 2013; Kolanowski et al. 2010; Nunez et al. 2018).

‘You need to get to know the person, to know their life, their history, to know everything about them, what they worked at, what they like to eat, their family, all their likes and dislikes’ (*P5, Psychiatric nurse*). (p. 6 Resources and interventions to support people with dementia and responsive behaviour, paragraph 5). (Clifford and Doody 2018). Yeah, I had a resident who was a farmer and you know very withdrawn and … not engaged in life. Had lost meaning and purpose but by engaging him in therapeutic activities of just gardening that allowed him to continue to explore and to give him a sense of identity again. (p. 4, Reaching Out to the Person with Dementia, paragraph 2) (Kolanowski et al. 2010). ‘You have to be calm, you have to monitor your own behaviour, you have to respect their space, what they want, what they don't want regardless of the fact that they might not be able to express it’ (*P6, General nurse*). (p. 7 The care environment, paragraph 5) (Clifford and Doody 2018). A recreation coordinator explains ‘…the more time you [staff] spend with them [residents], the more you figure out what works. So, consistency in staff and routine for them [residents] is [a] big [factor]’ (*Recreational coordinator*). (p. 6 Facilitators and Barriers for NPI Implementation in LTC, paragraph 1)

(Janzen et al. 2013)

*Synthesised Finding 3: Familiarity of the care staff with NPIs and effective training comprehension may enhance NPI implementation.*

Familiarity of the care teams (nurses, managers, personal care workers or caregivers and others in the team) with the intervention, as well as their ability to comprehend and communicate the knowledge gained during training about NPIs will enhance their implementation (Ducak et al. 2018; Griffiths et al. 2019; Gulliver et al. 2021; Kolanowski et al. 2010; McKenna et al. 2022; Tasseron‐Dries et al. 2021).

One staff member believed that since they already conducted musical sessions, they would not find it difficult (Kolanowski et al. 2010). ‘I still don't understand it … no one has been able to understand it to me fully…’ (*Manager*) (p. 10) … ‘The trouble is, when they (staff) came back [from the training], they weren't able to explain properly what they had to do’ (*Staff member*) (Griffiths et al. 2019) … when I brought home all of the resource materials from the course I sat down with my staff and we … went through each one as to how it would benefit or how they would explain it to other staff if they said, well, you know, ‘Why are you scooping golf balls into a muffin tin?’ So, we rationalised … ‘OK, that motion will maintain dexterity so that they can continue to feed themselves’. So that makes sense to a nurse, to a personal support worker (PSW), so because there's always that thought, ‘Well, this is childish or this is not appropriate’. Or that type of thing (*Recreation manager*). (p. 17, enabling factors, paragraph 2)

(Ducak et al. 2018)

##### 5.3.2.2 COM‐B Category: Capability – Physical Capability

###### 5.3.2.2.1 TDF Domain 2: Skills

Eight findings were categorised in the *skills* domain that informed three categories (Appendix 6). These include lack of experience, lack of skills and difficulty of intervention individualisation. These three categories informed synthesised finding 4 (Table 4).

*Synthesised Finding 4 mapped to the skills domain.*

*Synthesised Finding 4: Lack of skills and experience amongst the care staff may impair the implementation of NPIs.*

The staff's lack of experience and skills to tailor interventions to individual needs, inability to communicate in a language that the resident understands, inability to explain what is going to be done and failure to discuss the care plan and care needs of the resident with their family may hinder the implementation of NPIs (Clifford and Doody 2018; Cohen‐Mansfield and Meschiany 2022; Ervin et al. 2014; Garrido et al. 2021; Griffiths et al. 2019; Kolanowski et al. 2010).

‘I have no experience’, ‘Lack of experience’ (p. 4, Cognitive oriented strategies in dementia care, paragraph 3) (Ervin et al. 2014). ‘It's not just a case of having music. It's being able to hear the type of music that that person can relate to’ (*P2 Group 3, Carer husband*) (p. 8, Challenges to Implementing Music Programs in Aged Care, paragraph 9) (Garrido et al. 2021). ‘How to achieve communication [is a need]. Are there any tools that can be given to therapists? Warmth, smiles? Most employees do not have the skills to do so’ (*P21, Chief Executive Officer, CEO*). (p. 4 Concerns related to other staff, paragraph 1)

(Cohen‐Mansfield and Meschiany 2022)

##### 5.3.2.3 COM‐B Category: Opportunity – Physical Opportunity

###### 5.3.2.3.1 TDF Domain 3: Environmental Context and Resources

Seventy‐seven findings were categorised in the *environmental context and resources* domain that informed twelve categories (Appendix 6). These include competing priorities, organisational environment, funding, organisational support, rigid routine, time constraints, under‐staffing, resident reluctance to engage in activities, level of impairment and resident's behaviour. These 10 categories informed synthesised findings 5–14 (Table 4).

*Synthesised Findings 5–14 mapped to the environmental context and resources domain.*

*Synthesised Finding 5: The competing demands on managers, coupled with the lack of attention from caregivers regarding the effectiveness of NPIs may impact the implementation.*

Managers should find ways to overcome the competing demands on top of NPIs (e.g., dementia care mapping). In addition, caregivers should prioritise NPIs over other competing activities and value the effectiveness of NPIs. For example, a music therapist pointed out that caregivers often treat the music as background noise, not considering whether it is beneficial or detrimental to the resident's well‐being (Garrido et al. 2021; Griffiths et al. 2019).

‘We get inspected by health and safety, infection control, the social workers, CQC [regulatory authority] come, social services come, y'know, it's just ongoing and they are all asking for more paperwork … we are struggling to do the paperwork that we have got already’. (*Manager*) (p. 6) (Griffiths et al. 2019). ‘I've had situations where there's just music on in the background and a lot of our carers, they're not conscious of it. They're not thinking of “how is music being effective or not effective in this situation,” because for them they can shut it out. It's just background noise. Whereas it might be really important for the resident. There needs to be something built into the cycle around monitoring it, so it really brings it into the consciousness’. (*P3 Group 1, Music therapist*) (P6, Challenges to Implementing Music Programs in Aged Care, paragraph 4)

(Garrido et al. 2021)

*Synthesised Finding 6: Environmental modification tailored to aged‐care residents with dementia may enhance the implementation of NPIs.*

The provision of NPIs for BPSD may need making changes to the physical surroundings to accommodate the unique requirements of the aged‐care residents with dementia. For example, a separate dementia‐specific unit, a dedicated storage room for equipment and ample natural light outside (Ducak et al. 2018; Ervin et al. 2014; Kong et al. 2022; Miller et al. 2021).

‘Residents with behaviours disrupt all residents in the facility and should be in dementia specific units’. (p. 5, Overall comments regarding management of BPSD, paragraph 5) (Ervin et al. 2014). ‘We had a challenge trying to figure out how best to store them (headphones) safely while also providing good access to everybody’ (*P6 Group 2, Lifestyle manager*). (p. 8, Challenges to Implementing Music Programs in Aged Care, paragraph 8) (Garrido et al. 2021). Multiple sites reported using natural light indoors by opening window blinds, and one staff stated, ‘[resident's] mood is better when it's bright and sunny outside (*Staff*)’. (p. 4 Natural light, paragraph 1) (Miller et al. 2021). We're just dealing with a primarily medical environment, right? (*R12, Recreational manager*)

(Ducak et al. 2018)

*Synthesised Finding 7: The lack of sufficient funds may hinder the use of NPIs.*

Lack of adequate funds for training, equipment, activities, resources and appropriate salaries for the care staff may impair the implementation of NPIs (Clifford and Doody 2018; Cohen‐Mansfield and Meschiany 2022; Ducak et al. 2018; Forget et al. 2021; Kaasalainen et al. 2019; Lawrence et al. 2016).

‘I think some of our barriers here are more financial barriers because I think it would be beneficial to send more staff to the workshop but financially that's not feasible’ (participant, *R4, Manager/supervisor*) (p. 13 limiting factors, paragraph 11) (Ducak et al. 2018). ‘When asked what they would like to change, staff consistently responded “salary” (e.g., *22, social worker*)’ (Cohen‐Mansfield and Meschiany 2022). ‘…it (the programme) finished because of the cost of the programme and the cost of care is not reflected in the fees that are negotiated through the National Treatment Purchase Fund’ (*P8, General nurse*) (p. 6 Resources and interventions to support people with dementia and responsive behaviour, paragraph 3)

(Clifford and Doody 2018)

*Synthesised Finding 8: The lack of an effective manager at RACHs may hinder the implementation of NPIs.*

Lack of a strong, committed, well‐aware and open‐ended manager who avoids scapegoating is open to being challenged positively by the care staff and leads the intervention could impair the smooth application of NPIs (Backhouse et al. 2016; Clifford and Doody 2018; Ducak et al. 2018; Forget et al. 2021; Griffiths et al. 2019; Kaasalainen et al. 2019).

‘Nursing management needs to be on board, they need to understand, they wouldn't understand what it is you are trying to do for the patient, I don't think they understand why you are harping on about the compliment of staff and why you are constantly looking for (additional resources)’ (*P5, Psychiatric nurse*) (p. 7, The care environment, paragraph 4) (Clifford and Doody 2018). ‘It's mainly from a confidence perspective, [they] were clearly not confident to challenge a manager who was not supporting’. (*DCM expert*) (p. 7, Griffiths et al. 2019). ‘I think management support, you know, it can either be amazing when it's amazing or it can be a real difficulty if the manager isn't supportive’. (*DCM expert*) (p. 7)

*Synthesised Finding 9: Lack of flexibility in daily routines could deter the use of NPIs and increase reliance on medication.*

Lack of well‐established individualised routines, such as mealtime, bathing time, personal care and sleep time, hinder the adoption of NPIs (e.g., Montessori interventions). Additionally, the lack of targeting at triggers of behaviours (e.g., sleeplessness) and the absence of adjustment to sleep time based on individuals' routines could increase the use of medications (Cohen‐Mansfield and Meschiany 2022; Ducak et al. 2018).

‘There's such a routine, a rigid routine, like meals are this time and bath is this time and, personal care is this time’ (*R2, Assistant*) (p. 13 limiting factors, paragraph 1) (Ducak et al. 2018). For example, some residents were given sleeping pills to adjust their schedule to the majority: ‘[…] There is an hour when the majority goes to sleep with some given sleeping pills [only] to [to be] awoken at early hours in the morning’ (*#14, Chief Executive Officer, CEO*). (p. 4, Concerns related to residents, paragraph 5) (Cohen‐Mansfield and Meschiany 2022). ‘Behaviours are presented but they're looked at as behaviours that need to be treated rather than looking at what causes these behaviours. consultant’ (*C2, Regional manager/educator*) (p. 13, limiting factors, paragraph 4)

(Ducak et al. 2018)

*Synthesised Finding 10: Lack of adequate staff time hinders personalised NPIs.*

Lack of sufficient staff time could slow the implementation of individualised NPIs as it may result in some residents being overlooked (Ducak et al. 2018; Ervin et al. 2014; Garrido et al. 2021; Griffiths et al. 2019; Gulliver et al. 2021; Kaasalainen et al. 2019; Kong et al. 2022; Kwak et al. 2021; Lawrence et al. 2016).

‘Time limitations, not enough time and staff to cover for 1:1 time for patientsʼ. ‘Staff numbers and time taken to use this strategy (behaviour‐oriented strategy) is a major limitation’. ‘1:1 time. I feel others miss out while time is devoted to the person with behaviourʼ (p. 4, Behaviour oriented strategies in dementia care, paragraph 2) (Ervin et al. 2014). Several staff members believed insufficient time was a significant barrier to conducting these types of activities (music engagement) – ‘No … they're focused on getting their care work done … to do something like that you need someone that's going to sit there with them’ (*S2, Staff*). (p. 7 Issues in continuing the program. Paragraph 1)

(Gulliver et al. 2021)

*Synthesised Finding 11: Lack of well‐trained, qualified and stable staff could be a barrier to the use of NPIs.*

The absence of a sufficient number of well‐trained and qualified permanently employed nurses, caregivers and other staff members, as well as volunteers could be a barrier to the use of NPIs. Staff turnover may cause staff burnout due to high workload leading to inadequate implementation of NPIs (Chaudhry et al. 2020; Clifford and Doody 2018; Cohen‐Mansfield and Meschiany 2022; Ducak et al. 2018; Forget et al. 2021; Griffiths et al. 2019; Hussin et al. 2021; Janzen et al. 2013; Kaasalainen et al. 2019; Kong et al. 2022; Kwak et al. 2021; Nunez et al. 2018; Webster et al. 2022).

An RN described the typical situation, ‘…at times there's so little staff and there's a lot of behaviours all at once. It's just kind of putting out fires and keep things rolling… (*Registered nurse*)’ (p. 6 Facilitators and Barriers for NPI Implementation in LTC, paragraph 3) (Janzen et al. 2013). ‘There are almost no permanent employees, high degree of burnout, many employees have to work in additional places’ (*#14, Chief Executive Officer, CEO*). (p. 5 Institutional barriers, paragraph 2) (Cohen‐Mansfield and Meschiany 2022). ‘We haven't got nurses and staff, so my staff aren't that confident anyway … I'm glad we got involved because we got a lot out of it, I'm just disappointed that we weren't able to continue.’ (*Manager*) ‘Care homes are really, really busy. Turnover of staff in care homes can be quite dramatic at times’ (DCM expert)

(p. 5, Griffiths et al. 2019)

*Synthesised Finding 12: Resident reluctance to engage in activities hinders NPI implementation.*

Challenges faced by caregivers in engaging individuals with advanced dementia in activities, such as difficulties in keeping them awake during the day and overcoming initial resistance to participation lead to slow implementation of NPIs (Backhouse et al. 2016; Tasseron‐Dries et al. 2021; Webster et al. 2022).

‘We try to keep him awake but it's no chance. You can't. He just sit[s] down and he's too tired and he closes his eyes’. (*1; female care assistant*) ‘Earlier on this month there was a time he was so tired he would just eat a piece of his breakfast and go to bed and sleep. We try as much to keep him busy during the day but he's quite a strong headed person’. (*3; female team leader*) (p. 5 Evening strategies to promote sleep, paragraph 1) (Webster et al. 2022). ‘Holly, an activity worker, touches on the issue: I have to try and get them … say “oh come on, do you want to do it?” “no, no, no” “come on” but once they're doing it they're absolutely fine, it's like when we done all the sunflowers … Mable was going “oh I can't do that, I can't draw” but … she absolutely loved it in the end’. (*Holly, Activity Worker, CH2*) (p. 5 Barriers to including residents in activities, paragraph 9)

(Backhouse et al. 2016)

*Synthesised Finding 13: The severity of the resident's physical and cognitive impairment as well as comorbid conditions may slow the use of NPIs.*

Residents with severe cognitive impairment, physical incapability and poorly managed comorbid conditions before being transferred to a nursing home are less likely to participate in activities (Backhouse et al. 2016; Ervin et al. 2014; Griffiths et al. 2019; Kolanowski et al. 2010).

‘They don't keep residents in the hospital a long time. So, you know you're dealing with a lot of medical things. That unfortunately has to be our priority and the poor resident that's here with dementia is sort of left behind. (Pg 3 The Changing Landscape, paragraph 2)’ (Kolanowski et al. 2010). ‘Because we are only a residential home, erm, y'know, “…some of our residents are quite poorly so it (dementia care mapping) doesn't work for them, it just depends how well they are.”’ (*Staff member*) (p. 8, Griffiths et al. 2019). A lot of the elderly people forget what they can and cannot do. So many of them think they can walk. They want to be independent and they're not able to because of physical disabilities. So, we're trying to keep them safe, but I think at times they feel like we're trying to hold them back and which will aggravate them even more. (p. 4 resident behaviours, paragraph 3)

(Kolanowski et al. 2010)

*Synthesised Finding 14: The severity of the behaviour amongst aged‐care residents with dementia could make it difficult to implement NPIs.*

The resident's aggressiveness (e.g., as a result of incompatibility or disagreement between caregivers and residents with dementia), mood swings and tendency to cross boundaries with volunteers make it difficult to provide tailored NPIs (Chaudhry et al. 2020; Clifford and Doody 2018; Kolanowski et al. 2010; Van Der Ploeg et al. 2012).

‘Yes, they do have a lot of mood swings. At one moment they are very cooperative, but in the next they become totally opposite, like at one moment they agreed to take a bath, but when they were taken to the washroom, they started beating us. Their behaviour changes so abruptly’ (*PCW2, Paid care worker*). (p. 5 table 2 Experience of working with older adults) (Chaudhry et al. 2020). ‘We are used to dealing with people that strip or call out, or spit, but if it's ongoing, if it's constant every day, that puts too much pressure on and it's stressing for them and other residents’ (*P3, Intellectual disability nurse*). (Clifford and Doody 2018). ‘Like if you give out your phone number, not only are you opening yourself up to get random phone calls when you're not in your volunteer role. I suppose it's not appropriate to get to that, to keep within those bounds and just remember your place and that you're not a family member and you're not a doctor. (*#18, Volunteer*) (p. 5–6 Perceived benefits and difficulties, paragraph 11)’ (Van Der Ploeg et al. 2012). Sometimes we have an all‐black crew, and this person does not like black people and is saying don't let that ‘N’ touch me … we cannot possibly say well we'll get a white certified nursing assistant (CNA) for you. So, you just try to assure them that, that person is there to help them (p. 4 The Changing Landscape, paragraph 2)

(Kolanowski et al. 2010)

##### 5.3.2.4 COM‐B Category: Opportunity – Social Opportunity

###### 5.3.2.4.1 TDF Domain 4: Social Influences

Thirty‐six findings were categorised in the *social influences* domain that informed two categories (Appendix 6). These include collaboration and communication. These two categories informed synthesised findings 15 and 16 (Table 4).

*Synthesised Findings 15 and 16 mapped to the social influence domain.*

*Synthesised Finding 15: Collaboration amongst care staff, volunteers and families of aged‐care residents with dementia may influence the implementation of NPIs.*

The lack of collaboration amongst the care staff, between day and night shift staff and between family and staff and volunteers, could make it challenging to implement NPIs (Clifford and Doody 2018; Ducak et al. 2018; Griffiths et al. 2019; Hussin et al. 2021; Kong et al. 2022; Lawrence et al. 2016; McKenna et al. 2022; Nunez et al. 2018; Pieper et al. 2018; Van Der Ploeg et al. 2012; Webster et al. 2022).

One time we wanted to place a bedridden resident with dementia in a wheelchair and take her for a walk, but some staff disagreed. So, we could not do it. Although we were co‐workers, our opinions were often different. (*Participant 10, care worker*) (p. 7 Conflicts among/between staff and families, paragraph 1)

(Kong et al. 2022)

‘Do not worry about it, the day staff will be in a minute. It's always like this battle [laughter]. That's what we need to stop; we need to work as one, as opposed to working as a battle against each other’ (Nunez et al. 2018). ‘She just ate but she forgot, and she tells her daughter that she did not eat. The daughter came and scolded me for not feeding her. I snapped a picture of her eating and “WhatsApp” the daughter.’ (*Yona, caregiver*) (Hussin et al. 2021). So, the nurse told us that we should reduce the wine in the daytime. Because it's making him sleep less in the night. But when his children come [to visit], her daughter, when she comes, she brings some from the house. (*16; female care assistant*)

(Webster et al. 2022)

On the other hand, the passion of the staff for change, collaboration amongst the staff, between family and staff, teamwork between allied health professionals and caregivers, fostering good relationships between caregivers and residents' relatives may be the facilitators of NPI implementation (Clifford and Doody 2018; Ducak et al. 2018; Griffiths et al. 2019; Hussin et al. 2021; Lawrence et al. 2016; McKenna et al. 2022; Pieper et al. 2018; Van Der Ploeg et al. 2012).

We all work as a cogina wheel and if one of those cogs breaks then the wheel doesn't turn, does it? So, what we do is we all work together it's like they work upstairs with the carers and if something is wrong, they report here and then it gets reported to the doctor … That is the heart of the person‐centred care because if we don't have that we won't know the person's needs. It won't be met without us knowing. (*3001*) (p. 4 table 1, (2c) Relationships within the team) (Lawrence et al. 2016). ‘While we have an activity coordinator, we also do dementia specific activities, we do reminiscence, relaxation therapy, and we give each other feedback to say they enjoyed it’ (*P9, General Nurse*) (Clifford and Doody 2018). ‘We do run into who pays for it. In this case though, the families have never had a problem with that because really, it's never very expensive, it's usually less than 20 dollars, and they, the families, love the individualised attention’. *Consultant (C4)*

(Ducak et al. 2018)

*Synthesised Finding 16: The quality of communication amongst staff, between staff and aged‐care residents with dementia and between staff and families may influence the implementation of NPIs.*

Communication amongst staff members about the resident's care plan, promoting communication between caregivers and residents of the same ethnic and linguistic background, engaging in slow and attentive conversation with eye contact with the resident the use of elderspeak when necessary and ensuring effective communication between, staff and aged‐care residents with dementia may promote the implementation of NPIs (Forget et al. 2021; Hussin et al. 2021; Kong et al. 2022; Nunez et al. 2018; Van Der Ploeg et al. 2012).

‘Staff have to know the care plan of the residents and do they miss out on days, because of days off.’ *CSFG 2* ‘so although I did ask the respite care manager to let me know whether they administer her (resident) any sleeping medication or to keep her agitation down, they never did—’ *FCFG 2* (p. 5, table 1) (Nunez et al. 2018). ‘I changed her caregiver to be with the same ethnicity. As Indian prefers to be with an Indian to take care of them because they can talk in the same language.’ (Gaya, caregiver) (Hussin et al. 2021). ‘We have to slow talk with them while making eye contact and body language. At that moment they will express their feeling, maybe they want something. From there, we are able to identify what is their need.’ (*Akim, caregiver*) (p. 9 Strategy: Verbal and non‐verbal communication paragraph 1) (Hussin et al. 2021). ‘Yes, we treat them as a baby and so far, none has become angry because we treat them like that. They in fact like it, I don't know … maybe they like the tone of our voice when we call them’ (*Catherine, caregiver*) (p. 10 Elderspeak paragraph 1). ‘If they are still aggressive, we just speak gently to them and never oppose whatever they said at that time. If they think that their children will pick them in the evening, we just have to agree with them even if it's not going to happen’ (*Akim, caregiver*)

(Hussin et al. 2021)

On the other hand, a lack of effective communication between staff and families could impair the implementation of individualised NPIs (Kong et al. 2022; Tasseron‐Dries et al. 2021).

‘Some families are very sensitive, so it is better put some distance between. They misunderstand my words and then they go to the office of administrator and complain about that. So, it is very difficult to communicate with them’. (*Participant 8, care worker*). ‘When families institutionalise their loved ones, they do not share one hundred percent of the information about their loved ones. They seem to be afraid that their loved ones will be rejected [for admission] by the nursing home. Families do not tell us details about their loved ones, which hinders our implementation of person‐centred care for residents with dementia’ (*Participant 19, NA, Nurse's aide*)

(Kong et al. 2022)

Additionally, volunteers' loneliness in terms of their gender may demotivate them from participation in the implementation of NPIs.

I was the only man, you know. But, so, on Wednesday there are two male volunteers. That is really good…. (*Family caregiver, spouse*) (p. 6 ‘Personal circumstances’: Personal context of family caregivers, paragraph 4)

(Van Der Ploeg et al. 2012)

##### 5.3.2.5 COM‐B Category: Motivation – Automatic Motivation

###### 5.3.2.5.1 TDF Domain 5: Reinforcement

Two findings were categorised in the *reinforcement* domain that informed one category, which is recognition (Appendix 6). This category informed synthesised finding 17 (Table 4).

*Synthesised Finding 17 mapped to the reinforcement domain.*

*Synthesised Finding 17: Lack of recognition for the work of caregivers and allied health professionals could slow the implementation of NPIs.*

Implementation of NPIs may not be likely to be successful if caregivers and allied health professionals don't feel appreciated and supported by the government, residents' families and the community (Kwak et al. 2021; Lawrence et al. 2016).

So, they [the government] really have to recognise that the care workers are doing a highly skilled, professional job, they don't take it seriously. Even when I am out there and somebody asks me, ‘what are you doing?’ you know a care job and the way people, even the way that the relatives look at you because you are doing this job, you can't win. And they can't do it. So, really, I feel that they don't recognise the care job is a good thing, they think we just come here to wash somebody, but that is not what we do. (*2004*) (p. 3 table 1 (1a) Lack of recognition) (Lawrence et al. 2016). My concern as a music therapist is that facilities will think they can just throw headphones on seniors to give them music, which could in turn devalue the work of a music therapist. (p. 7 Value of M&M., paragraph 3)

(Kwak et al. 2021)

##### 5.3.2.6 COM‐B Category: Motivation – Automatic Motivation

###### 5.3.2.6.1 TDF Domain 6: Emotion

Fifteen findings were categorised in the *emotion* domain informing two categories (Appendix 6). These include feelings towards intervention and feelings towards communication and interaction. These two categories informed synthesised findings 18 and 19 (Table 4).

*Synthesised Findings 18 and 19 mapped to the emotion domain.*

*Synthesised Finding 18: Feeling of staff resistance, fear from families and feeling overwhelmed hinder NPI implementation, while resident preference alignment and ownership of NPI enhance its implementation.*

Feeling resistance from staff about the intervention, fear of families about the intervention, misperception of the word intervention as a correction of something wrong and feeling scared with the intervention (e.g., staff frightening of letting their voice out) are hindrances whereas matching between the preferences of residents and the interventions and sense of ownership of the intervention by the resident are enhancers of NPIs implementation (Forget et al. 2021; Garrido et al. 2021; Griffiths et al. 2019; Gulliver et al. 2021; Kwak et al. 2021; Lawrence et al. 2016; Tasseron‐Dries et al. 2021).

‘I felt that there was a reluctance to look at that. And there was quite a lot of defensive response’. (*DCM expert*) (p. 7–8, Griffiths et al. 2019). ‘The downside is for families who are afraid of dog when they see one, but it's quite rare’; ‘there are people who don't like dogs, or even fear them’ (Forget et al. 2021). As soon as you say we are having an intervention, it's like what you have done wrong needs to be assessed and then we are going to better it through our intervention and we are going to intervene in activities, we are going to intervene in this and this. And to me it's more of an association with us, working with us to do these things and helping to guide whereas intervention sounds like we have done something wrong. (*2004*) (p. 3 table 1 (1a) Lack of recognition) (Lawrence et al. 2016). Staff may be ‘a little bit frightened of letting their voice out and … because it is a daunting thing’ (*S2*), and that they felt you had to have ‘a good voice’ (*S3*). (p. 7 Issues in continuing the program. Paragraph 1) ‘They liked hearing all their favourite songs back‐to‐back. Many will smile when we put it on them. They like knowing that they have their own music that they don't have to share.’ (p. 8 What residents liked about M&M, paragraph 1)

(Kwak et al. 2021)

*Synthesised Finding 19: Volunteers' frustration with staff communication, care staff's sense of undervalued knowledge and experience and residents' uncooperative behaviour hinder NPI implementation, while staff empathy enhances its implementation.*

Volunteers' frustration with staff communication, the sense that care staff knowledge and experience are undervalued in the view of the expert (e.g., psychologist), caregivers' feeling that residents are uncooperative, restless and shouting is hindrance to NPIs implementation, while staff empathy for residents enhances their implementation (Backhouse et al. 2016; Cohen‐Mansfield and Meschiany 2022; Ducak et al. 2018; Janzen et al. 2013; Kolanowski et al. 2010; Lawrence et al. 2016; McKenna et al. 2022; Tasseron‐Dries et al. 2021).

‘I think it would be prudent to say, of course you can visit, but please remember that the Namaste program is underway and please slow down, relax. Yes, exactly. That it works differently. That you don't put up barriers in advance, like, well it's Namaste, so we'd better not visit then.’ (*Family caregiver, daughter*) (p. 7 ‘Communication’: Communication between family caregiver, staff and volunteer, paragraph 3) (Tasseron‐Dries et al. 2021). Work on the floor where she comes, that's my, the floor I work on, and she hasn't spoken to me once … I don't feel like I can approach them, I wouldn't approach them to ask them, any information … like I say they don't really acknowledge, they just come in, do their job and then they go … [*Participant 2*] (p. 5 Theme 2: Working together, paragraph 2) (McKenna et al. 2022). ‘Sometimes [we are subject to] residents' lack of cooperation, restlessness, shouting’ (*#14, Chief Executive Officer, CEO*) (p. 3 Concerns related to residents, paragraph 1) (Cohen‐Mansfield and Meschiany 2022) ‘Empathy of the staff appeared to coincide with openness to using NPIs (*Unit manager*) (p. 6 Facilitators and Barriers for NPI Implementation in LTC, paragraph 2)’

(Janzen et al. 2013)

##### 5.3.2.7 COM‐B Category: Motivation – Reflective Motivation

###### 5.3.2.7.1 TDF Domain 7: Intentions

Seven findings were categorised in the *intentions* domain that informed two categories, which include staff motivation and resident motivation (Appendix 6). These two categories informed synthesised finding 20 (Table 4).

*Synthesised Finding 20 mapped to the intention domain.*

*Synthesised Finding 20: The facility manager and staff buy‐in are critical in the implementation success of NPIs.*

When the manager is excited about an intervention, the care staff is supportive and the residents are also on board, the NPI is more likely to be implemented smoothly (Ducak et al. 2018; Forget et al. 2021; Griffiths et al. 2019; Kong et al. 2022).

‘The manager would come in and you know be really enthusiastic.’ (*DCM Expert*) (p. 7, Griffiths et al. 2019). ‘a lot of times it's easier to say, “Oh, that's her job, not mine.” But if I come from the outside, I'm hoping that the nursing staff will do it more…. And the nursing staff that I've talked to have been very supportive about it. They really liked it’ (*Regional manager/educator*). (p. 13 limiting factors, paragraph 7)

(Ducak et al. 2018)

##### 5.3.2.8 COM‐B Category: Motivation – Reflective Motivation

###### 5.3.2.8.1 TDF Domain 8: Belief About Consequences

Twenty‐two findings were categorised in the belief *about consequences* domain that informed two categories (Appendix 6). These include the efficacy of the intervention and the negative consequences of the intervention. These two categories informed synthesised findings 21 and 22 (Table 4).

*Synthesised Findings 21 and 22 mapped to the belief about consequences domain.*

*Synthesised Finding 21: The care staff's belief in the benefit of NPIs and families seeing their effectiveness could enhance their implementation.*

When both care staff and families see the impact of NPIs on the quality of life of aged‐care residents with dementia, their implementation could be enhanced (Backhouse et al. 2016; Ducak et al. 2018; Ervin et al. 2014; Forget et al. 2021; Kwak et al. 2021; McKenna et al. 2022; Pieper et al. 2018; Tasseron‐Dries et al. 2021; Van Der Ploeg et al. 2012).

‘We targeted people with dementia or motor disorders’; ‘I remember a gentleman who did not leave his room and he agreed to go for walks with the dog.’ (*PSY08, ANIM09, ANIM10*) (P3 Affected audience, paragraph 1). ‘Residents agree to walk in “her” presence’; ‘they stroke “her,” brush…’; ‘they reach out to the animal to touch “him,” pet “him” as he goes by’ (*PSY02, ANIM03, ANIM10*) (Forget et al. 2021). ‘I thought it was great that my mother connected with that doll. Because for the first time, I saw some expression on her face again. Her eyes lit up again.’ (family caregiver, daughter). ‘A kind of “seeing is believing.” And that makes it really really good.’ (*Activity coordinator*) (p. 4 ‘Activities’: Preferences of family caregivers for activities with their relative living with dementia, paragraph 4) (Tasseron‐Dries et al. 2021). Seeing the positive effects of M&M on residents and residents' characteristics (e.g., being calm, enjoyment, residents wanting to listen to music) was another facilitator (*n* = 43); (p. 9 Facilitators of Providing M&M., paragraph 1)

(Kwak et al. 2021)

*Synthesised Finding 22: The care staff's concern about the consequences of NPI use could impair their implementation.*

The adverse effects (e.g., Namaste care) and the concern about the health risk (e.g., animal hygiene) from the NPIs may impair their implementation, Additionally, the fear of resident's behavioural outbursts during the attempt to use NPIs may also deter their future implementation (Forget et al. 2021; Garrido et al. 2021; Kaasalainen et al. 2019; Kong et al. 2022).

‘When we initially started putting residents in there and they went for the four hours a day. Those residents who had maybe previous ulcers, re opened. (Site 1, director of care, page 2) Well it doesn't hurt them, the residents. Except there are some that can't go twice a day because they have skin break down, I mean they just can't handle it body wise. (Site 2, PSW/CA, Personal support worker/care aider)’ (p. 9 Barriers to implementing ‘Namaste Care’, paragraph 4) (Kaasalainen et al. 2019). ‘There could be a concern about hygiene, dog hair, if “he” licks things’; ‘families could say that it was not very hygienic’; ‘the downside is in terms of cleaning’; ‘our management isn't very animal‐friendly in our structure, it's a matter of hygiene.’ (*ASG01, PSY02, ANIM03, PSY04*) (P3 hygiene, paragraph 1) (Forget et al. 2021). In my case, I treat residents as my family, but some residents with dementia become violent. They often bite, hit, and spit at me. (*Participant 18, care worker*) (p. 6 Staff's hurtful experiences, paragraph 1)

(Kong et al. 2022)

##### 5.3.2.9 COM‐B Category: Motivation – Reflective Motivation

###### 5.3.2.9.1 TDF Domain 9: Social/Professional Roles and Identity

Eleven findings were categorised in the *social/professional roles and identity* domain that informed three categories (Appendix 6). These include the professional role, leadership role and commitment. These three categories informed synthesised finding 23 (Table 4).

*Synthesised Finding 23 mapped to the social/professional roles and identity domain.*

*Synthesised Finding 23: The lack of intervention ownership amongst the staff and role mismatching may impair the implementation of NPIs.*

When nursing staff is not taking ownership of behaviour management, jobs are not assigned in line with the scope of practice for caregivers and volunteers, it could deter the uptake of NPIs. Additionally, if caregivers don't consider NPI as their job and families leave their loved ones to the care staff, the implementation of NPIs may be slowed (Ervin et al. 2014; Griffiths et al. 2019; Hussin et al. 2021; Lawrence et al. 2016; Nunez et al. 2018; Tasseron‐Dries et al. 2021; Van Der Ploeg et al. 2012).

‘Diversional Therapy (DT) is responsible for these therapies as nursing staff are too busy with personal care. Behaviours increase in severity on weekends when DT is absent’ ‘Diversional therapy are involved with this not nursing staff’ (p. 4, Cognitive oriented strategies in dementia care, paragraph 2) (Ervin et al. 2014). What will happen is they will talk, smile and pretend to understand and then after it will be a different thing. Some of them have the attitude, ‘It's not my job, I am just here to clean him, feed him, that's it, I don't need to do anything else, it's not my job’. (*2002*) (p. 4 table 1, (2c) Relationships within the team) (Lawrence et al. 2016). ‘They (families) don't want to bring the mother to the hospital and refuse medication even though we give the options, so it's like, “as long as we pay the monthly fee, then that's it”….’ (*Shuhada, caregiver*) ‘Complicated task, such as putting in the catheter, I don't really know how to do it.’ (*Taripnan, caregiver*) (p. 12 Barrier: Responsibility outside the job scope paragraph 1)

However, championing the interventions by someone who has a good leadership style and is well‐known and accepted by the staff may enhance the implementation of NPIs (Griffiths et al. 2019).

‘It's people that you know and peer‐led, it's, you know, it's not like somebody from outside coming and talking with them, it engages the staff.’ (*Manager*) (p. 8)

(Griffiths et al. 2019)

##### 5.3.2.10 COM‐B Category: Motivation – Reflective Motivation

###### 5.3.2.10.1 TDF Domain 10: Belief About Capability

Three findings were categorised in the *belief about capability* domain that informed one category, which is the belief about capability (Appendix 6). This category informed synthesised finding 24 (Table 4).

*Synthesised Finding 24 mapped to the belief about the capability domain.*

*Synthesised Finding 24: The lack of trust by care staff in psychologists and the loss of hope amongst families regarding their loved one's participation in activities could deter NPI implementation.*

When the care staff lacks motivation because of their mistrust in the abilities of psychologists to provide innovative ideas for managing residents' behaviour and family caregivers hesitate to participate in activities with their loved ones, NPI implementation may be slowed (McKenna et al. 2022; Tasseron‐Dries et al. 2021).

‘It is so painful every time to see your wife no longer able to do anything. Then you won't participate in this kind of program.’ (*Family caregiver, daughter*) (p. 6 ‘Personal circumstances’: Personal context of family caregivers, paragraph 3) (Tasseron‐Dries et al. 2021). I know it's a bit awful to say that, but we always say, well we've known that, that's what we've done… [*Participant 3, staff*] … she's only told us what we know. She's not told us any other ways to deal with him… [*Participant 4, staff*] (p. 7 Theme 3: Understanding, paragraph 5)

(McKenna et al. 2022)

## 6 Discussion

### 6.1 Summary of Main Results

Our study identified different factors influencing the implementation of NPIs for the management of BPSD at RACHs. We categorised these factors and mapped them to 10 out of the 14 domains of the TDF. This alignment underscores the multifaceted nature of implementation, highlighting that many of the identified factors are rooted in the theoretical constructs proposed by the TDF. However, we noted that four TDF domains did not capture any of the identified factors. This suggests that certain theoretical constructs may not be relevant to the specific context of BPSD management in RACHs. The following are some factors classified under TDF‐COM‐B categories.

#### 6.1.1 COM‐B Category: Capability

##### 6.1.1.1 TDF Category: Knowledge

###### 6.1.1.1.1 Familiarity With the Intervention

Familiarity of the care staff with the NPI enhances its implementation, while unfamiliarity acts as a barrier to its implementation. This finding aligns with the research conducted by Park et al., where they found that familiarity with the social and physical environment enhances participation in activities (Park et al. 2022). The finding is also similar to the finding that a lack of awareness or knowledge amongst providers about NPIs for chronic pain management was a barrier to its implementation (Becker et al. 2017). The time delay between training and implementation, the complexity of the intervention (Griffiths et al. 2019), being naive to the intervention (McKenna et al. 2022), staff resistance to buy‐in (Ducak et al. 2018), lack of knowledge about the efficacy of the intervention (Kolanowski et al. 2010), the unfamiliarity with the use of technology (Kwak et al. 2021) were perceived to be the reason for the unfamiliarity of the staff with intervention. Onsite instructions via an overhead projector (Tasseron‐Dries et al. 2021), repetitive reminders that intervention is not time‐consuming (Ducak et al. 2018), being a newer staff (eager to learn what is going on) (McKenna et al. 2022), prior exposure (Gulliver et al. 2021) are opportunities to be familiar with the intervention.

###### 6.1.1.1.2 Familiarity With Aged‐Care Residents With Dementia

The extent to which care staff are familiar with the likes and dislikes history of their resident influences the implementation of NPIs. Not knowing the residents' preferences (Garrido et al. 2021; Kolanowski et al. 2010; Nunez et al. 2018), lack of their history and being new staff who don't know the residents (Kolanowski et al. 2010) were a hindrance to the implementation of NPIs. This is supported by the unmet need model, which suggests that the caregivers either fail to meet the needs of individuals or address them in ways that do not align with their preferences, habits and disabilities (Cohen‐Mansfield et al. 2015). Knowing residents' life history, everything about them, what they worked at, what they like to eat, their family, all their likes and dislikes, respecting their space, what they want and what they don't want (Clifford and Doody 2018), gearing up to what is for them at that moment (Kolanowski et al. 2010), being consistent in a resident routine (Janzen et al. 2013), facilitates the delivery of person‐centred care.

###### 6.1.1.1.3 Education and Training

Education and training could also influence the implementation of NPIs. Lack of training amongst nurses (Ervin et al. 2014), absence of courses about behaviour management in the education format of the physician, nurses and certified nurse assistants (Kolanowski et al. 2010), lack of person‐centred dementia care in the annual continuing education and the gap between theory and practice, lack of understanding dementia amongst the families of residents (Kong et al. 2022), lack of continuing practical education and training of person‐centred dementia care for caregivers (Clifford and Doody 2018; Cohen‐Mansfield and Meschiany 2022; Hussin et al. 2021; Kong et al. 2022), turnover of the trained staff (Kwak et al. 2021) were hindering factors related to education and training. This finding is similar to the finding that a lack of education amongst nurses is one of the hurdles in the implementation of NPIs for pain management (Tohol et al. 2023). On the other hand, training on the management of changed behaviour (Clifford and Doody 2018) and training and support (Kwak et al. 2021) on the implementation of music and memory programmes helped the implementation of NPIs.

#### 6.1.2 COM‐B Category: Opportunity

##### 6.1.2.1 TDF Category: Social Influences

###### 6.1.2.1.1 Collaboration

This systematic review revealed the importance of collaboration, with an enhancing effect on the implementation of NPIs when present and the opposite effect if it is lacking in a system. Lack of collaboration amongst the care staff and between the care staff and families of residents with dementia were the obstacles to the implementation of NPIs. This is perceived to be caused by differences in attitude towards the intervention and lack of staff buy‐in for the intervention (Graham et al. 2019; Lawrence et al. 2016), conflict of interest between daytime and night shift staff (Nunez et al. 2018), lack of understanding amongst families about the level of impairment of their loved ones, some staff members are perceived to be fault‐finders rather than supporters, having different schedules and days off amongst staff (Pieper et al. 2018), feeling of inferiority (Ducak et al. 2018), lack of trust between the resident's family and the care staff (Kong et al. 2022). However, one qualitative study has revealed that collaboration and good relationships between family members of residents with dementia and the care staff are essential to delivering individualised care, which avoids expecting residents to fit in with routines (Hughes et al. 2019). This underscores the importance of shared decision‐making (SDM) (Bunn et al. 2018) in complex interventions such as NPIs, where a one‐size‐fits‐all approach is ineffective (Kales et al. 2015). SDM is a process where healthcare choices are made collaboratively by the patient, their significant others, or both, together with one or more healthcare professionals (Légaré et al. 2018).

###### 6.1.2.1.2 Communication

Communication is key to the delivery of individualised care (Kwame and Petrucka 2021). Lack of updates about any change done to the resident care plan during the night (Nunez et al. 2018), language barrier (Hussin et al. 2021), volunteer lack of matching gender from other volunteers (Tasseron‐Dries et al. 2021), misunderstanding between care staff and family, families' reluctancy to disclose all details about their loved ones to the RACH due to rejection fear (Kong et al. 2022), rough communication between volunteers and care staff (Van Der Ploeg et al. 2012) were perceived to contribute to communication barrier. The presence of a dog (Forget et al. 2021), slow talk with eye contact and body language, use of elderspeak where applicable (Hussin et al. 2021) and laughter (Van Der Ploeg et al. 2012) facilitated communication and implementation of person‐centred care. One study has shown that good communication (informal contact) between the care staff and the family of residents with dementia not only builds a personal connection but also increases the trust and satisfaction of those involved (Hoek et al. 2020).

##### 6.1.2.2 TDF Category: Environmental Context and Resources

###### 6.1.2.2.1 Understaffing

Our findings indicated that inadequate staffing is one of the challenges to implement NPIs. Staff turnover (Kong et al. 2022), the time‐demanding nature of the intervention (Forget et al. 2021) and the increased care needs of residents with dementia (Hussin et al. 2021) are perceived to be factors that lead to insufficient staffing.

###### 6.1.2.2.2 Time Constraints

Lack of time amongst the care staff is also mentioned in the included studies. Factors such as work overload (Griffiths et al. 2019; Kaasalainen et al. 2019), inadequate staff (Ervin et al. 2014; Garrido et al. 2021; Griffiths et al. 2019; Gulliver et al. 2021; Kaasalainen et al. 2019), lack of funding (Lawrence et al. 2016), too many dementia residents at a time and the time demanding nature of emotional care (Kong et al. 2022) perceived to lead to the time constraints. This corresponds to the results of the study conducted by Swinton et al., where care workers mentioned that it was frequent for their plans to become disrupted, often due to conflicting demands and insufficient time to engage fully and be present with residents living with dementia (Swinton et al. 2024).

###### 6.1.2.2.3 Funding

The lack of funding to cover various costs of NPIs was reported as a barrier to NPI implementation, while sufficient funds facilitated the NPI implementation. Costs of training (Ducak et al. 2018; Forget et al. 2021), costs of running the programme and care (Clifford and Doody 2018), costs of gardening and activities, cost of bathing chair, balcony, salary (Cohen‐Mansfield and Meschiany 2022) and hearing aids (Cohen‐Mansfield and Meschiany 2022; Kwak et al. 2021) were hindrances to implementing NPIs for managing BPSD. Equipment characteristics such as accessibility, portability, small size and ease of use and financial support and donations were facilitators of implementing music and memory programmes in nursing homes (Kwak et al. 2021).

###### 6.1.2.2.4 Organisational Support

The support provided by RACH managers is crucial for NPI implementation. Lack of support from the RACH manager (Clifford and Doody 2018; Forget et al. 2021; Griffiths et al. 2019), the hierarchical influence of the manager (Griffiths et al. 2019), close‐minded management (Forget et al. 2021), partiality in allocating activity for residents who can voice and who are not (Backhouse et al. 2016), management scapegoating (Clifford and Doody 2018) are the hindrance to NPIs implementation. On the other hand, management support (Griffiths et al. 2019; Kaasalainen et al. 2019) and administrative buy‐in (Ducak et al. 2018) were enhancers of NPI implementation. This is consistent with a systematic review finding that underscores organisational culture as the most significant factor affecting the implementation of evidence‐based practice (Li et al. 2018).

#### 6.1.3 COM‐B Category: Motivation

##### 6.1.3.1 TDF Category: Belief About the Consequences

###### 6.1.3.1.1 Beliefs in the Efficacy of the Intervention

Individuals' belief about the effectiveness of NPI influences its implementation. The lack of perceived efficacy of the intervention is a hindrance to its implementation. This might be due to a belief that what works once doesn't work again (Ervin et al. 2014), a lack of seeing a change in the behaviour despite the intervention in place (McKenna et al. 2022) and considering the intervention as a secondary measure to manage BPSD (Backhouse et al. 2016). In contrast, when the intervention is perceived as effective, its implementation will be facilitated. This can happen when the residents show interest in engaging in activities, their quality of life improves (Ducak et al. 2018; Forget et al. 2021; Kwak et al. 2021; Pieper et al. 2018) and the staff is confident about the outcome expectancy (McKenna et al. 2022).

### 6.2 Overall Completeness and Applicability of Evidence

The evidence obtained from this systematic review is comprehensive and applicable since it encompasses the voices of relevant stakeholders including RACH managers, RACH care staff (e.g., nurses, caregivers, psychologists, physiotherapists), families of residents with dementia and volunteers. The included studies answered the research question by pinpointing relevant factors influencing NPI implementation to be used by decision‐makers.

#### 6.2.1 Quality of the Evidence

The quality of evidence for the synthesised findings was based on a quality assessment checklist using JBI SUMARI, in which almost all included studies scored at least 60%. Confidence in the quality of evidence was measured using the ConQual score, which considers the type of studies (qualitative or expert opinion), dependability based on the number of ‘yes’ responses from the five questions (Table 4) and credibility based on the extent to which the finding is supported by the accompanying illustration or quotes. Most of the synthesised findings had at least a moderate ConQual score. It can also be generalised to the RACHs since it involves different participants engaging in various roles within RACHs.

#### 6.2.2 Potential Biases in the Review Process

We excluded qualitative studies conducted on people living with dementia that did not discuss the results in association with BPSD, neuropsychiatric symptoms, challenging behaviours, or other related terms, as our research focuses on BPSD. This may potentially exclude NPIs relevant to BPSD. However, it ensures a more targeted analysis of relevant data, thus strengthening the coherence and applicability of our findings within the scope of our research objectives.

#### 6.2.3 Other Limitations

We acknowledge that the review did not focus on specific implementation strategies or address the complexity of individual interventions in detail. Additionally, no detailed analysis of the effects of different strategies and factors has been conducted.

#### 6.2.4 Agreements and Disagreements With Other Studies or Reviews

There have been no previous systematic reviews on this topic. However, one systematic review conducted in an inpatient mental health ward identified several barriers to the implementation of psychosocial interventions, including insufficient staff time, limited understanding of the interventions and high staff turnover (Thompson et al. 2024). This is in line with our findings, where these factors were listed as factors influencing the implementation of NPIs in our systematic reviews.

## 7 Authors' Conclusions

This systematic review highlighted and synthesised the critical factors influencing the implementation of NPIs for managing BPSD in RACHs. Key factors include collaboration amongst staff and families, organisational support, staffing, education and staff familiarity with both the interventions and residents. Strengthening these areas could enhance care outcomes for aged‐care residents with BPSD.

### 7.1 Implications for Practice, Policy and Research

#### 7.1.1 Implication for Practice

For decision‐makers, the findings of this systematic review highlight the need for comprehensive strategies to improve NPI implementation, which may include:

- RACHs may benefit from creating an environment that promotes collaboration amongst day and night shift staff, champions and other employees to enhance the implementation of NPIs for BPSD.
- RACHs may need to develop systems for identifying and documenting effective strategies for each resident, ensuring that interventions are tailored to their individual needs and that care staff recognise their effectiveness.
- To reduce burnout and support the implementation of personalised NPIs for BPSD, care providers might benefit from having qualified staff, a positive work culture and volunteer assistance, ensuring a stable and well‐supported workforce.
- RACHs may need committed managers who are well‐trained and supported to lead the successful implementation of NPIs for BPSD, while also fostering an environment where care staff can positively challenge and contribute to leadership decisions.
- Caregivers may need on‐going training and support to stay calm, connect meaningfully with residents and respect their dignity, thereby improving the delivery of person‐centred care for BPSD management.

#### 7.1.2 Implication for Policy

- Policies could be developed that encourage a mechanism to support team collaboration between shifts and across different roles to ensure consistency in care for BPSD.
- Policymakers could mandate the implementation of resident‐specific documentation systems in RACHs to ensure individual needs and preferences are recorded and met for BPSD.
- There may be a need for policies focused on minimum staffing levels, staff recruitment, retention and well‐being, including offering incentives for qualified staff and providing resources for a supportive working environment in the management of BPSD.
- Policy frameworks could be implemented to ensure that managers in RACHs receive the necessary leadership training and support to effectively lead NPI programmes for BPSD, with mechanisms for accountability and staff feedback.

#### 7.1.3 Implications for Research

For researchers, the findings from this systematic review provide valuable insights, including:

- Future research could investigate the impact of fostering a collaborative work culture on the success of NPI implementation for BPSD, particularly how different team dynamics (e.g., day vs night shifts) affect outcomes.
- Further research may be needed to evaluate how tailored intervention documentation impacts the quality of care and BPSD management outcomes over time.
- Research could be conducted to assess the long‐term impact of on‐going training for care teams on the success of NPI implementation and overall care delivery for BPSD.
- Future research could examine the role of leadership in RACHs, particularly how manager training and staff‐manager collaboration contribute to the effective uptake of NPIs for BPSD.

## Author Contributions

The review team includes Hunduma Dinsa Ayeno (M.Sc.), Dr. Gizat M. Kassie (Ph.D.), Dr. Mustafa Atee (Ph.D.), Assoc. Prof. Tuan Anh Nguyen (Ph.D., Associate professor). All the members of the review team possess expertise on the subject matter, systematic review methods, statistical analysis and information retrieval.

**Content:** Mr. Hunduma Ayeno, Dr. Gizat Kassie, Dr. Mustafa Atee and Associate Professor Tuan Nguyen.

**Systematic review methods:** Mr. Hunduma Ayeno, Dr. Gizat Kassie, Dr. Mustafa Atee and Associate Professor Tuan Nguyen.

**Data extraction:** Mr. Hunduma Ayeno, Dr. Gizat Kassie, Dr. Mustafa Atee and Associate Professor Tuan Nguyen.

**Data synthesis:** Mr. Hunduma Ayeno, Dr. Gizat Kassie, Dr. Mustafa Atee and Associate Professor Tuan Nguyen.

**Information retrieval:** Mr. Hunduma Ayeno, Dr. Gizat Kassie, Dr. Mustafa Atee and Associate Professor Tuan Nguyen.

All authors participated in conceptualising and designing the paper. HA drafted the paper with the contribution of GK, MA and TN. All the authors were involved in reviewing the work and have given their approval for the final version to be submitted.

## Conflicts of Interest

Mustafa Atee is a Research and Practice Lead at ‘The Dementia Centre; a research, education and consultancy arm of HammondCare, an independent Christian charity that auspices Dementia Support Australia (DSA) programmes – the Dementia Behaviour Management Advisory Service (DBMAS) and the Severe Behaviour Response Teams (SBRT)’. These programmes embrace and use multimodal, person‐centred psychosocial and non‐pharmacological interventions as part of their support strategies. The other authors declare no conflicts of interest.

## Plans for Updating This Review

The systematic review has been finalised.

## Differences Between Protocol and Review

There is no difference between the protocol and the review.

## Sources of Support

### Internal Sources

- Scholarship support, Australia

No public, commercial or not‐for‐profit sectors provided any specific grant for this paper. However, Hunduma Ayeno is supported by the University of South Australia and Australian Government research training programme scholarship.

### External Sources

- New Source of support, Other

No public, commercial or not‐for‐profit sectors provided any specific grant for this paper.

## Supporting information

Supporting information.
