# Supplementary material for: Factors Influencing the Implementation of Non‐Pharmacological Interventions for Behavioural and Psychological Symptoms of Dementia in Residential Aged‐Care Homes: A Systematic Review and Qualitative Evidence Synthesis: A systematic review
Source: Campbell Syst Rev. 2025 Mar 25;21(2):e70029. doi: 10.1002/cl2.70029 (PMC11933851; doi:10.1002/cl2.70029)
Supplement: Supplementary file 1 — Supporting information. [file CL2-21-e70029-s001.docx]

# Appendices

# Appendix 1. Search strategy

**Ovid MEDLINE(R) ALL <1946 to March 10, 2023>**

| **S. N** | **Search terms combined with Boolean logic using truncation** | **Results from 14 Mar 2023 search** |
| --- | --- | --- |
| 1 | exp residential facilities/ or housing for the elderly/ or exp long term care/ | 80,925 |
| 2 | (aged care or Assisted living or care facilit* or care home* or continu* care retirement communit* or convalescenc* home* or convalescenc* hospital* or geriatric* home* or group home* or half way house* or Halfway House* or health service* for the aged or home* for the aged or home* for the elder* or Housing for Older Person* or housing for the elder* or life care cent* or long term care or LTCF or nursing care cent* or nursing home* or old age* home* or old people* home* or Residential Care Institution* or residential facilit* or residential home* or residential institution or respite care or retirement cent* or Retirement Communit* or retirement home* or Sanatorium* or senior residence facilit* or Skilled nursing facilit* or Community living cent* or community cent* or adult family home* or memory care setting).ti,ab,kf. | 100,363 |
| 3 | dementia/ or alzheimer disease/ | 167,398 |
| 4 | (Alzheimer* or amentia* or dement* or pseudodementia).ti,ab,kf. | 272,978 |
| 5 | exp Sensory Art Therapies/ or spiritual therapies/ or Massage/ or exp Psychotherapy/ or sensory deprivation/ or Exercise Therapy/ or Occupational therapy/ or Recreation Therapy/ or Speech Therapy/ or Voice Training/ or Mind-body therapies/ | 340,796 |
| 6 | ("Acceptance and Commitment Therap*" or acoustic stimul* or activit* engagement or Acupressure or Acupuncture or anger management or animal assisted therap* or animal facilitated therap* or applied behavio* analys* or aroma therap* or Aromatherap* or art gallery or art treatment* or Art* therap* or auditory stimul* or Autogenic Training* or behavio* therap* or behavio* treatment* or Bibliotherap* or Cogniti* Remediation or cogniti* rehabilitation or Cogniti* Stimulation* or cogniti* therap* or cognitive enhancement* or Cognitive Intervention* or dance movement or Dance Therap* or Distraction or drama therap* or Environmental modification* or Environmental Therap* or ergotherap* or Exercise Intervention* or exercise therap* or Exposure Therap* or eye movement desensitization or family intervention* or Family Therap* or garden* therap* or Gardening or group therap* or group treatment* or Guided Imagery or Hearing aid* or hearing rehabilitation* or horticultur* therap* or hypnos* or life story book* or light therap* or magic table* or Massage or Meditation* or Memory training* or Milieu Therap* or mindfulness or Montessori activit* or multicomponent intervention* or multisensory environment* or multisensory therap* or Music or nonpharmacolog* or non-pharmacolog* or occupation* therap* or Outdoor activit* or person cent* or Pet Therap* or physical activit* or Physical exercise* or physiotherapy or Prayer or Psychotherap* or reality therap* or recreation* therap* or relaxation therap* or Reminiscence* or sensory environment* or sensory stimul* or Simulated presence or snoezelen or social interaction* or Spaced retrieval or Speech Therap* or spiritual or therapeutic touch* or Tovertafel or validation therap* or Video Respite or Virtual Reality or visual feedback* or Yoga or namaste care).ti,ab,kf. | 561,700 |
| 7 | Qualitative research/ or interview/ or ethnology/ or focus groups/ or personal narratives/ or Interviews as Topic/ or grounded theory/ | 191,633 |
| 8 | (audiointerview* or content analys* or discourse analys* or ethnogeograph* or ethnograph* or ethnolog* or Focus Group* or grounded approach* or Grounded Theor* or Hermeneutic* or Interview* or Narrative Analys* or Naturalistic Inquir* or oral history as topic or Patient Reported or personal narrative* or Phenomenolog* or provider reported or Qualitative or Self Report or Structured Categor* or Thematic Analys* or theoretical sampling* or Unstructured Categor* or Videointerview).ti,ab,kf. | 813,674 |
| 9 | 1 or 2 | 137,417 |
| 10 | 3 or 4 | 290,856 |
| 11 | 5 or 6 | 767,000 |
| 12 | 7 or 8 | 853,716 |
| 13 | 9 and 10 and 11 and 12 | 620 |
| 14 | limit 13 to English language | 605 |

**Ovid Emcare <1995 to 2023 Week 09>**

| **S. N** | **Search terms combined with Boolean logic using truncation** | **Results from 14 Mar 2023 search** |
| --- | --- | --- |
| 1 | Residential home/ or Nursing home/ or halfway house/ or assisted living facility/ or home for the aged/ | 27,535 |
| 2 | (aged care or Assisted living or care facilit* or care home* or continu* care retirement communit* or convalescenc* home* or convalescenc* hospital* or geriatric* home* or group home* or half way house* or Halfway House* or health service* for the aged or home* for the aged or home* for the elder* or Housing for Older Person* or housing for the elder* or life care cent* or long term care or LTCF or nursing care cent* or nursing home* or old age* home* or old people* home* or Residential Care Institution* or residential facilit* or residential home* or residential institution or respite care or retirement cent* or Retirement Communit* or retirement home* or Sanatorium* or senior residence facilit* or Skilled nursing facilit* or Community living cent* or community cent* or adult family home* or memory care setting).ti,ab,kf. | 64,775 |
| 3 | dementia/ or Alzheimer disease/ | 72,769 |
| 4 | (Alzheimer* or amentia* or dement* or pseudodementia).ti,ab,kf. | 111,340 |
| 5 | exp Psychotherapy/ or Massage/ or sensory deprivation/ or sensory stimulation/ or auditory rehabilitation/ or kinesiotherapy/ or Occupational therapy/ or Recreation Therapy/ or Speech Therapy/ or Voice Training/ or phototherapy/ or animal assisted therapy/ or Virtual Reality Exposure Therapy/ or cognitive remediation therapy/ or meditation/ or aromatherapy/ or spiritual healing/ | 131,241 |
| 6 | ("Acceptance and Commitment Therap*" or acoustic stimul* or activit* engagement or Acupressure or Acupuncture or anger management or animal assisted therap* or animal facilitated therap* or applied behavio* analys* or aroma therap* or Aromatherap* or art gallery or art treatment* or Art* therap* or auditory stimul* or Autogenic Training* or behavio* therap* or behavio* treatment* or Bibliotherap* or Cogniti* Remediation or cogniti* rehabilitation or Cogniti* Stimulation* or cogniti* therap* or cognitive enhancement* or Cognitive Intervention* or dance movement or Dance Therap* or Distraction or drama therap* or Environmental modification* or Environmental Therap* or ergotherap* or Exercise Intervention* or exercise therap* or Exposure Therap* or eye movement desensitization or family intervention* or Family Therap* or garden* therap* or Gardening or group therap* or group treatment* or Guided Imagery or Hearing aid* or hearing rehabilitation* or horticultur* therap* or hypnos* or life story book* or light therap* or magic table* or Massage or Meditation* or Memory training* or Milieu Therap* or mindfulness or Montessori activit* or multicomponent intervention* or multisensory environment* or multisensory therap* or Music or nonpharmacolog* or non-pharmacolog* or occupation* therap* or Outdoor activit* or person cent* or Pet Therap* or physical activit* or Physical exercise* or physiotherapy or Prayer or Psychotherap* or reality therap* or recreation* therap* or relaxation therap* or Reminiscence* or sensory environment* or sensory stimul* or Simulated presence or snoezelen or social interaction* or Spaced retrieval or Speech Therap* or spiritual or therapeutic touch* or Tovertafel or validation therap* or Video Respite or Virtual Reality or visual feedback* or Yoga or namaste care).ti,ab,kf. | 332,785 |
| 7 | Qualitative research/ or exp interview/ or ethnology/ or grounded theory/ or phenomenology/ or exp discourse analysis/ or exp thematic analysis/ or exp content analysis/ or ethnography/ | 281,462 |
| 8 | (audiointerview* or content analys* or discourse analys* or ethnogeograph* or ethnograph* or ethnolog* or Focus Group* or grounded approach* or Grounded Theor* or Hermeneutic* or Interview* or Narrative Analys* or Naturalistic Inquir* or oral history as topic or Patient Reported or personal narrative* or Phenomenolog* or provider reported or Qualitative or Self Report or Structured Categor* or Thematic Analys* or theoretical sampling* or Unstructured Categor* or Videointerview).ti,ab,kf. | 511,754 |
| 9 | 1 or 2 | 71,317 |
| 10 | 3 or 4 | 123,200 |
| 11 | 5 or 6 | 386,267 |
| 12 | 7 or 8 | 540,375 |
| 13 | 9 and 10 and 11 and 12 | 551 |
| 14 | limit 13 to english language | 539 |

**Embase Classic+Embase <1947 to 2023 March 10>**

| **S. N** | **Search terms combined with Boolean logic using truncation** | **Results from 14 Mar 2023 search** |
| --- | --- | --- |
| 1 | Residential home/ or Nursing home/ or halfway house/ or assisted living facility/ or home for the aged/ | 81,166 |
| 2 | (aged care or Assisted living or care facilit* or care home* or continu* care retirement communit* or convalescenc* home* or convalescenc* hospital* or geriatric* home* or group home* or half way house* or Halfway House* or health service* for the aged or home* for the aged or home* for the elder* or Housing for Older Person* or housing for the elder* or life care cent* or long term care or LTCF or nursing care cent* or nursing home* or old age* home* or old people* home* or Residential Care Institution* or residential facilit* or residential home* or residential institution or respite care or retirement cent* or Retirement Communit* or retirement home* or Sanatorium* or senior residence facilit* or Skilled nursing facilit* or Community living cent* or community cent* or adult family home* or memory care setting).ti,ab,kf. | 136,139 |
| 3 | dementia/ or Alzheimer disease/ | 354,791 |
| 4 | (Alzheimer* or amentia* or dement* or pseudodementia).ti,ab,kf. | 392,734 |
| 5 | exp Psychotherapy/ or Massage/ or sensory deprivation/ or sensory stimulation/ or auditory rehabilitation/ or kinesiotherapy/ or Occupational therapy/ or Recreation Therapy/ or Speech Therapy/ or Voice Training/ or phototherapy/ or animal assisted therapy/ or Virtual Reality Exposure Therapy/ or cognitive remediation therapy/ or meditation/ or aromatherapy/ or spiritual healing/ | 465,445 |
| 6 | ("Acceptance and Commitment Therap*" or acoustic stimul* or activit* engagement or Acupressure or Acupuncture or anger management or animal assisted therap* or animal facilitated therap* or applied behavio* analys* or aroma therap* or Aromatherap* or art gallery or art treatment* or Art* therap* or auditory stimul* or Autogenic Training* or behavio* therap* or behavio* treatment* or Bibliotherap* or Cogniti* Remediation or cogniti* rehabilitation or Cogniti* Stimulation* or cogniti* therap* or cognitive enhancement* or Cognitive Intervention* or dance movement or Dance Therap* or Distraction or drama therap* or Environmental modification* or Environmental Therap* or ergotherap* or Exercise Intervention* or exercise therap* or Exposure Therap* or eye movement desensitization or family intervention* or Family Therap* or garden* therap* or Gardening or group therap* or group treatment* or Guided Imagery or Hearing aid* or hearing rehabilitation* or horticultur* therap* or hypnos* or life story book* or light therap* or magic table* or Massage or Meditation* or Memory training* or Milieu Therap* or mindfulness or Montessori activit* or multicomponent intervention* or multisensory environment* or multisensory therap* or Music or nonpharmacolog* or non-pharmacolog* or occupation* therap* or Outdoor activit* or person cent* or Pet Therap* or physical activit* or Physical exercise* or physiotherapy or Prayer or Psychotherap* or reality therap* or recreation* therap* or relaxation therap* or Reminiscence* or sensory environment* or sensory stimul* or Simulated presence or snoezelen or social interaction* or Spaced retrieval or Speech Therap* or spiritual or therapeutic touch* or Tovertafel or validation therap* or Video Respite or Virtual Reality or visual feedback* or Yoga or namaste care).ti,ab,kf. | 783,498 |
| 7 | Qualitative research/ or exp interview/ or ethnology/ or grounded theory/ or phenomenology/ or exp discourse analysis/ or exp thematic analysis/ or exp content analysis/ or ethnography/ | 546,814 |
| 8 | (audiointerview* or content analys* or discourse analys* or ethnogeograph* or ethnograph* or ethnolog* or Focus Group* or grounded approach* or Grounded Theor* or Hermeneutic* or Interview* or Narrative Analys* or Naturalistic Inquir* or oral history as topic or Patient Reported or personal narrative* or Phenomenolog* or provider reported or Qualitative or Self Report or Structured Categor* or Thematic Analys* or theoretical sampling* or Unstructured Categor* or Videointerview).ti,ab,kf. | 1,072,058 |
| 9 | 1 or 2 | 165,117 |
| 10 | 3 or 4 | 452,384 |
| 11 | 5 or 6 | 1,025,901 |
| 12 | 7 or 8 | 1,224,693 |
| 13 | 9 and 10 and 11 and 12 | 820 |
| 14 | limit 13 to english language | 809 |

**APA PsycInfo <1806 to February Week 4 2023>**

| **S. N** | **Search terms combined with Boolean logic using truncation** | **Results from 14 Mar 2023 search** |
| --- | --- | --- |
| 1 | Residential Care Institutions/ or Nursing homes/ or Halfway Houses/ or assisted living/ or Sanatoriums/ or Retirement Communities/ or long term care/ or Group Homes/ | 27,567 |
| 2 | (aged care or Assisted living or care facilit* or care home* or continu* care retirement communit* or convalescenc* home* or convalescenc* hospital* or geriatric* home* or group home* or half way house* or Halfway House* or health service* for the aged or home* for the aged or home* for the elder* or Housing for Older Person* or housing for the elder* or life care cent* or long term care or LTCF or nursing care cent* or nursing home* or old age* home* or old people* home* or Residential Care Institution* or residential facilit* or residential home* or residential institution or respite care or retirement cent* or Retirement Communit* or retirement home* or Sanatorium* or senior residence facilit* or Skilled nursing facilit* or Community living cent* or community cent* or adult family home* or memory care setting).ti,ab. | 34,864 |
| 3 | Dementia/ or Alzheimer's Disease/ | 84,364 |
| 4 | (Alzheimer* or amentia* or dement* or pseudodementia).ti,ab. | 115,383 |
| 5 | exp psychotherapy/ or Auditory Stimulation/ or Massage/ or exp alternative medicine/ or exp Feedback/ or exp Behavior modification/ or Bibliotherapy/ or Crisis Intervention/ or Horticulture Therapy/ or Milieu Therapy/ or mindfulness/ or Mindfulness-Based Interventions/ or Catharsis/ or Cognitive Behavior Therapy/ or Mind Body Therapy/ or meditation/ or Cognitive Remediation/ or Sensory Deprivation/ or Hearing Aids/ or Occupational therapy/ or Speech Therapy/ | 374,834 |
| 6 | ("Acceptance and Commitment Therap*" or acoustic stimul* or activit* engagement or Acupressure or Acupuncture or anger management or animal assisted therap* or animal facilitated therap* or applied behavio* analys* or aroma therap* or Aromatherap* or art gallery or art treatment* or Art* therap* or auditory stimul* or Autogenic Training* or behavio* therap* or behavio* treatment* or Bibliotherap* or Cogniti* Remediation or cogniti* rehabilitation or Cogniti* Stimulation* or cogniti* therap* or cognitive enhancement* or Cognitive Intervention* or dance movement or Dance Therap* or Distraction or drama therap* or Environmental modification* or Environmental Therap* or ergotherap* or Exercise Intervention* or exercise therap* or Exposure Therap* or eye movement desensitization or family intervention* or Family Therap* or garden* therap* or Gardening or group therap* or group treatment* or Guided Imagery or Hearing aid* or hearing rehabilitation* or horticultur* therap* or hypnos* or life story book* or light therap* or magic table* or Massage or Meditation* or Memory training* or Milieu Therap* or mindfulness or Montessori activit* or multicomponent intervention* or multisensory environment* or multisensory therap* or Music or nonpharmacolog* or non-pharmacolog* or occupation* therap* or Outdoor activit* or person cent* or Pet Therap* or physical activit* or Physical exercise* or physiotherapy or Prayer or Psychotherap* or reality therap* or recreation* therap* or relaxation therap* or Reminiscence* or sensory environment* or sensory stimul* or Simulated presence or snoezelen or social interaction* or Spaced retrieval or Speech Therap* or spiritual or therapeutic touch* or Tovertafel or validation therap* or Video Respite or Virtual Reality or visual feedback* or Yoga or namaste care).ti,ab. | 440,839 |
| 7 | exp Qualitative research/ or exp Interviews/ or ethnology/ or exp phenomenology/ or ethnography/ or Hermeneutics/ | 67,028 |
| 8 | (audiointerview* or content analys* or discourse analys* or ethnogeograph* or ethnograph* or ethnolog* or Focus Group* or grounded approach* or Grounded Theor* or Hermeneutic* or Interview* or Narrative Analys* or Naturalistic Inquir* or oral history as topic or Patient Reported or personal narrative* or Phenomenolog* or provider reported or Qualitative or Self Report or Structured Categor* or Thematic Analys* or theoretical sampling* or Unstructured Categor* or Videointerview).ti,ab. | 632,576 |
| 9 | 1 or 2 | 45,807 |
| 10 | 3 or 4 | 117,619 |
| 11 | 5 or 6 | 637,718 |
| 12 | 7 or 8 | 638,929 |
| 13 | 9 and 10 and 11 and 12 | 387 |
| 14 | limit 13 to english language | 352 |

**CINAHL**

|  | Tuesday, March 14, 2023 4:32:07 AM |  |  |  |
| --- | --- | --- | --- | --- |
| **S. N** | **Search terms combined with Boolean logic using truncation** | **Limiters/Expanders** | **Last Run Via** | **Results (14 March 2023)** |
| S14 | S9 AND S10 AND S11 AND S12 | Expanders - Apply related words; Apply equivalent subjects Narrow by Language: - english Search modes - Boolean/Phrase | Interface - EBSCOhost Research Databases Search Screen - Advanced Search Database - CINAHL Complete | 1,283(1279 exported) |
| S13 | S9 AND S10 AND S11 AND S12 | Expanders - Apply related words; Apply equivalent subjects Search modes - Boolean/Phrase | Interface - EBSCOhost Research Databases Search Screen - Advanced Search Database - CINAHL Complete | 1,317 |
| S12 | S7 OR S8 | Expanders - Apply related words; Apply equivalent subjects Search modes - Boolean/Phrase | Interface - EBSCOhost Research Databases Search Screen - Advanced Search Database - CINAHL Complete | 620,865 |
| S11 | S5 OR S6 | Expanders - Apply related words; Apply equivalent subjects Search modes - Boolean/Phrase | Interface - EBSCOhost Research Databases Search Screen - Advanced Search Database - CINAHL Complete | 649,828 |
| S10 | S3 OR S4 | Expanders - Apply related words; Apply equivalent subjects Search modes - Boolean/Phrase | Interface - EBSCOhost Research Databases Search Screen - Advanced Search Database - CINAHL Complete | 109,725 |
| S9 | S1 OR S2 | Expanders - Apply related words; Apply equivalent subjects Search modes - Boolean/Phrase | Interface - EBSCOhost Research Databases Search Screen - Advanced Search Database - CINAHL Complete | 175,189 |
| S8 | TI ( audiointerview* OR content analys* OR discourse analys* OR ethnogeograph* OR ethnograph* OR ethnolog* OR Focus Group* OR grounded approach* OR Grounded Theor* OR Hermeneutic* OR Interview* OR Narrative Analys* OR Naturalistic Inquir* OR oral history as topic OR Patient Reported OR personal narrative* OR Phenomenolog* OR provider reported OR Qualitative OR Self Report OR Structured Categor* OR Thematic Analys* OR theoretical sampling* OR Unstructured Categor* OR Videointerview ) OR AB ( audiointerview* OR content analys* OR discourse analys* OR ethnogeograph* OR ethnograph* OR ethnolog* OR Focus Group* OR grounded approach* OR Grounded Theor* OR Hermeneutic* OR Interview* OR Narrative Analys* OR Naturalistic Inquir* OR oral history as topic OR Patient Reported OR personal narrative* OR Phenomenolog* OR provider reported OR Qualitative OR Self Report OR Structured Categor* OR Thematic Analys* OR theoretical sampling* OR Unstructured Categor* OR Videointerview ) | Expanders - Apply related words; Apply equivalent subjects Search modes - Boolean/Phrase | Interface - EBSCOhost Research Databases Search Screen - Advanced Search Database - CINAHL Complete | 501,799 |
| S7 | (MH "Qualitative Studies+") OR (MH "Interviews+") OR (MH "Ethnology")OR (MH "Ethnological Research") OR (MH "Focus Groups") OR (MH "Phenomenology") OR (MH "Phenomenological Research") | Expanders - Apply related words; Apply equivalent subjects Search modes - Boolean/Phrase | Interface - EBSCOhost Research Databases Search Screen - Advanced Search Database - CINAHL Complete | 343,356 |
| S6 | TI ( "Acceptance and Commitment Therap*" OR acoustic stimul* OR activit* engagement OR Acupressure OR Acupuncture OR anger management OR animal assisted therap* OR animal facilitated therap* OR applied behavio* analys* OR aroma therap* OR Aromatherap* OR art gallery OR art treatment* OR Art* therap* OR auditory stimul* OR Autogenic Training* OR behavio* therap* OR behavio* treatment* OR Bibliotherap* OR Cogniti* Remediation OR cogniti* rehabilitation OR Cogniti* Stimulation* OR cogniti* therap* OR cognitive enhancement* OR Cognitive Intervention* OR dance movement OR Dance Therap* OR Distraction OR drama therap* OR Environmental modification* OR Environmental Therap* OR ergotherap* OR Exercise Intervention* OR exercise therap* OR Exposure Therap* OR eye movement desensitization OR family intervention* OR Family Therap* OR garden* therap* OR Gardening OR group therap* OR group treatment* OR Guided Imagery OR Hearing aid* OR hearing rehabilitation* OR horticultur* therap* OR hypnos* OR life story book* OR light therap* OR magic table* OR Massage OR Meditation* OR Memory training* OR Milieu Therap* OR mindfulness OR Montessori activit* OR multicomponent intervention* OR multisensory environment* OR multisensory therap* OR Music OR nonpharmacolog* OR non-pharmacolog* OR occupation* therap* OR Outdoor activit* OR person cent* OR Pet Therap* OR physical activit* OR Physical exercise* OR physiotherapy OR Prayer OR Psychotherap* OR reality therap* OR recreation* therap* OR relaxation therap* OR Reminiscence* OR sensory environment* OR sensory stimul* OR Simulated presence OR snoezelen OR social interaction* OR Spaced retrieval OR Speech Therap* OR spiritual OR therapeutic touch* OR Tovertafel OR validation therap* OR Video Respite OR Virtual Reality OR visual feedback* OR Yoga OR namaste care ) OR AB ( "Acceptance and Commitment Therap*" OR acoustic stimul* OR activit* engagement OR Acupressure OR Acupuncture OR anger management OR animal assisted therap* OR animal facilitated therap* OR applied behavio* analys* OR aroma therap* OR Aromatherap* OR art gallery OR art treatment* OR Art* therap* OR auditory stimul* OR Autogenic Training* OR behavio* therap* OR behavio* treatment* OR Bibliotherap* OR Cogniti* Remediation OR cogniti* rehabilitation OR Cogniti* Stimulation* OR cogniti* therap* OR cognitive enhancement* OR Cognitive Intervention* OR dance movement OR Dance Therap* OR Distraction OR drama therap* OR Environmental modification* OR Environmental Therap* OR ergotherap* OR Exercise Intervention* OR exercise therap* OR Exposure Therap* OR eye movement desensitization OR family intervention* OR Family Therap* OR garden* therap* OR Gardening OR group therap* OR group treatment* OR Guided Imagery OR Hearing aid* OR hearing rehabilitation* OR horticultur* therap* OR hypnos* OR life story book* OR light therap* OR magic table* OR Massage OR Meditation* OR Memory training* OR Milieu Therap* OR mindfulness OR Montessori activit* OR multicomponent intervention* OR multisensory environment* OR multisensory therap* OR Music OR nonpharmacolog* OR non-pharmacolog* OR occupation* therap* OR Outdoor activit* OR person cent* OR Pet Therap* OR physical activit* OR Physical exercise* OR physiotherapy OR Prayer OR Psychotherap* OR reality therap* OR recreation* therap* OR relaxation therap* OR Reminiscence* OR sensory environment* OR sensory stimul* OR Simulated presence OR snoezelen OR social interaction* OR Spaced retrieval OR Speech Therap* OR spiritual OR therapeutic touch* OR Tovertafel OR validation therap* OR Video Respite OR Virtual Reality OR visual feedback* OR Yoga OR namaste care ) | Expanders - Apply related words; Apply equivalent subjects Search modes - Boolean/Phrase | Interface - EBSCOhost Research Databases Search Screen - Advanced Search Database - CINAHL Complete | 445,906 |
| S5 | TI ( (MH "Psychotherapy+") OR (MH "Massage") OR (MH "Sensory Stimulation+") OR (MH "Aromatherapy") OR (MH "Color Therapy") OR (MH "Mind Body Techniques") OR (MH "Sensory Deprivation") OR (MH "Therapeutic Exercise") OR (MH "Occupational Therapy") OR (MH "Recreational Therapy") OR (MH "Speech Therapy") ) OR AB ( (MH "Psychotherapy+") OR (MH "Massage") OR (MH "Sensory Stimulation+") OR (MH "Aromatherapy") OR (MH "Color Therapy") OR (MH "Mind Body Techniques") OR (MH "Sensory Deprivation") OR (MH "Therapeutic Exercise") OR (MH "Occupational Therapy") OR (MH "Recreational Therapy") OR (MH "Speech Therapy") ) | Expanders - Apply related words; Apply equivalent subjects Search modes - Boolean/Phrase | Interface - EBSCOhost Research Databases Search Screen - Advanced Search Database - CINAHL Complete | 308,897 |
| S4 | TI ( Alzheimer* OR amentia* OR dement* OR Pseudodement* ) OR AB ( Alzheimer* OR amentia* OR dement* OR Pseudodement* ) | Expanders - Apply related words; Apply equivalent subjects Search modes - Boolean/Phrase | Interface - EBSCOhost Research Databases Search Screen - Advanced Search Database - CINAHL Complete | 93,657 |
| S3 | (MH "Dementia") OR (MH "Alzheimer's Disease") | Expanders - Apply related words; Apply equivalent subjects Search modes - Boolean/Phrase | Interface - EBSCOhost Research Databases Search Screen - Advanced Search Database - CINAHL Complete | 78,809 |
| S2 | TI ( aged care OR Assisted living OR care facilit* OR care home* OR continu* care retirement communit* OR convalescenc* home* OR convalescenc* hospital* OR geriatric* home* OR group home* OR half way house* OR Halfway House* OR health service* for the aged OR home* for the aged OR home* for the elder* OR Housing for Older Person* OR housing for the elder* OR life care cent* OR long term care OR LTCF OR nursing care cent* OR nursing home* OR old age* home* OR old people* home* OR Residential Care Institution* OR residential facilit* OR residential home* OR residential institution OR respite care OR retirement cent* OR Retirement Communit* OR retirement home* OR Sanatorium* OR senior residence facilit* OR Skilled nursing facilit* OR Community living cent* OR community cent* OR adult family home* OR memory care setting ) OR AB ( aged care OR Assisted living OR care facilit* OR care home* OR continu* care retirement communit* OR convalescenc* home* OR convalescenc* hospital* OR geriatric* home* OR group home* OR half way house* OR Halfway House* OR health service* for the aged OR home* for the aged OR home* for the elder* OR Housing for Older Person* OR housing for the elder* OR life care cent* OR long term care OR LTCF OR nursing care cent* OR nursing home* OR old age* home* OR old people* home* OR Residential Care Institution* OR residential facilit* OR residential home* OR residential institution OR respite care OR retirement cent* OR Retirement Communit* OR retirement home* OR Sanatorium* OR senior residence facilit* OR Skilled nursing facilit* OR Community living cent* OR community cent* OR adult family home* OR memory care setting ) | Expanders - Apply related words; Apply equivalent subjects Search modes - Boolean/Phrase | Interface - EBSCOhost Research Databases Search Screen - Advanced Search Database - CINAHL Complete | 148,831 |
| S1 | (MH "Residential Facilities+") OR (MH "Housing for Older Persons") OR (MH "Assisted Living") OR (MH "Long Term Care") | Expanders - Apply related words; Apply equivalent subjects Search modes - Boolean/Phrase | Interface - EBSCOhost Research Databases Search Screen - Advanced Search Database - CINAHL Complete | 61,417 |

**Search updates**

**Database:**
Ovid MEDLINE(R) ALL <1946 to January 04, 2024>

| **S.N** | **Query** | **Results from 5 Jan 2024** |
| --- | --- | --- |
| 1 | exp residential facilities/ or housing for the elderly/ or exp long term care/ | 82,248 |
| 2 | (aged care or Assisted living or care facilit* or care home* or continu* care retirement communit* or convalescenc* home* or convalescenc* hospital* or geriatric* home* or group home* or half way house* or Halfway House* or health service* for the aged or home* for the aged or home* for the elder* or Housing for Older Person* or housing for the elder* or life care cent* or long term care or LTCF or nursing care cent* or nursing home* or old age* home* or old people* home* or Residential Care Institution* or residential facilit* or residential home* or residential institution or respite care or retirement cent* or Retirement Communit* or retirement home* or Sanatorium* or senior residence facilit* or Skilled nursing facilit* or Community living cent* or community cent* or adult family home* or memory care setting).ti,ab,kf. | 104,915 |
| 3 | dementia/ or alzheimer disease/ | 175,864 |
| 4 | (Alzheimer* or amentia* or dement* or pseudodementia).ti,ab,kf. | 290,282 |
| 5 | exp Sensory Art Therapies/ or spiritual therapies/ or Massage/ or exp Psychotherapy/ or sensory deprivation/ or Exercise Therapy/ or Occupational therapy/ or Recreation Therapy/ or Speech Therapy/ or Voice Training/ or Mind-body therapies/ | 348,314 |
| 6 | ("Acceptance and Commitment Therap*" or acoustic stimul* or activit* engagement or Acupressure or Acupuncture or anger management or animal assisted therap* or animal facilitated therap* or applied behavio* analys* or aroma therap* or Aromatherap* or art gallery or art treatment* or Art* therap* or auditory stimul* or Autogenic Training* or behavio* therap* or behavio* treatment* or Bibliotherap* or Cogniti* Remediation or cogniti* rehabilitation or Cogniti* Stimulation* or cogniti* therap* or cognitive enhancement* or Cognitive Intervention* or dance movement or Dance Therap* or Distraction or drama therap* or Environmental modification* or Environmental Therap* or ergotherap* or Exercise Intervention* or exercise therap* or Exposure Therap* or eye movement desensitization or family intervention* or Family Therap* or garden* therap* or Gardening or group therap* or group treatment* or Guided Imagery or Hearing aid* or hearing rehabilitation* or horticultur* therap* or hypnos* or life story book* or light therap* or magic table* or Massage or Meditation* or Memory training* or Milieu Therap* or mindfulness or Montessori activit* or multicomponent intervention* or multisensory environment* or multisensory therap* or Music or nonpharmacolog* or non-pharmacolog* or occupation* therap* or Outdoor activit* or person cent* or Pet Therap* or physical activit* or Physical exercise* or physiotherapy or Prayer or Psychotherap* or reality therap* or recreation* therap* or relaxation therap* or Reminiscence* or sensory environment* or sensory stimul* or Simulated presence or snoezelen or social interaction* or Spaced retrieval or Speech Therap* or spiritual or therapeutic touch* or Tovertafel or validation therap* or Video Respite or Virtual Reality or visual feedback* or Yoga or namaste care).ti,ab,kf. | 597,309 |
| 7 | Qualitative research/ or interview/ or ethnology/ or focus groups/ or personal narratives/ or Interviews as Topic/ or grounded theory/ | 197,790 |
| 8 | (audiointerview* or content analys* or discourse analys* or ethnogeograph* or ethnograph* or ethnolog* or Focus Group* or grounded approach* or Grounded Theor* or Hermeneutic* or Interview* or Narrative Analys* or Naturalistic Inquir* or oral history as topic or Patient Reported or personal narrative* or Phenomenolog* or provider reported or Qualitative or Self Report or Structured Categor* or Thematic Analys* or theoretical sampling* or Unstructured Categor* or Videointerview).ti,ab,kf. | 869,815 |
| 9 | 1 or 2 | 142,046 |
| 10 | 3 or 4 | 308,331 |
| 11 | 5 or 6 | 805,320 |
| 12 | 7 or 8 | 910,202 |
| 13 | 9 and 10 and 11 and 12 | 672 |
| 14 | limit 13 to English language | 656 |
| 15 | limit 14 to dt=20230310-20240101 | 48 |

**Database:**
Embase Classic+Embase <1947 to 2024 February 27>

| **#** | **Query** | **Results from 5 Jan 2024** |
| --- | --- | --- |
| 1 | Residential home/ or Nursing home/ or halfway house/ or assisted living facility/ or home for the aged/ | 83,798 |
| 2 | (aged care or Assisted living or care facilit* or care home* or continu* care retirement communit* or convalescenc* home* or convalescenc* hospital* or geriatric* home* or group home* or half way house* or Halfway House* or health service* for the aged or home* for the aged or home* for the elder* or Housing for Older Person* or housing for the elder* or life care cent* or long term care or LTCF or nursing care cent* or nursing home* or old age* home* or old people* home* or Residential Care Institution* or residential facilit* or residential home* or residential institution or respite care or retirement cent* or Retirement Communit* or retirement home* or Sanatorium* or senior residence facilit* or Skilled nursing facilit* or Community living cent* or community cent* or adult family home* or memory care setting).ti,ab,kf. | 141,268 |
| 3 | dementia/ or Alzheimer disease/ | 372,046 |
| 4 | (Alzheimer* or amentia* or dement* or pseudodementia).ti,ab,kf. | 408,941 |
| 5 | exp Psychotherapy/ or Massage/ or sensory deprivation/ or sensory stimulation/ or auditory rehabilitation/ or kinesiotherapy/ or Occupational therapy/ or Recreation Therapy/ or Speech Therapy/ or Voice Training/ or phototherapy/ or animal assisted therapy/ or Virtual Reality Exposure Therapy/ or cognitive remediation therapy/ or meditation/ or aromatherapy/ or spiritual healing/ | 487,498 |
| 6 | ("Acceptance and Commitment Therap*" or acoustic stimul* or activit* engagement or Acupressure or Acupuncture or anger management or animal assisted therap* or animal facilitated therap* or applied behavio* analys* or aroma therap* or Aromatherap* or art gallery or art treatment* or Art* therap* or auditory stimul* or Autogenic Training* or behavio* therap* or behavio* treatment* or Bibliotherap* or Cogniti* Remediation or cogniti* rehabilitation or Cogniti* Stimulation* or cogniti* therap* or cognitive enhancement* or Cognitive Intervention* or dance movement or Dance Therap* or Distraction or drama therap* or Environmental modification* or Environmental Therap* or ergotherap* or Exercise Intervention* or exercise therap* or Exposure Therap* or eye movement desensitization or family intervention* or Family Therap* or garden* therap* or Gardening or group therap* or group treatment* or Guided Imagery or Hearing aid* or hearing rehabilitation* or horticultur* therap* or hypnos* or life story book* or light therap* or magic table* or Massage or Meditation* or Memory training* or Milieu Therap* or mindfulness or Montessori activit* or multicomponent intervention* or multisensory environment* or multisensory therap* or Music or nonpharmacolog* or non-pharmacolog* or occupation* therap* or Outdoor activit* or person cent* or Pet Therap* or physical activit* or Physical exercise* or physiotherapy or Prayer or Psychotherap* or reality therap* or recreation* therap* or relaxation therap* or Reminiscence* or sensory environment* or sensory stimul* or Simulated presence or snoezelen or social interaction* or Spaced retrieval or Speech Therap* or spiritual or therapeutic touch* or Tovertafel or validation therap* or Video Respite or Virtual Reality or visual feedback* or Yoga or namaste care).ti,ab,kf. | 826,577 |
| 7 | Qualitative research/ or exp interview/ or ethnology/ or grounded theory/ or phenomenology/ or exp discourse analysis/ or exp thematic analysis/ or exp content analysis/ or ethnography/ | 577,018 |
| 8 | (audiointerview* or content analys* or discourse analys* or ethnogeograph* or ethnograph* or ethnolog* or Focus Group* or grounded approach* or Grounded Theor* or Hermeneutic* or Interview* or Narrative Analys* or Naturalistic Inquir* or oral history as topic or Patient Reported or personal narrative* or Phenomenolog* or provider reported or Qualitative or Self Report or Structured Categor* or Thematic Analys* or theoretical sampling* or Unstructured Categor* or Videointerview).ti,ab,kf. | 1,138,432 |
| 9 | 1 or 2 | 170,974 |
| 10 | 3 or 4 | 473,360 |
| 11 | 5 or 6 | 1,080,112 |
| 12 | 7 or 8 | 1,298,622 |
| 13 | 9 and 10 and 11 and 12 | 878 |
| 14 | limit 13 to english language | 866 |
| 15 | limit 14 to dc=20230310-20240101 | 70 |

**Database:**
Ovid Emcare <1995 to 2023 Week 52>

| **S. N** | **Query** | **Results from 5 Jan 2024** |
| --- | --- | --- |
| 1 | Residential home/ or Nursing home/ or halfway house/ or assisted living facility/ or home for the aged/ | 28,299 |
| 2 | (aged care or Assisted living or care facilit* or care home* or continu* care retirement communit* or convalescenc* home* or convalescenc* hospital* or geriatric* home* or group home* or half way house* or Halfway House* or health service* for the aged or home* for the aged or home* for the elder* or Housing for Older Person* or housing for the elder* or life care cent* or long term care or LTCF or nursing care cent* or nursing home* or old age* home* or old people* home* or Residential Care Institution* or residential facilit* or residential home* or residential institution or respite care or retirement cent* or Retirement Communit* or retirement home* or Sanatorium* or senior residence facilit* or Skilled nursing facilit* or Community living cent* or community cent* or adult family home* or memory care setting).ti,ab,kf. | 68,535 |
| 3 | dementia/ or Alzheimer disease/ | 74,393 |
| 4 | (Alzheimer* or amentia* or dement* or pseudodementia).ti,ab,kf. | 119,317 |
| 5 | exp Psychotherapy/ or Massage/ or sensory deprivation/ or sensory stimulation/ or auditory rehabilitation/ or kinesiotherapy/ or Occupational therapy/ or Recreation Therapy/ or Speech Therapy/ or Voice Training/ or phototherapy/ or animal assisted therapy/ or Virtual Reality Exposure Therapy/ or cognitive remediation therapy/ or meditation/ or aromatherapy/ or spiritual healing/ | 135,206 |
| 6 | ("Acceptance and Commitment Therap*" or acoustic stimul* or activit* engagement or Acupressure or Acupuncture or anger management or animal assisted therap* or animal facilitated therap* or applied behavio* analys* or aroma therap* or Aromatherap* or art gallery or art treatment* or Art* therap* or auditory stimul* or Autogenic Training* or behavio* therap* or behavio* treatment* or Bibliotherap* or Cogniti* Remediation or cogniti* rehabilitation or Cogniti* Stimulation* or cogniti* therap* or cognitive enhancement* or Cognitive Intervention* or dance movement or Dance Therap* or Distraction or drama therap* or Environmental modification* or Environmental Therap* or ergotherap* or Exercise Intervention* or exercise therap* or Exposure Therap* or eye movement desensitization or family intervention* or Family Therap* or garden* therap* or Gardening or group therap* or group treatment* or Guided Imagery or Hearing aid* or hearing rehabilitation* or horticultur* therap* or hypnos* or life story book* or light therap* or magic table* or Massage or Meditation* or Memory training* or Milieu Therap* or mindfulness or Montessori activit* or multicomponent intervention* or multisensory environment* or multisensory therap* or Music or nonpharmacolog* or non-pharmacolog* or occupation* therap* or Outdoor activit* or person cent* or Pet Therap* or physical activit* or Physical exercise* or physiotherapy or Prayer or Psychotherap* or reality therap* or recreation* therap* or relaxation therap* or Reminiscence* or sensory environment* or sensory stimul* or Simulated presence or snoezelen or social interaction* or Spaced retrieval or Speech Therap* or spiritual or therapeutic touch* or Tovertafel or validation therap* or Video Respite or Virtual Reality or visual feedback* or Yoga or namaste care).ti,ab,kf. | 356,537 |
| 7 | Qualitative research/ or exp interview/ or ethnology/ or grounded theory/ or phenomenology/ or exp discourse analysis/ or exp thematic analysis/ or exp content analysis/ or ethnography/ | 296,575 |
| 8 | (audiointerview* or content analys* or discourse analys* or ethnogeograph* or ethnograph* or ethnolog* or Focus Group* or grounded approach* or Grounded Theor* or Hermeneutic* or Interview* or Narrative Analys* or Naturalistic Inquir* or oral history as topic or Patient Reported or personal narrative* or Phenomenolog* or provider reported or Qualitative or Self Report or Structured Categor* or Thematic Analys* or theoretical sampling* or Unstructured Categor* or Videointerview).ti,ab,kf. | 551,209 |
| 9 | 1 or 2 | 75,083 |
| 10 | 3 or 4 | 131,124 |
| 11 | 5 or 6 | 410,611 |
| 12 | 7 or 8 | 580,258 |
| 13 | 9 and 10 and 11 and 12 | 592 |
| 14 | limit 13 to English language | 576 |
| 15 | limit 14 to dc=20230310-20240101 | 36 |

**Database:**
APA PsycInfo <1806 to January Week 1 2024>

| **S. N** | **Query** | **Results from 5 Jan 2024** |
| --- | --- | --- |
| 1 | Residential Care Institutions/ or Nursing homes/ or Halfway Houses/ or assisted living/ or Sanatoriums/ or Retirement Communities/ or long term care/ or Group Homes/ | 28,579 |
| 2 | (aged care or Assisted living or care facilit* or care home* or continu* care retirement communit* or convalescenc* home* or convalescenc* hospital* or geriatric* home* or group home* or half way house* or Halfway House* or health service* for the aged or home* for the aged or home* for the elder* or Housing for Older Person* or housing for the elder* or life care cent* or long term care or LTCF or nursing care cent* or nursing home* or old age* home* or old people* home* or Residential Care Institution* or residential facilit* or residential home* or residential institution or respite care or retirement cent* or Retirement Communit* or retirement home* or Sanatorium* or senior residence facilit* or Skilled nursing facilit* or Community living cent* or community cent* or adult family home* or memory care setting).ti,ab. | 36,112 |
| 3 | Dementia/ or Alzheimer's Disease/ | 88,368 |
| 4 | (Alzheimer* or amentia* or dement* or pseudodementia).ti,ab. | 119,914 |
| 5 | exp psychotherapy/ or Auditory Stimulation/ or Massage/ or exp alternative medicine/ or exp Feedback/ or exp Behavior modification/ or Bibliotherapy/ or Crisis Intervention/ or Horticulture Therapy/ or Milieu Therapy/ or mindfulness/ or Mindfulness-Based Interventions/ or Catharsis/ or Cognitive Behavior Therapy/ or Mind Body Therapy/ or meditation/ or Cognitive Remediation/ or Sensory Deprivation/ or Hearing Aids/ or Occupational therapy/ or Speech Therapy/ | 371,260 |
| 6 | ("Acceptance and Commitment Therap*" or acoustic stimul* or activit* engagement or Acupressure or Acupuncture or anger management or animal assisted therap* or animal facilitated therap* or applied behavio* analys* or aroma therap* or Aromatherap* or art gallery or art treatment* or Art* therap* or auditory stimul* or Autogenic Training* or behavio* therap* or behavio* treatment* or Bibliotherap* or Cogniti* Remediation or cogniti* rehabilitation or Cogniti* Stimulation* or cogniti* therap* or cognitive enhancement* or Cognitive Intervention* or dance movement or Dance Therap* or Distraction or drama therap* or Environmental modification* or Environmental Therap* or ergotherap* or Exercise Intervention* or exercise therap* or Exposure Therap* or eye movement desensitization or family intervention* or Family Therap* or garden* therap* or Gardening or group therap* or group treatment* or Guided Imagery or Hearing aid* or hearing rehabilitation* or horticultur* therap* or hypnos* or life story book* or light therap* or magic table* or Massage or Meditation* or Memory training* or Milieu Therap* or mindfulness or Montessori activit* or multicomponent intervention* or multisensory environment* or multisensory therap* or Music or nonpharmacolog* or non-pharmacolog* or occupation* therap* or Outdoor activit* or person cent* or Pet Therap* or physical activit* or Physical exercise* or physiotherapy or Prayer or Psychotherap* or reality therap* or recreation* therap* or relaxation therap* or Reminiscence* or sensory environment* or sensory stimul* or Simulated presence or snoezelen or social interaction* or Spaced retrieval or Speech Therap* or spiritual or therapeutic touch* or Tovertafel or validation therap* or Video Respite or Virtual Reality or visual feedback* or Yoga or namaste care).ti,ab. | 458,858 |
| 7 | exp Qualitative research/ or exp Interviews/ or ethnology/ or exp phenomenology/ or ethnography/ or Hermeneutics/ | 70,560 |
| 8 | (audiointerview* or content analys* or discourse analys* or ethnogeograph* or ethnograph* or ethnolog* or Focus Group* or grounded approach* or Grounded Theor* or Hermeneutic* or Interview* or Narrative Analys* or Naturalistic Inquir* or oral history as topic or Patient Reported or personal narrative* or Phenomenolog* or provider reported or Qualitative or Self Report or Structured Categor* or Thematic Analys* or theoretical sampling* or Unstructured Categor* or Videointerview).ti,ab. | 666,336 |
| 9 | 1 or 2 | 47,437 |
| 10 | 3 or 4 | 122,281 |
| 11 | 5 or 6 | 655,172 |
| 12 | 7 or 8 | 672,956 |
| 13 | 9 and 10 and 11 and 12 | 415 |
| 14 | limit 13 to English language | 385 |
| 15 | limit 14 to up=20230310-20240101 | 26 |

CINAHL updated search results.

|  | Thursday, February 29, 2024, 12:30:40 AM |  |  |  |
| --- | --- | --- | --- | --- |
| **#** | **Query** | **Limiters/Expanders** | **Last Run Via** | **Results** |
| S15 | S9 AND S10 AND S11 AND S12 AND EM 20230310-20240101 | Expanders - Apply related words; Apply equivalent subjects Narrow by Language: - **English** Search modes - Boolean/Phrase | Interface - EBSCOhost Research Databases Search Screen - Advanced Search Database - CINAHL Complete | 79 |
| S14 | S9 AND S10 AND S11 AND S12 AND EM 20230310-20240101 | Expanders - Apply related words; Apply equivalent subjects Search modes - Boolean/Phrase | Interface - EBSCOhost Research Databases Search Screen - Advanced Search Database - CINAHL Complete | 83 |
| S13 | S9 AND S10 AND S11 AND S12 | Expanders - Apply related words; Apply equivalent subjects Search modes - Boolean/Phrase | Interface - EBSCOhost Research Databases Search Screen - Advanced Search Database - CINAHL Complete | 1,392 |
| S12 | S7 OR S8 | Expanders - Apply related words; Apply equivalent subjects Search modes - Boolean/Phrase | Interface - EBSCOhost Research Databases Search Screen - Advanced Search Database - CINAHL Complete | 629,928 |
| S11 | S5 OR S6 | Expanders - Apply related words; Apply equivalent subjects Search modes - Boolean/Phrase | Interface - EBSCOhost Research Databases Search Screen - Advanced Search Database - CINAHL Complete | 650,056 |
| S10 | S3 OR S4 | Expanders - Apply related words; Apply equivalent subjects Search modes - Boolean/Phrase | Interface - EBSCOhost Research Databases Search Screen - Advanced Search Database - CINAHL Complete | 110,811 |
| S9 | S1 OR S2 | Expanders - Apply related words; Apply equivalent subjects Search modes - Boolean/Phrase | Interface - EBSCOhost Research Databases Search Screen - Advanced Search Database - CINAHL Complete | 172,751 |
| S8 | TI ( audiointerview* OR content analys* OR discourse analys* OR ethnogeograph* OR ethnograph* OR ethnolog* OR Focus Group* OR grounded approach* OR Grounded Theor* OR Hermeneutic* OR Interview* OR Narrative Analys* OR Naturalistic Inquir* OR oral history as topic OR Patient Reported OR personal narrative* OR Phenomenolog* OR provider reported OR Qualitative OR Self Report OR Structured Categor* OR Thematic Analys* OR theoretical sampling* OR Unstructured Categor* OR Videointerview ) OR AB ( audiointerview* OR content analys* OR discourse analys* OR ethnogeograph* OR ethnograph* OR ethnolog* OR Focus Group* OR grounded approach* OR Grounded Theor* OR Hermeneutic* OR Interview* OR Narrative Analys* OR Naturalistic Inquir* OR oral history as topic OR Patient Reported OR personal narrative* OR Phenomenolog* OR provider reported OR Qualitative OR Self Report OR Structured Categor* OR Thematic Analys* OR theoretical sampling* OR Unstructured Categor* OR Videointerview ) | Expanders - Apply related words; Apply equivalent subjects Search modes - Boolean/Phrase | Interface - EBSCOhost Research Databases Search Screen - Advanced Search Database - CINAHL Complete | 508,516 |
| S7 | (MH "Qualitative Studies+") OR (MH "Interviews+") OR (MH "Ethnology")OR (MH "Ethnological Research") OR (MH "Focus Groups") OR (MH "Phenomenology") OR (MH "Phenomenological Research") | Expanders - Apply related words; Apply equivalent subjects Search modes - Boolean/Phrase | Interface - EBSCOhost Research Databases Search Screen - Advanced Search Database - CINAHL Complete | 359,876 |
| S6 | TI ( "Acceptance and Commitment Therap*" OR acoustic stimul* OR activit* engagement OR Acupressure OR Acupuncture OR anger management OR animal assisted therap* OR animal facilitated therap* OR applied behavio* analys* OR aroma therap* OR Aromatherap* OR art gallery OR art treatment* OR Art* therap* OR auditory stimul* OR Autogenic Training* OR behavio* therap* OR behavio* treatment* OR Bibliotherap* OR Cogniti* Remediation OR cogniti* rehabilitation OR Cogniti* Stimulation* OR cogniti* therap* OR cognitive enhancement* OR Cognitive Intervention* OR dance movement OR Dance Therap* OR Distraction OR drama therap* OR Environmental modiﬁcation* OR Environmental Therap* OR ergotherap* OR Exercise Intervention* OR exercise therap* OR Exposure Therap* OR eye movement desensitization OR family intervention* OR Family Therap* OR garden* therap* OR Gardening OR group therap* OR group treatment* OR Guided Imagery OR Hearing aid* OR hearing rehabilitation* OR horticultur* therap* OR hypnos* OR life story book* OR light therap* OR magic table* OR Massage OR Meditation* OR Memory training* OR Milieu Therap* OR mindfulness OR Montessori activit* OR multicomponent intervention* OR multisensory environment* OR multisensory therap* OR Music OR nonpharmacolog* OR non-pharmacolog* OR occupation* therap* OR Outdoor activit* OR person cent* OR Pet Therap* OR physical activit* OR Physical exercise* OR physiotherapy OR Prayer OR Psychotherap* OR reality therap* OR recreation* therap* OR relaxation therap* OR Reminiscence* OR sensory environment* OR sensory stimul* OR Simulated presence OR snoezelen OR social interaction* OR Spaced retrieval OR Speech Therap* OR spiritual OR therapeutic touch* OR Tovertafel OR validation therap* OR Video Respite OR Virtual Reality OR visual feedback* OR Yoga OR namaste care ) OR AB ( "Acceptance and Commitment Therap*" OR acoustic stimul* OR activit* engagement OR Acupressure OR Acupuncture OR anger management OR animal assisted therap* OR animal facilitated therap* OR applied behavio* analys* OR aroma therap* OR Aromatherap* OR art gallery OR art treatment* OR Art* therap* OR auditory stimul* OR Autogenic Training* OR behavio* therap* OR behavio* treatment* OR Bibliotherap* OR Cogniti* Remediation OR cogniti* rehabilitation OR Cogniti* Stimulation* OR cogniti* therap* OR cognitive enhancement* OR Cognitive Intervention* OR dance movement OR Dance Therap* OR Distraction OR drama therap* OR Environmental modiﬁcation* OR Environmental Therap* OR ergotherap* OR Exercise Intervention* OR exercise therap* OR Exposure Therap* OR eye movement desensitization OR family intervention* OR Family Therap* OR garden* therap* OR Gardening OR group therap* OR group treatment* OR Guided Imagery OR Hearing aid* OR hearing rehabilitation* OR horticultur* therap* OR hypnos* OR life story book* OR light therap* OR magic table* OR Massage OR Meditation* OR Memory training* OR Milieu Therap* OR mindfulness OR Montessori activit* OR multicomponent intervention* OR multisensory environment* OR multisensory therap* OR Music OR nonpharmacolog* OR non-pharmacolog* OR occupation* therap* OR Outdoor activit* OR person cent* OR Pet Therap* OR physical activit* OR Physical exercise* OR physiotherapy OR Prayer OR Psychotherap* OR reality therap* OR recreation* therap* OR relaxation therap* OR Reminiscence* OR sensory environment* OR sensory stimul* OR Simulated presence OR snoezelen OR social interaction* OR Spaced retrieval OR Speech Therap* OR spiritual OR therapeutic touch* OR Tovertafel OR validation therap* OR Video Respite OR Virtual Reality OR visual feedback* OR Yoga OR namaste care ) | Expanders - Apply related words; Apply equivalent subjects Search modes - Boolean/Phrase | Interface - EBSCOhost Research Databases Search Screen - Advanced Search Database - CINAHL Complete | 438,440 |
| S5 | TI ( (MH "Psychotherapy+") OR (MH "Massage") OR (MH "Sensory Stimulation+") OR (MH "Aromatherapy") OR (MH "Color Therapy") OR (MH "Mind Body Techniques") OR (MH "Sensory Deprivation") OR (MH "Therapeutic Exercise") OR (MH "Occupational Therapy") OR (MH "Recreational Therapy") OR (MH "Speech Therapy") ) OR AB ( (MH "Psychotherapy+") OR (MH "Massage") OR (MH "Sensory Stimulation+") OR (MH "Aromatherapy") OR (MH "Color Therapy") OR (MH "Mind Body Techniques") OR (MH "Sensory Deprivation") OR (MH "Therapeutic Exercise") OR (MH "Occupational Therapy") OR (MH "Recreational Therapy") OR (MH "Speech Therapy") ) | Expanders - Apply related words; Apply equivalent subjects Search modes - Boolean/Phrase | Interface - EBSCOhost Research Databases Search Screen - Advanced Search Database - CINAHL Complete | 313,882 |
| S4 | TI ( Alzheimer* OR amentia* OR dement* OR Pseudodement* ) OR AB ( Alzheimer* OR amentia* OR dement* OR Pseudodement* ) | Expanders - Apply related words; Apply equivalent subjects Search modes - Boolean/Phrase | Interface - EBSCOhost Research Databases Search Screen - Advanced Search Database - CINAHL Complete | 93,848 |
| S3 | (MH "Dementia") OR (MH "Alzheimer's Disease") | Expanders - Apply related words; Apply equivalent subjects Search modes - Boolean/Phrase | Interface - EBSCOhost Research Databases Search Screen - Advanced Search Database - CINAHL Complete | 79,455 |
| S2 | TI ( aged care OR Assisted living OR care facilit* OR care home* OR continu* care retirement communit* OR convalescenc* home* OR convalescenc* hospital* OR geriatric* home* OR group home* OR half way house* OR Halfway House* OR health service* for the aged OR home* for the aged OR home* for the elder* OR Housing for Older Person* OR housing for the elder* OR life care cent* OR long term care OR LTCF OR nursing care cent* OR nursing home* OR old age* home* OR old people* home* OR Residential Care Institution* OR residential facilit* OR residential home* OR residential institution OR respite care OR retirement cent* OR Retirement Communit* OR retirement home* OR Sanatorium* OR senior residence facilit* OR Skilled nursing facilit* OR Community living cent* OR community cent* OR adult family home* OR memory care setting ) OR AB ( aged care OR Assisted living OR care facilit* OR care home* OR continu* care retirement communit* OR convalescenc* home* OR convalescenc* hospital* OR geriatric* home* OR group home* OR half way house* OR Halfway House* OR health service* for the aged OR home* for the aged OR home* for the elder* OR Housing for Older Person* OR housing for the elder* OR life care cent* OR long term care OR LTCF OR nursing care cent* OR nursing home* OR old age* home* OR old people* home* OR Residential Care Institution* OR residential facilit* OR residential home* OR residential institution OR respite care OR retirement cent* OR Retirement Communit* OR retirement home* OR Sanatorium* OR senior residence facilit* OR Skilled nursing facilit* OR Community living cent* OR community cent* OR adult family home* OR memory care setting ) | Expanders - Apply related words; Apply equivalent subjects Search modes - Boolean/Phrase | Interface - EBSCOhost Research Databases Search Screen - Advanced Search Database - CINAHL Complete | 146,041 |
| S1 | (MH "Residential Facilities+") OR (MH "Housing for Older Persons") OR (MH "Assisted Living") OR (MH "Long Term Care") | Expanders - Apply related words; Apply equivalent subjects Search modes - Boolean/Phrase | Interface - EBSCOhost Research Databases Search Screen - Advanced Search Database - CINAHL Complete | 61,736 |

# Appendix 2. Search strategies for other sources

**For AAIC abstract:** non-pharmacological interventions for behaviors and psychological symptoms of dementia (https://alz-journals.onlinelibrary.wiley.com/action/doSearch?AllField=nonpharmacological+interventions+for+behaviors+and+psychological+symptoms+of+dementia+&SeriesKey=15525279&pageSize=20&startPage=1)

**For trove and ProQuest**: Factors influencing the implementation of non-pharmacological interventions for behaviors and psychological symptoms of dementia in residential aged care homes

**Trove**: https://trove.nla.gov.au/search/advanced/category/research?keyword=Factors%20influencing%20the%20implementation%20of%20nonpharmacological%20interventions%20for%20behaviors%20and%20psychological%20symptoms%20of%20dementia%20in%20residential%20aged%20care%20homes&l-format=Thesis

**ProQuest**: https://www.proquest.com/pqdtglobal/selecteditems?accountid=14649# Research & Reports Show advanced search Simple search Keyword: Factors influencing the implementation of non-pharmacological interventions for behaviors and psychological symptoms of dementia in residential aged care homes

**RESEARCH & REPORTS** 5 total results Sort by: Relevance Year (latest first) Year (earliest first)

Ethics of antipsychotic treatment for people with behavioural and psychological symptoms of dementia in long-term care facilities. Thesis - 2021Hojjat Soofi ... facilities (LTCFs) to manage behavioural and psychological symptoms of dementia (BPSD). The current evidence ... Ethics of antipsychotic treatment for people with behavioural and psychological symptoms of ... dementia in long-term care facilities ... original and detailed investigation into the ethical justifiability of using APs for managing residents ... At MacquariUni Inst Rep

Is it ethically justifiable to use antipsychotics in long-term care facilities for managing people with behavioral and psychological symptoms of dementia? Thesis - 2017Hojjat Soofi ... Is it ethically justifiable to use antipsychotics in long-term care facilities for managing people ... with behavioral and psychological symptoms of dementia? ... people with behavioral and psychological symptoms of dementia (BPSD). This practice gives rise to various ... The use of antipsychotics (APs) is common practice in long-term care facilities (LTCFs) to manage ... At MacquariUni Inst Rep

Psychosocial interventions for pain management in older adults with dementia: A systematic review of randomized controlled trials Article, Article/Other article, Article/Journal or magazine article - 2 editions: 2019Pu, Lihui; Moyle, Wendy; Jones, Cindy +1 more ... dementia. No Full Text AIM: To assess the effectiveness of psychosocial interventions on pain in older
... of randomized controlled trials ... Psychosocial interventions for pain management in older adults with dementia: A systematic review ... potential reduction of pain and pain medication in people with dementia. Healthcare providers may wish to ...

The Development of a Personalised Multimedia System for Individuals with Dementia Thesis - 2018KANVAR SINGH NAYER ... The Development of a Personalised Multimedia System for Individuals with Dementia ... This research aimed to propose and develop a response to challenges posed by the common symptoms of ... dementia prevalent in residents at aged-care facilities, in the form of a non-medicinal intervention. The ... At Monash Online

Functional maintenance initiatives for acutely hospitalised older adults Thesis - 2020ALETHEA YI-CHEN KAVANAGH ... Functional maintenance initiatives for acutely hospitalised older adults ... evaluation, and the importance of considering
key factors influencing implementation success. ... interventions demonstrated a reduction in the likelihood of discharge to nursing-staffed facilities. However ... At Monash Online

# Appendix 3: Definition for the list of non-pharmacological interventions included in the systematic review.

| **Study** | **Type of non-pharmacologic interventions** | **Definition** |
| --- | --- | --- |
| Backhouse et al., 2016 {published data only} | Aromatherapy massage | A type of complementary and alternative medicine that uses rubbing and kneading of the skin with plant oils that give off strong, pleasant aromas (smells) to promote relaxation, a sense of well-being, and healing.( <https://www.cancer.gov/publications/dictionaries/cancer-terms/def/aromatherapy-massage>) |
|  | Arts and crafts | activities that need both artistic and practical skills, such as making cloth, jewellery and [pottery](https://www.oxfordlearnersdictionaries.com/definition/english/pottery) (<https://www.oxfordlearnersdictionaries.com/definition/english/arts-and-crafts>) |
|  | Behavioural therapy | refers to a range of treatments and techniques which are used to change an individual's maladaptive responses to specific situations (<https://www.sciencedirect.com/science/article/pii/B0080430767037311>). |
|  | Bingo | a game of chance played with cards having numbered squares corresponding to numbers drawn at random (as by a game host or computer program) and won by covering five such squares in a row.( <https://www.merriam-webster.com/dictionary/bingo>) |
|  | Catch | A catch game is a game played by two players (<https://link.springer.com/article/10.1007/s00182-018-0640-z>).  The repetition of throwing and catching a ball is calming, plus it's good for coordination. Use a ball that is soft, beanbag, or stuffed animal to prevent injuries if your loved one gets hit accidentally.( <https://www.webmd.com/alzheimers/activities-for-people-with-dementia>) |
|  | Church service | a service with care homes which aim to enable to person with dementia to:   - Approach God - Worship and praise God and feel his caring presence - To come to God with their pain and sorrows as well as joy and thanksgiving and praise - To seek God for help, to be comforted and to ask for healing - To spark a faith-filled response to God.( <https://d3hgrlq6yacptf.cloudfront.net/5f3ffd8a9f6aa/content/pages/documents/care-home-worship-services.pdf>) |
|  | Cooking | Is an activity of stirring, kneading, and dividing food items into portions which can improve dexterity and fine motor skills, which are often severely impaired in people with dementia. Plus, the activities also provide a sense of purpose to the senior. (<https://bethesdahealth.org/blog/therapeutic-benefits-of-cooking-for-seniors-with-dementia/#:~:text=Stirring%2C%20kneading%2C%20and%20dividing%20food,by%20many%20types%20of%20dementia>.) |
|  | Dancing | is a therapy which offers individuals the opportunity to being truly present, to engage in their senses, and to tap into their independence, individuality, and self-awareness. It’s always worth remembering that moving the body goes hand in hand with moving the mind! (<https://neuroclin.com/dancing-and-dementia/#:~:text=So%20far%2C%20research%20has%20shown,their%20self%2Dawareness%20and%20self%2D>) |
|  | Doll therapy | is sometimes be referred to as 'child representation’ and can provide people with dementia an opportunity to interact with a 'lifelike' baby doll in a manner that may be therapeutic to them. Dolls have been used as an intervention to reduce the impact of behaviours or unmet needs. (<https://www.dementia.com.au/resource-hub/the-use-of-dolls-in-dementia-care#:~:text=Using%20dolls%2C%20or%20what%20can,of%20behaviours%20or%20unmet%20needs>.) |
|  | Dominoes | is a game which provides a safe and fun environment providing an opportunity to experience pleasure, evokes positive memories of games played in the past and provides hours of entertainment to people who rarely (and unfortunately) may be unable to do much throughout their day.( <https://letstalkaboutdementia.wordpress.com/2022/10/06/how-i-learnt-the-meaning-of-purposeful-occupation-through-playing-a-game-of-dominoes/>) |
|  | Exercises | Activities which including, but not limited to, doing housework, taking your dog for a walk, pacing while waiting for the kettle to boil.( <https://www.alzheimers.org.uk/get-support/daily-living/exercise/types-ideas>) |
|  | Flower arranging | is an activity that allows for creative expression, provides gentle exercise, and offers a genuine connection with nature. Whether you’re a seasoned gardener or simply someone who appreciates beauty, flower arranging can enrich your life in surprising ways. Flowers offer a feast for the senses. Their vibrant colours delight the eyes, their fragrances awaken memories, and the touch of their delicate petals soothes the soul.( <https://www.welcometomonarchlanding.com/blog/floral-arranging-for-seniors/#:~:text=Cognitive%20Stimulation,focus%2C%20and%20overall%20cognitive%20function>.) |
|  | Skittles | a [game](https://dictionary.cambridge.org/dictionary/english/game) [played](https://dictionary.cambridge.org/dictionary/english/play) [especially](https://dictionary.cambridge.org/dictionary/english/especially) in [Britain](https://dictionary.cambridge.org/dictionary/english/britain) in which [players](https://dictionary.cambridge.org/dictionary/english/player) [roll](https://dictionary.cambridge.org/dictionary/english/roll) a [ball](https://dictionary.cambridge.org/dictionary/english/ball) at [objects](https://dictionary.cambridge.org/dictionary/english/object) [shaped](https://dictionary.cambridge.org/dictionary/english/shaped) like [bottles](https://dictionary.cambridge.org/dictionary/english/bottle) to [try](https://dictionary.cambridge.org/dictionary/english/try) to [knock](https://dictionary.cambridge.org/dictionary/english/knock) them down and [score](https://dictionary.cambridge.org/dictionary/english/score) [points](https://dictionary.cambridge.org/dictionary/english/focus).( <https://dictionary.cambridge.org/dictionary/english/skittle>)  It is an activity that helps with balance and hand-eye coordination. Taking part can also provide good opportunities for meeting and interacting with other people. As the movements are quite slow, this reduces the risk of injury. (<https://www.alzheimers.org.uk/get-support/daily-living/exercise/types-ideas#:~:text=Indoor%20bowls%20or%20skittles,reduces%20the%20risk%20of%20injury>.) |
|  | Gardening | is using the natural world—specifically gardens and the[act of gardening](https://www.drjohnlapuma.com/naturetherapy/how-gardening-heals/)—to help mitigate some of the medical, behavioural and emotional issues that adults with dementia deal with.( <https://www.drjohnlapuma.com/wellness-and-health/what-is-gardening-for-dementia/>)  It can bring a range of benefits for people living with dementia, including improved memory, attention, social interaction, reduced stress and increased feelings of calm and relaxation.( <https://forwardwithdementia.au/news/gardening-and-people-living-with-dementia/>) |
|  | Halloween party | is a celebration that can provide an opportunity to trigger positive memories through reminiscence therapy. It encourages individuals with dementia to share their own Halloween memories, looking at old photos or telling stories about past experiences. This can bring a sense of joy and connectedness, even if their short-term memory is compromised.( <https://hiddenharbors.com/blog/f/halloween-and-dementia-celebrating-while-supporting-memory>) |
|  | Hand massage | is a therapy that has been found to reduce cortisol levels and produce a physiological relaxation response. Gently pressing the palm and rubbing the knuckles in therapeutic motions has helped dementia patients reduce their agitated behaviour during morning care routines.( <https://acfb.edu.au/3-massages-that-can-help-dementia-patients/#:~:text=Gently%20pressing%20the%20palm%20and,behaviour%20during%20morning%20care%20routines>.) |
|  | Holiday | A holiday offers the chance to have new and stimulating experiences. These could include talking to different people, going to new places or doing different types of activities.( <https://www.alzheimers.org.uk/get-support/staying-independent/holidays-and-travelling>) |
|  | Jigsaws | A jigsaw or jigsaw [puzzle](https://www.collinsdictionary.com/dictionary/english/puzzle) is a [picture](https://www.collinsdictionary.com/dictionary/english/picture) on [cardboard](https://www.collinsdictionary.com/dictionary/english/cardboard) or [wood](https://www.collinsdictionary.com/dictionary/english/wood) that has been [cut](https://www.collinsdictionary.com/dictionary/english/cut) up into [odd](https://www.collinsdictionary.com/dictionary/english/odd) shapes. You have to make the picture again by [putting](https://www.collinsdictionary.com/dictionary/english/putt) the pieces [together](https://www.collinsdictionary.com/dictionary/english/together) correctly. (<https://www.collinsdictionary.com/dictionary/english/jigsaw>)  Jigsaws for dementia can be very useful. They’re designed to contain less pieces – typically between 12 and 35 pieces – so they’re simpler to complete, but with images that are suitable for an adult, and which may help to stimulate reminiscence and conversation.( <https://www.liftedcare.com/news/what-are-the-benefits-of-dementia-jigsaw-puzzles/>) |
|  | Jubilee celebration | A [jubilee](https://www.collinsdictionary.com/dictionary/english/jubilee) is a special anniversary of an event, [especially](https://www.collinsdictionary.com/dictionary/english/especially) the 25th or 50th anniversary.( <https://www.collinsdictionary.com/dictionary/english/jubilee-celebration>) |
|  | Multisensory bath | is ideally suited for snoozing, because it already offers a number of sensory experiences of its own, for example the warmth and security of the water and personal contact. It is a bathroom with a real feeling of well-being, both client and carer can feel comfortable in this room. (LED) Light, fragrances and sounds always give the room its own character. (<https://www.nenko.com/projects-overview/multi-sensory-bathroom/>) |
|  | Music Therapy | Music therapy is a therapeutic approach that uses the [naturally mood-lifting properties of music](https://www.verywellmind.com/surprising-psychological-benefits-of-music-4126866) to help people improve their mental health and overall well-being. (<https://www.verywellmind.com/benefits-of-music-therapy-89829>) |
|  | Music: CD | is the ’Simple Music Player’ that has been designed to be as easy as possible to operate for the end user; that is, the person with dementia. Setting up the player is undertaken by friends, family or caregivers, as a one-time process.( <https://www.alzstore.com/simple-music-player-dementia-alzheimers-p/2115.htm>) |
|  | Nail varnishing | Also known as nail polish is one of the primary forms of nail beautification. ( <https://www.mdpi.com/2079-9284/4/3/24>) |
|  | Newspapers | a [document](https://dictionary.cambridge.org/dictionary/english/document) [published](https://dictionary.cambridge.org/dictionary/english/publish) [regularly](https://dictionary.cambridge.org/dictionary/english/regularly), consisting of [news](https://dictionary.cambridge.org/dictionary/english/news) [reports](https://dictionary.cambridge.org/dictionary/english/report), [articles](https://dictionary.cambridge.org/dictionary/english/article), [photographs](https://dictionary.cambridge.org/dictionary/english/photograph), and [advertisements](https://dictionary.cambridge.org/dictionary/english/advertisement) that are [printed](https://dictionary.cambridge.org/dictionary/english/printed) on [large](https://dictionary.cambridge.org/dictionary/english/large) [sheets](https://dictionary.cambridge.org/dictionary/english/sheet) of [paper](https://dictionary.cambridge.org/dictionary/english/paper) [folded](https://dictionary.cambridge.org/dictionary/english/fold) together. (<https://dictionary.cambridge.org/dictionary/english/newspaper>) |
|  | Olympic celebration | is a nursing home Olympic games that may need some moderation to be applicable to a particular person and modified as needed. (<https://thisinsidiousdementia.com/summer-olympic-games-ideas-for-a-memory-care-unit/>) |
|  | Outside entertainment | Activities such as going outdoors and performing household tasks which can help people with dementia live well with their condition, reduce their symptoms, and even slow the progression of dementia.( <https://www.medicalnewstoday.com/articles/dementia-activities>) |
|  | Pat Dog | is a pet therapy that involves the use of dogs and other animals to help people cope with health problems and recover from diseases and disorders. For people living with dementia, this therapy involves guided interactions between the individual, a trained animal, and the animal’s handler. ( <https://www.scalabrini.com.au/the-benefits-of-pet-therapy-for-people-living-with-dementia/#:~:text=What%20is%20Pet%20Therapy%3F,animal%2C%20and%20the%20animal's%20handler>.) |
|  | Quiz/giant crossword | A games like which may help older adults prevent [brain plaques associated with Alzheimer’s disease](http://www.cbsnews.com/news/alzheimers-brain-plaques-prevented-by-lifetime-of-puzzles-study-suggests/).( <https://dailycaring.com/free-large-print-crossword-puzzles-for-seniors/>) |
|  | Read to residents | is a reading aloud to groups of people with dementia which has been found to stimulate memories and imagination.( <https://www.theguardian.com/society/2010/oct/05/reading-aloud-dementia-patients>) |
|  | Reading with residents | is a bibliotherapy which is one type of reading group that can help seniors with dementia. It’s built around the benefits of reading aloud as a group.( <https://www.memorycare.com/power-of-reading-and-benefits-of-libraries-for-people-with-dementia/>) |
|  | Reflexology | is treatment that may be beneficial in the management of distress in nursing home residents with mild to moderate stage dementia.( <https://naturalmedicineweek.com.au/reflexology-for-nursing-home-residents-with-mild-dementia/>) |
|  | Reminiscence | is a therapy that involves the discussion of memories and past experiences with other people using tangible prompts such as photographs or music to evoke memories and stimulate conversation. ( <https://www.ncbi.nlm.nih.gov/pmc/articles/PMC6494367/>) |
|  | Staff leaving party | is a farewell party which is an event or get-together that celebrate an employee’s departure from a company and show appreciation for their service. ( <https://teambuilding.com/blog/farewell-party-ideas>) |
|  | Television | A Memory Lane TV which is a simple, safe, and inexpensive way for families and caregivers to help someone with dementia lower anxiety and boost energy levels. (<https://www.memory-lane.tv/#:~:text=Backed%20by%20over%2050%20years,memories%20and%20relive%20past%20joys>.) |
|  | Trips out | is a memory trips often for seniors living with dementia who lose their short and long-term memory. It involves taking trips to memorable or fond places that can help spark memories in your loved one. (<https://inspiredliving.care/tips-for-dementia-day-trips/#:~:text=Places%20like%2C%20botanical%20gardens%2C%20museums,beneficial%20for%20slowing%20cognitive%20decline>.) |
|  | Walks outside | is an exercise that helps to work off the restless urge to wander that is typical of Alzheimer’s patients. It is useful to combine the walk with a useful errand, such as going to the shops for milk or exercising the dog.( <https://www.betterhealth.vic.gov.au/health/conditionsandtreatments/dementia-activities-and-exercise>) |
| Chaudhry et al., 2020 {published data only} | Montessori interventions | is a method that has found to be moderately effective in improving certain behavioural outcomes such as eating behaviours, agitation and mood. This intervention is based on designing and offering activities that take into consideration the interests, needs, past experiences and preferences of the group or participants. ( <https://doi.org/10.1192/bjo.2020.49>) |
| Clifford & Doody, 2018 {published data only} | Person-centred care (PCC) | is a sociopsychological treatment approach that recognizes the individuality of the patient in relation to the attitudes and care practices that surround them. The PCC approach recognizes that there are unmet needs, such as isolation, that may be the basis of behavioural symptoms or NPS in patients with dementia. The PCC approach enables health care providers to understand and provide support for the unmet needs of the individual with dementia. (<https://www.ncbi.nlm.nih.gov/pmc/articles/PMC5322939/>) |
| Cohen-Mansfield & Meschiany, 2022 {published data only} | Quality of care to improve quality of life. | is an approach that promote patient choice and autonomy (e.g., rights, not using restraints), dignity, individuality, physical and emotional comfort, access to meaningful activity and relationships. It involves maximising residents’ engagement and pleasure and minimising disruption to residents’ natural cycle of activity and inactivity, such as sleep, walking and movement. ( <https://doi.org/10.1016/j.gerinurse.2021.12.012>) |
| Ducak et al., 2018 {published data only} | Montessori Methods | is a method that can help address responsive behaviours experienced by long-term care residents living with dementia by increasing their participation in and enjoyment of daily life while decreasing fear, anger, anxiety, agitation and social withdrawal.( <https://doi.org/10.1177/1471301215625342>) |
| Ervin et al., 2014 {published data only} | Behaviour oriented strategies; Cognitive oriented strategies; Stimulation oriented strategies; Emotion oriented strategies | **Behaviour oriented strategy** is about creating individualised strategies for people with disability that are responsive to the person’s needs, in a way that reduces and eliminates the need for the use of regulated restrictive practices. Behaviour support focuses on evidence-based strategies and person-centred supports that address the needs of the person with disability and the underlying causes of behaviours of concern, while safeguarding the dignity and quality of life of people with disability who require specialist behaviour support.( <https://www.ndiscommission.gov.au/providers/understanding-behaviour-support-and-restrictive-practices-providers>)  **Cognitive oriented strategy** is an umbrella term referring to several non-pharmacological treatment approaches which apply a range of techniques to engage thinking and cognition with various degrees of breadth and specificity. For example, cognitive training involves repeated practice on a set of structured and standardized tasks, designed to target one or several cognitive abilities.( [10.1007/s11065-020-09434-8](https://doi.org/10.1007%2Fs11065-020-09434-8))  **Stimulation oriented strategies**: Cognitive stimulation (CS) is an intervention for people with dementia offering a range of enjoyable activities providing general stimulation for thinking, concentration and memory, usually in a social setting, such as a small group.( [10.1002/14651858.CD005562.pub3](https://doi.org/10.1002%2F14651858.CD005562.pub3))  **Emotion oriented strategies**: Emotion-oriented care approaches offer the opportunity to tailor the care to the individual needs of dementing elderly. (<https://modernedementiezorg.nl/upl/kwaliteit_van_zorg/Finnema%202000%20review.pdf>) |
| Forget et al., 2021 {published data only} | Animal assisted intervention | is the use of animal to create social stimulation for the elderly with normal aging, i.e. it provides opportunities for patients to engage in social interaction and initiate social behaviours. For the elderly with Alzheimer’s dementia, the literature shows that the presence of an animal such as a dog can fill the feeling of loneliness and generate positive emotions.( <https://doi.org/10.1016/j.ctim.2020.102591>)  it is meant to improve physical, social, emotional or cognitive functioning, with animals as integral part of the treatment.( [10.1016/j.eujim.2016.05.005](https://doi.org/10.1016%2Fj.eujim.2016.05.005)) |
| Garrido et al., 2021 {published data only} | Music playlist | is a personalised playlist interventions that involves the creation of music playlists based on individual music preferences without the involvement of a registered music therapist and have also been shown to be effective ways to improve symptoms in people with dementia (<https://doi.org/10.2147/JMDH.S293764>) |
| Griffiths et al., 2019 {published data only} | Dementia Care Mapping (staff-led psychosocial intervention) | Dementia Care Mapping (DCM™) is an observational tool set within a practice development process, which aims to support staff working in formal care settings to record and understand the care experience of people with dementia and to use this as a basis for person-centred care planning. It is an established practice development tool and process aimed to help care home staff deliver more person-centred care. (<https://doi.org/10.1186/s12877-019-1045-y>) |
| Gulliver et al., 2021 {published data only} | Music engagement program | The music engagement program (MEP) fosters an approach to music where participants of all ages, abilities and skill levels become co-facilitators of others’ music making. In the context of residential aged care, a typical MEP outreach-singing event involves participating in group singing sessions led by a facilitator, with or without simple accompaniment.(<https://doi.org/10.1016/j.evalprogplan.2021.101930>) |
| Janzen et al., 2013 {published data only} | Non-pharmacologic interventions such as pet therapy, calming music, reminiscence therapy and 17 other interventions. | **Calming Music**: Calming or relaxation music is characterized by qualities that are soothing and alter the stress response. (<file:///C:/Users/ayehd001/Downloads/calming_music_and_hand_massage_with_agitated.8.pdf>)  **Physical activity**: is an activity that provide benefits for people with dementia including improved cognition, activities of daily life and independence, functional ability, and mental health. It is likely that social benefits can be significant: for example, if physical activity is undertaken in a group, it can increase social networks and reduce feelings of loneliness and isolation, known to be issues for many people with dementia. ( <http://www.biomedcentral.com/1471-2318/13/129>)  **Singing**: is a working choir that welcomes those living with dementia and their care partner, who can benefit from and contribute to practices and performances.( <https://bhabrisbane.org.au/singing-dementia/>)  **Reminiscence therapy:** is a therapy that involves the discussion of memories and past experiences with other people using tangible prompts such as photographs or music to evoke memories and stimulate conversation. ( <https://www.ncbi.nlm.nih.gov/pmc/articles/PMC6494367/>)  **Humour:** is the [ability](https://dictionary.cambridge.org/dictionary/english/ability) to [find](https://dictionary.cambridge.org/dictionary/english/find) things [funny](https://dictionary.cambridge.org/dictionary/english/funny), the way in which [people](https://dictionary.cambridge.org/dictionary/english/people) [see](https://dictionary.cambridge.org/dictionary/english/see) that some things are [funny](https://dictionary.cambridge.org/dictionary/english/funny), or the [quality](https://dictionary.cambridge.org/dictionary/english/quality) of being [funny](https://dictionary.cambridge.org/dictionary/english/funny).( <https://dictionary.cambridge.org/dictionary/english/humour>)  **Pet therapy:** is a guided interaction between a person and a trained animal. It also involves the animal’s handler. (<https://www.healthline.com/health/pet-therapy>)  **The activities of daily living (ADLs)**: is a term used to collectively describe fundamental skills required to independently care for oneself, such as eating, bathing, and mobility.( <https://www.ncbi.nlm.nih.gov/books/NBK470404/#:~:text=Introduction,.%5B1%5D%5B2%5D>)  **Cognitive games:** is a cognitive brain training that challenges your brain and helps to improve cognitive function. This can include memory games, logic puzzles, and even Sudoku. ( <https://acognitiveconnection.com/what-is-cognitive-brain-training/>)  **Horticultural therapy**: is an approach to therapy in which activities related to cultivating plants are used with a view to improve an individual's social, emotional, educational, psychological, and physical wellbeing. Horticultural therapies may occur inside as well as in outdoor garden areas. (<https://www.alzscot.org/living-with-dementia/getting-support/accessing-alzheimer-scotland-support/therapeutic-activity/horticultural-therapies#:~:text=Horticultural%20activities%20at%20Alzheimer%20Scotland,%2C%20psychological%2C%20and%20physical%20wellbeing>.)  **Hand massage:** is a therapy that has been found to reduce cortisol levels and produce a physiological relaxation response. Gently pressing the palm and rubbing the knuckles in therapeutic motions has helped dementia patients reduce their agitated behaviour during morning care routines.( <https://acfb.edu.au/3-massages-that-can-help-dementia-patients/#:~:text=Gently%20pressing%20the%20palm%20and,behaviour%20during%20morning%20care%20routines>.)  [Table Game](https://www.lawinsider.com/dictionary/table-game)**:** means any house-banked game played with cards, dice, equipment or any device, including but not limited to blackjack, twenty-one, poker, craps, roulette.( <https://www.lawinsider.com/dictionary/table-game>)  **Cooking groups**: is the use of therapeutic cooking programs to motivate residents to attend, socialize, perform cognitive activities in a small group, and enjoy the shared experience.( <https://libres.uncg.edu/ir/uncg/f/L_Buettner_Therapeutic_2003.pdf>)  **Reading group** is a bibliotherapy which is one type of reading group that can help seniors with dementia. It’s built around the benefits of reading aloud as a group.( <https://www.memorycare.com/power-of-reading-and-benefits-of-libraries-for-people-with-dementia/>)  **Doll therapy:** is sometimes be referred to as 'child representation’ and can provide people with dementia an opportunity to interact with a 'lifelike' baby doll in a manner that may be therapeutic to them. Dolls have been used as an intervention to reduce the impact of behaviours or unmet needs. (<https://www.dementia.com.au/resource-hub/the-use-of-dolls-in-dementia-care#:~:text=Using%20dolls%2C%20or%20what%20can,of%20behaviours%20or%20unmet%20needs>.)  **Multi-sensory therapy** is a therapeutic approach that has been shown to have benefits for people with dementia. By engaging different senses such as touch, taste, smell, sight, and sound, multi-sensory stimulation can help individuals with dementia to connect with their environment and improve their cognitive and emotional functioning.( <https://www.physioinq.com.au/blog/transform-dementia-care-with-multi-sensory-stimulation>)  **A physical restraint:** involves the use of action or physical force to prevent, restrict or subdue movement of a person’s body, or part of their body, for the primary purpose of influencing their behaviour.( <https://dcj.nsw.gov.au/service-providers/deliver-disability-services/restrictive-practices-authorisation-portal/resources/restrictive-practices-guidance-physical-restraint.html>)  **Aromatherapy**: is defined as any therapy entailing the use of essential oils extracted from herbs, flowers and other plants to improve physical, emotional, and spiritual well-being.( <https://www.sciencedirect.com/topics/medicine-and-dentistry/aromatherapy>)  **Social restraint**: Psycho-social restraint is the use of ‘power-control’ strategies. Examples of psycho-social restraints include but are not limited to:   - Requiring a person to stay in one area of the house until told they can leave - Directing a person to stay in a unlocked room, corner of an area, or stay in a specific space until requested to leave (also known as ‘exclusionary time-out’). (<https://www.senseswa.com.au/wp-content/uploads/2016/01/02-UseOfRestrictivePracticesProcedure.docx>)   **Thermal bath** involves the use of heated, commercially available, premoistened disposable washcloths or towels. Use of the thermal bath demonstrated an overall reduced frequency of agitated and aggressive behaviours. (<https://www.healthplexus.net/files/content/2009/October/1209dementia.pdf>)  **Bright light therapy** is safe and may be beneficial in treating depression and agitation in older. (<https://doi.org/10.1192/bja.2020.5>) |
| Kaasalainen et al., 2019 {published data only} | Namaste care | ‘Namaste Care’ comes from a Hindu term meaning ‘To honour the spirit within’. It emphasises the dignity that persons with advanced-stage dementia deserve despite their level of ability. Two main principles that guide the ‘Namaste Care’ programme are a special environment and a loving touch. These principles are honoured as care providers engage with the residents and provide meaningful activities such as gentle hand or foot massages, application of a familiar scented lotion and calming music therapy. (DOI: 10.1177/1744987119832932) |
| Kolanowski et al., 2010 {published data only} | recreational activities, aromatherapy, music and relaxation, behavioural techniques (distraction and non-confrontational interaction) | **Recreational activities** are activities that people participate in for leisure. These are activities that are meant to engage persons living with dementia and are not specifically intended to meet therapeutic outcomes.( <https://www.dementiahub.sg/living-well-with-dementia/recreational-activities/#:~:text=Recreational%20activities%20are%20activities%20that,intended%20to%20meet%20therapeutic%20outcomes>.)  **Aromatherapy**: is defined as any therapy entailing the use of essential oils extracted from herbs, flowers and other plants to improve physical, emotional and spiritual well-being.( <https://www.sciencedirect.com/topics/medicine-and-dentistry/aromatherapy>)  **Music therapy** is a therapeutic approach that uses the [naturally mood-lifting properties of music](https://www.verywellmind.com/surprising-psychological-benefits-of-music-4126866) to help people improve their mental health and overall well-being. (<https://www.verywellmind.com/benefits-of-music-therapy-89829>)  **Relaxation techniques** include Yoga or meditation; exercising/walking; writing your thoughts and feelings in a journal; gardening; listening to music; or watching a favourite television program that you find relaxing or enjoyable.( <https://www.alz.org/help-support/i-have-alz/live-well/reducing-stress>)  **Distraction techniques** are useful to help redirect energy away from challenging behaviour and towards something more positive. For example, if a person is agitated try giving them a task such as making a cup of tea or drawing. Suggesting alternative activities such as going for a walk or playing games also helps divert attention away from any negative emotions. (<https://timian.co.uk/using-de-escalation-techniques-for-managing-challenging-behaviour/>)  **De-escalation techniques** are non-confrontational methods used to defuse potentially volatile situations, while maintaining respect for all parties involved. These strategies focus on communication in order for us to better understand a person’s needs and respond in the most effective way possible. (<https://timian.co.uk/using-de-escalation-techniques-for-managing-challenging-behaviour/>) |
| Kong & Kim, 2022 {published data only} | Person-centred care (PCC) | is a sociopsychological treatment approach that recognizes the individuality of the patient in relation to the attitudes and care practices that surround them. The PCC approach recognizes that there are unmet needs, such as isolation, that may be the basis of behavioural symptoms or NPS in patients with dementia. The PCC approach enables health care providers to understand and provide support for the unmet needs of the individual with dementia. (<https://www.ncbi.nlm.nih.gov/pmc/articles/PMC5322939/>) |
| Kwak et al., 2021 {published data only} | Music and Memory program | **Music and Memory program** is a popular intervention that provides individualized music listening, has shown potential to improve residents’ quality of life. (<https://doi.org/10.1177/1471301220962234>) |
| Lawrence et al., 2016 {published data only} | Psychosocial interventions | **Psychosocial interventions** were defined as any program that incorporates techniques that aim to reduce psychosocial distress, by reducing anxiety and depression and increasing social activities.( <https://www.sciencedirect.com/topics/psychology/psychosocial-intervention>) |
| McKenna et al., 2022 {published data only} | Team formulation-led care | Team formulation is defined as “a forum where time is dedicated to supporting the team to use psychologically informed frameworks that take into account social, cultural and biological factors to develop a compassionate understanding of a person's difficulties and needs from multiple perspectives, central to which is the person's history and their way of making sense of experience.” (DOI: 10.1002/jclp.23481) |
| Hussin et al., 2021 {published data only} | physical exercise, music therapy, reminiscence therapy and pet therapy | **Physical exercise** has been seen as a beneficial non-pharmacological therapy in the prevention and management of dementia, and possible benefits may not only impact on participants, but also indirectly on their caregivers.( <https://doi.org/10.1186/s12877-020-01938-5>)  **Music therapy** is a therapeutic approach that uses the [naturally mood-lifting properties of music](https://www.verywellmind.com/surprising-psychological-benefits-of-music-4126866) to help people improve their mental health and overall well-being. (<https://www.verywellmind.com/benefits-of-music-therapy-89829>)  **Reminiscence therapy:** is a therapy that involves the discussion of memories and past experiences with other people using tangible prompts such as photographs or music to evoke memories and stimulate conversation. ( <https://www.ncbi.nlm.nih.gov/pmc/articles/PMC6494367/>)  **Pet therapy:** is a guided interaction between a person and a trained animal. It also involves the animal’s handler. (<https://www.healthline.com/health/pet-therapy>) |
| Miller et al., 2021 {published data only} | Psychosocial and environmental care | **Psychosocial and environmental care** practices are recommended to address behavioral expressions in persons with dementia.( <https://doi.org/10.1016/j.gerinurse.2020.09.003>) |
| Nunez et al., 2018 {published data only} | Person-centred care (PCC) | is a sociopsychological treatment approach that recognizes the individuality of the patient in relation to the attitudes and care practices that surround them. The PCC approach recognizes that there are unmet needs, such as isolation, that may be the basis of behavioural symptoms or NPS in patients with dementia. The PCC approach enables health care providers to understand and provide support for the unmet needs of the individual with dementia. (<https://www.ncbi.nlm.nih.gov/pmc/articles/PMC5322939/>) |
| Pieper et al., 2018 {published data only} | Multidisciplinary and multicomponent intervention | **A stepwise,** **multidisciplinary and multicomponent intervention** (called STA OP!) was implemented in Dutch nursing home units, which included a comprehensive multidisciplinary team training. Training an entire multidisciplinary nursing home team facilitates interdisciplinary learning, collaboration and communication. ( DOI: <https://doi.org/10.5334/ijic.3973>) |
| Tasseron-Dries et al., 2021 {published data only} | Namaste care family program | **Namaste Care** is a program based on a palliative and person-centered care approach and aims to increase quality of life of nursing home residents with advanced dementia. Namaste Care consists of psychological, social, and spiritual components. It responds to the five most important psychological needs of people with dementia, as identified by [Kitwood (1997)](https://www.sciencedirect.com/science/article/pii/S0020748921001139#bib0017). These five needs are comfort, attachment, identity, being involved in the process of life (occupation) and feeling part of a group (inclusion).( <https://doi.org/10.1016/j.ijnurstu.2021.103968>) |
| Van Der Ploeg et al., 2012 {published data only} | Volunteers’ engagement with residents with BPSD | **Volunteer** work has been defined as unpaid work that benefits other persons to whom no obligation is owed. Volunteers were perceived by facility representatives as helpful to residents through provision of stimulation and company. Approximately half of the staff members mentioned the engagement and stimulation resulting from volunteers’ visits and bonding between residents and volunteers as positives. As an alternative resource, aged care volunteers could assist with the implementation of personalized activities (doi:10.1017/S1041610212000798) |
| Webster et al., 2022 {published data only} | Multi-component non-pharmacological interventions | **Multi-component non-pharmacological interventions** for managing nighttime sleep disturbances include food and drink, comfort and company, occupying and tiring the resident, quit and dark bedroom, assessing and managing pain and discomfort, monitoring residents. (<https://doi.org/10.1371/journal.pone.0272814>) |

# Appendix 4: Characteristics of excluded studies [ordered by study ID]

| **Study** | **Reason for exclusion** |
| --- | --- |
| Almutairi et al., 2022 | Ineligible phenomenon of interest: it is effectiveness of online training program. There is no mention of barrier or facilitator or related terms |
| Alsawy et al., 2020 | Ineligible context/setting. It is home setting. |
| Argyle & Kelly, 2015 | Ineligible context/setting. It is home setting. |
| Bauer et al., 2012 | Ineligible study design. Telephone interview quantitative survey. |
| Beach & Kramer, 1999 | Ineligible phenomenon of interest: There is no mention of barrier or facilitator or related terms. |
| Berendonk & Caine, 2017 | Ineligible phenomenon of interest: There is no mention of barrier or facilitator or related terms. |
| Boersma et al., 2017 | Ineligible population: there is no mention of Behavioural and psychological symptoms of dementia or similar terms in the article |
| Bott et al., 2022 | Ineligible phenomenon of interest. no mention of barriers or facilitator or similar terms |
| Brett et al., 2018 | Ineligible population: there is no mention of Behavioural and psychological symptoms of dementia or similar terms in the article |
| (Buist et al., 2018) | Ineligible population: there is no mention of Behavioural and psychological symptoms of dementia or similar terms in the article |
| Campo & Chaudhury, 2012 | Ineligible context. Setting: non-institutional or home like ambiance |
| Cioffi et al., 2007 | Ineligible phenomenon of interest. Facilitator/barrier---no. it seemed to be discussing the effect of environmental change on the residents with dementia |
| Collier & Jakob, 2017 | Ineligible phenomenon of interest. Facilitator/barrier: none voiced by the participants |
| Cooke, 2018 | Ineligible population. Dementia without BPSD |
| Dickinson et al., 2017 | Ineligible population. Population: Dementia without BPSD |
| Douglas et al., 2021 | Ineligible population. Population: Dementia (no BPSD) |
| Doyle, 2014 | Ineligible population. Population: Dementia (no BPSD or related terms) |
| du Toit & Buchanan, 2018 | Ineligible phenomena of interest. Barrier/facilitators: none. Mixed population. |
| Ekra & Dale, 2020 | Ineligible population. Dementia without BPSD. No barrier or facilitator. |
| Evans et al., 2018 | Ineligible phenomena of interest. no non-pharmacologic intervention |
| Evans et al., 2019 | Ineligible population. Population: Dementia without BPSD |
| Figueiredo et al., 2013 | Ineligible phenomenon of interest. Facilitator/barrier: none identified; it seemed to be discussing the effectiveness of the training program on staff knowledge. |
| Francis et al., 2020 | Ineligible phenomena of interest. Barrier/facilitator: none- it discusses the effectiveness of biographical films in knowing the resident with dementia to improve care. |
| Garrido et al., 2021 | Ineligible context. Dementia home care and aged care facilities |
| Gaviola et al., 2021 | Ineligible population. Dementia without BPSD |
| Gebhard & Mir, 2021 | Ineligible population. Dementia without BPSD |
| Griffiths et al., 2021 | Ineligible population. Dementia and other older people. no BPSD |
| Gundersen & Johannessen, 2018 | Ineligible population. All nursing home residents. |
| Hale et al., 2020 | Ineligible context. Setting: both home and RACHs |
| Harmer & Orrell, 2008 | Ineligible population. Dementia without BPSD |
| Haunch et al., 2023 | Ineligible population. Dementia without BPSD |
| (Hebert et al., 2018) | Ineligible population. Mixed population. population: all residents in the long-term care facilities |
| Hicks et al., 2022 | Ineligible population. Population: Dementia (no BPSD or related terms mentioned) |
| Hung et al., 2016 | Ineligible phenomena of interest. no barrier or facilitator |
| Hung et al., 2021 | Full text not available |
| Hunter et al., 2017 | Ineligible population. All older population in care homes |
| Ibsen & Eriksen, 2021 | Ineligible context. Setting: home |
| Kelley et al., 2020 | Ineligible population. Dementia without BPSD |
| Kim et al., 2018 | Ineligible context. Setting: home |
| Law et al., 2019 | Ineligible phenomena of interest. NPI: None Barriers/facilitator or related term: None. It discusses the overall caring experiences of healthcare assistants |
| Sook et al., 2016 | Ineligible phenomena of interest. Barriers/facilitators: none. It seems to be discussing the effect of environment on resident behaviours |
| Lee et al., 2020 | Full text not available |
| Lee et al., 2021 | Ineligible phenomena of interest. Barrier/facilitator: none. it discusses the effect of physical environment on the care |
| Levy-Storms & Chen, 2020 | Ineligible phenomena of interest. Barrier/facilitators: none. it focuses on how to communicate emotional support |
| McCarroll et al., 2020 | Ineligible phenomena of interest. NPI: none Barrier/facilitator: none. it discusses how domiciliary physiotherapists are perceived by the allied health professionals |
| McCarthy et al., 2023 | Ineligible phenomena of interest. Barrier/facilitator: none. it discusses the experiences and perceptions of proxies caring for resident with dementia. |
| McDermott et al., 2014 | Ineligible phenomena of interest. no barrier or facilitator |
| McKeown et al., 2010 | Ineligible phenomena of interest. no barrier or facilitator |
| McNiel & Westphal, 2018 | Ineligible phenomena of interest. no barrier or facilitator |
| Morgan & Stewart, 1999 | Ineligible phenomena of interest. no barrier or facilitator |
| Moyle et al., 2015 | Ineligible phenomena of interest. NPI: none Barriers/facilitators: none. it discusses the influencers of quality of life |
| Moyle et al., 2019 | Ineligible phenomena of interest. Barriers/facilitators: none. it discusses the effectiveness of lifelike baby dolls. |
| Murphy et al., 2018 | Ineligible phenomenon of interest. no barrier/facilitator with illustration |
| Noguchi et al., 2013 | Ineligible phenomenon of interest. Facilitator/barrier: none |
| O'Donnell et al., 2021 | Full text not available |
| Palan et al., 2022 | Ineligible phenomena of interest. no non-pharmacologic intervention |
| Paun & Cothran, 2019 | Ineligible phenomenon of interest. no non-pharmacological intervention |
| Powers & Watson, 2011 | Ineligible population. Dementia without BPSD |
| Ragneskog & Kihlgren, 1997 | Ineligible phenomena of interest. no barrier or facilitator |
| Rokstad et al., 2015 | Ineligible population. Dementia without BPSD |
| Rosvik et al., 2011 | Ineligible phenomenon of interest. Facilitator/barrier or similar term= none. |
| Schwartz et al., 2020 | Full text not available |
| Tak et al., 2015 | Ineligible population. Population: mixed Dementia and general elderly |
| Thoft et al., 2022 | Ineligible population. Mixed population. Dementia + other older people |
| Toivonen et al., 2022 | Ineligible context. Home care and long-term care facilities |
| Van Der Geer et al., 2009 | Ineligible phenomena of interest. no barrier or facilitator |
| van Haeften-van et al., 2015 | Ineligible population: there is no mention of Behavioural and psychological symptoms of dementia or similar terms in the article |
| van Wyk et al., 2017 | Ineligible phenomenon of interest. NPI: not mentioned. but general care Barrier/facilitator: none |
| Watson & Hatcher, 2021 | Ineligible population. population: mixed population NPI: none Facilitator/barrier: none |
| Williams et al., 2015 | Ineligible phenomena of interest. Barrier/facilitators: none. Mixed population. |
| Windle et al., 2020 | Ineligible phenomenon of interest. Barrier/facilitator: none. Dementia without BPSD |
| Zimmerman et al., 2005 | Ineligible phenomena of interest. NPI: not mentioned Barrier/facilitator: None: it discusses about the caregiver attitude, stress and satisfaction with dementia care |

# Appendix 5. List of Study Findings with Illustrations

| **Study:** Kolanowski et al., 2010 | |
| --- | --- |
| Finding | Caregivers feeling of insecurity (U) |
| Illustration | “We said we can medicate her, the greatest good for the greatest number [in relation to woman who was screaming].” |
| Finding | Lack of education (U) |
| Illustration | “I don’t think that new CNAs come out of class knowing how to deal with combative, aggressive, or apathetic residents.” One participant stated, “. . . they don’t seek to understand the behavior; they just try to address it and I think that’s when you come up on failure because you don’t really understand what’s causing that behavior.” Physicians, CNAs, and nurse participants echoed that education “is just something that’s been missing in our educational format.” (Pg 5 The Educational Needs of Staff, paragraph 1) |
| Finding | The inability of staff to meet the resident where they are at the moment (U) |
| Illustration | They [the resident] had an increased confusion and they were trying to bring them back to reality and it wasn’t working at all and everyone was thinking urinary tract infection ... . There wasn’t an increase in confusion it was just the one person trying to bring them over here instead of stepping into their world. (Pg 4 resident behaviors, paragraph 1) This one gentleman that urinated in the heater every night ... it was something to do with the bathroom ... the heater unit actually looked like the latrine or whatever he was used to when he was in the service.” (Domain of time) (Pg 4 resident behaviors, paragraph 2) |
| Finding | Staff’s lack of skills (C) |
| Illustration | majority of participants stated that interventions are “not going to fit every person” and a variety of interventions or activities are attempted or “pulled out of your bag of tricks and used.” One participant conveyed, “we try to reorient them; try to focus their attention on something else ... explore all possibilities.” (Pg 5 The Educational Needs of Staff, paragraph 2) |
| Finding | the changing landscape (U) |
| Illustration | For example, individuals with dementia constitute a large percentage of the residents staff cares for, and these residents are much more impaired today than in the past: Twelve years ago ... we had more physically able folks... .When I first started here there weren’t all these personal care homes. So now that they are, by the time they come to us it’s really difficult to get them involved in things that they can actually do. (Domain of time.) (Pg 3 The Changing Landscape, paragraph 1) |
| Finding | Aggressiveness against caregiver (U) |
| Illustration | Sometimes we have an all-black crew and this person does not like black people and is saying don’t let that “N” touch me ... we cannot possibly say well we’ll get a white CNA for you. So, you just try to assure them that, that person is there to help them. Sometimes we can switch a male CNA with a female CNA because some of the older white ladies perceive black men as being a danger to them. (Pg 4 The Changing Landscape, paragraph 2) |
| Finding | Lack of knowledge about the effectiveness of NPIs (U) |
| Illustration | One participant expressed, “Just a little touch of something (medication) is helping her get to activities, not crying, not upset all evening and night you know .... So, I don’t think no pharmacology whatsoever is the answer.” |
| Finding | Knowing residents timing and the concept of time (U) |
| Illustration | So, it’s a matter of us spending time, it’s not a matter of how big our budget is. But it’s not like we have all the time in the world ... when you have residents coming in now that are in their 90’s. Time was also referred to in reference to the resident’s altered sense of time. Yeah, cause they’re on their own time zone. I mean 3 o’clock in the morning to us is whatever it is to them. They don’t follow any particular time ...so you really have to gear up to what is for them at that moment. (Pg 5 Reaching Out to the Person with Dementia, paragraph 8) |
| Finding | rise in resident acuity level or impairments (U) |
| Illustration | They don’t keep residents in the hospital a long time. So, you know you’re dealing with a lot of medical things. That unfortunately has to be our priority and the poor resident that’s here with dementia is sort of left behind. (Pg 3 The Changing Landscape, paragraph 2) |
| Finding | Not knowing residents (U) |
| Illustration | “Sometimes families aren’t able to give us a whole lot of history and if they haven’t had a close relationship with mom or dad ... that could really be a barrier.” (Pg 4 Reaching Out to the Person with Dementia, paragraph 3) |
| Finding | lack of understanding among newly hired employees (U) |
| Illustration | For example, one participant conveyed, if it’s a CNA that’s been here for a while and they know that particular person, then they’re going to know how to deal with the behaviours... versus somebody that’s brand new coming in and doesn’t know that particular resident. (Pg 5 The Educational Needs of Staff, paragraph 4) |
| Finding | knowing residents’ past occupation and their interests (U) |
| Illustration | Yeah, I had a resident who was a farmer, and you know very withdrawn and ... not engaged in life. Had lost meaning and purpose but by engaging him in therapeutic activities of just gardening that allowed him to continue to explore and to give him a sense of identity again. (Pg 4 Reaching Out to the Person with Dementia, paragraph 2) |
| Finding | Residents' inability to recognise one's level of impairments (U) |
| Illustration | A lot of the elderly people forget what they can and cannot do. So many of them think they can walk. They want to be independent and they’re not able to because of physical disabilities. So, we’re trying to keep them safe, but I think at times they feel like we’re trying to hold them back and which will aggravate them even more. (Pg 4 resident behaviours, paragraph 3) |
| **Study:**Kaasalainen et al., 2019 | |
| Finding | Understaffing (U) |
| Illustration | I think there would be a need for more staff or at least more volunteers because I think it would be manageable with the appropriate amount. (Site 2, daughter, page 3) (Pg 10 Recommendations to implement ‘Namaste Care’ in new LTC homes, 4) |
| Finding | staff burden over time (U) |
| Illustration | It’s really really busy in the morning that we usually find it hard for us because you can’t be alone and we still have people in bed and you have to wait for the other side to get help, but the other side maybe they are still busy too you know. That’s the struggle that we have right now. (Site 2, PSW/CA, page 1) Being that is a very busy time of the morning, and getting people there you know with the care aides it is hard to get people there in the morning, and we don’t have the staff. (Site 2, recreation therapist, page 1) There have been families that have complained ...that they don’t like their people not getting enough attention, because someone has gone off the floor. (Site 2, PSW/CA, page 3). The care needs now are much, much heavier than they were 10 years ago ..., I think there are many staff that are almost overwhelmed in their day-to-day responsibilities without Namaste. (Site 2, nurse, page 3) (Pg 9 Barriers to implementing ‘Namaste Care, paragraph 2) |
| Finding | adverse events such as incidence of skin breakdown (U) |
| Illustration | When we initially started putting residents in there and they went for the four hours a day. Those residents who had maybe previous ulcers, re opened. (Site 1, director of care, page 2) Well it doesn’t hurt them, the residents. Except there are some that can’t go twice a day because they have skin break down, I mean they just can’t handle it body wise. (Site 2, PSW/CA, page 3) (Pg 9 Barriers to implementing ‘Namaste Care, paragraph 4) |
| Finding | Lack of funding (U) |
| Illustration | If we could have funding for someone to be in there, I would definitely be on the waiting list to try and apply for that position. (Site 2, PSW/CA, page 4) (Pg 10 Recommendations to implement ‘Namaste Care’ in new LTC homes, 5) |
| Finding | strong support from administration (U) |
| Illustration | Oh, the staff comes to me and tells me there’s nobody in the room so I have to organise with the staff who is willing to go and who is willing to stay on the floor. So, we talk, like the team on the first floor we talk about who can go. (Site 1, nurse, page 2) (Pg 9 Facilitators to implementing ‘Namaste Care’, paragraph 1) |
| **Study:** Kwak et al., 2021 | |
| Finding | Using headphones (U) |
| Illustration | “They liked hearing all their favourite songs back-to-back. Many will smile when we put it on them. They like knowing that they have their own music that they don’t have to share.” (Pg 8 What residents liked about M&M, paragraph 1) |
| Finding | inadequate staffing (U) |
| Illustration | Other related barriers included inadequate staffing (n = 9) (Pg 8 Implementation barriers, challenges, and sustainability, paragraph 1) |
| Finding | Use of technology (N) |
| Illustration | Use of technology was another major barrier (n = 62). Respondents reported issues related to charging the equipment (n = 22), using iTunes (n = 4) and Wi-Fi (n = 3), downloading music (n = 3), as well as other equipment issues (n = 17), programming issues (n = 3), and problems with accessibility to iPods or a computer to load songs (n = 12). (Pg 8-9 Implementation barriers, challenges, and sustainability, paragraph 2) |
| Finding | family and volunteers’ involvement (N) |
| Illustration | family involvement (bringing music, donating shuffles to use, turning the iPod on, being supportive of the program) (n = 33). (Pg 9 Facilitators of Providing M&M., paragraph 1) support from others (mostly volunteers) (n = 15) (Pg 9 Facilitators of Providing M&M., paragraph 1) |
| Finding | Calming effect of music (U) |
| Illustration | One respondent stated that “it triggered memory and seemed to calm behaviors during times of sun downing.” (Pg 8 What residents liked about M&M, paragraph 2). Another respondent stated that “they seem more alert. Some residents will often sing along or hum to the music they hear, others will listen and often brings a smile to their face,” noting how residents seemed more engaged with their social environment and expressive. (Pg 8 What residents liked about M&M, paragraph 2) |
| Finding | support of facility personnel (N) |
| Illustration | The most frequently cited facilitator was support of facility personnel (basically all staff including administrators), family, and volunteers to implement and maintain the program (n = 55) (Pg 9 Facilitators of Providing M&M., paragraph 1) |
| Finding | Funding (N) |
| Illustration | financial support and donations (n = 20) (Pg 9 Facilitators of Providing M&M., paragraph 1) equipment donations (n = 15). (Pg 9 Facilitators of Providing M&M., paragraph 1) |
| Finding | Lack of time (U) |
| Illustration | Respondents listed lack of time required by the program—time to “set up and maintain the playlist,” “distribute iPods,” “keep the program up to date and current for new residents,” and “assure iPods is still playing and follow up.” (Pg 8 Implementation barriers, challenges, and sustainability, paragraph 2) |
| Finding | Lack of buy-in by direct care staff (N) |
| Illustration | Lack of buy-in by direct care staff (e.g., nurses, CNAs, and other direct care staff) to initiate, deliver, and follow up with M&M for residents was the most frequently cited barrier to providing M&M (n = 85). (Pg 8 Implementation barriers, challenges, and sustainability, paragraph 1) |
| Finding | difficulty in identifying specific songs for the playlist (N) |
| Illustration | identifying preferred songs (difficulty in identifying specific songs for the playlist), finding music (n = 22), |
| Finding | Seeing the positive effects of M&M (U) |
| Illustration | Seeing the positive effects of M&M on residents and residents’ characteristics (e.g., being calm, enjoyment, residents wanting to listen to music) was another facilitator (n = 43); so too was, as one respondent said, “CNA staff observing success.” (Pg 9 Facilitators of Providing M&M., paragraph 1) |
| Finding | need to educate initial staff and new staff (N) |
| Illustration | the need to educate initial staff and new staff due to turnover (n = 14) (Pg 8 Implementation barriers, challenges, and sustainability, paragraph 1) |
| Finding | Costs for buying music (N) |
| Illustration | Costs for buying music, iPods, and headphones were also a barrier (n = 28) (Pg 9 Implementation barriers, challenges, and sustainability, paragraph 3) |
| Finding | Lack of resident buy-in (N) |
| Illustration | resident buy-in (n = 19). (Pg 9 Implementation barriers, challenges, and sustainability, paragraph 3) |
| Finding | lack or inconsistency of volunteers and family support (N) |
| Illustration | Other barriers included lack or inconsistency of volunteers (n = 3), and families that were not supportive or helpful (n = 12). (Pg 9 Implementation barriers, challenges, and sustainability, paragraph 3) |
| Finding | training and support for M&M (N) |
| Illustration | Other facilitators included having been provided training and support for M&M (n = 33) (Pg 9 Facilitators of Providing M&M., paragraph 1) |
| Finding | accessibility of equipment (N) |
| Illustration | accessibility of equipment (n = 31); equipment characteristics, such as being portable, small, and easy to use (n = 21); (Pg 9 Facilitators of Providing M&M., paragraph 1) |
| Finding | Lack of being valued by the residential aged care facilities (U) |
| Illustration | My concern as a music therapist is that facilities will think they can just throw headphones on seniors to give them music, which could in turn devalue the work of a music therapist, costing music therapists their jobs. Music listening is a very tiny piece of the puzzle of the power of music. It can also do damage if not administered properly.(Pg 7 Value of M&M., paragraph 3) |
| **Study:** Janzen et al., 2013 | |
| Finding | time constraints and low staff-to resident ratios (U) |
| Illustration | An RN described the typical situation, ‘‘... at times there’s so little staff and there’s a lot of behaviors all at once. It’s just kind of putting out fires and keep things rolling ... ’’ (Pg 6 Facilitators and Barriers for NPI Implementation in LTC, paragraph 3) |
| Finding | empathy exhibited by the staff. (U) |
| Illustration | A highly empathetic unit manager understood that living in a complex LTC environment influenced adverse behavioural responses, ‘‘imagine leaving your own environment, coming into a place where it’s not familiar ... living with other unfamiliar people ... and having care done by people you don’t recognize.’’ Empathy of the staff appeared to coincide with openness to using NPIs (Pg 6 Facilitators and Barriers for NPI Implementation in LTC, paragraph 2) |
| Finding | perception that NPI application was based on trial and error (N) |
| Illustration | Multiple factors (e.g., time of day, personality of the staff or resident, and environment) made the outcome of the NPI use unpredictable. |
| Finding | Need little training to implement (Pg 6 enhancing factors and Hindering factors for NPI Implementation in LTC, paragraph 2) (N) |
| Illustration | none |
| Finding | familiarity with the resident (U) |
| Illustration | A recreation coordinator explains ‘‘ ... the more time you [staff] spend with them [residents], the more you figure out what works. So, consistency in staff and routine for them [residents] is [a] big [factor].’’ (Pg 6 Facilitators and Barriers for NPI Implementation in LTC, paragraph 1) |
| **Study:**Kong & Kim, 2022 | |
| Finding | Inappropriate physical environment (U) |
| Illustration | Participants said that sharing a room made it difficult for residents with dementia and/or families to decorate the room or behave as they would, which hindered staff members' implementation of respecting the preferences of residents with dementia: Personally, I ask residents and their family not to bring special, precious, or expensive personal belongings to the room unless residents strongly want them because of the worry of the possibility of loss. (Participant 1, RN) (Pg 5 Inappropriate physical environment paragraph 1). Cognitively intact residents who are somewhat aggressive don't like the repetitive behaviors of residents with dementia, so they become unkind to those residents with dementia, which makes residents with dementia more withdrawn …. There are many conflicts between them. (Participant 2, RN) (Pg 5 Inappropriate physical environment paragraph 1). On the second floor, there is only one room where residents with severe dementia stay together. I think there should be more space for residents with dementia to stay, wander around, and receive personcentered dementia care without interfering with other residents' lives. (Participant 20, care worker) (Pg 5 Inappropriate physical environment paragraph 2) |
| Finding | Staff’s negative attitudes (U) |
| Illustration | One participant said, ‘I think that some staff's mindset was wrong. They do not have a sense of mission’ (Participant 1, RN) Some staff have worked for a long time so they have mannerisms… When we care for residents with dementia, we have to talk to the resident more and provide any stimulus, but they only change diapers, take the residents to physical therapy, and feed them lunch; that's it. I feel sorry about that. (Participant 13, RN) (Pg 6 Staff's negative attitudes, paragraph 1) The problem is that some senior staff don't want to provide person-centered care for residents with dementia. They are lazy. So some passionate new staff are bullied so they are discouraged in the provision of person-centered care. (Participant 2, RN) (Pg 6 Staff's negative attitudes, paragraph 2) |
| Finding | Lack of communication among staff, residents and families (U) |
| Illustration | Some families are very sensitive, so it is better put some distance between. They misunderstand my words and then they go to the office of administrator and complain about that. So it is very difficult to communicate with them. (Participant 8, care worker) (Pg 7 Lack of communication among staff, residents and families, paragraph 1) When families institutionalize their loved ones, they do not share one hundred percent of the information about their loved ones. They seem to be afraid that their loved ones will be rejected [for admission] by the nursing home. Families do not tell us details about their loved ones, which hinders our implementation of person-centered care for residents with dementia. (Participant 19, NA) (Pg 7 Lack of communication among staff, residents and families, paragraph 1) When an older adult is admitted to the nursing home, a nurse and a social worker take a history of the older adult. Usually care workers do not attend. Although we share important information, it is impossible to share all the information about the residents among 10 care workers on the team for the implementation of person-centered care. (Participant 3, care worker) (Pg 7 Lack of communication among staff, residents and families, paragraph 2) |
| Finding | Family’s lack of education (U) |
| Illustration | Many families don't understand the dementia of their loved ones. As the residents with dementia are getting older, their dementia status is getting worse. But families don't understand the deterioration and show very sensitive response. (Participant 1, RN) (Pg 6 Family's lack of education, paragraph 1) There is a lack of understanding about dementia care among families of residents. We try to provide families with some education when meeting with them. But the number of families who participate in those meetings is usually very small … many families usually have little knowledge about dementia care in our nursing home. (Participant 2, RN) (Pg 6 Family's lack of education, paragraph 1) ‘Many families did not visit often residents with dementia so they did not cooperate with us about person-centered care for those residents’ (Participant 2, RN). (Pg 6 Family's lack of education, paragraph 2) Families seem to need education about dementia and person-centered care. Through the education, they will be able to understand dementia of their loved one and the benefits of person-centered care. (Participant 12, care worker(Pg 6 Family's lack of education, paragraph 2) |
| Finding | Staff’s hurtful experiences (U) |
| Illustration | In my case, I treat residents as my family, but some residents with dementia become violent. They often bite, hit, and spit at me. (Participant 18, care worker) (Pg 6 Staff's hurtful experiences, paragraph 1) Because residents present dementia-related behaviours to care workers who provide the most care … many negative feelings build up, so some care workers treat residents badly in return. (Participant 9, RN) (Pg 6 Staff's hurtful experiences, paragraph 1) Sometimes, familyes are upset, angry, and out of control. When they behave like that, we are so shocked. Some families even say swear words to care workers. (Participant 24, NA) (Pg 7 Staff's hurtful experiences, paragraph 2) Some familyes observe us carefully to find our mistakes and then accuse us. In that case, frankly speaking, we feel bad and become less kind to them. (Participant 7, care worker) (Pg 7 Staff's hurtful experiences, paragraph 2) |
| Finding | Lack of trust between staff and families (U) |
| Illustration | For the successful implementation of person-centered dementia care, I think that families need to totally trust staff. All of us, as a team, have to take care of residents with dementia. When wandering, some residents with dementia might have an accident and get a small bruise or skin excoriation on their arms but some families think it is caused by staff's abuse, there is mistrust. (Participant 2, RN) (Pg 7 Lack of trust between staff and families, paragraph 1) |
| Finding | Insufficient staff (U) |
| Illustration | All participants mentioned insufficient number of RNs, NAs and care workers per resident pointing out inappropriate national staffing standards: Primarily, there are not enough staff compared to the number of residents. Currently, the government mandates at least one care worker for 2.5 residents and one nurse for 25 residents. But considering that we have three shifts per day, two nurses are now expected to take care of 90 to 100 residents and we have three care workers for 24 residents. (Participant 9, RN) (Pg 5 Insufficient staff paragraph 1) |
| Finding | lack of time (U) |
| Illustration | Participants mentioned too many residents and lack of time as difficulties in implementing person-centred dementia care: Now, for us, it's hard to provide person-centered care to our residents with dementia because we have too many residents. For breakfast, I have to assist eight residents, so I am too busy to pay attention to other residents, like what and how much they eat. (Participant 8, care worker) (Pg 5 Insufficient staff paragraph 2) Emotional care requires sufficient time. But we don't have enough time to sit and talk with our residents. (Participant 2, RN) (Pg 5 Insufficient staff paragraph 2) |
| Finding | Staff’s lack of education (U) |
| Illustration | Much of the contents of continuing education focus on acute hospital care. I think we really need this kind of education, person-centered dementia care, in annual continuing education. (Participant 12, care worker) (Pg 5 Staff's lack of education, paragraph 1) Some educations are very theoretical. There is a gap between theory and practice. (Participant 8, care worker) All participants requested continuing practical education of person-centred dementia care especially for care workers, as mentioned by one participant: ‘To provide person-centered care for residents with dementia, care workers should have received education about person-centered dementia care’ (Participant 4, RN). One care worker also said, ‘Nurses take education about dementia through continuing education or on their own, but care workers usually don't do that’ (Participant 3, care worker). (Pg 5 Staff's lack of education, paragraph 2) |
| Finding | Conflicts among/between staff and families (U) |
| Illustration | One time we wanted to place a bedridden resident with dementia in a wheelchair and take her for a walk, but some staff disagreed. So we could not do it. Although we were co-workers, our opinions were often different. (Participant 10, care worker) (Pg 7 Conflicts among/between staff and families, paragraph 1) When there are accidents, some families grill us about whose fault it is. (Participant 3, care worker) (Pg 7 Conflicts among/between staff and families, paragraph 1) [To provide person-centered care] we have to establish good relationships with families. If family cooperate well, it is easy to discuss and deal with the behaviors of the residents with dementia. But there are many families who do not cooperate. (Participant 2, RN) (Pg 7-8 Conflicts among/between staff and families, paragraph 2) |
| **Study:**Gulliver et al., 2021 | |
| Finding | insufficient time (U) |
| Illustration | Several staff members believed insufficient time was a significant barrier to conducting these types of activities – “No…they’re focused on getting their care work done…to do something like that you need someone that’s going to sit there with them” (S2). (Pg 7 Issues in continuing the program. Paragraph 1) |
| Finding | Fear of not producing a good voice (U) |
| Illustration | Other staff were adamant that this would not be possible, suggesting staff may be “a little bit frightened of letting their voice out and… because it is a daunting thing” (S2), and that they felt you had to have “a good voice” (S3). (Pg 7 Issues in continuing the program. Paragraph 1) |
| Finding | Staff familiarity with music (U) |
| Illustration | One staff member believed that since they already conducted musical sessions, they would not find it difficult - “one of the family member’s daughter, she plays the piano. And then we had another family member, the son plays the ukulele…they come in and we do the singing” (S5). (Pg 7 Issues in continuing the program. Paragraph 1) |
| **Study:**Van Der Ploeg et al., 2012 | |
| Finding | bonding between residents and volunteers as positives (U) |
| Illustration | I think if they didn’t have the volunteers then my thinking would be that they’re not stimulated enough to self-motivate and stimulate themselves, so therefore they would just sit there and drop off to sleep. (#13) (Pg 5 Perceived benefits and difficulties, paragraph 3) And the fact that somebody’s on one to one basis with the residents, that’s the whole ... it’s not about [painting of] the [finger]nails, it’s about the contact. They [residents] often open up to the volunteers better than to staff, because we basically attend, some of us attend to tasks only, not necessarily to do ... be ... a holistic communication. (#4) (Pg 5 Perceived benefits and difficulties, paragraph 3) |
| Finding | Staff perceived value of volunteers (U) |
| Illustration | Two-thirds of facility staff reported they considered volunteers as a needed extra pair of hands, an additional resource. They are invaluable, volunteers are invaluable. Dementia specific facilities need a high staff ratio and whilst we can cope with the care side of things, the lifestyle, the quality-of-life activities would be desperately lacking if we didn’t have volunteers, especially for people with dementia, because they need more one to one, they need small group activities. (#14) (Pg 4 Perceived benefits and difficulties, paragraph 2) |
| Finding | residents crossed boundaries (U) |
| Illustration | Like if you give out your phone number, not only are you opening yourself up to get random phone calls when you’re not in your volunteer role. I suppose it’s not appropriate to get to that, to keep within those bounds and just remember your place and that you’re not a family member and you’re not a doctor. (#18) (Pg 5-6 Perceived benefits and difficulties, paragraph 11) |
| Finding | Being asked to perform jobs that only staff members are trained to do. (U) |
| Illustration | Yes sometimes they ask me to take them to the toilet and I say “Sorry, I can’t do it.” (#41) |
| Finding | interacting with older people was enjoyable as perceived by volunteers (U) |
| Illustration | I have laughter here, belly laughter .... So I feel quite relaxed and like this is my second home. I get more back from what I give them, I receive more so I’m leaving here quite uplifted. (#29) |
| Finding | managing a range of personalities in group activities (U) |
| Illustration | Not towards me, not towards volunteers no, but I see things that I don’t like .... There was somebody who couldn’t breathe, he couldn’t talk and I said “look this man needs some oxygen, he can’t breathe.” (Staff response:) “Oh we don’t.” I said, “You’ve got to have oxygen, of course” and she repeated “We don’t have oxygen” and she just didn’t care. And because I insisted she went and got the bottle of oxygen to help. See things like that. (#42) (Pg 5 Perceived benefits and difficulties, paragraph 9) |
| Finding | witnessing agitation as a Hindering factors (U) |
| Illustration | There’s a lady here right this moment who is very distressing. She’s ... you might have heard her calling out. (#38) One thing that’s overall distressing is watching people become sicker and sicker, and eventually be moved on ... it’s also a sad thing to watch people suffer from dementia. (#47)(Pg ) (Pg 5-6 Perceived benefits and difficulties, paragraph 12) |
| Finding | increased well-being for resident as perceived by volunteers (U) |
| Illustration | When one of them is really depressed and down, the lifestyle coordinator lets me know to begin with so I can spend more time, but when they put the dog on their knee ... just the look on their face and they profusely say “thank you for bringing him.” (#30) And there’s so many out there that feel so alone, and missing their home and their own belongings and just being able to express what they feel, I feel, is very beneficial for them. (#22) (Pg 5 Perceived benefits and difficulties, paragraph 5) |
| **Study:** Backhouse et al., 2016 | |
| Finding | view that activities or NPIs are extras (U) |
| Illustration | At times of staff shortages, the activity staff would often be reallocated to their other roles, meaning no activities occurred on those days. a lot of people just see the activities side as a bolt-on’ (Susan, Manager, CH2) ) (Pg 5 Barriers to including residents in activities, paragraph 7) |
| Finding | inequality in the allocation of activity provision (U) |
| Illustration | that are able to voice or … can show their frustrations about not having something to do, whereas other people that are sitting in their chair might feel equally as bad, but can’t voice it or express it in any way (Barbara, General Nurse, CH3 (Pg 5 Barriers to including residents in activities, paragraph 5) it’s normally the same people, the same little group (Holly, Activity Worker, CH2) (Pg 5 Barriers to including residents in activities, paragraph 4) |
| Finding | feeling ‘uneasy’ around those with BPSD (U) |
| Illustration | It’s like outings … I don’t think they’re [care staff] willing to help out as much because they don’t want to help people with dementia. Where they’re more willing to help people that … have got their full faculties … so I think some dementia people do get, um, misunderstood and mistreated, um, not saying physically mistreated, or, I’m just like neglected with … activities (Karen, Activity Worker/Senior Care Worker, CH1) |
| Finding | physical or mental impairment (U) |
| Illustration | I feel awful saying this … it’s just very hard to actually get them to do, that they can physically do anything .. once they [care staff] did suggest may be go and put a tambourine in their [the residents’] hand … I just found that must be patronising to be honest .. I didn’t feel comfortable doing that … I suppose I’ve kind of veered away from those residents um, because … it’s very hard to know activity wise what to do. (Jess, Activity Worker, CH3) (Pg 5 Barriers to including residents in activities, paragraph 1) |
| Finding | Residents’ reluctance to take part in activities (U) |
| Illustration | Holly, an activity worker, touches on the issue: I have to try and get them … say ‘oh come on, do you want to do it?’ ‘no, no, no’ ‘come on’ but once they’re doing it they’re absolutely fine, it’s like when we done all the sunflowers … Mable was going ‘oh I can’t do that, I can’t draw’ but … she absolutely loved it in the end (Holly, Activity Worker, CH2) (Pg 5 Barriers to including residents in activities, paragraph 9) |
| **Study:**Clifford & Doody, 2018 | |
| Finding | Lack of management support (U) |
| Illustration | “Nursing management need to be on board, they need to understand, they wouldn’t understand what it is you are trying to do for the patient, I don’t think they understand why you are harping on about the compliment of staff and why you are constantly looking for (additional resources)” (P5). (Pg 7 The care environment, paragraph 4) |
| Finding | Shortage of staff (U) |
| Illustration | “When you are down staff, your hands are tied because you can’t provide the quality of care that you want and it’s frustrating because you know the other staff are going to get stressed” (Pg 6 Resources and interventions to support people with dementia and responsive behaviour , paragraph 2). |
| Finding | Collaboration between nurses and activities coordinator (U) |
| Illustration | “While we have an activities coordinator we also do dementia specific activities, we do reminiscence, relaxation therapy, and we give each other feedback to say they enjoyed it” (P9). (Pg 6 Resources and interventions to support people with dementia and responsive behaviour, paragraph 4). |
| Finding | good communication skills (U) |
| Illustration | “..good communication skills, making sure you explain everything you are going to do, having regular meetings with staff that are looking after someone and discuss the care plan, discuss their care needs, discuss with the family more often ” (P7). (Pg 7 The care environment, paragraph 7) |
| Finding | getting to know the person (U) |
| Illustration | “You need to get to know the person, to know their life, their history, to know everything about them, what they worked at, what they like to eat, their family, all their likes and dislikes” (P5). (Pg 6 Resources and interventions to support people with dementia and responsive behaviour, paragraph 5). |
| Finding | Lack of education and training (U) |
| Illustration | “Knowledge with nursing staff is at a level where they can understand, however, for the caring staff, mainly their knowledge isn’t the same, some of them have attended training, sometime their knowledge lacks in ways that it makes it hard for them to respond to responsive behaviours or try to deal with a situation” (P9). (Pg 7 The impact of education on nursing practice, paragraph 4) |
| Finding | Frequency and severity of resident's behaviour (U) |
| Illustration | “We are used to dealing with people that strip or call out, or spit, but if it’s ongoing, if it’s constant every day, that puts too much pressure on and it’s stressing for them and other residents” (P3). (Pg 7 The care environment, paragraph 3) |
| Finding | attitudes and being self -aware when caring (U) |
| Illustration | “You have to be calm, you have to monitor your own behaviour, you have to respect their space, what they want, what they don’t want regardless of the fact that they might not be able to express it” (P6). (Pg 7 The care environment, paragraph 5) |
| Finding | continuing education (U) |
| Illustration | “For someone that had no understanding of dementia care I learned from experience and interacting with people, going on day courses, but that three-day course while intense, it gave a huge amount of tools to manage people with dementia, and to manage behaviours that are challenging and an overall view of how the person with dementia can act” (P8). (Pg 6 The impact of education on nursing practice, paragraph 1) “I must say much of it (information learned) would have been experience on the job, the study days have provided the tools and the mind -set so that you could go about it a different way” (P4). (Pg 6 The impact of education on nursing practice, paragraph 2) “It definitely would pay off for anyone working in dementia care to have some training in dementia and top up session, definitely a course or some documentation that they could sit down and actually read, just to understand what might be going on” (P9). (Pg 6 The impact of education on nursing practice, paragraph 3) |
| Finding | lack of funding (U) |
| Illustration | “..it (the programme) finished because of the cost of the programme and the cost of care is not reflected in the fees that are negotiated through the National Treatment Purchase Fund” (P8). (Pg 6 Resources and interventions to support people with dementia and responsive behaviour, paragraph 3). |
| Finding | lack of manager support by scapegoating (U) |
| Illustration | “Management would say that if the resident needs behaviour management, that maybe here is not the best facility for them, should we consider special dementia care unit, so we don’t get any support” (P1). (Pg 7 The care environment, paragraph 6) “I think my colleagues would think at times that the person is doing it out of spite, it’s the person not the dementia’s fault.” (P5). (Pg 7 The care environment, paragraph 6) |
| **Study:**Chaudhry et al., 2020 | |
| Finding | Workload with engagement of staff in routine activities (U) |
| Illustration | ‘Many times, I had to send your team back without doing any work. I am very sorry for that as sometime our staff are bit stuck in other activities that’s why I was unable to get participant to deliver some of your sessions on time’ (PCW2); ‘Every care worker has different responsibilities and schedule due to which it is difficult for all of us to attend something at same time’ (PCW3) |
| Finding | fluctuations in mood, behavioural problems, verbal outbursts of residents (U) |
| Illustration | ‘Yes, they do have a lot of mood swings. At one moment they are very cooperative, but in the next they become totally opposite, like at one moment they agreed to take a bath, but when they were taken to the washroom, they started beating us. Their behaviour changes so abruptly’ (PCW2). (Pg 5 table 2 Experience of working with older adults) |
| **Study:** Nunez et al., 2018 | |
| Finding | Nurse burden and responsibilities (U) |
| Illustration | ‘And simply because they have to air their feelings, you know? They get frustrated. Sometimes they may feel that they are delegating their duties to the best of their ability, and the staff … So it’s unfortunate sometimes that the night nurse feel that they have not got that assertiveness to be rude to—they do not like the word, instruct. You know? Tell, the staff, “This is what is required to do.” Because they should know anyway. But there is that kind of an issue sometimes’ CSFG 2 (Pg 7 table 1 Nurse burden and responsibilities) ‘They have to make decisions. You know, really life-threatening decision. And sometimes you think you are doing the right thing and sometimes you might not, so you always need to have a second opinion. But if you have had a fairly regular training and you are supported by the management team... but it’s always handy, reassuring when someone is actually working together with you, I think. Then you feel that whatever is going to happen, you are going to have a reflective practice, and to learn from that. Because everybody is a human being and you make mistakes.’ CSFG 2 (Pg 7 table 1 Nurse burden and responsibilities) |
| Finding | Difficulty of connecting with and knowing residents (U) |
| Illustration | ‘Because we are a diverse team ... But due to cultural differences we have found that some of the staff have got great difficulty in connecting and engaging with the residents. CSFG 2 ‘It’s just the little touches and if somebody comes in and is told, “Put bed number 24, 25 and 26 to bed now,” they do not know what their routine is. My mother cannot tell them. So sometimes it’s a bad night’s sleep.’ FCFG 2 (Pg 6 table 1 Connection and knowing a person) |
| Finding | Insufficient staffing levels (U) |
| Illustration | ‘I know when I see one nurse and one care assistant on duty for a whole floor, there’s shouting going on.’ FCFG 2 (Pg 6, table 1 Insufficient staffing levels) ‘Is there any factors which affect your ability to provide good care at night?’ ‘It’s always short-staffed.’ FCFG 1(Pg 6, table 1 Insufficient staffing levels) ‘I was worried at night because I knew there were less staff on. It was more likely that it would happen when nobody was there to stop him.’ FCFG 1(Pg 6, table 1 Insufficient staffing levels) ‘Not enough staff’ FCOS ‘But sometimes, not matter how much surveillance you can have, accidents are going to happen. Yes? You could have somebody who falls in front of you. So nighttime, you could be dealing with somebody having heart failure. Another person falling down at the same time. And they have to deal with emergencies, so the nurses have to think very, very quickly.’ CSFG 2 ‘She fell off a chair on one occasion, and when I came in the next time, I could see that they were more concerned that I was going to sue them than they were about sorting— it’s health and safety gone mad, which is true in so many areas of society at the moment.’ FCFG 2 (Pg 7, table 1 Insufficient staffing levels) |
| Finding | Night and day care staff working relationship (U) |
| Illustration | ‘Do not worry about it, the day staff will be in a minute. It’s always like this battle [laughter]. That’s what we need to stop; we need to work as one, as opposed to working as a battle against each other. “We are better than them.” Or “They are better.” “We do a better job.” Or “We do not know what we do.” I think, all staff, really, should workday and that we should work night’ CSFG 2 ‘they will blame it on the night staff, because they think they have not attended to them. It’s just somehow you need—both parties have to know their residents. So that if you know somebody is resistant to care, you know you are not going to be able to attend to this person when you think you want to give that care.’ CSFG 2 ‘ (Pg 7 table 1Night and day care staff working relationship) ‘Because we are thinking, “It’s harder in the day than night, because they are just sleeping.” All the patients are just lying in their bed’ CSFG 2 ‘So, you need tell your colleague, “Look, we have tried, tried, tried. It did not work out.”’ So then there’s not the kind of surprise as, “Oh, my God, look at that. They have let it go to us and we are not able to fix it.”’ CSFG 2 That is a day plan, okay? So I doubt very much again—not being sort of overcritical. Those staff where my wife was, they come for the evening, for the night shift, and they want to have a good sleep, the staff. FCFG 2 (Pg 6 table 1 Connection and knowing a person) |
| Finding | Communication as a challenge (U) |
| Illustration | ‘Staff have to know the care plan of the residents and do they miss out on days, because of days off. And when they come on duty, they know...aware of any changes of the person’s condition. Because that’s forever changing. ’CSFG 2‘so although I did ask the respite care manager to let me know whether they administer any sleeping medication or to keep her agitation down, they never did—’FCFG 2‘I think the only feedback I got about nights was if something had basically happened. If he had been in some sort of incident, they are legally obliged to tell you anyway...But I knew if I came in at, say, 11:00 in the morning and he wasn’t there, then something probably went wrong at night. ’FCFG 1‘I’d see my husband had bruises. I’d look and I’d think, “How? He could not get out of bed. “You know they never volunteer the information; you have to go and ask. ‘FCFG 2‘with the first 2 ambulance trips in at night because of pneumonia, I found out that he’d been put on haloperidol. This was to stop all the problems the care home was having with him at night, but I’d never been told about them, or the fact that they’d asked his GP or Rx haloperidol for him. ’FCOS (Pg 5 table 1 Communication as a challenge) |
| **Study:**McKenna et al., 2022 | |
| Finding | Being newer staff (U) |
| Illustration | …this is useful for someone who first comes in… [Participant 1] (Pg 6 Theme 3: Understanding, paragraph 3) … there’s a lot of new staff, and I think sitting down and actually listening to somebody think of why she’s actually behaving like this, I think they’d [like to] know… [Participant 9] (Pg 6 Theme 3: Understanding, paragraph 3) |
| Finding | Being pessimistic (U) |
| Illustration | …not everyone with dementia can be fixed… [Participant 1] …[following the intervention] there was nothing more we could possibly do with her […] we basically can’t try anymore… [Participant 9] (Pg 4 Theme 1: Expectation, paragraph 8) |
| Finding | Team working (U) |
| Illustration | … the rest [of the ideas] was from us. But she was just inputting what could you do to, you know, like steer him away from getting agitated, getting aggressive, and this is what we come up with […] so it should be quite simple, you know to put together… [Participant 10] (Pg 6 Theme 2: Working together, paragraph 6) involve like a more, like a two way sort of, questioning between both. The psychologist could say something, and then the staff member could say, “well I’ve tried this but when I was trying this he or she did this”, so we know it’s not working this way. So it could be more like a two-way sort of thing… [Participant 3] (Pg 6 Theme 2: Working together, paragraph 7) |
| Finding | absence of joint working (U) |
| Illustration | …she [the psychologist] thinks she read it in a book and she’ll come and tell us what we’re doing wrong or right I think. I’ve known [the resident] for years and, and you just come and you say, “it’s not”. I don’t say it but some say, like a bit of a kid coming in telling me how to, you know, tell your grandma how to suck eggs… [Participant 1] (Pg 5 Theme 2: Working together, paragraph 3) |
| Finding | Intervention not meeting staff’s expectation (U) |
| Illustration | …at first, I was happy, ‘cos I was thinking, it’s [psychological formulation] gonna really help us…It’s just pointless. You’re doing, you’re doing the jobs…but then the behaviour’s still happening, but it’s like, you’ve done the recommendations so everything should be fine… [Participant 2] (Pg 4 Theme 1: Expectation, paragraph 5) …you think the problem’s gonna be solved…I think, at first you’re looking for a quick fix aren’t you? You think someone’s here that can fix it all […] we’re always open to new ideas and that’s what you hope for I think when they come - new input. But it doesn’t seem to be the case… [Participant 1] (Pg 4 Theme 1: Expectation, paragraph 6) |
| Finding | Sense of frustration and powerlessness that their knowledge and experience undervalued (U) |
| Illustration | …I work on the floor where she comes, that’s my, the floor I work on, and she hasn’t spoke to me once…I don’t feel like I can approach them, I wouldn’t approach them to ask them, any information…like I say they don’t really acknowledge, they just come in, do their job and then they go… [Participant 2] (Pg 5 Theme 2: Working together, paragraph 2) |
| Finding | lack of originality of the psychologist suggestions (U) |
| Illustration | …you do understand what they’re saying, but you think, well, we knew that anyway (laughs). I know it’s a bit awful to say that, but we always say, well we’ve known that, that’s what we’ve done… [Participant 3] …she’s only told us what we know. She’s not told us any other ways to deal with him… [Participant 4] (Pg 7 Theme 3: Understanding, paragraph 5) |
| Finding | Benefit gained(understanding) from the intervention or confidence (U) |
| Illustration | …so yeah, I understand a little bit, but now I actually know that I can, I can do that, and have the confidence in doing it or saying, it’s alright I’ll have a go, I’ll try… [Participant 5] (Pg 6 Theme 3: Understanding, paragraph 2) |
| Finding | Little awareness of psychologist support (U) |
| Illustration | …I couldn’t tell you what, what psychology means […] will it change the person? Will it do anything?... [Participant 7] (Pg 4 Theme 1: Expectation, paragraph 3) |
| **Study:** Griffiths et al., 2019 | |
| Finding | Staffing related issues (lack of nurses and other staff, staff turnover, mappers attrition) (U) |
| Illustration | “Because we are only a residential home, erm, y’know, we haven’t got nurses and stuff so my staff aren’t that confident anyway… I’m glad we got involved because we got a lot out of it, I’m just disappointed that we weren’t able to continue.” (Manager) Pg 4 “Care homes are really, really busy. Turnover of staff in care homes can be quite dramatic at times, and the realities are there’s other pressures on them isn’t there.” (DCM™ expert) Pg 5 “It were definitely better having two rather than having just doing it on my own, because I think I would’ve struggled a lot more” (Mapper) Pg 6 |
| Finding | Staff reluctance to support the mapper (U) |
| Illustration | “I would say in that home there’s two very definite groups of staff, the ones who want to see progress, who would support the mapper, who would want to encourage her and make it work, and there was also a very strong group of people who say you know ‘what she thinks she’s telling us’.” (DCM™ expert) Pg 8 “It became a chore and one lady I can think of in particular was very excited and motivated about it, and became less so because of the challenges. And that’s really sad to see. Someone who had that real passion to just go “do you know it’s just too hard”, but initially is like “I’m happy to come in on my day off because I think it’s marvellous”, but when you’re not then getting that support it you know wears you out really. Wears you down.” (DCM™ expert) Pg 9 |
| Finding | Complex nature of DCM™ (U) |
| Illustration | “Some of the things that certainly I picked up on, some of the things they found more difficult was around the kind of data analysis and report writing. That was the area that people seemed to find most difficult.” (DCM™ expert) Pg11 “So the report writing, yeah, was horrific to be honest. Very time consuming. Obviously we both had different roles at that point so quite demanding, so getting time, and it’s not a very quick process. Like I say it took quite a lengthy period of time … it was very demanding.” (Mapper) Pg11 |
| Finding | whole home’ engagement approach (U) |
| Illustration | “There was a really big crowd actually, and it did include lots of different disciplines of staff, including the painter and decorator and maintenance man, which was great.” (DCM™ expert) Pg 7 |
| Finding | Negative attitude towards DCM™ (U) |
| Illustration | “I felt that the ways that people had been working prior to that, the culture of the place, whilst there was a lot about it which I would really commend it for, there were definitely some things that needed to be looked at. And I felt that there was a reluctance to look at that. And there was quite a lot of defensive response.” (DCM™ expert) Pg 7-8 |
| Finding | Staff time constraints (U) |
| Illustration | “It’s all getting the time and people are rushed off their feet in the morning and they haven’t got time to come here for half an hour.” (Mapper) Pg 6 |
| Finding | difficulty to engage the staff team (U) |
| Illustration | “The second time around we held a meeting and nobody came … We did try like you know individual, a few minutes at a time, but I don’t think they took it seriously enough, do you know what I mean?” (Mapper) Pg 8 |
| Finding | Collective reflection on DCM™ feedback (U) |
| Illustration | “In most cases when it happens, it’s a negative experience because there’s inspectors from various organisations, so I think it wasn’t until we started giving feedback and there was quite a bit of positives in there that the staff really got engaged with the process.” (Manager) Pg 8 |
| Finding | The hierarchical nature of care homes sometimes acted as a Hindering factor (U) |
| Illustration | “It’s mainly from a confidence perspective, [they] were clearly not confident to challenge a manager who was not supporting.” (DCM™ expert) Pg 7 |
| Finding | Lack of Care home manager support (U) |
| Illustration | “As far as I’m aware they were just pushed to be doing other stuff and it kept getting left and left and not done.” (Staff Member) Pg 6 |
| Finding | Competing priorities (U) |
| Illustration | “We get inspected by health and safety, infection control, the social workers, CQC [regulatory authority] come, social services come, y’know, it’s just ongoing and they are all asking for more paperwork… we are struggling to do the paperwork that we have got already.” (Manager) Pg 6 |
| Finding | Time gap between training and undertaking DCM implementation (U) |
| Illustration | “They went for that training … then there was a gap and I kind of think if they had just gone straight in and done the mapping, they might have done it. But I feel that when a few weeks passed, they were struggling to say how we do this… maybe they didn’t have the confidence, you know what, to roll it out.” (Manager) Pg 7 |
| Finding | Care home manager support (U) |
| Illustration | “I think management support, you know, it can either be amazing when it’s amazing or it can be a real difficulty if the manger isn’t supportive.” (DCM™ expert) Pg 7 “They were very clear that they thought DCM™ was fantastic, because they saw it as a way of improving the quality of their care to take their home CQC [UK regulator of care homes] rating from good to outstanding.” (DCM™ expert) Pg 7 |
| Finding | Lack of skills or experience (U) |
| Illustration | “For some of the care workers writing anything was a real challenge. You know they just not, not used to putting descriptions down, let alone sort of feedback type questions to ask.” (DCM™ expert) Pg10 |
| Finding | DCM™ did not suit some residents (U) |
| Illustration | “…some of our residents are quite, quite poorly so it doesn’t work for them, it just depends how well they are.” (Staff member) Pg 8 |
| Finding | Staff motivation (U) |
| Illustration | “The manager would come in and you know be really enthusiastic. They came to the briefing, everybody was at the briefing, the whole home, the manager of the home, do you know what I mean. The company really bought, really bought in to DCM™. And the two girls, the two mappers were just really enthusiastic about it, … and really, really tried their hardest.” (DCM™ Expert) Pg 7 “You really have to get quite a few people across the organisation thinking in the same way to sort of drive that change.” (Manager) Pg 7 |
| Finding | Mappers’ time constraints (U) |
| Illustration | “I’d say it depends on your workloads and things that day, like how much is implemented. This thing took a lot of time, when there is not as many staff on as you need, and like I say, we have several people who are end of life, and things like that. Priorities are more that way at the time.” (Mapper) Pg10 |
| Finding | Mappers’ lack of understanding the DCM (U) |
| Illustration | “The trouble is, when they came back [from the training], they weren’t able to explain properly what they had to do. So, you know, they were trying to explain it to us and we were finding difficulty understanding what was actually involved.” (Staff Member) Pg10 “I still don’t understand it … no one has been able to understand it to me fully… Every time I asked them [the mappers] to explain they were struggling. So I never got a full grasp of what it was all about.” (Manager) Pg10 |
| Finding | Mapper status and leadership skills: respect held for the mappers within the home (U) |
| Illustration | “It’s people that you know and peer-led, it’s, you know, it’s not like somebody from outside coming and talking with them, it engages the staff.” (Manager) Pg 8 |
| **Study:**Ervin et al., 2014 | |
| Finding | Lack of effectiveness of the interventions (U) |
| Illustration | ‘‘Very difficult to know which strategy will work with each client’’ ‘‘Resident may not comprehend, and family may not consent to participation’’ ‘‘Depends on day and time. What works once doesn’t work again. They get tired of some strategies being too frequently used. Sometimes improvisation works’’ (Pg 4, Behaviour oriented strategies in dementia care, paragraph 4) ‘‘Sometimes when talking about spouses the demented person thinks they are still alive and starts looking for them’’ ‘‘Some residents become upset talking about family. We have to be mindful of past history’’ (Pg 5, Emotion oriented strategies in dementia care, paragraph 3) |
| Finding | Time constraints (U) |
| Illustration | ‘‘Time limitations, not enough time and staff to cover for 1:1 time for patients’’ ‘‘Staff numbers and time taken to use this strategy is a major limitation’’ ‘‘1:1 time. I feel others miss out while time is devoted to the person with behaviour’’ (Pg 4, Behaviour oriented strategies in dementia care, paragraph 2) ‘‘Time — too busy’’ ‘‘Lack of staff and time’’ ‘‘Time is a huge constraint for nurses’’ ‘‘Lack of staff and time’’ (Pg 4, Stimulation oriented strategies in dementia care, paragraph 2) ‘‘Always very busy and rushed attending to personal care needs. Only get very limited 1:1 time’’ ‘‘Lack of staff numbers to allow time for these strategies’’ (Pg 4, Emotion oriented strategies in dementia care, paragraph 2) ‘‘The greatest impact on good outcomes for behaviour management is time limits. Nurses are always under pressure to hurry. We need more staff allocated to spend time with residents who have behaviour problems’’. ‘‘The biggest problem is time constraints, if we had more staff then we could spend more time with people with behaviours. For one staff member to be devoted to caring for a behaviour management problem leaves the rest of the ward short’’ (Pg 5, Overall comments regarding management of BPSD, paragraph 2) |
| Finding | Lack of dementia specific unit (U) |
| Illustration | ‘‘Aggressive residents are inappropriately placed in facilities who cannot manage. Problem behaviours affect all other residents. Some young staff are intimidated and afraid of aggressive residents’’. ‘‘Residents with behaviours disrupt all residents in the facility and should be in dementia specific units’’. (Pg 5, Overall comments regarding management of BPSD, paragraph 5) |
| Finding | Not viewing as nursing responsibility (U) |
| Illustration | ‘‘Diversional Therapy (DT) is responsible for these therapies as nursing staff are too busy with personal care. Behaviours increase in severity on weekends when DT is absent’’ ‘‘Diversional therapy are involved with this not nursing staff’’ (Pg 4, Cognitive oriented strategies in dementia care, paragraph 2) ‘‘No time for nurses to do this strategy, the domain of DT’s. Evenings are worse as nurses are busy and many residents are sundowners’’ (Pg 4, Stimulation oriented strategies in dementia care, paragraph 3) |
| Finding | Resident’s refusal to comply or participate (U) |
| Illustration | ‘‘Sometimes the residents refuse to participate’’ ‘‘Residents cognitive ability and attention span can limit this intervention’’ ‘‘Residents who have advanced dementia do not respond to these strategies’’ (Pg 4, Cognitive oriented strategies in dementia care, paragraph 4) ‘‘Usually patients are not cognitively able to communicate these memories, those that can seem to become depressed from such discussions and therefore it is avoided’’ ‘‘In severe dementia they don’t even recognise familiar people’’ (Pg 5, Emotion oriented strategies in dementia care, paragraph 4) |
| Finding | Lack of experience (U) |
| Illustration | ‘‘I have no experience’’, ‘‘Lack of experience’’ (Pg 4, Cognitive oriented strategies in dementia care, paragraph 3) |
| Finding | Inadequate staff education as a limitation (U) |
| Illustration | ‘‘Nurses need more training in behaviour management’’ ‘‘The skill mix of the team is sometimes a problem’’ (Pg 4, Behaviour oriented strategies in dementia care, paragraph 3) ‘‘Need education in this strategy’’ (Pg 4, Cognitive oriented strategies in dementia care, paragraph 3) ‘‘We need more training, by the right people, closer to home and at no cost’’ (Pg 5, Overall comments regarding management of BPSD, paragraph 3) |
| **Study:**Garrido et al., 2021 | |
| Finding | “set and forget” attitude (using the music as an entertainment rather than as an opportunity for interaction with the resident) (U) |
| Illustration | “It’s something to interact with. When you’re interacting with the resident not just putting headphones on and walking away, we found that that does not work nearly as well as when you are interacting with the music. (P6 Group 2)” (P, Challenges to Implementing Music Programs in Aged Care, paragraph 3) |
| Finding | tendency for staff to have background music on (U) |
| Illustration | “I’ve had situations where there’s just music on in the background and a lot of our carers, they’re not conscious of it. They’re not thinking of ‘how is music being effective or not effective in this situation’, because for them they can shut it out. It’s just background noise. Whereas it might be really important for the resident. There needs to be something built into the cycle around monitoring it so it really brings it into the consciousness. (P3 Group 1)” (P6, Challenges to Implementing Music Programs in Aged Care, paragraph 4) |
| Finding | Lack of accurate information about individual preferences (U) |
| Illustration | I found it difficult. I had to rely on the music that he had in his CD collection. Communication was thumbs up or thumbs down a lot of the time and he also showed no emotion so it was difficult to know whether he was enjoying it or not enjoying it. (P5 Group 3) (P8, Challenges to Implementing Music Programs in Aged Care, paragraph 10) |
| Finding | Lack of time (U) |
| Illustration | “They would turn the TV set on in the room in the morning … They just didn’t have the staff number to really carry on music therapy, just even playing CDs for my mum. (P3 Group 3)” (P6, Challenges to Implementing Music Programs in Aged Care, paragraph 1) “It seems that time is always a concern as well for staff because they see it as an extra task to be done. I think that’s the result of not understanding how music can benefit them, the resident, and how they can use it. (P4 Group 1)” (P6, Challenges to Implementing Music Programs in Aged Care, paragraph 2) “They’re doing what they have to do in order to make sure they don’t get sued instead of ‘let’s do this to make this person’s life as wonderful as it can be’. It’s a time factor and a culture factor. (P4 Group 3)” (P6, Challenges to Implementing Music Programs in Aged Care, paragraph 2) |
| Finding | Challenge with safe storage of iPod or headphones (U) |
| Illustration | “We had a challenge trying to figure out how best to store them safely while also providing good access to everybody” (P6 Group 2).” (P8, Challenges to Implementing Music Programs in Aged Care, paragraph 8) |
| Finding | not seeing the value of music (U) |
| Illustration | “It’s actually quite a challenging space to provide the evidence, because a lot of the time we’re talking about qualitative perceptions and observations where staff are just saying ‘Oh well Mr Smith seemed happier’, or ‘she was singing’ and things like that … Sometimes collecting that evidence then turns into a barrier. We used to ask people to fill in a little survey after they had listened to music with someone and then I found that people just stopped listening to the music because they couldn’t be bothered to fill in the evaluation. (P3 Group 1)” (P8, Challenges to Implementing Music Programs in Aged Care, paragraph 5) |
| Finding | Difficulty of music individualisation (U) |
| Illustration | “It’s not just a case of having music. It’s being able to hear the type of music that that person can relate to” (P2 Group 3) (P8, Challenges to Implementing Music Programs in Aged Care, paragraph 9) I could not get mum to enjoy really anything new or something she wasn’t familiar with … It didn’t matter how beautiful the music was, how closely related it was to something else that she really liked. If it didn’t strike that familiarity …. (P3 Group 3) (P8, Challenges to Implementing Music Programs in Aged Care, paragraph 9) |
| **Study:**Hussin et al., 2021 | |
| Finding | Lack of Training (U) |
| Illustration | “Only if the centre sends a representative to train the caregivers. It is at our own effort if we want to go for extra courses or classes.” (Shuhada, caregiver) (Pg 8, Barrier: Lack of Training and Skills paragrapgh 2) “I think I have never heard of this Malaysian Clinical Guideline.” (Rahmat, caregiver) (Pg 8, Barrier: Lack of Training and Skills paragrapgh 2) “My friend who was already in this field always taught me how to care for dementia patients. He doesn’t have any certificate of professionalism on how to care for them. So, he taught me only based on his experience.” (Taripnan, caregiver) (Pg 8, Barrier: Lack of Training and Skills paragrapgh 2) |
| Finding | Responsibility outside the job scope (U) |
| Illustration | “They don’t want to bring the mother to the hospital and refuse medication even though we give the options, so it’s like, ‘as long as we pay the monthly fee, then that’s it’….” (Shuhada, caregiver) “Complicated task, such as putting in the catheter, I don’t really know how to do it.” (Taripnan, caregiver) (Pg 12 Barrier: Responsibility outside the job scope paragraph 1) |
| Finding | Inadequate staff numbers and time constraints (U) |
| Illustration | “These dementia residents have more needs than normal elderlies. Sometimes, more than one of them act up at the same time. When I go to one patient, another one acts up and shows behaviours. That is why sometimes we just tie one patient while we go to entertain another.” (Taripnan, caregiver) (Pg 8, Barrier: Inadequate staff numbers and time constraints paragrapgh 2) |
| Finding | Less supportive family members (U) |
| Illustration | "“But some clients are very egoistic … I don’t think they will take the tips and change according to our suggested plan and tips.” (Gaya, caregiver) “But when the children come, they will talk about all the negative points and fight with the elderly. This makes the elderly depressed. Family unity is not there.” (Gaya,caregiver) “She just ate but she forgot, and she tells her daughter that she did not eat. The daughter came and scolded me for not feeding her. I snapped a picture of her eating and ‘WhatsApp’ the daughter.” (Yona, caregiver)(Pg 11 Barrier: Less supportive family members paragraph 1)" |
| Finding | creating meaningful conversations with PWD (U) |
| Illustration | “If they are still aggressive, we just speak gently to them and never oppose whatever they said at that time. If they think that their children will pick them in the evening, we just have to agree with them even if it’s not going to happen, and use that modality to persuade them to go eat or change diaper.” (Venus, caregiver) “We have to slow talk with them while making eye contact and body language. At that moment they will express their feeling, maybe they want something. From there, we are able to identify what is their need.” (Akim, caregiver) (Pg 9 Strategy: Verbal and non-verbal communication paragraph 1) |
| Finding | Elderspeak (U) |
| Illustration | “Yes, we treat them as a baby and so far, none has become angry because we treat them like that. They in fact like it, I don’t know … maybe they like the tone of our voice when we call them.” (Catherine, caregiver) (Pg 10 Elderspeak paragraph 1) “The staff addresses each resident with politeness and respect by using their name and credentials such as Doctor or Professor. The staff speaks to them by normal communication, like an adult-to-adult communication.” (Observational notes) (Pg 11 Elderspeak paragraph 1) |
| Finding | Communication Hindering factors (U) |
| Illustration | “I changed her caregiver to be with the same race. As Indian prefers to be with an Indian to take care of them because they can talk in the same language.” (Gaya, caregiver) “At the start, she speaks English but towards the end, she will start to speak Malay … so obviously I don’t understand her, and I have to remind her to speak English.” (Venus, caregiver) (Pg 10 Barrier: Communication barrier paragraph 1) |
| Finding | Multidisciplinary collaboration (U) |
| Illustration | “Doctors and nurse often come. They will come this evening and check the patients, staff, rooms and sometimes they also check the facilities here.” (Venus, caregiver) “I notify the relatives about the behaviour and how frequent it happened. Then the decision is from the relative or family member, it’s either they want to bring them to a private doctor, in house doctor or the geriatrician to start on medications.” (Yona, caregiver) (Pg 11 Strategy: Multidisciplinary collaboration paragraph 1) |
| **Study:**Tasseron-Dries et al., 2021 | |
| Finding | Feeling like a man in a woman’s world (U) |
| Illustration | “I was the only man, you know. And then, well, it’s different. And then you see how those women interact with the residents. Yes, that is different. Plus, the residents are all different. All different. But, so, on Wednesday there are two male volunteers. That is really good. Makes a change ….” (family caregiver, spouse) (Pg 6 ‘Personal circumstances’: Personal context of family caregivers, paragraph 4) |
| Finding | A clear structure for Namaste (C) |
| Illustration | “Maybe if you do this again, you could say more about what actually happens on these mornings. That’s a possibility. Like: we have a fantastic overhead projector and that will be there, and the music and you are welcome to join in. You could give your father and mother a hand massage, or we can do that. Maybe little more like that" (family caregiver, daughter) (Pg 7 ‘Communication’: Communication between family caregiver, staff and volunteer, Paragraph 2) |
| Finding | Reluctance of family caregiver to take extra obligation (U) |
| Illustration | “Yes, I have taken advantage of it in the sense of: oh, there is singing here this afternoon and she really enjoys that. She’ll be willing to go. And then I can do something else. And I will come in tomorrow”. (Family caregiver, son) (Pg 6 ‘Activities’: Preferences of family caregivers for activities with their relative living with dementia, paragraph 8) |
| Finding | (old) age of the family caregiver, conflicts within the family and caregiver burden (N) |
| Illustration | none |
| Finding | family caregivers did not feel welcome (U) |
| Illustration | “I think it would be prudent to say, of course you can visit, but please remember that the Namaste program is underway and please slow down, relax. Yes, exactly. That it works differently. That you don’t put up barriers in advance, like, well it’s Namaste, so we’d better not visit then.” (family caregiver, daughter) (Pg 7 ‘Communication’: Communication between family caregiver, staff and volunteer, paragraph 3) |
| Finding | Activities they felt comfortable with (U) |
| Illustration | “They also do music [….]. If you don’t like this, then you don’t go. But if there is table shuffeboard [old Dutch board game] or whatever, and you enjoy that, then you will go there. So, I think that that is very personal.” (family caregiver, daughter) (Pg 4 ‘Activities’: Preferences of family caregivers for activities with their relative living with dementia, paragraph 2) |
| Finding | Lack of time (N) |
| Illustration | No illustration |
| Finding | Perceived difficulty to participate (U) |
| Illustration | “You can tell on all sides that it generates feelings of helplessness. People are willing, but they don’t really know how. It’s only few hours. I manage that pretty well now, although it can be sad sometimes, I can more or less accept how far gone she is. But I have seen a group of family members who attended twice, it is so painful every time to see your wife no longer able to do anything. Then you won’t participate in this kind of program.” (family caregiver, daughter) (Pg 6 ‘Personal circumstances’: Personal context of family caregivers, paragraph 3) |
| Finding | Positive response in the resident to an activities (U) |
| Illustration | “I thought it was great that my mother connected with that doll. Because for the first time, I saw some expression on her face again. Her eyes lit up again.” (family caregiver, daughter) “A kind of ‘seeing is believing’. And that makes it really really good.” (activity coordinator) (Pg 4 ‘Activities’: Preferences of family caregivers for activities with their relative living with dementia, paragraph 4) |
| Finding | Living close to nursing home (N) |
| Illustration | No illustration |
| Finding | Lack of skills to undertake activities Pg6 paragraph 3 (N) |
| Illustration | No illustration |
| Finding | The culture of leaving everything to the nursing home (U) |
| Illustration | “Family could do more, but we - as care professionals - should also encourage that. Now we say: you can’t continue like this, you need to go to the nursing home and they can take care of everything there. And then we don’t have to do anything anymore. That is the shift we need to make. We do tend to take over completely and are very much hospitalized in that sense. In this shift the family would also be allowed more and do more if they want to. This is still too far away. First there is this whole other step that needs to be realized” (manager) (Pg 6 ‘Activities’: Preferences of family caregivers for activities with their relative living with dementia, paragraph 7) |
| Finding | Reluctance to engage in unfamiliar activities (C) |
| Illustration | “They (family caregivers) say: “I am not very keen on sitting next to my mother and then giving her that hand massage… It makes people uncomfortable. But if you bring in balloons to shoot those across the table, then they’ll join in without a problem. That’s not physical….”. (manager) (Pg 6 ‘Activities’: Preferences of family caregivers for activities with their relative living with dementia, paragraph 3) |
| Finding | Misconceptions and unclear communication (N) |
| Illustration | No illustration |
| Finding | Lack of knowledge (N) |
| Illustration | None |
| **Study:**Lawrence et al., 2016 | |
| Finding | Lack of recognition from society, managers and relatives (U) |
| Illustration | So, they [the government] really have to recognise that the care workers are doing a highly skilled, professional job, they don’t take it seriously. Even when I am out there and somebody asks me, ‘what are you doing?’ you know a care job and the way people, even the way that the relatives look at you because you are doing this job, you can’t win. And they can’t do it. So really, I feel that they don’t recognise the care job is a good thing, they think we just come here to wash somebody, but that is not what we do. (2004) (Pg 3 table 1 (1a) Lack of recognition) |
| Finding | Time constraints likely to undermine intervention (C) |
| Illustration | There has been so much focus on it recently, dealing with challenging behaviour, creating different activities, etc. Every nursing home in the country would be more than happy to do that, but what people need to realise is that to do that costs a lot more money. To give one to one intervention is very expensive. Whereas it’s dead easy isn’t it if you give them a few tablets? (2002) (Pg 3 table 1 (1b) Lack of resources) |
| Finding | Dislike of word ‘intervention’ (U) |
| Illustration | As soon as you say we are having an intervention, it’s like what you have done wrong needs to be assessed and then we are going to better it through our intervention and we are going to intervene in activities, we are going to intervene in this and this. And to me it’s more of an association with us, working with us to do these things and helping to guide whereas intervention sounds like we have done something wrong. (2004) (Pg 3 table 1 (1a) Lack of recognition) |
| Finding | Lack of resources-pressurized environment (U) |
| Illustration | You feel like you’re not doing your job properly. You actually feel that you’re letting the residents down. Yeah that you’re letting them down. You say, ‘I’ll be with you in a minute, I’ll be back’…and you’re not, you’re running off for something else. (3003) (Pg 3 table 1 (1b) Lack of resources) |
| Finding | Relationships strained with relatives critical of staff (U) |
| Illustration | I think they just don’t understand what dementia is, they try to blame the staff for whatever has happened. I think this is frustrating because when we are trying our best and then somebody will come in and tell you ‘no, no, no’. It’s like we have a lady who goes in her room and messes all her clothes up and her daughter comes in and then they think she thinks it’s the care staff that have to put it away properly, when it isn’t. (1002) (Pg 3 table 1, (2b) Relationships with relatives) |
| Finding | Fear of criticism from training team (U) |
| Illustration | I’m a little bit concerned now because obviously somebody’s going to be coming in and you know again it goes back to the active living team when we had them in and they, the way that they spoke to us and the way it was sort of didn’t they, we weren’t good enough and they were sort of telling us how to do our job and that’s what I’m a little bit concerned about that someone’s going to come in and say do this do that and we think ‘hang on’. (1004) (Pg 3 table 1 (1a) Lack of recognition) |
| Finding | Concern about engaging all staff in intervention (U) |
| Illustration | What will happen is they will talk, smile and pretend to understand and then after it will be a different thing. Some of them have the attitude, ‘It’s not my job, I am just here to clean him, feed him, that’s it, I don’t need to do anything else, it’s not my job’. (2002) (Pg 4 table 1, (2c) Relationships within the team) |
| Finding | Mutual respect and reciprocity key to good care (U) |
| Illustration | People bring their dogs in, bring their family in, pictures, we all send postcards if we go on holiday…it’s a lot like life as a general is all shared.(1001) And they like a laugh, a joke, rather than treating them like, you treat them like you treat your Nan… because obviously they were born and bred in Dagenham, so they talk like we do…there’s no need for airs and graces or anything like that. (3004) (Pg 3 table 1, (2b) Relationships with relatives) |
| Finding | Collective responsibility enables staff to meet resident needs (U) |
| Illustration | We all work as a cog in a wheel and if one of those cogs breaks then the wheel doesn’t turn does it? So what we do is we all work together it’s like they work upstairs with the carers and if something is wrong they report here and then it gets reported to the doctor…That is the heart of the person centred care because if we don’t have that we won’t know the person’s needs. It won’t be met without us knowing. (3001) (Pg 4 table 1, (2c) Relationships within the team) |
| Finding | Divisions between staff groups (U) |
| Illustration | What, hang on, what if you’ve got staff which you get in every home with a really negative attitude or say are just very negative, ‘oh no that’s not going to work’. Yeah and very narrow minded and it’s going to be a case of trying, as well as trying to implement this with the residents, it’s going to be difficult trying to get the staff to act on this as well. (1004) (Pg 4 table 1, (2c) Relationships within the team) |
| **Study:**Pieper et al., 2018 | |
| Finding | Staff turnover, shortage of staff and high workload(Pg 7, Facilitating and impeding factors associated with the level of the organisation, paragraph 2) (N) |
| Illustration | None |
| Finding | Interdisciplinary learning and cooperation (U) |
| Illustration | Nurse assistant: “What I really liked was the fact that we were participating in this training as a whole multidisciplinary team including all related disciplines, not only as a single nursing team. For example, a psychologist looks at problems in a different way, i.e. from another point of view. I thought: I’ve never really seen it that way – but I guess you’re right.” ……… “I think it contributed to the fact that the barriers for contacting the other disciplines have become smaller, they’re more easily accessible now.” (Pg 8 Facilitating and impeding factors associated with the level of the team, paragraph 2) Psychologist: “The nursing staff has a lot of fun in finding out why someone behaves in a certain way. Now, they ask me at an earlier stage how to deal with challenging behaviours, and as such I can do my job better, more targeted, and with more members of the team.” (Pg 8 Facilitating and impeding factors associated with the level of the team, paragraph 2) |
| Finding | Seeing results motivated them to utilise the intervention (U) |
| Illustration | Nurse: “Well, the moment of getting her out of bed was always… how shall I say…. Well, most of the time we thought: we’ll help her after our coffee break, around 11 o’clock–11.30. But then I noticed, when we helped her to get out of bed, say, around 8 o’clock–8.30, that she came singing out of bed, went to breakfast, and was quite relaxed.” (Pg 9 Facilitating and impeding factors associated with the level of the individual resident/professional, paragraph 2) Nurse: “It’s actually easier now to try out pain medication. Elderly care physicians were often reluctant – but with this stepwise intervention we have more evidence to support our request for treatment.” (Pg 9 Facilitating and impeding factors associated with the level of the individual resident/professional, paragraph 2) |
| Finding | Presence of a person with a motivational leadership style (Pg 7 Facilitating and impeding factors associated with the level of the team, paragraph 5) (N) |
| Illustration | None |
| Finding | Organisational changes or other innovations at the time of the implementation (Pg 7, Facilitating and impeding factors associated with the level of the organisation, paragraph 1) (N) |
| Illustration | None |
| Finding | Having different schedule hindered interdisciplinary cooperation (U) |
| Illustration | Registered nurse: “The hardest thing was working together on-the job in subgroups, which consisted of different disciplines. Since we all had different schedules and days off, but at the same time had to assess the steps in groups, someone took the lead and then others took over if we had only a short time together to fill-out the forms. That’s how we solved it.” (Pg 8 Facilitating and impeding factors associated with the level of the team, paragraph 5) |
| **Study:**Ducak et al., 2018 | |
| Finding | support from management (U) |
| Illustration | ... making sure from top-down in the long-term care facility that there’s administrative buy-in that the management staff, the director of care buys into this too, because ... it’s a bottom-up training so it’s at the field level, anybody can use it, we just need the top-down to support it. consultants (C5) (Pg 18, enabling factors, paragraph 12) ... any given day there could be so many emergencies and so many crisis situations because ...they don’t have [adequate] resources...this Montessori for example, goes under unless there is a manager who is very strong and who can very determinedly take the agenda forward. consultant (C2) (Pg 18, enabling factors, paragraph 13) ...sometimes it’s just an ear when I want to vent and say, ‘‘What do you think about this?’’ And sometimes it’s validation, like they’ve been certified and can go, ‘‘No let’s go back to the theory and practice.’’ You know, to get me back on the right track. consultant (C3) ) (Pg 18, enabling factors, paragraph 14) |
| Finding | Lack of funding (U) |
| Illustration | ‘It’s always an issue ... We’re trying to make the best out of what we have’. participant (R1) (Pg 13 limiting factors, paragraph 9) ‘I think some of our barriers here are more financial barriers because I think it would be beneficial to send more staff to the workshop but financially that’s not feasible’ participant (R4) (Pg 13 limiting factors, paragraph 11) ‘... we have six people in our department and they couldn’t afford to send every one of us’. recreation assistant (R5) (Pg 13 limiting factors, paragraph 11) |
| Finding | unwillingness of nursing staff to use MMD (U) |
| Illustration | C4: I think sometimes it’s better to have an outside source come in and promote it. And then they see that it’s something that the resident needs rather than something that the activity department is responsible for. ... a lot of times it’s easier to say, ‘‘Oh, that’s her job, not mine.’’ But if I come from the outside, I’m hoping that the nursing staff will do it more. ... And the nursing staff that I’ve talked to have been very supportive about it. They really liked it. (Pg 13 limiting factors, paragraph 7) |
| Finding | Knowledge of usefulness (C) |
| Illustration | ... you can either spend the time responding to those behaviours OR you can implement the little two second activity and let them work on it for half an hour... Again they need that theory and the training and repetitive reminders as to it really isn’t time consuming – where do you want to spend YOUR time? consultant (C3) (Pg 17, enabling factors, paragraph 3) |
| Finding | medicalized nature of LTC and adherence to routines (U) |
| Illustration | ‘behaviours are presented but they’re looked at as behaviours that need to be treated rather than looking at what causes these behaviours’. consultant (C2) ) (Pg 13 limiting factors, paragraph 4) |
| Finding | limited time available for recreation staff to get resident onboard (U) |
| Illustration | ‘it’s hard because we only have three programs in there a day so it’s... nursing staff and PSWs [Personal Support Workers] that are there with them all the time but we’re having a hard time getting them on board.’ participant (R6) (Pg 13 limiting factors, paragraph 6) |
| Finding | lack of staff buy-in (U) |
| Illustration | Staff buy-in is another problem where I go in and I say, ‘‘I would like you to try this.’’ And they’re like, ‘‘Well, I have to get eleven people out of bed. I’m not doing that for him.’’ It’s a very task-oriented work environment and so to add something that is sort of unique or a little bit out of the box is threatening to some of the staff, especially those who have been there forever... So staff attitudes is a big deal. consultant (C5) (Pg 13 limiting factors, paragraph 3) ... everybody can do this, it’s not just programming, PSWs can do these activities, the registered nurse when she’s going by with the med cart can ... it’s getting everybody’s buy-in that they’re all responsible. They really still think it’s just programs’ and recreation’s job. (Pg 13 limiting factors, paragraph 6) |
| Finding | Fear of disapproval from ministry (U) |
| Illustration | Some of the barriers [are], you know, nursing being task-oriented and thinking that the Ministry is not going to allow for this. Everybody’s nervous, you know, and I’ll just say in our area ...residents... like to do household chores. So that would take over some of our staff’s chores if they allowed them to, but they’re so nervous of the Ministry. ...Like if they make the bed and not do it correctly. I know that they’re driven by the Ministry of Health, and so are we, but if [residents] don’t make the bed properly they figure if an inspector comes in and trying to explain that would just be way harder than just doing it themselves. recreation manager (R11) (Pg 13 limiting factors, paragraph 2) |
| Finding | Lack of interdisciplinary collaboration in LTC (U) |
| Illustration | My heart goes out to the recreation staff because it seems they really just lack credibility with the nursing staff ...They put them lower down on the totem pole, unfortunately. ...I think the rec staff know what they do but I don’t know that in their training they have that extra level of understanding the medical side of things, so sometimes that’s where the downfall is... And so over my years when I’ve been consulting to the recreation department, it’s even simply changing their language or understanding a diagnosis more because if you ask an RN [Registered Nurse] to explain what’s going on medically they’re happy to share it, but I think the rec staff get scared ...the nurses... can talk down to them, so I understand their fear too. consultant (C5) ) (Pg 13 limiting factors, paragraph 5) |
| Finding | Rigid routine (U) |
| Illustration | ‘there’s such a routine, a rigid routine, like meals are this time and bath is this time and, personal care is this time’ (R2) (Pg 13 limiting factors, paragraph 1) |
| Finding | Difficulty to alter a deeply ingrained medical nature of LTC (U) |
| Illustration | We’re just dealing with a primarily medical environment, right? And, while even the government’s trying to get away from that it’s hard when you’re dealing with people who have been in nursing for 20 years and suddenly be open to this whole other way of nursing. recreation manager (R12) (Pg 13 limiting factors, paragraph 4) |
| Finding | very low staff-to-resident ratio (U) |
| Illustration | ...the one-to-one sort of really intense programming, I would say, about 10 percent. Because it comes down to those ratios and the time, however, we do try to apply it to all the programs, well OK, say 80 percent of the programming we provide. recreation manager (R12) (Pg 13 limiting factors, paragraph 9) ... our dementia unit is quite active right at the moment and at this point it’s attention span and staffing levels. Because there’s only one activation person to 28 [residents] so ... if she’s not monitoring for agitation or aggressive people then the person’s able to utilize the Montessori but it, sometimes their role has to be different than programming. recreation manager (R7) (Pg 13 limiting factors, paragraph 10) |
| Finding | Support from family (U) |
| Illustration | ... we do run into who pays for it. In this case though, the families have never had a problem with that because really it’s never very expensive, it’s usually less than 20 dollars, and they, the families, love the individualized attention. consultant (C4) (Pg 18, enabling factors, paragraph 15) |
| Finding | Seeing Results is Believing (U) |
| Illustration | there and seeing it work and seeing the residents get engaged and smiling and taking part in an activity, especially residents when they don’t think that they’re capable really of doing much of anything ... it’s a bit of a shocker when they hear a resident being the first to call out the answer to something when they thought all they could do was repeat the same sentence or phrase over and over again and actually to see that those memories are there ... we just have to know how to get them to surface. consultant (C1) (Pg 18, enabling factors, paragraph 7) The reading program I would have to say is the biggest win that we’ve had not only for the resident themselves but to actually promote our department, like activation with nursing staff and families, and it’s like, ‘‘Ah, she reads! Oh my!’’ ... it really is truly amazing to listen to someone who may not have put two words together in such a long time and then be able to actually concentrate and read those words. recreation manager (R7) (Pg 18, enabling factors, paragraph 8) ...there’s always people that are kind of set in their ways and the naysayers... in particular the doll therapy where they thought it was infantilizing somebody to give them a doll, but again, when they see how they react to the doll and that it brings them joy ...they’re more likely to come on board with it. consultant (C1) (Pg 18, enabling factors, paragraph 9) |
| Finding | Lack of understanding (C) |
| Illustration | R4: ...some of the things that we’ve encountered is just the knowledge base of the staff ... we focus on different areas and so some people don’t understand when we’re trying to allow them to be successful and trying to focus on the positive. We’ve hit some resistance with some of the staff that they don’t understand why we just don’t do it for them. {chuckles} (Pg 16-17, enabling factors, paragraph 1 ) ... when I brought home all of the resource materials from the course I sat down with my staff and we ... went through each one as to how it would benefit or how they would explain it to other staff if they said, well, you know, ‘‘Why are you scooping golf balls into a muffin tin?’’ So we rationalized ... ‘‘OK, that motion will maintain dexterity so that they can continue to feed themselves.’’ So that makes sense to a nurse, to a PSW, so because there’s always that thought, ‘‘Well, this is childish or this is not appropriate.’’ Or that type of thing. (Pg 17, enabling factors, paragraph 2) |
| **Study:**Forget et al., 2021 | |
| Finding | Positive feedback from families and other caregivers (U) |
| Illustration | "At the team level, there is enthusiasm, they ask when the dog is going to come back"; "the families are pleased, they see that their relatives can still speak on a common subject". (PSY04, AMP05) (Pg 5 feedback paragraph 1) |
| Finding | Increased cognitive stimulation (U) |
| Illustration | "Silent people can start to speak, to express their feelings in the presence of the animal"; "that provides a point of reference, it is necessary to remember the day of the week when the animal is there"; "the animal revives memories" (ANIM10, ASG11) (Pg 5 cognitive stimulation paragraph 1) |
| Finding | Management constraint (U) |
| Illustration | "/close-minded management"; "I put pressure on the management", "reluctance of the management in terms of hygiene"; "we need to convince them" (ANIM03, PSY07, ANIM06) (P3 management, paragraph 1) |
| Finding | Budget constraint (U) |
| Illustration | "the first thing to be asked will be: How much does it cost?! "; "Expensive training and far away, which generates costs "; " the cost was much too high for this to be done "; " we did not have the budget "; " if we cut the budget aspect, because "; "the effects would be in the long term, it is difficult when everything is finance-led"(ANIM06, PSY04, AMP05, ANIM09, ANIM06) (P3 budget, paragraph 1) |
| Finding | Organizational constraints (U) |
| Illustration | "Difficulties in finding a person to be in charge of the dog because of the responsibilities involved"; "due to training in Alsace (remote location)"; "It is a question of time"; " there is always at least one person from the structure with ‘her’"; " we were told: a dog is not possible, because it needs surveillance"; “it needs to be taken out all the time "; " after each session we analyze what happened "; "little inconvenience except the logistics (feeding, nighttime, care)” (PSY04, ANIM03, PSY08, AMP05) (P3 Organizational constraints, paragraph 1) |
| Finding | Animal phobia (U) |
| Illustration | "The downside is for families who are afraid of dogs when they see one, but it’s quite rare,"; “there are people who don’t like dogs, or even fear them. I have seen fear and apprehension in people who have never had animals.” (PSY02, ANIM06) (P3 animal phobia, paragraph 1) |
| Finding | Physical stimulation (U) |
| Illustration | "Residents agree to walk in ‘her’ presence,"; "they stroke ‘her’, brush…"; "they reach out to the animal to touch ‘him’, pet ‘him’ as he goes by" (PSY02, ANIM03, ANIM10) (Pg 5 Physical stimulation paragraph 1) |
| Finding | Concern about animal hygiene (U) |
| Illustration | "There could be a concern about hygiene, dog hair, if ‘he’ licks things"; "families could say that it was not very hygienic,"; "the downside is in terms of cleaning"; "our management isn’t very animal-friendly in our structure, it’s a matter of hygiene." (ASG01, PSY02, ANIM03, PSY04) (P3 hygiene, paragraph 1) |
| Finding | Patient motivation (U) |
| Illustration | “As soon as people arrive”;” motivation to try to make gestures”;” a gentleman who never wants to leave his room comes to walk the dog”;” Ms. X accepts to be washed in the presence of the dog”; “it’s a new activity, which is not like the ones usually offered, and it affects other people” (ANIM03, ANIM09, PSY02 PSY08) (Pg 5 patient motivation paragraph 1) |
| Finding | Intervention effectiveness (U) |
| Illustration | "We targeted people with dementia or motor disorders"; "I remember a gentleman who did not leave his room and he agreed to go for walks with the dog."; " Elderly people with cognitive disorders or anxious people ";" end of life support ". (PSY08, ANIM09, ANIM10) (P3 Affected audience, paragraph 1) |
| Finding | Work overload (U) |
| Illustration | "Some have complained about fear of an additional workload"; "it would take time for that"; "the disadvantage is in terms of cleaning "; "no additional time is allocated to caregivers for activities”. (PSY08, PSY04, PSY02, ANIM09) (P3 workload, paragraph 1) |
| Finding | Improved social interaction (U) |
| Illustration | l. "They talk to each other about the dog, if they had ever had one"; "People show completely different behavior when the dogs are there. There are people who, basically, are completely withdrawn, sleeping, in their armchairs, completely curled up. The dogs arrive and that’s it they stand up, open their eyes, open up, look around themselves to see what’s going on, it’s different ";" we make contact with the person ";" people can start to speak. (ASG0, ANIM06, ANIM10) (Pg 5 social interaction paragraph 1) |
| Finding | Concern about animal’s quality of life (U) |
| Illustration | "The disadvantage is that XXX is an animal and so it is a sensitive living being, it will feel tension and be afraid of certain residents"; "we can’t force it"; "the dog is exhausted at the end of the day because it is called upon a lot”. (PSY 02, AMP05, PSY07) (P3 Animal’s quality of life paragraph 1) |
| Finding | Improved quality of life of the elderly (U) |
| Illustration | "The presence of XXX has improved the life quality of residents, it brings joy, spontaneity, affection, companionship, the impression that it understands them and that they can confide in it"; "There are little lights in their eyes … it relaxes them, there are smiles, laughs, the pleasure of seeing the dogs "; "The residents take them in their arms, caress them"; " anxiety is reduced ". (PSY02, ANIM03, ANIM06) (P3 quality of life paragraph 1) |
| **Study:**Webster et al., 2022 | |
| Finding | understaffing at night-time (U) |
| Illustration | I think it’s just loneliness because sometimes we’ll sit with her, and we can chat for hours. Sometimes we don’t have the time. (15; female care assistant) Although we have some nights that we’re short. . . Here in this floor, it should be four staff working. One is one to one, two staff on the floor and then the nurse. So, if one has cancelled their shift so the nurse will be working on the floor or sometimes sitting as one to one and then the other two staff will be ensuring the safety of other residents. (7; female nurse) (Pg 8 Barriers to managing sleep disturbances, paragraph 4) |
| Finding | difficult to encourage and keep people awake in the daytime (U) |
| Illustration | We try to keep him awake but it’s no chance. You can’t. He just sit[s] down and he’s too tired and he close his eyes. (1; female care assistant) Earlier on this month there was a time he was so tired he would just eat a piece of his breakfast and go to bed and sleep. We try as much to keep him busy during the day but he’s quite a strong headed person. (3; female team leader) (Pg 5 Evening strategies to promote sleep, paragraph 1) |
| Finding | difficulty during the night to encourage residents to go back to their rooms (U) |
| Illustration | If he doesn’t want to go to his room, we just have to leave him because we just don’t want him to kick you or hit you. So, whenever he’s sleeping in the chair, and you try maybe, “go to your room”, one, two, three [times]. He says, “no.” (5; female care assistant) Because she couldn’t really express every time that this is what I want. . . So, they don’t know what causes the agitation at night. (7; female nurse) (Pg 8 Barriers to managing sleep disturbances, paragraph 2) |
| Finding | residents’ family’s complaint to staff (U) |
| Illustration | Because the son is always complaining, “my mum is not comfortable. She has to go to bed.” So sometimes exactly after lunch, he’ll want his mum to go to bed. (5; female care assistant). So, the nurse told us that we should reduce the wine in the daytime. Because it’s making him sleep less in the night. But when his children comes [to visit], her daughter, when she comes, she brings some from the house. (16; female care assistant) (Pg 8 Barriers to managing sleep disturbances, paragraph 5) |
| **Study:**Cohen-Mansfield & Meschiany, 2022 | |
| Finding | Lack of regular, professional and individualized activities (U) |
| Illustration | “The main goal is to [keep the residents] interested. When there is an activity, it is better for the staff; they have no time for nonsense. There is no solution for about one-third of the residents who do not attend any activity” (#29, occupational therapist). (Pg 4 Concerns related to residents, paragraph 8) “No one comes to residents who do not cooperate or are sleepy. There are no afternoon activities” (#1, head nurse). (Pg 4 Concerns related to residents, paragraph 8) When staff were asked what changes, they would like to see implemented, they referred not only to the need for increased budget and workforce (#29, occupational therapist), but to activities for residents: “Residents need to [be occupied], many are sitting idle. There is no one to approach residents who do not cooperate” (#1, head nurse). (Pg 5 Institutional barriers, paragraph 1) |
| Finding | Rigid care routine (U) |
| Illustration | For example, some residents were given sleeping pills to adjust their schedule to the majority: “[...] There is an hour when the majority goes to sleep with some given sleeping pills [only] to [to be] awoken at early hours in the morning” (#14, CEO). (Pg 4 Concerns related to residents, paragraph 5) |
| Finding | Concerns related to residents -Behavioral challenges and violence (U) |
| Illustration | “Violent, full of violence, [we are] assaulted endlessly. Nothing [you can] do” (#2, nurse), (Pg 3 Concerns related to residents, paragraph 1) “Sometimes [we are subject to] residents’ lack of cooperation, restlessness, shouting” (#14, CEO) (Pg 3 Concerns related to residents, paragraph 1) “There is a resident whom one is not to touch; if I move him, he might give me a punch” (#18, activity worker) (Pg 3 Concerns related to residents, paragraph 1) “issues of communication and violence are really important” (#29, occupational therapist). (Pg 3 Concerns related to residents, paragraph 1) |
| Finding | Lack of communication skills among staff (U) |
| Illustration | “How to achieve communication [is a need]. Are there any tools that can be given to therapists? Warmth, smiles? Most employees do not have the skills to do so” (#21, CEO). (Pg 4 Concerns related to other staff, paragraph 1) “most of the staff are foreign workers ... language and turnover [are issues]” (#14). (Pg 4 Concerns related to other staff, paragraph 1) |
| Finding | Lack of financial resources (U) |
| Illustration | When asked what they would like to change, staff consistently responded “salary” (e.g., #22, social worker), “[I am] satisfied with everything except the salary” (#12, nursing assistant). (Pg 5 Institutional barriers, paragraph 1) “There is a salary problem. The Ministry of Health is willing to give [only] a minimum [wage]... The issue of wages is very difficult and therefore the whole issue of manpower” (#27, CEO). (Pg 5 Institutional barriers, paragraph 1) |
| Finding | Lack of equipment and adequate facilities (U) |
| Illustration | “Bad bathing chairs, which need to be moved from room to room” (#2, nurse). (Pg 5 Lack of equipment and adequate facilities, paragraph 1) “There is a need for a garden, a balcony, more activities” (#1, head nurse). “Nobody has hearing aids, an issue that is not addressed. Seventy percent do not hear well” (#15, occupational therapist). (Pg 5 Lack of equipment and adequate facilities, paragraph 1) |
| Finding | Lack of manpower (U) |
| Illustration | “Over the last five years, there has been a decline in the workforce, less [quality], employees work in three jobs. This is a problematic situation” (#21, CEO) (Pg 5 Institutional barriers, paragraph 2) : “Work practices are dictated by a lack of manpower ... a method of dividing work, each nurse has his or her own residents. You need to double the manpower...” (#24, head nurse). (Pg 5 Institutional barriers, paragraph 2) “There are almost no permanent employees, high degree of burnout, many employees have to work in additional places” (#14, CEO). (Pg 5 Institutional barriers, paragraph 2) |
| Finding | Lack of training (U) |
| Illustration | “Training the caregiver team it is naive to think that will happen. In the afternoon there is a heavy silence when there is no activity. They don't get enough compensation, so asking [staff] to do anything extra [e.g., undergoing training, conducting activities], is a joke” (#3, activity worker). (Pg 5 Institutional barriers, paragraph 4) |
| **Study:**Miller et al., 2021 | |
| Finding | activity supplies disappearing (Pg 4 Group interview, paragraph 2) (N) |
| Illustration | No illustration |
| Finding | Lack of time to implement aromatherapy (Pg 4 Group interview, paragraph 2) (N) |
| Illustration | No illustration |
| Finding | designating a single staff member responsible for filling and turning on the diffuser each day (Pg 4 Group interview, paragraph 2) (N) |
| Illustration | No illustration |
| Finding | one-on-one time requirements from staff to implement natural light ((Pg 4 Group interview, paragraph 2)) (N) |
| Illustration | No illustration |
| Finding | championing by activity director (Pg 4 Group interview, paragraph 2) (N) |
| Illustration | No illustration |
| Finding | unfavourable weather conditions to use natural light outside (U) |
| Illustration | "Multiple sites reported using natural light indoors by opening window blinds and one staff stated “[resident’s] mood is better when it’s bright and sunny outside.” (Pg 4 Natural light, paragraph 1)" |

U=Unequivocal; C=Credible; N= not supported by illustration

# Appendix 6. TDF categorised factors influencing the implementation of NPIs for the management of BPSD.

| **TDF Category** | | **Category** | **Finding** | **Citations** |
| --- | --- | --- | --- | --- |
| **1. Knowledge (an awareness of the existence of something)** | |  |  |  |
|  | | Education and training | Inadequate staff education as a limitation | Ervin et al., 2014 |
|  | |  | Lack of education | Kolanowski et al., 2010 |
|  | |  | Staff's lack of education | Kong & Kim, 2022 |
|  | |  | Family's lack of education | Kong & Kim, 2022 |
|  | |  | Lack of Training | Hussin et al., 2021 |
|  | |  | Lack of education and training | Clifford & Doody, 2018 |
|  | |  | Lack of training (Hindering factor) | Cohen-Mansfield & Meschiany, 2022 |
|  | |  | Need to educate initial staff and new staff | Kwak et al., 2021 |
|  | |  | Training and support for M&M | Kwak et al., 2021 |
|  | |  | Continuing education | Clifford & Doody, 2018 |
|  | | Familiarity with the resident | Lack of accurate information about individual preferences | Garrido et al., 2021 |
|  | |  | Difficulty of connecting with and knowing residents | Nunez et al., 2018 |
|  | |  | The inability of staff to meet the resident where they are at the moment | Kolanowski et al., 2010 |
|  | |  | Lack of understanding among newly hired employees | Kolanowski et al., 2010 |
|  | |  | Not knowing residents | Kolanowski et al., 2010 |
|  | |  | Knowing residents past occupation and their interests | Kolanowski et al., 2010 |
|  | |  | Knowing residents timing and the concept of time | Kolanowski et al., 2010 |
|  | |  | Familiarity with the resident | Janzen et al., 2013 |
|  | |  | Attitudes and being self -aware when caring | Clifford & Doody, 2018 |
|  | |  | Getting to know the person | Clifford & Doody, 2018 |
|  | | Familiarity with the intervention | Mappers’ lack of understanding the DCM | Griffiths et al., 2019 |
|  | |  | Complex nature of DCM™ | Griffiths et al., 2019 |
|  | |  | Lack of knowledge | Tasseron-Dries et al., 2021 |
|  | |  | Little awareness of psychologist support | McKenna et al., 2022 |
|  | |  | Lack of understanding | Ducak et al., 2018 |
|  | |  | Lack of knowledge about the effectiveness of NPIs | Kolanowski et al., 2010 |
|  | |  | Use of technology | Kwak et al., 2021 |
|  | |  | Staff familiarity with music | Gulliver et al., 2021 |
|  | |  | Being newer staff willingness to learn | McKenna et al., 2022 |
|  | |  | Knowledge of usefulness | Ducak et al., 2018 |
|  | |  | A clear structure for Namaste | Tasseron-Dries et al., 2021 |
| **2.Environmental context and resources (Any circumstance of a person's situation or environment that discourages or encourages the development of skills and abilities, independence, social competence and adaptive behaviour)** | |  |  |  |
|  | | Competing priorities | Competing priorities | Griffiths et al., 2019 |
|  | |  | Time gap between training and undertaking DCM implementation | Griffiths et al., 2019 |
|  | |  | Tendency for staff to have background music on | Garrido et al., 2021 |
|  | | Organisational environment | Lack of dementia specific unit | Ervin et al., 2014 |
|  | |  | Challenge with safe storage of iPod or headphones | Garrido et al., 2021 |
|  | |  | Unfavourable weather conditions to use natural light outside | Miller et al., 2021 |
|  | |  | Difficulty to alter a deeply ingrained medical nature of LTC | Ducak et al., 2018 |
|  | |  | The changing landscape | Kolanowski et al., 2010 |
|  | |  |  |  |
|  | |  | Inappropriate physical environment | Kong & Kim, 2022 |
|  | |  | Living close to nursing home enhanced volunteer support | Tasseron-Dries et al., 2021 |
|  | | Funding | Budget constraint | Forget et al., 2021 |
|  | |  | Lack of funding | Clifford & Doody, 2018 |
|  | |  | Lack of resources-pressurized environment | Lawrence et al., 2016 |
|  | |  | Activity supplies disappearing | Miller et al., 2021 |
|  | |  | Lack of funding (Hindering factor) | Ducak et al., 2018 |
|  | |  | Lack of regular, professional and individualized activities | Cohen-Mansfield & Meschiany, 2022 |
|  | |  | Lack of financial resources | Cohen-Mansfield & Meschiany, 2022 |
|  | |  | Lack of equipment and adequate facilities | Cohen-Mansfield & Meschiany, 2022 |
|  | |  | Lack of funding (Hindering factor) | Kaasalainen et al., 2019 |
|  | |  | Costs for buying music | Kwak et al., 2021 |
|  | |  | Accessibility of equipment | Kwak et al., 2021 |
|  | |  | Funding | Kwak et al., 2021 |
|  | | Organisational support | Lack of RACH manager support | Griffiths et al., 2019 |
|  | |  | The hierarchical nature of RACHs sometimes acted as a hindering factor | Griffiths et al., 2019 |
|  | |  | Organizational constraints | Forget et al., 2021 |
|  | |  | Management constraint | Forget et al., 2021 |
|  | |  | Lack of management support | Clifford & Doody, 2018 |
|  | |  | Organisational changes or other innovations at the time of the implementation | Pieper et al., 2018 |
|  |  | | Inequality in the allocation of activity provision | Backhouse et al., 2016 |
|  |  | | Lack of manager support by scapegoating | Clifford & Doody, 2018 |
|  |  | | RACH manager support | Griffiths et al., 2019 |
|  |  | | Support from management | Ducak et al., 2018 |
|  |  | | Strong support from administration | Kaasalainen et al., 2019 |
|  | Rigid routine | | Rigid routine | Ducak et al., 2018 |
|  |  | | Medicalized nature of LTC and adherence to routines | Ducak et al., 2018 |
|  |  | | Rigid care routine | Cohen-Mansfield & Meschiany, 2022 |
|  | Time constraints | | Staff time constraints | Griffiths et al., 2019 |
|  |  | | Mappers’ time constraints | Griffiths et al., 2019 |
|  |  | | Time constraints | Ervin et al., 2014 |
|  |  | | Lack of time | Garrido et al., 2021 |
|  |  | | Lack of time (Hindering factor) | Tasseron-Dries et al., 2021 |
|  |  | | Time constraints likely to undermine intervention | Lawrence et al., 2016 |
|  |  | | Lack of time to implement aromatherapy | Miller et al., 2021 |
|  |  | | Limited time available for recreation staff to get resident onboard | Ducak et al., 2018 |
|  |  | | Staff burden over time | Kaasalainen et al., 2019 |
|  |  | | Lack of time | Kwak et al., 2021 |
|  |  | | Insufficient time | Gulliver et al., 2021 |
|  |  | | lack of time | Kong & Kim, 2022 |
|  | RACH staffing | | Staffing related issues (lack of nurses and other staff, staff turnover, mappers attrition) | Griffiths et al., 2019 |
|  |  | | Work overload | Forget et al., 2021 |
|  |  | | Insufficient staffing levels | Nunez et al., 2018 |
|  |  | | Inadequate staff numbers and time constraints | Hussin et al., 2021 |
|  |  | | Shortage of staff | Clifford & Doody, 2018 |
|  |  | | Understaffing at night-time | Webster et al., 2022 |
|  |  | | One-on-one time requirements from staff to implement natural light | Miller et al., 2021 |
|  |  | | Staff turnover, shortage of staff and high workload | Pieper et al., 2018 |
|  |  | | Very low staff-to-resident ratio | Ducak et al., 2018 |
|  |  | | Lack of manpower | Cohen-Mansfield & Meschiany, 2022 |
|  |  | | Understaffing | Kaasalainen et al., 2019 |
|  |  | | Inadequate staffing | Kwak et al., 2021 |
|  |  | | Time constraints and low staff-to resident ratios | Janzen et al., 2013 |
|  |  | | Workload with engagement of staff in routine activities | Chaudhry et al., 2020 |
|  |  | | Insufficient staff | Kong & Kim, 2022 |
|  | Dementia resident reluctance to engage in activities | | Reluctance to engage in unfamiliar activities | Tasseron-Dries et al., 2021 |
|  |  | | difficult to encourage and keep people awake in the daytime | Webster et al., 2022 |
|  |  | | difficulty during the night to encourage residents to go back to their rooms | Webster et al., 2022 |
|  |  | | Residents’ reluctance to take part in activities | Backhouse et al., 2016 |
|  | Dementia resident’s level of impairment | | physical or mental impairment | Backhouse et al., 2016 |
|  |  | | DCM™ did not suit some residents | Griffiths et al., 2019 |
|  |  | | Resident’s refusal to comply or participate | Ervin et al., 2014 |
|  |  | | rise in resident acuity level or impairments | Kolanowski et al., 2010 |
|  |  | | Residents' inability to recognise one's level of impairments | Kolanowski et al., 2010 |
|  | Dementia resident's behaviour | | Frequency and severity of resident's behaviour | Clifford & Doody, 2018 |
|  |  | | fluctuations in mood, behavioural problems, verbal outbursts of residents | Chaudhry et al., 2020 |
|  |  | | residents crossed boundaries | Van Der Ploeg et al., 2012 |
|  |  | | witnessing agitation as a hindering factor | Van Der Ploeg et al., 2012 |
|  |  | | Aggressiveness towards cargiver | Kolanowski et al., 2010 |
| **3. Social influences** |  | |  |  |
|  | Collaboration | | Staff reluctance to support the mapper | Griffiths et al., 2019 |
|  |  | | difficulty to engage the staff team | Griffiths et al., 2019 |
|  |  | | Night and day care staff working relationship | Nunez et al., 2018 |
|  |  | | Less supportive family members | Hussin et al., 2021 |
|  |  | | (old) age of the family caregiver, conflicts within the family and caregiver burden | Tasseron-Dries et al., 2021 |
|  |  | | absence of joint working | McKenna et al., 2022 |
|  |  | | Relationships strained with relatives critical of staff | Lawrence et al., 2016 |
|  |  | | Divisions between staff groups | Lawrence et al., 2016 |
|  |  | | residents’ family’s complaint to staff | Webster et al., 2022 |
|  |  | | Having different schedule hindered interdisciplinary cooperation | Pieper et al., 2018 |
|  |  | | Lack of interdisciplinary collaboration in LTC | Ducak et al., 2018 |
|  |  | | lack or inconsistency of volunteers and family support | Kwak et al., 2021 |
|  |  | | Lack of trust between staff and families | Kong & Kim, 2022 |
|  |  | | Conflicts among/between staff and families | Kong & Kim, 2022 |
|  |  | | bonding between residents and volunteers as positives | Van Der Ploeg et al., 2012 |
|  |  | | whole home’ engagement approach | Griffiths et al., 2019 |
|  |  | | Collective reflection on DCM™ feedback | Griffiths et al., 2019 |
|  |  | | Multidisciplinary collaboration | Hussin et al., 2021 |
|  |  | | Collaboration between nurses and activities coordinator | Clifford & Doody, 2018 |
|  |  | | Team working | McKenna et al., 2022 |
|  |  | | Mutual respect and reciprocity key to good care | Lawrence et al., 2016 |
|  |  | | Interdisciplinary learning and cooperation | Pieper et al., 2018 |
|  |  | | support of facility personnel | Kwak et al., 2021 |
|  |  | | family and volunteers’ involvement | Kwak et al., 2021 |
|  |  | | Collective responsibility enables staff to meet resident needs | Lawrence et al., 2016 |
|  |  | | Support from family | Ducak et al., 2018 |
|  | Communication | | Communication as a challenge | Nunez et al., 2018 |
|  |  | | Communication hindering factor | Hussin et al., 2021 |
|  |  | | Feeling like a man in a woman’s world | Tasseron-Dries et al., 2021 |
|  |  | | Misconceptions and unclear communication | Tasseron-Dries et al., 2021 |
|  |  | | Lack of communication among staff, residents and families | Kong & Kim, 2022 |
|  |  | | managing a range of personalities in group activities | Van Der Ploeg et al., 2012 |
|  |  | | interacting with older people was enjoyable as perceived by volunteers | Van Der Ploeg et al., 2012 |
|  |  | | Elderspeak | Hussin et al., 2021 |
|  |  | | Improved social interaction | Forget et al., 2021 |
|  |  | | creating meaningful conversations with PWD | Hussin et al., 2021 |
| **4. Skills** |  | |  |  |
|  | Lack of experience | | Lack of experience | Griffiths et al., 2019 |
|  |  | | Lack of experience | Ervin et al., 2014 |
|  | Skills related | | Lack of skills to undertake activities Pg6 paragraph 3 | Tasseron-Dries et al., 2021 |
|  |  | | Lack of communication skills among staff | Cohen-Mansfield & Meschiany, 2022 |
|  |  | | Staff’s lack of skills | Kolanowski et al., 2010 |
|  |  | | good communication skills | Clifford & Doody, 2018 |
|  | Difficulty of intervention individualisation | | difficulty in identifying specific songs for the playlist | Kwak et al., 2021 |
|  |  | | Difficulty of music individualisation | Garrido et al., 2021 |
| **5. Reinforcement** |  | |  |  |
|  | Recognition | | Lack of recognition from society, managers and relatives | Lawrence et al., 2016 |
|  |  | | Lack of being valued by the residential aged care facilities | Kwak et al., 2021 |
| **6. Intentions** |  | |  |  |
|  | Staff motivation | | lack of staff buy-in | Ducak et al., 2018 |
|  |  | | unwillingness of nursing staff to use MMD | Ducak et al., 2018 |
|  |  | | Lack of buy-in by direct care staff | Kwak et al., 2021 |
|  |  | | Staff's negative attitudes | Kong & Kim, 2022 |
|  |  | | Staff motivation | Griffiths et al., 2019 |
|  | Resident motivation | | Lack of resident buy-in | Kwak et al., 2021 |
|  |  | | Patient motivation | Forget et al., 2021 |
| **7. Emotion** |  | |  |  |
|  | Feeling towards interventions | | Negative attitude towards DCM™ | Griffiths et al., 2019 |
|  |  | | “Set and forget” attitude (using the music as an entertainment rather than as an opportunity for interaction with the resident) | Garrido et al., 2021 |
|  |  | | Animal phobia | Forget et al., 2021 |
|  |  | | Dislike of word ‘intervention’ | Lawrence et al., 2016 |
|  |  | | Fear of not producing a good voice | Gulliver et al., 2021 |
|  |  | | Activities they felt comfortable with | Tasseron-Dries et al., 2021 |
|  |  | | Using headphones | Kwak et al., 2021 |
|  | Feeling towards communication and interaction | | family caregivers did not feel welcome | Tasseron-Dries et al., 2021 |
|  |  | | Sense of frustration and powerlessness that their knowledge and experience undervalued | McKenna et al., 2022 |
|  |  | | Fear of criticism from training team | Lawrence et al., 2016 |
|  |  | | Fear of disapproval from ministry | Ducak et al., 2018 |
|  |  | | Concerns related to residents -Behavioural challenges and violence | Cohen-Mansfield & Meschiany, 2022 |
|  |  | | Caregivers feeling of insecurity | Kolanowski et al., 2010 |
|  |  | | feeling ‘uneasy’ around those with BPSD | Backhouse et al., 2016 |
|  |  | | empathy exhibited by the staff. | Janzen et al., 2013 |
| **8. Belief about consequences** |  | |  |  |
|  | Efficacy of the intervention | | Lack of effectiveness of the interventions | Ervin et al., 2014 |
|  |  | | Intervention not meeting staff’s expectation | McKenna et al., 2022 |
|  |  | | perception that NPI application was based on trial and error | Janzen et al., 2013 |
|  |  | | view that activities or NPIs are extras | Backhouse et al., 2016 |
|  |  | | Being pessimistic | McKenna et al., 2022 |
|  |  | | not seeing the value of music | Garrido et al., 2021 |
|  |  | | Intervention effectiveness | Forget et al., 2021 |
|  |  | | Improved quality of life of the elderly | Forget et al., 2021 |
|  |  | | Increased cognitive stimulation | Forget et al., 2021 |
|  |  | | Physical stimulation | Forget et al., 2021 |
|  |  | | Positive feedback from families and other caregivers | Forget et al., 2021 |
|  |  | | Positive response in the resident to an activity | Tasseron-Dries et al., 2021 |
|  |  | | Seeing results motivated them to utilise the intervention | Pieper et al., 2018 |
|  |  | | Seeing Results is believing | Ducak et al., 2018 |
|  |  | | Calming effect of music | Kwak et al., 2021 |
|  |  | | Seeing the positive effects of M&M | Kwak et al., 2021 |
|  |  | | Benefit gained(understanding) from the intervention or confidence | McKenna et al., 2022 |
|  |  | | Staff perceived value of volunteers | Van Der Ploeg et al., 2012 |
|  |  | | increased well-being for resident as perceived by volunteers | Van Der Ploeg et al., 2012 |
|  | Concern about the consequence of the intervention | | Concern about animal hygiene | Forget et al., 2021 |
|  |  | | Concern about animal’s quality of life | Forget et al., 2021 |
|  |  | | adverse events such as incidence of skin breakdown | Kaasalainen et al., 2019 |
|  |  | | Staff's hurtful experiences | Kong & Kim, 2022 |
| **9.Social/professional role and identity** |  | |  |  |
|  | Professional role | | Not viewing as nursing responsibility | Ervin et al., 2014 |
|  |  | | Nurse burden and responsibilities | Nunez et al., 2018 |
|  |  | | Concern about engaging all staff in intervention | Lawrence et al., 2016 |
|  |  | | Being asked to perform jobs that only staff members are trained to do. | Van Der Ploeg et al., 2012 |
|  |  | | Responsibility outside the job scope | Hussin et al., 2021 |
|  | Leadership | | designating a single staff member responsible for filling and turning on the diffuser each day (Pg 4 Group interview, paragraph 2) | Miller et al., 2021 |
|  |  | | championing by activity director (Pg 4 Group interview, paragraph 2) | Miller et al., 2021 |
|  |  | | Mapper status and leadership skills: respect held for the mappers within the home | Griffiths et al., 2019 |
|  |  | | Presence of a person with a motivational leadership style (Pg 7 Facilitating and impeding factors associated with the level of the team, paragraph 5) | Pieper et al., 2018 |
|  | Commitment | | Reluctance of family caregiver to take extra obligation | Tasseron-Dries et al., 2021 |
|  |  | | The culture of leaving everything to the nursing home | Tasseron-Dries et al., 2021 |
| **10. Belief about capability** |  | |  |  |
|  | Belief about capability | | Perceived difficulty to participate | Tasseron-Dries et al., 2021 |
|  |  | | lack of originality of the psychologist suggestions | McKenna et al., 2022 |
|  |  | | Need little training to implement (Pg 6 Enhancing factors and Hindering factors for NPI Implementation in LTC, paragraph 2) | Janzen et al., 2013 |

# Appendix 7. Synthesised findings with corresponding illustrations

| **COM-B Category: Capability- Psychological capability** | **Citations** | **Findings** | **illustrations** |
| --- | --- | --- | --- |
| **Knowledge** |  |  |  |
| **Synthesised Finding 1**: The education gap among the care staff and families may contribute to the slow implementation of NPIs. | Ervin et al., 2014 | Inadequate staff education as a limitation (U) | ‘‘Nurses need more training in behaviour management’’ ‘‘The skill mix of the team is sometimes a problem’’ (Pg 4, Behaviour oriented strategies in dementia care, paragraph 3) ‘‘Need education in this strategy’’ (Pg 4, Cognitive oriented strategies in dementia care, paragraph 3) ‘‘We need more training, by the right people, closer to home and at no cost’’ (Pg 5, Overall comments regarding management of BPSD, paragraph 3) |
| The implementation of NPIs may be facilitated if annual continuing education and short-term training for the senior as well as newly recruited care staff, incorporated courses on the causes and management of changed behaviours in dementia. In addition, bridging the gap between theoretical knowledge and practice may be beneficial. For example, physicians, certified nursing assistants and nurses highlighted that the current education format lacks adequate training. Finally, providing families of residents with dementia with education on dementia and its management may further support effective implementation. In addition, there is a sense of frustration and skepticism about the feasibility of training for caregivers at RACHs. For example, an activity coordinator reported that it is difficult to consider caregiver training due to work overload | Clifford & Doody, 2018 | Continuing education (U) | “It definitely would pay off for anyone working in dementia care to have some training in dementia and top up session, definitely a course or some documentation that they could sit down and actually read, just to understand what might be going on” (P9). (Pg 6 The impact of education on nursing practice, paragraph 3) |
|  | Kolanowski et al., 2010 | Lack of education (U) | “I don’t think that new CNAs come out of class knowing how to deal with combative, aggressive, or apathetic residents.” One participant stated, “. . . they don’t seek to understand the behavior; they just try to address it and I think that’s when you come up on failure because you don’t really understand what’s causing that behavior.” Physicians, CNAs, and nurse participants echoed that education “is just something that’s been missing in our educational format.” (Pg 5 The Educational Needs of Staff, paragraph 1) |
|  | Kong & Kim, 2022 | Staff's lack of education (U) | All participants requested continuing practical education of person-centred dementia care especially for care workers, as mentioned by one participant: ‘To provide person-centered care for residents with dementia, care workers should have received education about person-centered dementia care’ (Participant 4, RN). One care worker also said, ‘Nurses take education about dementia through continuing education or on their own, but care workers usually don't do that’ (Participant 3, care worker). (Pg 5 Staff's lack of education, paragraph 2) |
|  | Kong & Kim, 2022 | Family's lack of education (U) | Many families don't understand the dementia of their loved ones. As the residents with dementia are getting older, their dementia status is getting worse. But families don't understand the deterioration and show very sensitive response. (Participant 1, RN) (Pg 6 Family's lack of education, paragraph 1) There is a lack of understanding about dementia care among families of residents. We try to provide families with some education when meeting with them. But the number of families who participate in those meetings is usually very small … many families usually have little knowledge about dementia care in our nursing home. (Participant 2, RN) (Pg 6 Family's lack of education, paragraph 1) ‘Many families did not visit often residents with dementia, so they did not cooperate with us about person-centered care for those residents’ (Participant 2, RN). (Pg 6 Family's lack of education, paragraph 2) Families seem to need education about dementia and person-centered care. Through the education, they will be able to understand dementia of their loved one and the benefits of person-centered care. (Participant 12, care worker (Pg 6 Family's lack of education, paragraph 2) |
|  | Kwak et al., 2021 | Need to educate initial staff and new staff (N) | the need to educate initial staff and new staff due to turnover (n = 14) (Pg 8 Implementation barriers, challenges, and sustainability, paragraph 1) |
|  | Hussin et al., 2021 | Lack of Training (U) | “Only if the centre sends a representative to train the caregivers. It is at our own effort if we want to go for extra courses or classes.” (Shuhada, caregiver) (Pg 8, Barrier: Lack of Training and Skills paragrapgh 2) “I think I have never heard of this Malaysian Clinical Guideline.” (Rahmat, caregiver) (Pg 8, Barrier: Lack of Training and Skills paragrapgh 2) “My friend who was already in this field always taught me how to care for dementia patients. He doesn’t have any certificate of professionalism on how to care for them. So, he taught me only based on his experience.” (Taripnan, caregiver) (Pg 8, Barrier: Lack of Training and Skills paragrapgh 2) |
|  | Clifford & Doody, 2018 | Lack of education and training (U) | “Knowledge with nursing staff is at a level where they can understand, however, for the caring staff, mainly their knowledge isn’t the same, some of them have attended training, sometime their knowledge lacks in ways that it makes it hard for them to respond to responsive behaviours or try to deal with a situation” (P9). (Pg 7 The impact of education on nursing practice, paragraph 4) |
|  | Cohen-Mansfield & Meschiany, 2022 | Lack of training (U) | “Training the caregiver team it is naive to think that will happen. In the afternoon there is a heavy silence when there is no activity. They don't get enough compensation, so asking [staff] to do anything extra [e.g., undergoing training, conducting activities], is a joke” (#3, activity worker). (Pg 5 Institutional barriers, paragraph 4) |
|  | Kwak et al., 2021 | Training and support for M&M (N) | Other facilitators included having been provided training and support for M&M (n = 33) (Pg 9 Facilitators of Providing M&M., paragraph 1) |
| **Synthesised Finding 2**: Familiarity of RACH care staff with aged care residents with dementia may facilitate the use of NPIs. | Garrido et al., 2021 | Lack of accurate information about individual preferences(U) | I found it difficult. I had to rely on the music that he had in his CD collection. Communication was thumbs up or thumbs down a lot of the time and he also showed no emotion so it was difficult to know whether he was enjoying it or not enjoying it. (P5 Group 3) (P8, Challenges to Implementing Music Programs in Aged Care, paragraph 10) |
| The use of NPIs may be easier when caregivers know the residents well and maintain accurate records of their preferences. Additionally, remaining calm, devoting adequate time and connecting with them in a way that respects their dignity and independence could help residents feel respected and receptive to the interventions. For example, a general nurse highlighted the need for caregivers to manage their own behaviour, be calm and respect the personal space and unspoken desires of the individuals they care for, even when those individuals cannot clearly express what they want or don't want | Nunez et al., 2018 | Difficulty of connecting with and knowing residents (U) | ‘Because we are a diverse team ... But due to cultural differences we have found that some of the staff have got great difficulty in connecting and engaging with the residents. CSFG 2 ‘It’s just the little touches and if somebody comes in and is told, “Put bed number 24, 25 and 26 to bed now,” they do not know what their routine is. My mother cannot tell them. So sometimes it’s a bad night’s sleep.’ FCFG 2 (Pg 6 table 1 Connection and knowing a person) |
|  | Clifford & Doody, 2018 | Getting to know the person (U) | “ You need to get to know the person, to know their life, their history, to know everything about them, what they worked at, what they like to eat, their family, all their likes and dislikes” (P5). (Pg 6 Resources and interventions to support people with dementia and responsive behaviour , paragraph 5). |
|  | Kolanowski et al., 2010 | The inability of staff to meet the resident where they are at the moment (U) | “sometimes families aren’t able to give us a whole lot of history and if they haven’t had a close relationship with mom or dad ... that could really be a barrier.” (Pg 4 Reaching Out to the Person with Dementia, paragraph 3) |
|  | Kolanowski et al., 2010 | Lack of understanding among newly hired employees (U) | For example, one participant conveyed, If it’s a CNA that’s been here for a while and they know that particular person, then they’re going to know how to deal with the behaviors... versus somebody that’s brand new coming in and doesn’t know that particular resident. (Pg 5 The Educational Needs of Staff, paragraph 4) |
|  | Kolanowski et al., 2010 | Not knowing residents (U) | “sometimes families aren’t able to give us a whole lot of history and if they haven’t had a close relationship with mom or dad ... that could really be a barrier.” (Pg 4 Reaching Out to the Person with Dementia, paragraph 3) |
|  | Kolanowski et al., 2010 | Knowing residents’ past occupation and their interests (U) | Yeah I had a resident who was a farmer and you know very withdrawn and ... not engaged in life. Had lost meaning and purpose but by engaging him in therapeutic activities of just gardening that allowed him to continue to explore and to give him a sense of identity again. (Pg 4 Reaching Out to the Person with Dementia, paragraph 2) |
|  | Kolanowski et al., 2010 | Knowing residents timing and the concept of time (U) | So it’s a matter of us spending time, it’s not a matter of how big our budget is. But it’s not like we have all the time in the world ... when you have residents coming in now that are in their 90’s. Time was also referred to in reference to the resident’s altered sense of time. Yeah cause they’re on their own time zone. I mean 3 o’clock in the morning to us is whatever it is to them. They don’t follow any particular time ...so you really have to gear up to what is for them at that moment. (Pg 5 Reaching Out to the Person with Dementia, paragraph 8) |
|  | Janzen et al., 2013 | Familiarity with the resident (U) | A recreation coordinator explains ‘‘ ... the more time you [staff] spend with them [residents], the more you figure out what works. So, consistency in staff and routine for them [residents] is [a] big [factor].’’ (Pg 6 Facilitators and Barriers for NPI Implementation in LTC, paragraph 1) |
|  | Clifford & Doody, 2018 | Attitudes and being self -aware when caring (U) | “You have to be calm, you have to monitor your own behaviour, you have to respect their space, what they want, what they don’t want regardless of the fact that they might not be able to express it” (P6). (Pg 7 The care environment, paragraph 5) |
| **Synthesised Finding 3**: Familiarity of the care staff with NPIs and effective training comprehension may enhance NPI implementation. | Griffiths et al., 2019 | Mappers’ lack of understanding the DCM (U) | “The trouble is, when they came back [from the training], they weren’t able to explain properly what they had to do. So, you know, they were trying to explain it to us and we were finding difficulty understanding what was actually involved.” (Staff Member) Pg10 “I still don’t understand it … no one has been able to understand it to me fully… Every time I asked them [the mappers] to explain they were struggling. So I never got a full grasp of what it was all about.” (Manager) Pg10 |
| Familiarity of the care teams (nurses, managers, personal care workers or caregivers and others in the team) with the intervention, as well as their ability to comprehend and communicate the knowledge gained during training about NPIs will enhance their implementation. Additionally, the eagerness to learn among new staff may facilitate the implementation of NPIs | Griffiths et al., 2019 | Complex nature of DCM™ (U) | “Some of the things that certainly I picked up on, some of the things they found more difficult was around the kind of data analysis and report writing. That was the area that people seemed to find most difficult.” (DCM™ expert) Pg11  “So the report writing, yeah, was horrific to be honest. Very time consuming. Obviously we both had different roles at that point so quite demanding, so getting time, and it’s not a very quick process. Like I say it took quite a lengthy period of time … it was very demanding.” (Mapper) Pg11 |
|  | Tasseron-Dries et al., 2021 | Lack of knowledge (N) | None |
|  | Tasseron-Dries et al., 2021 | A clear structure for Namaste  (C) | “Maybe if you do this again, you could say more about what actually happens on these mornings. That’s a possibility. Like: we have a fantastic overhead projector and that will be there, and the music and you are welcome to join in. You could give your father and mother a hand massage, or we can do that. Maybe little more like that" (family caregiver, daughter) (Pg 7 ‘Communication’: Communication between family caregiver, staff and volunteer, Paragraph 2) |
|  | McKenna et al., 2022 | Little awareness of psychologist support (U) | …I couldn’t tell you what, what psychology means […] will it change the person? Will it do anything?... [Participant 7] (Pg 4 Theme 1: Expectation, paragraph 3) |
|  | McKenna et al., 2022 | Being newer staff willingness to learn (U) | …this is useful for someone who first comes in… [Participant 1] (Pg 6 Theme 3: Understanding, paragraph 3) … there’s a lot of new staff, and I think sitting down and actually listening to somebody think of why she’s actually behaving like this, I think they’d [like to] know… [Participant 9] (Pg 6 Theme 3: Understanding, paragraph 3) |
|  | Ducak et al., 2018 | Lack of understanding (C) | ... when I brought home all of the resource materials from the course I sat down with my staff and we ... went through each one as to how it would benefit or how they would explain it to other staff if they said, well, you know, ‘‘Why are you scooping golf balls into a muffin tin?’’ So we rationalized ... ‘‘OK, that motion will maintain dexterity so that they can continue to feed themselves.’’ So that makes sense to a nurse, to a PSW, so because there’s always that thought, ‘‘Well, this is childish or this is not appropriate.’’ Or that type of thing. (Pg 17, enabling factors, paragraph 2) |
|  | Ducak et al., 2018 | Knowledge of usefulness (C) | ... you can either spend the time responding to those behaviours OR you can implement the little two second activity and let them work on it for half an hour... Again they need that theory and the training and repetitive reminders as to it really isn’t time consuming – where do you want to spend YOUR time? consultant (C3) (Pg 17, enabling factors, paragraph 3) |
|  | Kolanowski et al., 2010 | Lack of knowledge about the effectiveness of NPIs (U) | One participant expressed, “Just a little touch of something (medication) is helping her get to activities, not crying, not upset all evening and night you know ... . So I don’t think no pharmacology whatsoever is the answer.” |
|  | Kwak et al., 2021 | Use of technology (N) | Use of technology was another major barrier (n = 62). Respondents reported issues related to charging the equipment (n = 22), using iTunes (n = 4) and Wi-Fi (n = 3), downloading music (n = 3), as well as other equipment issues (n = 17), programming issues (n = 3), and problems with accessibility to iPods or a computer to load songs (n = 12). (Pg 8-9 Implementation barriers, challenges, and sustainability, paragraph 2) |
|  | Gulliver et al., 2021 | Staff familiarity with music (U) | One staff member believed that since they already conducted musical sessions, they would not find it difficult - “one of the family member’s daughter, she plays the piano. And then we had another family member, the son plays the ukulele…they come in and we do the singing” (S5). (Pg 7 Issues in continuing the program. Paragraph 1) |
| **COM-B Category: Capability- Physical capability** |  |  |  |
| **Skills** |  |  |  |
| **Synthesised Finding 4**: Lack of skills and experience among the care staff may impair the implementation of NPIs. | Griffiths et al., 2019 | Lack of skills or experience (U) | “For some of the care workers writing anything was a real challenge. You know they just not, not used to putting descriptions down, let alone sort of feedback type questions to ask.” (DCM™ expert) Pg10 |
| The staff's lack of experience and skills to tailor interventions to individual needs, inability to communicate in a language that the resident understands, inability to explain what is going to be done and failure to discuss the care plan and care needs of the resident with their family may hinder to the implementation of NPIs. | Ervin et al., 2014 | Lack of experience  (U) | ‘‘I have no experience’’, ‘‘Lack of experience’’ (Pg 4, Cognitive oriented strategies in dementia care, paragraph 3) |
|  | Garrido et al., 2021 | Difficulty of music individualisation  (U) | “It’s not just a case of having music. It’s being able to hear the type of music that that person can relate to” (P2 Group 3) (P8, Challenges to Implementing Music Programs in Aged Care, paragraph 9) I could not get mum to enjoy really anything new or something she wasn’t familiar with … It didn’t matter how beautiful the music was, how closely related it was to something else that she really liked. If it didn’t strike that familiarity …. (P3 Group 3) (P8, Challenges to Implementing Music Programs in Aged Care, paragraph 9) |
|  | Clifford & Doody, 2018 | Good communication skills  (U) | “..good communication skills, making sure you explain everything you are going to do, having regular meetings with staff that are looking after someone and discuss the care plan, discuss their care needs, discuss with the family more often ” (P7). (Pg 7 The care environment, paragraph 7) |
|  | Tasseron-Dries et al., 2021 | Lack of skills to undertake activities (N) | none |
|  | Cohen-Mansfield & Meschiany, 2022 | Lack of communication skills among staff (U) | “How to achieve communication [is a need]. Are there any tools that can be given to therapists? Warmth, smiles? Most employees do not have the skills to do so” (#21, CEO). (Pg 4 Concerns related to other staff, paragraph 1) “most of the staff are foreign workers ... language and turnover [are issues]” (#14). (Pg 4 Concerns related to other staff, paragraph 1) |
|  | Kolanowski et al., 2010 | Staff’s lack of skills (C) | majority of participants stated that interventions are “not going to fit every person” and a variety of interventions or activities are attempted or “pulled out of your bag of tricks and used.” One participant conveyed, “we try to reorient them; try to focus their attention on something else ... explore all possibilities.” (Pg 5 The Educational Needs of Staff, paragraph 2) |
|  | Kwak et al., 2021 | Difficulty in identifying specific songs for the playlist (N) | identifying preferred songs (difficulty in identifying specific songs for the playlist), finding music (n = 22) |
| **COM-B Category: Opportunity- Physical opportunity** |  |  |  |
| **Environmental context and resources** |  |  |  |
| **Synthesised Finding 5**: The competing demands on managers, coupled with the lack of attention from caregivers regarding the effectiveness of NPIs may impact the implementation. | Griffiths et al., 2019 | Competing priorities (U) | “We get inspected by health and safety, infection control, the social workers, CQC [regulatory authority] come, social services come, y’know, it’s just ongoing and they are all asking for more paperwork… we are struggling to do the paperwork that we have got already.” (Manager) Pg 6 |
| Managers should find ways to overcome the competing demands on top of non-pharmacological interventions (e.g., dementia care mapping). In addition, caregivers should prioritise NPIs over other competing activities and value the effectiveness of NPIs. For example, a music therapist pointed out that caregivers often treat the music as background noise, not considering whether it is beneficial or detrimental to the resident's well-being | Griffiths et al., 2019 | Time gap between training and undertaking DCM implementation(U) | “They went for that training … then there was a gap and I kind of think if they had just gone straight in and done the mapping, they might have done it. But I feel that when a few weeks passed, they were struggling to say how we do this… maybe they didn’t have the confidence, you know what, to roll it out.” (Manager) Pg 7 |
|  | Garrido et al., 2021 | Tendency for staff to have background music on (U) | “I’ve had situations where there’s just music on in the background and a lot of our carers, they’re not conscious of it. They’re not thinking of ‘how is music being effective or not effective in this situation’, because for them they can shut it out. It’s just background noise. Whereas it might be really important for the resident. There needs to be something built into the cycle around monitoring it so it really brings it into the consciousness. (P3 Group 1)” (P6, Challenges to Implementing Music Programs in Aged Care, paragraph 4) |
| **Synthesised Finding 6**: Environmental modification tailored to aged care residents with dementia may enhance the implementation of NPIs. | Ervin et al., 2014 | Lack of dementia specific unit (U) | ‘‘Aggressive residents are inappropriately placed in facilities who cannot manage. Problem behaviours affect all other residents. Some young staff are intimidated and afraid of aggressive residents’’. ‘‘Residents with behaviours disrupt all residents in the facility and should be in dementia specific units’’. (Pg 5, Overall comments regarding management of BPSD, paragraph 5) |
| The provision of NPIs for BPSD may need making changes to the physical surroundings to accommodate the unique requirements of the aged care residents with dementia. For example, a separate dementia speciﬁc unit, a dedicated storage room for equipment, ample natural light outside. The proximity of volunteers’ residence to the nursing homes may increase their participation in the implementation of NPIs | Garrido et al., 2021 | Challenge with safe storage of iPod or headphones (U) | “We had a challenge trying to figure out how best to store them safely while also providing good access to everybody” (P6 Group 2).” (P8, Challenges to Implementing Music Programs in Aged Care, paragraph 8) |
|  | Tasseron-Dries et al., 2021 | Living close to nursing home enhanced volunteer support (N) | none |
|  | Miller et al., 2021 | Unfavorable weather conditions to use natural light outside (U) | Multiple sites reported using natural light indoors by opening window blinds, and one staff stated “[resident’s] mood is better when it’s bright and sunny outside.” (Pg 4 Natural light, paragraph 1) |
|  | Ducak et al., 2018 | Difficulty to alter a deeply ingrained medical nature of LTC (U) | We’re just dealing with a primarily medical environment, right? And, while even the government’s trying to get away from that it’s hard when you’re dealing with people who have been in nursing for 20 years and suddenly be open to this whole other way of nursing. recreation manager (R12) (Pg 13 limiting factors, paragraph 4) |
|  | Kolanowski et al., 2010 | The changing landscape (U) | For example, individuals with dementia constitute a large percentage of the residents staff cares for, and these residents are much more impaired today than in the past: Twelve years ago ... we had more physically able folks... .When I first started here there weren’t all these personal care homes. So now that they are, by the time they come to us it’s really difficult to get them involved in things that they can actually do. (Domain of time.) (Pg 3 The Changing Landscape, paragraph 1) |
|  | Kong & Kim, 2022 | Inappropriate physical environment (U) | On the second floor, there is only one room where residents with severe dementia stay together. I think there should be more space for residents with dementia to stay, wander around, and receive personcentered dementia care without interfering with other residents' lives. (Participant 20, care worker) (Pg 5 Inappropriate physical environment paragraph 2) |
| **Synthesised Finding 7**: The lack of sufficient funds may hinder the use of NPIs. | Forget et al., 2021 | Budget constraint (U) | "the first thing to be asked will be: How much does it cost?! "; "Expensive training and far away, which generates costs "; " the cost was much too high for this to be done "; " we did not have the budget "; " if we cut the budget aspect, because "; "the effects would be in the long term, it is difficult when everything is finance-led"(ANIM06, PSY04, AMP05, ANIM09, ANIM06) (P3 budget, paragraph 1) |
| Lack of adequate funds for training, accessible and easy-to-use equipment, buying music iPod and headphones, activities, resources and appropriate salaries for the care staff may impair the implementation of NPIs | Clifford & Doody, 2018 | Lack of funding (U) | “..it (the programme) finished because of the cost of the programme and the cost of care is not reflected in the fees that are negotiated through the National Treatment Purchase Fund” (P8). (Pg 6 Resources and interventions to support people with dementia and responsive behaviour , paragraph 3). |
|  | Lawrence et al., 2016 | Lack of resources-pressurized environment (U) | You feel like you’re not doing your job properly. You actually feel that you’re letting the residents down. Yeah that you’re letting them down. You say, ‘I’ll be with you in a minute, I’ll be back’…and you’re not, you’re running off for something else. (3003) (Pg 3 table 1 (1b) Lack of resources) |
|  | Miller et al., 2021 | Activity supplies disappearing (N) | none |
|  | Ducak et al., 2018 | Lack of funding (U) | ‘I think some of our barriers here are more financial barriers because I think it would be beneficial to send more staff to the workshop but financially that’s not feasible’ participant (R4) (Pg 13 limiting factors, paragraph 11) ‘... we have six people in our department and they couldn’t afford to send every one of us’. recreation assistant (R5) (Pg 13 limiting factors, paragraph 11) |
|  | Cohen-Mansfield & Meschiany, 2022 | Lack of regular, professional and individualized activities (U) | When staff were asked what changes, they would like to see implemented, they referred not only to the need for increased budget and workforce (#29, occupational therapist), but to activities for residents: “Residents need to [be occupied], many are sitting idle. There is no one to approach residents who do not cooperate” (#1, head nurse). (Pg 5 Institutional barriers, paragraph 1) |
|  | Cohen-Mansfield & Meschiany, 2022 | Lack of financial resources (U) | “There is a salary problem. The Ministry of Health is willing to give [only] a minimum [wage]... The issue of wages is very difficult and therefore the whole issue of manpower” (#27, CEO). (Pg 5 Institutional barriers, paragraph 1) |
|  | Cohen-Mansfield & Meschiany, 2022 | Lack of equipment and adequate facilities (U) | “Bad bathing chairs, which need to be moved from room to room” (#2, nurse). (Pg 5 Lack of equipment and adequate facilities, paragraph 1) “There is a need for a garden, a balcony, more activities” (#1, head nurse). “Nobody has hearing aids, an issue that is not addressed. Seventy percent do not hear well” (#15, occupational therapist). (Pg 5 Lack of equipment and adequate facilities, paragraph 1) |
|  | Kaasalainen et al., 2019 | Lack of funding (U) | If we could have funding for someone to be in there I would definitely be on the waiting list to try and apply for that position. (Site 2, PSW/CA, page 4) (Pg 10 Recommendations to implement ‘Namaste Care’ in new LTC homes, 5) |
|  | Kwak et al., 2021 | Costs for buying music (N) | Costs for buying music, iPods, and headphones were also a barrier (n = 28) (Pg 9 Implementation barriers, challenges, and sustainability, paragraph 3) support from others (mostly volunteers) (n = 15) (Pg 9 Facilitators of Providing M&M., paragraph 1) |
|  | Kwak et al., 2021 | Accessibility of equipment (N) | accessibility of equipment (n = 31); equipment characteristics, such as being portable, small, and easy to use (n = 21); (Pg 9 Facilitators of Providing M&M., paragraph 1) |
|  | Kwak et al., 2021 | Funding (N) | financial support and donations (n = 20) (Pg 9 Facilitators of Providing M&M., paragraph 1) equipment donations (n = 15). (Pg 9 Facilitators of Providing M&M., paragraph 1) |
| **Synthesised Finding 8**: The lack of an effective manager at RACHs may hinder the implementation of NPIs. | Griffiths et al., 2019 | Lack of RACH manager support (U) | “as far as I’m aware they were just pushed to be doing other stuff and it kept getting left and left and not done.” (Staff Member) Pg 6 |
| The lack of a strong, committed, well-aware, innovative and open-ended manager who avoids scapegoating is open to being challenged positively by the care staff and leads the intervention could impair the smooth application of NPIs. | Griffiths et al., 2019 | The hierarchical nature of RACHs sometimes acted as a barrier (U) | “It’s mainly from a confidence perspective, [they] were clearly not confident to challenge a manager who was not supporting.” (DCM™ expert) Pg 7 |
|  | Griffiths et al., 2019 | RACH manager support (U) | “I think management support, you know, it can either be amazing when it’s amazing or it can be a real difficulty if the manger isn’t supportive.” (DCM™ expert) Pg 7“They were very clear that they thought DCM™ was fantastic, because they saw it as a way of improving the quality of their care to take their home CQC [UK regulator of care homes] rating from good to outstanding.” (DCM™ expert) Pg 7 |
|  | Forget et al., 2021 | Organizational constraints (U) | "Difficulties in finding a person to be in charge of the dog because of the responsibilities involved"; "due to training in Alsace (remote location)"; "It is a question of time"; " there is always at least one person from the structure with ‘her’"; " we were told: a dog is not possible, because it needs surveillance"; “it needs to be taken out all the time "; " after each session we analyze what happened "; "little inconvenience except the logistics (feeding, nighttime, care)” (PSY04, ANIM03, PSY08, AMP05) (P3 Organizational constraints, paragraph 1) |
|  | Forget et al., 2021 | Management constraint (U) | "/close-minded management"; "I put pressure on the management", "reluctance of the management in terms of hygiene"; "we need to convince them" (ANIM03, PSY07, ANIM06) (P3 management, paragraph 1) |
|  | Clifford & Doody, 2018 | Lack of management support (U) | “Nursing management need to be on board, they need to understand, they wouldn’t understand what it is you are trying to do for the patient, I don’t think they understand why you are harping on about the compliment of staff and why you are constantly looking for (additional resources)” (P5). (Pg 7 The care environment, paragraph 4) |
|  | Pieper et al., 2018 | Organisational changes or other innovations at the time of the implementation (N) | none |
|  | Ducak et al., 2018 | Support from management (U) | ... making sure from top-down in the long-term care facility that there’s administrative buy-in that the management staff, the director of care buys into this too, because ... it’s a bottom-up training so it’s at the field level, anybody can use it, we just need the top-down to support it. consultants (C5) (Pg 18, enabling factors, paragraph 12) ... any given day there could be so many emergencies and so many crisis situations because ...they don’t have [adequate] resources...this Montessori for example, goes under unless there is a manager who is very strong and who can very determinedly take the agenda forward. consultant (C2) (Pg 18, enabling factors, paragraph 13) ...sometimes it’s just an ear when I want to vent and say, ‘‘What do you think about this?’’ And sometimes it’s validation, like they’ve been certified and can go, ‘‘No let’s go back to the theory and practice.’’ You know, to get me back on the right track. consultant (C3) ) (Pg 18, enabling factors, paragraph 14) |
|  | Kaasalainen et al., 2019 | Strong support from administration (U) | Oh the staff comes to me and tells me there’s nobody in the room so I have to organise with the staff who is willing to go and who is willing to stay on the floor. So we talk, like the team on the first floor we talk about who can go. (Site 1, nurse, page 2) (Pg 9 Facilitators to implementing ‘Namaste Care’, paragraph 1) |
|  | Backhouse et al., 2016 | Inequality in the allocation of activity provision (U) | that are able to voice or … can show their frustrations about not having something to do, whereas other people that are sitting in their chair might feel equally as bad, but can’t voice it or express it in any way (Barbara, General Nurse, CH3 (Pg 5 Barriers to including residents in activities, paragraph 5) it’s normally the same people, the same little group (Holly, Activity Worker, CH2) (Pg 5 Barriers to including residents in activities, paragraph 4) |
|  | Clifford & Doody, 2018 | Lack of manager support by scapegoating (U) | “Management would say that if the resident needs behaviour management, that maybe here is not the best facility for them, should we consider special dementia care unit, so we don’t get any support” (P1). (Pg 7 The care environment, paragraph 6) “I think my colleagues would think at times that the person is doing it out of spite, it’s the person not the dementia’s fault.” (P5). (Pg 7 The care environment, paragraph 6) |
| **Synthesised Finding 9**: Lack of flexibility in daily routines could deter the use of NPIs and increase reliance on medication. | Ducak et al., 2018 | Rigid routine (U) | ‘there’s such a routine, a rigid routine, like meals are this time and bath is this time and, personal care is this time’ (R2) (Pg 13 limiting factors, paragraph 1) |
| Lack of well-established individualised routines, such as mealtime, bathing time, personal care and sleep time, hinder the adoption of NPIs (e.g., Montessori interventions). Additionally, the lack of targeting at triggers of behaviours (e.g., sleeplessness) and the absence of adjustment to sleep time based on individuals’ routines could increase the use of medications. | Ducak et al., 2018 | Medicalized nature of LTC and adherence to routines (U) | ‘behaviours are presented but they’re looked at as behaviours that need to be treated rather than looking at what causes these behaviours’. consultant (C2) ) (Pg 13 limiting factors, paragraph 4) |
|  | Cohen-Mansfield & Meschiany, 2022 | Rigid care routine (U) | For example, some residents were given sleeping pills to adjust their schedule to the majority: “[...] There is an hour when the majority goes to sleep with some given sleeping pills [only] to [to be] awoken at early hours in the morning” (#14, CEO). (Pg 4 Concerns related to residents, paragraph 5) |
| **Synthesised Finding 10**: Lack of *adequate staff time hinders personalised NPIs.* | Griffiths et al., 2019 | Staff time constraints (U) | “It’s all getting the time and people are rushed off their feet in the morning and they haven’t got time to come here for half an hour.” (Mapper) Pg 6 |
| Lack of sufficient staff time could slow the implementation of individualised NPIs as it may result in some residents being overlooked. | Griffiths et al., 2019 | Mappers’ time constraints (U) | “I’d say it depends on your workloads and things that day, like how much is implemented. This thing took a lot of time, when there is not as many staff on as you need, and like I say, we have several people who are end of life, and things like that. Priorities are more that way at the time.” (Mapper) Pg10“I had to do my normal working hours, plus a lot of the time, a lot of extra hours, because we were short. I was sometimes doing 40 odd hours a week. Then, coming in and trying to do the typing up on top of that, especially the last one with it being over Christmas - it was taking a lot longer to do, than I would have liked really.” (Mapper) Pg11 |
|  | Ervin et al., 2014 | Time constraints (U) | ‘‘Time limitations, not enough time and staff to cover for 1:1 time for patients’’ ‘‘Staff numbers and time taken to use this strategy is a major limitation’’ ‘‘1:1 time. I feel others miss out while time is devoted to the person with behaviour’’ (Pg 4, Behaviour oriented strategies in dementia care, paragraph 2) ‘‘Time — too busy’’ ‘‘Lack of staff and time’’ ‘‘Time is a huge constraint for nurses’’ ‘‘Lack of staff and time’’ (Pg 4, Stimulation oriented strategies in dementia care, paragraph 2) ‘‘Always very busy and rushed attending to personal care needs. Only get very limited 1:1 time’’ ‘‘Lack of staff numbers to allow time for these strategies’’ (Pg 4, Emotion oriented strategies in dementia care, paragraph 2) ‘‘The greatest impact on good outcomes for behaviour management is time limits. Nurses are always under pressure to hurry. We need more staff allocated to spend time with residents who have behaviour problems’’. ‘‘The biggest problem is time constraints, if we had more staff then we could spend more time with people with behaviours. For one staff member to be devoted to caring for a behaviour management problem leaves the rest of the ward short’’ (Pg 5, Overall comments regarding management of BPSD, paragraph 2) |
|  | Garrido et al., 2021 | Lack of time (U) | “They would turn the TV set on in the room in the morning … They just didn’t have the staff number to really carry on music therapy, just even playing CDs for my mum. (P3 Group 3)” (P6, Challenges to Implementing Music Programs in Aged Care, paragraph 1) “It seems that time is always a concern as well for staff because they see it as an extra task to be done. I think that’s the result of not understanding how music can benefit them, the resident, and how they can use it. (P4 Group 1)” (P6, Challenges to Implementing Music Programs in Aged Care, paragraph 2) |
|  | Tasseron-Dries et al., 2021 | Lack of time (N) | none |
|  | Lawrence et al., 2016 | Time constraints likely to undermine intervention (C) | There has been so much focus on it recently, dealing with challenging behaviour, creating different activities, etc. Every nursing home in the country would be more than happy to do that, but what people need to realise is that to do that costs a lot more money. To give one to one intervention is very expensive. Whereas it’s dead easy isn’t it if you give them a few tablets? (2002) (Pg 3 table 1 (1b) Lack of resources) |
|  | Miller et al., 2021 | Lack of time to implement aromatherapy (N) | none |
|  | Ducak et al., 2018 | Limited time available for recreation staff to get resident onboard (U) | ‘it’s hard because we only have three programs in there a day so it’s... nursing staff and PSWs [Personal Support Workers] that are there with them all the time but we’re having a hard time getting them on board.’ participant (R6) (Pg 13 limiting factors, paragraph 6) |
|  | Kaasalainen et al., 2019 | Staff burden over time (U) | It’s really really busy in the morning that we usually find it hard for us because you can’t be alone and we still have people in bed and you have to wait for the other side to get help, but the other side maybe they are still busy too you know. That’s the struggle that we have right now. (Site 2, PSW/CA, page 1) Being that is a very busy time of the morning, and getting people there you know with the care aides it is hard to get people there in the morning, and we don’t have the staff. (Site 2, recreation therapist, page 1) There have been families that have complained ...that they don’t like their people not getting enough attention, because someone has gone off the floor. (Site 2, PSW/CA, page 3). The care needs now are much, much heavier than they were 10 years ago ..., I think there are many staff that are almost overwhelmed in their day-to-day responsibilities without Namaste. (Site 2, nurse, page 3) (Pg 9 Barriers to implementing ‘Namaste Care, paragraph 2) |
|  | Kwak et al., 2021 | Lack of time (U) | Respondents listed lack of time required by the program—time to “set up and maintain the playlist,” “distribute iPods,” “keep the program up to date and current for new residents,” and “assure iPods is still playing and follow up.” (Pg 8 Implementation barriers, challenges, and sustainability, paragraph 2) |
|  | Gulliver et al., 2021 | Insufficient time (U) | Several staff members believed insufficient time was a significant barrier to conducting these types of activities – “No…they’re focused on getting their care work done…to do something like that you need someone that’s going to sit there with them” (S2). (Pg 7 Issues in continuing the program. Paragraph 1) |
|  | Kong & Kim, 2022 | Lack of time (U) | Participants mentioned too many residents and lack of time as difficulties in implementing person-centred dementia care: Now, for us, it's hard to provide person-centered care to our residents with dementia because we have too many residents. For breakfast, I have to assist eight residents, so I am too busy to pay attention to other residents, like what and how much they eat. (Participant 8, care worker) (Pg 5 Insufficient staff paragraph 2) Emotional care requires sufficient time. But we don't have enough time to sit and talk with our residents. (Participant 2, RN) (Pg 5 Insufficient staff paragraph 2) |
| **Synthesised Finding 11**: Lack of well-trained, qualified and stable staff, could be a barrier to the use of NPIs. | Griffiths et al., 2019 | Staffing related issues (lack of nurses and other staff, staff turnover, mappers attrition) (U) | “Because we are only a residential home, erm, y’know, we haven’t got nurses and staff so my staff aren’t that confident anyway… I’m glad we got involved because we got a lot out of it, I’m just disappointed that we weren’t able to continue.” (Manager) Pg 4“Care homes are really, really busy. Turnover of staff in care homes can be quite dramatic at times, and the realities are there’s other pressures on them isn’t there.” (DCM™ expert) Pg 5“It were definitely better having two rather than having just doing it on my own, because I think I would’ve struggled a lot more” (Mapper) Pg 6 |
| The absence of a sufficient number of well-trained and qualiﬁed permanently employed nurses, caregivers and other staff members, as well as volunteers could be a barrier to the use of NPIs. Staff turnover may cause staff burnout due to high workload leading to inadequate implementation of NPIs. | Forget et al., 2021 | Work overload (U) | "Some have complained about fear of an additional workload"; "it would take time for that"; "the disadvantage is in terms of cleaning "; "no additional time is allocated to caregivers for activities”. (PSY08, PSY04, PSY02, ANIM09) (P3 workload, paragraph 1) |
|  | Nunez et al., 2018 | Insufficient staffing levels (U) | ‘I know when I see one nurse and one care assistant on duty for a whole floor, there’s shouting going on.’ FCFG 2 (Pg 6, table 1 Insufficient staffing levels) ‘Is there any factors which affect your ability to provide good care at night?’ ‘It’s always short-staffed.’ FCFG 1(Pg 6, table 1 Insufficient staffing levels) ‘I was worried at night because I knew there were less staff on. It was more likely that it would happen when nobody was there to stop him.’ FCFG 1(Pg 6, table 1 Insufficient staffing levels) ‘Not enough staff’ FCOS ‘But sometimes, not matter how much surveillance you can have, accidents are going to happen. Yes? You could have somebody who falls in front of you. So night time, you could be dealing with somebody having heart failure. Another person falling down at the same time. And they have to deal with emergencies, so the nurses have to think very, very quickly.’ CSFG 2 ‘She fell off a chair on one occasion, and when I came in the next time, I could see that they were more concerned that I was going to sue them than they were about sorting— it’s health and safety gone mad, which is true in so many areas of society at the moment.’ FCFG 2 (Pg 7, table 1 Insufficient staffing levels) |
|  | Hussin et al., 2021 | Inadequate staff numbers and time constraints (U) | “These dementia residents have more needs than normal elderlies. Sometimes, more than one of them act up at the same time. When I go to one patient, another one acts up and shows behaviours. That is why sometimes we just tie one patient while we go to entertain another.” (Taripnan, caregiver) (Pg 8, Barrier: Inadequate staff numbers and time constraints paragrapgh 2) |
|  | Clifford & Doody, 2018 | Shortage of staff (U) | “When you are down staff, your hands are tied because you can’t provide the quality of care that you want and it’s frustrating because you know the other staff are going to get stressed” (Pg 6 Resources and interventions to support people with dementia and responsive behaviour , paragraph 2). |
|  | Webster et al., 2022 | Understaffing at night-time (U) | I think it’s just loneliness because sometimes we’ll sit with her, and we can chat for hours. Sometimes we don’t have the time. (15; female care assistant) Although we have some nights that we’re short. . . Here in this floor, it should be four staff working. One is one to one, two staff on the floor and then the nurse. So, if one has cancelled their shift so the nurse will be working on the floor or sometimes sitting as one to one and then the other two staff will be ensuring the safety of other residents. (7; female nurse) (Pg 8 Barriers to managing sleep disturbances, paragraph 4) |
|  | Miller et al., 2021 | One-on-one time requirements from staff to implement natural light (N) | none |
|  | Pieper et al., 2018 | Staff turnover, shortage of staff and high workload (N) | none |
|  | Ducak et al., 2018 | Very low staff-to-resident ratio (U) | ...the one-to-one sort of really intense programming, I would say, about 10 percent. Because it comes down to those ratios and the time, however, we do try to apply it to all the programs, well OK, say 80 percent of the programming we provide. recreation manager (R12) (Pg 13 limiting factors, paragraph 9) ... our dementia unit is quite active right at the moment and at this point it’s attention span and staffing levels. Because there’s only one activation person to 28 [residents] so ... if she’s not monitoring for agitation or aggressive people then the person’s able to utilize the Montessori but it, sometimes their role has to be different than programming. recreation manager (R7) (Pg 13 limiting factors, paragraph 10) |
|  | Cohen-Mansfield & Meschiany, 2022 | Lack of manpower (U) | “Over the last five years, there has been a decline in the workforce, less [quality], employees work in three jobs. This is a problematic situation” (#21, CEO) (Pg 5 Institutional barriers, paragraph 2) Work practices are dictated by a lack of manpower ... a method of dividing work, each nurse has his or her own residents. You need to double the manpower...” (#24, head nurse). (Pg 5 Institutional barriers, paragraph 2) “There are almost no permanent employees, high degree of burnout, many employees have to work in additional places” (#14, CEO). (Pg 5 Institutional barriers, paragraph 2) |
|  | Kaasalainen et al., 2019 | Understaffing (U) | I think there would be a need for more staff or at least more volunteers because I think it would be manageable with the appropriate amount. (Site 2, daughter, page 3) (Pg 10 Recommendations to implement ‘Namaste Care’ in new LTC homes, 4) |
|  | Kwak et al., 2021 | Inadequate staffing (U) | Other related barriers included inadequate staffing (n = 9) (Pg 8 Implementation barriers, challenges, and sustainability, paragraph 1) |
|  | Janzen et al., 2013 | Time constraints and low staff-to resident ratios (U) | An RN described the typical situation, ‘‘... at times there’s so little staff and there’s a lot of behaviors all at once. It’s just kind of putting out fires and keep things rolling ... ’’ (Pg 6 Facilitators and Barriers for NPI Implementation in LTC, paragraph 3) |
|  | Chaudhry et al., 2020 | Workload with engagement of staff in routine activities (U) | ‘Many times I had to send your team back without doing any work. I am very sorry for that as sometime our staff are bit stuck in other activities that’s why I was unable to get participant to deliver some of your sessions on time’ (PCW2); ‘Every care worker have different responsibilities and schedule due to which it is difficult for all of us to attend something at same time’ (PCW3) |
|  | Kong & Kim, 2022 | Insufficient staff  (U) | All participants mentioned insufficient number of RNs, NAs and care workers per resident pointing out inappropriate national staffing standards: Primarily, there are not enough staff compared to the number of residents. Currently, the government mandates at least one care worker for 2.5 residents and one nurse for 25 residents. But considering that we have three shifts per day, two nurses are now expected to take care of 90 to 100 residents and we have three care workers for 24 residents. (Participant 9, RN) (Pg 5 Insufficient staff paragraph 1) |
| **Synthesised Finding 12**: Resident reluctance to engage in activities hinders NPI implementation. | Tasseron-Dries et al., 2021 | Reluctance to engage in unfamiliar activities  (C) | “They (family caregivers) say: “I am not very keen on sitting next to my mother and then giving her that hand massage… It makes people uncomfortable. But if you bring in balloons to shoot those across the table, then they’ll join in without a problem. That’s not physical….”. (manager) (Pg 6 ‘Activities’: Preferences of family caregivers for activities with their relative living with dementia, paragraph 3) |
| Challenges faced by caregivers in engaging individuals with advanced dementia in activities, such as difficulties in keeping them awake during the day and overcoming initial resistance to participation lead to slow implementation of NPIs. | Webster et al., 2022 | Difficult to encourage and keep people awake in the daytime (U) | We try to keep him awake but it’s no chance. You can’t. He just sit[s] down and he’s too tired and he close his eyes. (1; female care assistant) Earlier on this month there was a time he was so tired he would just eat a piece of his breakfast and go to bed and sleep. We try as much to keep him busy during the day but he’s quite a strong headed person. (3; female team leader) (Pg 5 Evening strategies to promote sleep, paragraph 1) |
|  | Webster et al., 2022 | Difficulty during the night to encourage residents to go back to their rooms (U) | If he doesn’t want to go to his room, we just have to leave him because we just don’t want him to kick you or hit you. So, whenever he’s sleeping in the chair, and you try maybe, “go to your room”, one, two, three [times]. He says, “no.” (5; female care assistant) Because she couldn’t really express every time that this is what I want. . . So, they don’t know what causes the agitation at night. (7; female nurse) (Pg 8 Barriers to managing sleep disturbances, paragraph 2) |
|  | Backhouse et al., 2016 | Residents’ Reluctance to take part in activities  (U) | Holly, an activity worker, touches on the issue: I have to try and get them … say ‘oh come on, do you want to do it?’ ‘no, no, no’ ‘come on’ but once they’re doing it they’re absolutely fine, it’s like when we done all the sunflowers … Mable was going ‘oh I can’t do that, I can’t draw’ but … she absolutely loved it in the end (Holly, Activity Worker, CH2) (Pg 5 Barriers to including residents in activities, paragraph 9) |
| **Synthesised Finding 13**: The severity of the resident's physical and cognitive impairment as well as comorbid conditions may slow the use of NPIs. |  |  |  |
| Residents with severe cognitive impairment, physical incapability and poorly managed comorbid conditions before being transferred to a nursing home are less likely to participate in activities. | Kolanowski et al., 2010 | Rise in resident acuity level or impairments (U) | They don’t keep residents in the hospital a long time. So you know you’re dealing with a lot of medical things. That unfortunately has to be our priority and the poor resident that’s here with dementia is sort of left behind. (Pg 3 The Changing Landscape, paragraph 2) |
|  | Kolanowski et al., 2010 | Residents' inability to recognise one's level of impairments (U) | A lot of the elderly people forget what they can and cannot do. So many of them think they can walk. They want to be independent and they’re not able to because of physical disabilities. So we’re trying to keep them safe, but I think at times they feel like we’re trying to hold them back and which will aggravate them even more. (Pg 4 resident behaviors, paragraph 3) “sometimes families aren’t able to give us a whole lot of history and if they haven’t had a close relationship with mom or dad ... that could really be a barrier.” (Pg 4 Reaching Out to the Person with Dementia, paragraph 3) |
|  | Backhouse et al., 2016 | Physical or mental impairment (U) | I feel awful saying this … it’s just very hard to actually get them to do, that they can physically do anything .. once they [care staff] did suggest may be go and put a tambourine in their [the residents’] hand … I just found that must be patronising to be honest .. I didn’t feel comfortable doing that … I suppose I’ve kind of veered away from those residents um, because … it’s very hard to know activity wise what to do. (Jess, Activity Worker, CH3) (Pg 5 Barriers to including residents in activities, paragraph 1) |
|  | Griffiths et al., 2019 | DCM™ did not suit some residents (U) | “…some of our residents are quite, quite poorly so it doesn’t work for them, it just depends how well they are.” (Staff member) Pg 8 |
|  | Ervin et al., 2014 | Resident’s refusal to comply or participate  (U) | ‘‘Sometimes the residents refuse to participate’’ ‘‘Residents cognitive ability and attention span can limit this intervention’’ ‘‘Residents who have advanced dementia do not respond to these strategies’’ (Pg 4, Cognitive oriented strategies in dementia care, paragraph 4) ‘‘Usually patients are not cognitively able to communicate these memories, those that can seem to become depressed from such discussions and therefore it is avoided’’ ‘‘In severe dementia they don’t even recognise familiar people’’ (Pg 5, Emotion oriented strategies in dementia care, paragraph 4) |
|  |  |  |  |
| **Synthesised Finding 14**: **The severity of the behaviour among aged care residents with dementia could make it difficult to implement NPIs** | Clifford & Doody, 2018 | Frequency and severity of resident's behaviour (U) | “we are used to dealing with people that strip or call out, or spit, but if it’s ongoing, if it’s constant every day, that puts too much pressure on and it’s stressing for them and other residents ” (P3). (Pg 7 The care environment, paragraph 3) |
| The resident's aggressiveness (e.g., incompatibility between carers and aged care residents with dementia), mood swings and tendency to cross boundaries with volunteers make it difficult to provide tailored NPIs. | Chaudhry et al., 2020 | Fluctuations in mood, behavioural problems, verbal outbursts of residents (U) | ‘Yes, they do have a lot of mood swings. At one moment they are very cooperative, but in the next they become totally opposite, like at one moment they agreed to take a bath, but when they were taken to the washroom, they started beating us. Their behaviour changes so abruptly’ (PCW2). (Pg 5 table 2 Experience of working with older adults) |
|  | Van Der Ploeg et al., 2012 | Residents crossed boundaries (U) | Like if you give out your phone number, not only are you opening yourself up to get random phone calls when you’re not in your volunteer role. I suppose it’s not appropriate to get to that, to keep within those bounds and just remember your place and that you’re not a family member and you’re not a doctor. (#18) (Pg 5-6 Perceived benefits and difficulties, paragraph 11) |
|  | Van Der Ploeg et al., 2012 | Witnessing agitation as a barrier (U) | There’s a lady here right this moment who is very distressing. She’s ... you might have heard her calling out. (#38) One thing that’s overall distressing is watching people become sicker and sicker, and eventually be moved on ... it’s also a sad thing to watch people suffer from dementia. (#47)(Pg ) (Pg 5-6 Perceived benefits and difficulties, paragraph 12) |
|  | Kolanowski et al., 2010 | Aggressiveness against caregiver (U) | Sometimes we have an all black crew and this person does not like black people and is saying don’t let that “N” touch me ... we cannot possibly say well we’ll get a white CNA for you. So you just try to assure them that, that person is there to help them. Sometimes we can switch a male CNA with a female CNA because some of the older white ladies perceive black men as being a danger to them. (Pg 4 The Changing Landscape, paragraph 2) |
| **COM-B Category: Opportunity- Social opportunity** |  |  |  |
| **Social influences** |  |  |  |
| **Synthesised Finding 15**: Collaboration among care staff, volunteers and families of aged care residents with dementia may influence the implementation of NPIs. | Griffiths et al., 2019 | Staff reluctance to support the mapper (U) | “I would say in that home there’s two very definite groups of staff, the ones who want to see progress, who would support the mapper, who would want to encourage her and make it work, and there was also a very strong group of people who say you know ‘what she thinks she’s telling us’.” (DCM™ expert) Pg 8“It became a chore and one lady I can think of in particular was very excited and motivated about it, and became less so because of the challenges. And that’s really sad to see. Someone who had that real passion to just go “do you know it’s just too hard”, but initially is like “I’m happy to come in on my day off because I think it’s marvellous”, but when you’re not then getting that support it you know wears you out really. Wears you down.” (DCM™ expert) Pg 9 |
| The lack of collaboration among the care staff, between day and night shift staff and between family and staff and volunteers could make it challenging to implement NPIs. The advanced age of the family caregiver may also be a barrier to their active participation with nursing home staff. *On the other hand, the passion of the staff for change, collaboration among the staff, between family and staff, teamwork between allied health professionals and caregivers and fostering good relationships between caregivers and residents' relatives may be the facilitators of NPI implementation.* | Griffiths et al., 2019 | Difficulty to engage the staff team(U) | “The second time around we held a meeting and nobody came … We did try like you know individual, a few minutes at a time, but I don’t think they took it seriously enough, do you know what I mean?” (Mapper) Pg 8 |
|  | Griffiths et al., 2019 | Whole home’ engagement approach (U) | “There was a really big crowd actually, and it did include lots of different disciplines of staff, including the painter and decorator and maintenance man, which was great.” (DCM™ expert) Pg 7 |
|  | Griffiths et al., 2019 | Collective reflection on DCM™ feedback (U) | “In most cases when it happens, it’s a negative experience because there’s inspectors from various organisations, so I think it wasn’t until we started giving feedback and there was quite a bit of positives in there that the staff really got engaged with the process.” (Manager) Pg 8 |
|  | Nunez et al., 2018 | Night and day care staff working relationship (U) | ‘Do not worry about it, the day staff will be in a minute. It’s always like this battle [laughter]. That’s what we need to stop; we need to work as one, as opposed to working as a battle against each other. “We are better than them.” Or “They are better.” “We do a better job.” Or “We do not know what we do.” I think, all staff, really, should work day and that we should work night’ CSFG 2 ‘they will blame it on the night staff, because they think they have not attended to them. It’s just somehow you need—both parties have to know their residents. So that if you know somebody is resistant to care, you know you are not going to be able to attend to this person when you think you want to give that care.’ CSFG 2 ‘ (Pg 7 table 1Night and day care staff working relationship) ‘Because we are thinking, “It’s harder in the day than night, because they are just sleeping.” All the patients are just lying in their bed’ CSFG 2 ‘So, you need tell your colleague, “Look, we have tried, tried, tried. It did not work out.”’ So then there’s not the kind of surprise as, “Oh, my God, look at that. They have let it go to us and we are not able to fix it.”’ CSFG 2 That is a day plan, okay? So I doubt very much again—not being sort of overcritical. Those staff where my wife was, they come for the evening, for the night shift, and they want to have a good sleep, the staff. FCFG 2 (Pg 6 table 1 Connection and knowing a person) |
|  | Hussin et al., 2021 | Less supportive family members (U) | “But some clients are very egoistic … I don’t think they will take the tips and change according to our suggested plan and tips.” (Gaya, caregiver) “But when the children come, they will talk about all the negative points and fight with the elderly. This makes the elderly depressed. Family unity is not there.” (Gaya,caregiver) “She just ate but she forgot, and she tells her daughter that she did not eat. The daughter came and scolded me for not feeding her. I snapped a picture of her eating and ‘WhatsApp’ the daughter.” (Yona, caregiver)(Pg 11 Barrier: Less supportive family members paragraph 1) |
|  | Hussin et al., 2021 | Multidisciplinary collaboration (U) | “Doctors and nurse often come. They will come this evening and check the patients, staff, rooms and sometimes they also check the facilities here.” (Venus, caregiver) “I notify the relatives about the behaviour and how frequent it happened. Then the decision is from the relative or family member, it’s either they want to bring them to a private doctor, in house doctor or the geriatrician to start on medications.” (Yona, caregiver) (Pg 11 Strategy: Multidisciplinary collaboration paragraph 1) |
|  | Clifford & Doody, 2018 | Collaboration between nurses and activities coordinator (U) | “While we have an activities coordinator we also do dementia specific activities, we do reminiscence, relaxation therapy, and we give each other feedback to say they enjoyed it” (P9). (Pg 6 Resources and interventions to support people with dementia and responsive behaviour , paragraph 4). |
|  | Tasseron-Dries et al., 2021 | Old age of the family caregiver, conflicts within the family and caregiver burden (N) | none |
|  | McKenna et al., 2022 | Absence of joint working (U) | …she [the psychologist] thinks she read it in a book and she’ll come and tell us what we’re doing wrong or right I think. I’ve known [the resident] for years and, and you just come and you say, “it’s not”. I don’t say it but some say, like a bit of a kid coming in telling me how to, you know, tell your grandma how to suck eggs… [Participant 1] (Pg 5 Theme 2: Working together, paragraph 3) |
|  | McKenna et al., 2022 | Team working (U) | … the rest [of the ideas] was from us. But she was just inputting what could you do to, you know, like steer him away from getting agitated, getting aggressive, and this is what we come up with […] so it should be quite simple, you know to put together… [Participant 10] (Pg 6 Theme 2: Working together, paragraph 6) involve like a more, like a two way sort of, questioning between both. The psychologist could say something, and then the staff member could say, “well I’ve tried this but when I was trying this he or she did this”, so we know it’s not working this way. So it could be more like a two-way sort of thing… [Participant 3] (Pg 6 Theme 2: Working together, paragraph 7) |
|  | Lawrence et al., 2016 | Relationships strained with relatives critical of staff (U) | I think they just don’t understand what dementia is, they try to blame the staff for whatever has happened. I think this is frustrating because when we are trying our best and then somebody will come in and tell you ‘no, no, no’. It’s like we have a lady who goes in her room and messes all her clothes up and her daughter comes in and then they think she thinks it’s the care staff that have to put it away properly, when it isn’t. (1002) (Pg 3 table 1, (2b) Relationships with relatives) |
|  | Lawrence et al., 2016 | Divisions between staff groups (U) | What, hang on, what if you’ve got staff which you get in every home with a really negative attitude or say are just very negative, ‘oh no that’s not going to work’. Yeah and very narrow minded and it’s going to be a case of trying, as well as trying to implement this with the residents, it’s going to be difficult trying to get the staff to act on this as well. (1004) (Pg 4 table 1, (2c) Relationships within the team) |
|  | Lawrence et al., 2016 | Mutual respect and reciprocity key to good care (U) | People bring their dogs in, bring their family in, pictures, we all send postcards if we go on holiday…it’s a lot like life as a general is all shared.(1001) And they like a laugh, a joke, rather than treating them like, you treat them like you treat your Nan… because obviously they were born and bred in Dagenham, so they talk like we do…there’s no need for airs and graces or anything like that. (3004) (Pg 3 table 1, (2b) Relationships with relatives) |
|  | Lawrence et al., 2016 | Collective responsibility enables staff to meet resident needs (U) | We all work as a cog in a wheel and if one of those cogs breaks then the wheel doesn’t turn does it? So what we do is we all work together it’s like they work upstairs with the carers and if something is wrong they report here and then it gets reported to the doctor…That is the heart of the person centred care because if we don’t have that we won’t know the person’s needs. It won’t be met without us knowing. (3001) (Pg 4 table 1, (2c) Relationships within the team) |
|  | Webster et al., 2022 | Residents’ family’s complaint to staff (U) | Because the son is always complaining, “my mum is not comfortable. She has to go to bed.” So sometimes exactly after lunch, he’ll want his mum to go to bed. (5; female care assistant). So, the nurse told us that we should reduce the wine in the daytime. Because it’s making him sleep less in the night. But when his children comes [to visit], her daughter, when she comes, she brings some from the house. (16; female care assistant) (Pg 8 Barriers to managing sleep disturbances, paragraph 5) |
|  | Pieper et al., 2018 | Having different schedule hindered interdisciplinary cooperation (U) | Registered nurse: “The hardest thing was working together on-the job in subgroups, which consisted of different disciplines. Since we all had different schedules and days off, but at the same time had to assess the steps in groups, someone took the lead and then others took over if we had only a short time together to fill-out the forms. That’s how we solved it.” (Pg 8 Facilitating and impeding factors associated with the level of the team, paragraph 5) |
|  | Pieper et al., 2018 | Interdisciplinary learning and cooperation (U) | Nurse assistant: “What I really liked was the fact that we were participating in this training as a whole multidisciplinary team including all related disciplines, not only as a single nursing team. For example, a psychologist looks at problems in a different way, i.e. from another point of view. I thought: I’ve never really seen it that way – but I guess you’re right.” ……… “I think it contributed to the fact that the barriers for contacting the other disciplines have become smaller, they’re more easily accessible now.” (Pg 8 Facilitating and impeding factors associated with the level of the team, paragraph 2) Psychologist: “The nursing staff has a lot of fun in finding out why someone behaves in a certain way. Now, they ask me at an earlier stage how to deal with challenging behaviours, and as such I can do my job better, more targeted, and with more members of the team.” (Pg 8 Facilitating and impeding factors associated with the level of the team, paragraph 2) |
|  | Ducak et al., 2018 | Lack of interdisciplinary collaboration in LTC (U) | My heart goes out to the recreation staff because it seems they really just lack credibility with the nursing staff ...They put them lower down on the totem pole, unfortunately. ...I think the rec staff know what they do but I don’t know that in their training they have that extra level of understanding the medical side of things, so sometimes that’s where the downfall is... And so over my years when I’ve been consulting to the recreation department, it’s even simply changing their language or understanding a diagnosis more because if you ask an RN [Registered Nurse] to explain what’s going on medically they’re happy to share it, but I think the rec staff get scared ...the nurses... can talk down to them, so I understand their fear too. consultant (C5) ) (Pg 13 limiting factors, paragraph 5) |
|  | Ducak et al., 2018 | Support from family (U) | ... we do run into who pays for it. In this case though, the families have never had a problem with that because really it’s never very expensive, it’s usually less than 20 dollars, and they, the families, love the individualized attention. consultant (C4) (Pg 18, enabling factors, paragraph 15) |
|  | Kwak et al., 2021 | Lack or inconsistency of volunteers and family support (N) | Other barriers included lack or inconsistency of volunteers (n = 3), and families that were not supportive or helpful (n = 12). (Pg 9 Implementation barriers, challenges, and sustainability, paragraph 3) |
|  | Kwak et al., 2021 | Support of facility personnel (N) | The most frequently cited facilitator was support of facility personnel (basically all staff including administrators), family, and volunteers to implement and maintain the program (n = 55) (Pg 9 Facilitators of Providing M&M., paragraph 1) |
|  | Kwak et al., 2021 | Family and volunteers involvement (N) | family involvement (bringing music, donating shuffles to use, turning the iPod on, being supportive of the program) (n = 33). (Pg 9 Facilitators of Providing M&M., paragraph 1) |
|  | Kong & Kim, 2022 | Lack of trust between staff and families (U) | For the successful implementation of person-centered dementia care, I think that families need to totally trust staff. All of us, as a team, have to take care of residents with dementia. When wandering, some residents with dementia might have an accident and get a small bruise or skin excoriation on their arms but some families think it is caused by staff's abuse, there is mistrust. (Participant 2, RN) (Pg 7 Lack of trust between staff and families, paragraph 1) |
|  | Kong & Kim, 2022 | Conflicts among/between staff and families (U) | One time we wanted to place a bedridden resident with dementia in a wheelchair and take her for a walk, but some staff disagreed. So we could not do it. Although we were co-workers, our opinions were often different. (Participant 10, care worker) (Pg 7 Conflicts among/between staff and families, paragraph 1) When there are accidents, some families grill us about whose fault it is. (Participant 3, care worker) (Pg 7 Conflicts among/between staff and families, paragraph 1) [To provide person-centered care] we have to establish good relationships with families. If family cooperate well, it is easy to discuss and deal with the behaviors of the residents with dementia. But there are many families who do not cooperate. (Participant 2, RN) (Pg 7-8 Conflicts among/between staff and families, paragraph 2) |
|  | Van Der Ploeg et al., 2012 | Bonding between residents and volunteers as positives (U) | I think if they didn’t have the volunteers then my thinking would be that they’re not stimulated enough to self-motivate and stimulate themselves, so therefore they would just sit there and drop off to sleep. (#13) (Pg 5 Perceived benefits and difficulties, paragraph 3) And the fact that somebody’s on one to one basis with the residents, that’s the whole ... it’s not about [painting of] the [finger]nails, it’s about the contact. They [residents] often open up to the volunteers better than to staff, because we basically attend, some of us attend to tasks only, not necessarily to do ... be ... a holistic communication. (#4) (Pg 5 Perceived benefits and difficulties, paragraph 3) |
| **Synthesised Finding 16**: The quality of communication among staff, between staff and aged care residents with dementia and between staff and families may influence the implementation of NPIs. | Forget et al., 2021 | Improved social interaction (U) | l. "They talk to each other about the dog, if they had ever had one"; "People show completely different behavior when the dogs are there. There are people who, basically, are completely withdrawn, sleeping, in their armchairs, completely curled up. The dogs arrive and that’s it they stand up, open their eyes, open up, look around themselves to see what’s going on, it’s different ";" we make contact with the person ";" people can start to speak. (ASG0, ANIM06, ANIM10) (Pg 5 social interaction paragraph 1) |
| Communication among staff members about the resident's care plan, promoting communication between caregivers and residents of the same ethnic and linguistic background, engaging in slow and attentive conversation with eye contact with the resident the use of elderspeak when necessary and ensuring effective communication between staff and aged care residents with dementia may promote the implementation of NPIs. On the other hand, a lack of effective communication between staff and families could impair the implementation of individualised NPIs. Additionally, volunteers’ loneliness in terms of their gender may demotivate them from participation in the implementation of NPIs. | Nunez et al., 2018 | Communication as a challenge (U) | ‘Staff have to know the care plan of the residents and do they miss out on days, because of days off. And when they come on duty, they know...aware of any changes of the person’s condition. Because that’s forever changing. ’CSFG 2‘so although I did ask the respite care manager to let me know whether they administerher any sleeping medication or to keep her agitation down, they never did—’FCFG 2‘I think the only feedback I got about nights was if something had basically happened. Ifhe had been in some sort of incident, they are legally obliged to tell you anyway...But Iknew if I came in at, say, 11:00 in the morning and he wasn’t there, then somethingprobably went wrong at night.’FCFG 1‘I’d see my husband had bruises. I’d look and I’d think,“How? He could not get out ofbed.”You know they never volunteer the information, you have to go and ask.’FCFG 2‘with the first 2 ambulance trips in at night because of pneumonia, I found out that he’dbeen put on haloperidol. This was to stop all the problems the care home were havingwith him at night, but I’d never been told about them, or the fact that they’d asked his GPor Rx haloperidol for him.’FCOS (Pg 5 table 1 Communication as a challenge) |
|  | Hussin et al., 2021 | Communication barrier (U) | “I changed her caregiver to be with the same race. As Indian prefers to be with an Indian to take care of them because they can talk in the same language.” (Gaya, caregiver) “At the start, she speaks English but towards the end, she will start to speak Malay … so obviously I don’t understand her, and I have to remind her to speak English.” (Venus, caregiver) (Pg 10 Barrier: Communication barrier paragraph 1) |
|  | Hussin et al., 2021 | Creating meaningful conversations with PWD (U) | “If they are still aggressive, we just speak gently to them and never oppose whatever they said at that time. If they think that their children will pick them in the evening, we just have to agree with them even if it’s not going to happen, and use that modality to persuade them to go eat or change diaper.” (Venus, caregiver) “We have to slow talk with them while making eye contact and body language. At that moment they will express their feeling, maybe they want something. From there, we are able to identify what is their need.” (Akim, caregiver) (Pg 9 Strategy: Verbal and non-verbal communication paragraph 1) |
|  | Tasseron-Dries et al., 2021 | Feeling like a man in a woman’s world (U) | “I was the only man, you know. And then, well, it’s different. And then you see how those women interact with the residents. Yes, that is different. Plus, the residents are all different. All different. But, so, on Wednesday there are two male volunteers. That is really good. Makes a change ….” (family caregiver, spouse) (Pg 6 ‘Personal circumstances’: Personal context of family caregivers, paragraph 4) |
|  | Tasseron-Dries et al., 2021 | Misconceptions and unclear communication (N) | none |
|  | Kong & Kim, 2022 | Lack of communication among staff, residents and families (U) | Some families are very sensitive, so it is better put some distance between. They misunderstand my words and then they go to the office of administrator and complain about that. So it is very difficult to communicate with them. (Participant 8, care worker) (Pg 7 Lack of communication among staff, residents and families, paragraph 1) When families institutionalize their loved ones, they do not share one hundred percent of the information about their loved ones. They seem to be afraid that their loved ones will be rejected [for admission] by the nursing home. Families do not tell us details about their loved ones, which hinders our implementation of person-centered care for residents with dementia. (Participant 19, NA) (Pg 7 Lack of communication among staff, residents and families, paragraph 1) When an older adult is admitted to the nursing home, a nurse and a social worker take a history of the older adult. Usually care workers do not attend. Although we share important information, it is impossible to share all the information about the residents among 10 care workers on the team for the implementation of person-centered care. (Participant 3, care worker) (Pg 7 Lack of communication among staff, residents and families, paragraph 2) |
|  | Van Der Ploeg et al., 2012 | Managing a range of personalities in group activities  (U) | Not towards me, not towards volunteers no, but I see things that I don’t like .... There was somebody who couldn’t breathe, he couldn’t talk and I said “look this man needs some oxygen, he can’t breathe.” (Staff response:) “Oh we don’t.” I said, “You’ve got to have oxygen, of course” and she repeated “We don’t have oxygen” and she just didn’t care. And because I insisted she went and got the bottle of oxygen to help. See things like that. (#42) (Pg 5 Perceived benefits and difficulties, paragraph 9) |
|  | Van Der Ploeg et al., 2012 | Interacting with older people was enjoyable as perceived by volunteers  (U) | I have laughter here, belly laughter .... So I feel quite relaxed and like this is my second home. I get more back from what I give them, I receive more so I’m leaving here quite uplifted. (#29) |
|  | Hussin et al., 2021 | Elderspeak  (U) | “Yes, we treat them as a baby and so far, none has become angry because we treat them like that. They in fact like it, I don’t know … maybe they like the tone of our voice when we call them.” (Catherine, caregiver) (Pg 10 Elderspeak paragraph 1) “The staff addresses each resident with politeness and respect by using their name and credentials such as Doctor or Professor. The staff speaks to them by normal communication, like an adult-to-adult communication.” (Observational notes) (Pg 11 Elderspeak paragraph 1) |
| **COM-B Category: Motivation- Automatic motivation** |  |  |  |
| **Reinforcement** |  |  |  |
| **Synthesised Finding 17**: Lack of recognition for the work of caregivers and allied health professionals could slow the implementation of NPIs. | Lawrence et al., 2016 | Lack of recognition from society, managers and relatives (U) | So they [the government] really have to recognise that the care workers are doing a highly skilled, professional job, they don’t take it seriously. Even when I am out there and somebody asks me, ‘what are you doing?’ you know a care job and the way people, even the way that the relatives look at you because you are doing this job, you can’t win. And they can’t do it. So really I feel that they don’t recognise the care job is a good thing, they think we just come here to wash somebody, but that is not what we do. (2004) (Pg 3 table 1 (1a) Lack of recognition) |
| Implementation of NPIs may not be likely to be successful if caregivers and allied health professionals don’t feel appreciated and supported by the government, residents' families and the community. | Kwak et al., 2021 | Lack of being valued by the residential aged care facilities (U) | My concern as a music therapist is that facilities will think they can just throw headphones on seniors to give them music, which could in turn devalue the work of a music therapist, costing music therapists their jobs. Music listening is a very tiny piece of the puzzle of the power of music. It can also do damage if not administered properly. (Pg 7 Value of M&M., paragraph 3) |
| **COM-B Category: Motivation- Automatic motivation** |  |  |  |
| **Emotion** |  |  |  |
| **Synthesised Finding 18**: Feeling of staff resistance, fear from families and feeling overwhelmed hinder NPI implementation, while resident preference alignment and ownership of NPI enhance its implementation. | Griffiths et al., 2019 | Negative attitude towards DCM™ (U) | “I felt that the ways that people had been working prior to that, the culture of the place, whilst there was a lot about it which I would really commend it for, there were definitely some things that needed to be looked at. And I felt that there was a reluctance to look at that. And there was quite a lot of defensive response.” (DCM™ expert) Pg 7-8 |
| Feeling resistance from staff about the intervention, fear of families about the intervention, misperception of the word intervention as a correction of something wrong and feeling scared with the intervention (e.g., staff frightening of letting their voice out*)* are hindrances whereas matching between the preferences of residents and the interventions and sense of ownership of the intervention by the resident are enhancers of NPIs implementation. | Garrido et al., 2021 | “Set and forget” attitude (using the music as an entertainment rather than as an opportunity for interaction with the resident) (U) | “It’s something to interact with. When you’re interacting with the resident not just putting headphones on and walking away, we found that that does not work nearly as well as when you are interacting with the music. (P6 Group 2)” (P, Challenges to Implementing Music Programs in Aged Care, paragraph 3) |
|  | Forget et al., 2021 | Animal phobia (U) | "The downside is for families who are afraid of dogs when they see one, but it’s quite rare,"; “there are people who don’t like dogs, or even fear them. I have seen fear and apprehension in people who have never had animals.” (PSY02, ANIM06) (P3 animal phobia, paragraph 1) |
|  | Tasseron-Dries et al., 2021 | Activities they felt comfortable with (U) | “They also do music [….]. If you don’t like this, then you don’t go. But if there is table shuffeboard [old Dutch board game] or whatever, and you enjoy that, then you will go there. So, I think that that is very personal.” (family caregiver, daughter) (Pg 4 ‘Activities’: Preferences of family caregivers for activities with their relative living with dementia, paragraph 2) |
|  | Lawrence et al., 2016 | Dislike of word ‘intervention’ (U) | As soon as you say we are having an intervention, it’s like what you have done wrong needs to be assessed and then we are going to better it through our intervention and we are going to intervene in activities, we are going to intervene in this and this. And to me it’s more of an association with us, working with us to do these things and helping to guide whereas intervention sounds like we have done something wrong. (2004) (Pg 3 table 1 (1a) Lack of recognition) |
|  | Kwak et al., 2021 | Using headphones (U) | “They liked hearing all their favorite songs back to back. Many will smile when we put it on them. They like knowing that they have their own music that they don’t have to share.” (Pg 8 What residents liked about M&M, paragraph 1) |
|  | Gulliver et al., 2021 | Fear of not producing a good voice (U) | Other staff were adamant that this would not be possible, suggesting staff may be “a little bit frightened of letting their voice out and… because it is a daunting thing” (S2), and that they felt you had to have “a good voice” (S3). (Pg 7 Issues in continuing the program. Paragraph 1) |
| **Synthesised Finding 19**: Volunteers' frustration with staff communication, care staff's sense of undervalued knowledge and experience and residents' uncooperative behaviour hinder NPI implementation, while staff empathy enhances its implementation. | Tasseron-Dries et al., 2021 | Family caregivers did not feel welcome (U) | “I think it would be prudent to say, of course you can visit, but please remember that the Namaste program is underway and please slow down, relax. Yes, exactly. That it works differently. That you don’t put up barriers in advance, like, well it’s Namaste, so we’d better not visit then.” (family caregiver, daughter) (Pg 7 ‘Communication’: Communication between family caregiver, staff and volunteer, paragraph 3) |
| Volunteers' frustration with staff communication, the sense that care staff knowledge and experience are undervalued in the view of the expert (e.g., psychologist), caregivers' feeling that residents are uncooperative, restless and shouting is a hindrance to NPI implementation, while staff empathy for residents enhances their implementation. | McKenna et al., 2022 | Sense of frustration and powerlessness that their knowledge and experience undervalued (U) | …I work on the floor where she comes, that’s my, the floor I work on, and she hasn’t spoke to me once…I don’t feel like I can approach them, I wouldn’t approach them to ask them, any information…like I say they don’t really acknowledge, they just come in, do their job and then they go… [Participant 2] (Pg 5 Theme 2: Working together, paragraph 2) |
|  | Lawrence et al., 2016 | Fear of criticism from training team (U) | I’m a little bit concerned now because obviously somebody’s going to be coming in and you know again it goes back to the active living team when we had them in and they, the way that they spoke to us and the way it was sort of didn’t they, we weren’t good enough and they were sort of telling us how to do our job and that’s what I’m a little bit concerned about that someone’s going to come in and say do this do that and we think ‘hang on’. (1004) (Pg 3 table 1 (1a) Lack of recognition) |
|  | Ducak et al., 2018 | Fear of disapproval from ministry (U) | Some of the barriers [are], you know, nursing being task-oriented and thinking that the Ministry is not going to allow for this. Everybody’s nervous, you know, and I’ll just say in our area ...residents... like to do household chores. So that would take over some of our staff’s chores if they allowed them to, but they’re so nervous of the Ministry. ...Like if they make the bed and not do it correctly. I know that they’re driven by the Ministry of Health, and so are we, but if [residents] don’t make the bed properly they figure if an inspector comes in and trying to explain that would just be way harder than just doing it themselves. recreation manager (R11) (Pg 13 limiting factors, paragraph 2) |
|  | Cohen-Mansfield & Meschiany, 2022 | Concerns related to residents -Behavioral challenges and violence (U) | “Violent, full of violence, [we are] assaulted endlessly. Nothing [you can] do” (#2, nurse), (Pg 3 Concerns related to residents, paragraph 1) “Sometimes [we are subject to] residents’ lack of cooperation, restlessness, shouting” (#14, CEO) (Pg 3 Concerns related to residents, paragraph 1) “There is a resident whom one is not to touch; if I move him, he might give me a punch” (#18, activity worker) (Pg 3 Concerns related to residents, paragraph 1) “issues of communication and violence are really important” (#29, occupational therapist). (Pg 3 Concerns related to residents, paragraph 1) |
|  | Kolanowski et al., 2010 | Caregivers feeling of insecurity (U) | “We said we can medicate her, the greatest good for the greatest number [in relation to woman who was screaming].” |
|  | Janzen et al., 2013 | Empathy exhibited by the staff (U) | A highly empathetic unit manager understood that living in a complex LTC environment influenced adverse behavioral responses, ‘‘imagine leaving your own environment, coming into a place where it’s not familiar ... living with other unfamiliar people ... and having care done by people you don’t recognize.’’ Empathy of the staff appeared to coincide with openness to using NPIs(Pg 6 Facilitators and Barriers for NPI Implementation in LTC, paragraph 2) |
|  | Backhouse et al., 2016 | Feeling ‘uneasy’ around those with BPSD (U) | It’s like outings … I don’t think they’re [care staff] willing to help out as much because they don’t want to help people with dementia. Where they’re more willing to help people that … have got their full faculties … so I think some dementia people do get, um, misunderstood and mistreated, um, not saying physically mistreated, or, I’m just like neglected with … activities (Karen, Activity Worker/Senior Care Worker, CH1) |
| **COM-B Category: Motivation- Reflective motivation** |  |  |  |
| **Intentions** |  |  |  |
| **Synthesised Finding 20**: The facility manager and staff buy-in are critical in the implementation success of NPIs. | Griffiths et al., 2019 | Staff motivation (U) | “The manager would come in and you know be really enthusiastic. They came to the briefing, everybody was at the briefing, the whole home, the manager of the home, do you know what I mean. The company really bought, really bought in to DCM™. And the two girls, the two mappers were just really enthusiastic about it, … and really, really tried their hardest.” (DCM™ Expert) Pg 7“You really have to get quite a few people across the organisation thinking in the same way to sort of drive that change.” (Manager) Pg 7 |
| When the manager is excited about an intervention, the care staff is supportive and the residents are also on board, the NPI is more likely to be implemented smoothly. | Forget et al., 2021 | Patient motivation (U) | “As soon as people arrive”;” motivation to try to make gestures”;” a gentleman who never wants to leave his room comes to walk the dog”;” Ms. X accepts to be washed in the presence of the dog”; “it’s a new activity, which is not like the ones usually offered, and it affects other people” (ANIM03, ANIM09, PSY02 PSY08)(Pg 5 patient motivation paragraph 1) |
|  | Ducak et al., 2018 | Lack of staff buy-in (U) | Staff buy-in is another problem where I go in and I say, ‘‘I would like you to try this.’’ And they’re like, ‘‘Well, I have to get eleven people out of bed. I’m not doing that for him.’’ It’s a very task-oriented work environment and so to add something that is sort of unique or a little bit out of the box is threatening to some of the staff, especially those who have been there forever... So staff attitudes is a big deal. consultant (C5) (Pg 13 limiting factors, paragraph 3) ... everybody can do this, it’s not just programming, PSWs can do these activities, the registered nurse when she’s going by with the med cart can ... it’s getting everybody’s buy-in that they’re all responsible. They really still think it’s just programs’ and recreation’s job. (Pg 13 limiting factors, paragraph 6) |
|  | Ducak et al., 2018 | Unwillingness of nursing staff to use MMD (U) | C4: I think sometimes it’s better to have an outside source come in and promote it. And then they see that it’s something that the resident needs rather than something that the activity department is responsible for. ... a lot of times it’s easier to say, ‘‘Oh, that’s her job, not mine.’’ But if I come from the outside, I’m hoping that the nursing staff will do it more. ... And the nursing staff that I’ve talked to have been very supportive about it. They really liked it. (Pg 13 limiting factors, paragraph 7) |
|  | Kwak et al., 2021 | Lack of buy-in by direct care staff (N) | Lack of buy-in by direct care staff (e.g., nurses, CNAs, and other direct care staff) to initiate, deliver, and follow up with M&M for residents was the most frequently cited barrier to providing M&M (n = 85). (Pg 8 Implementation barriers, challenges, and sustainability, paragraph 1) |
|  | Kwak et al., 2021 | Lack of resident buy-in (N) | resident buy-in (n = 19). (Pg 9 Implementation barriers, challenges, and sustainability, paragraph 3) |
|  | Kong & Kim, 2022 | Staff's negative attitudes (U) | One participant said, ‘I think that some staff's mindset were wrong. They do not have a sense of mission’ (Participant 1, RN) Some staff have worked for a long time so they have mannerisms… When we care for residents with dementia, we have to talk to the resident more and provide any stimulus, but they only change diapers, take the residents to physical therapy, and feed them lunch; that's it. I feel sorry about that. (Participant 13, RN) (Pg 6 Staff's negative attitudes, paragraph 1) The problem is that some senior staff don't want to provide person-centered care for residents with dementia. They are lazy. So some passionate new staff are bullied so they are discouraged in the provision of person-centered care. (Participant 2, RN) (Pg 6 Staff's negative attitudes, paragraph 2) |
| **COM-B Category: Motivation- Reflective motivation** |  |  |  |
| **Belief about consequences** |  |  |  |
| **Synthesised Finding 21**: The care staff’s belief in the benefit of NPIs and families seeing their effectiveness could enhance their implementation. | Ervin et al., 2014 | Lack of effectiveness of the interventions (U) | ‘‘Very difficult to know which strategy will work with each client’’ ‘‘Resident may not comprehend, and family may not consent to participation’’ ‘‘Depends on day and time. What works once doesn’t work again. They get tired of some strategies being too frequently used. Sometimes improvisation works’’ (Pg 4, Behaviour oriented strategies in dementia care, paragraph 4) ‘‘Sometimes when talking about spouses the demented person thinks they are still alive and starts looking for them’’ ‘‘Some residents become upset talking about family. We have to be mindful of past history’’ (Pg 5, Emotion oriented strategies in dementia care, paragraph 3) |
| When both care staff and families see the impact of NPIs on the quality of life of aged care residents with dementia, their implementation could be enhanced. | Forget et al., 2021 | Intervention effectiveness (U) | "We targeted people with dementia or motor disorders"; "I remember a gentleman who did not leave his room and he agreed to go for walks with the dog."; " Elderly people with cognitive disorders or anxious people ";" end of life support ". (PSY08, ANIM09, ANIM10) (P3 Affected audience, paragraph 1) |
|  | Forget et al., 2021 | Improved quality of life of the elderly (U) | "The presence of XXX has improved the life quality of residents, it brings joy, spontaneity, affection, companionship, the impression that it understands them and that they can confide in it"; "There are little lights in their eyes … it relaxes them, there are smiles, laughs, the pleasure of seeing the dogs "; "The residents take them in their arms, caress them"; " anxiety is reduced ". (PSY02, ANIM03, ANIM06) (P3 quality of life paragraph 1) |
|  | Forget et al., 2021 | Increased cognitive stimulation (U) | "Silent people can start to speak, to express their feelings in the presence of the animal"; "that provides a point of reference, it is necessary to remember the day of the week when the animal is there"; "the animal revives memories" (ANIM10, ASG11) (Pg 5 cognitive stimulation paragraph 1) |
|  | Forget et al., 2021 | Physical stimulation (U) | "Residents agree to walk in ‘her’ presence,"; "they stroke ‘her’, brush…"; "they reach out to the animal to touch ‘him’, pet ‘him’ as he goes by" (PSY02, ANIM03, ANIM10) (Pg 5 Physical stimulation paragraph 1) |
|  | Forget et al., 2021 | Positive feedback from families and other caregivers (U) | "At the team level, there is enthusiasm, they ask when the dog is going to come back"; "the families are pleased, they see that their relatives can still speak on a common subject". (PSY04, AMP05) (Pg 5 feedback paragraph 1) |
|  | Tasseron-Dries et al., 2021 | Positive response in the resident to an activities (U) | “I thought it was great that my mother connected with that doll. Because for the first time, I saw some expression on her face again. Her eyes lit up again.” (family caregiver, daughter) “A kind of ‘seeing is believing’. And that makes it really really good.” (activity coordinator) (Pg 4 ‘Activities’: Preferences of family caregivers for activities with their relative living with dementia, paragraph 4) |
|  | McKenna et al., 2022 | Intervention not meeting staff’s expectation (U) | …at first, I was happy, ‘cos I was thinking, it’s [psychological formulation] gonna really help us…It’s just pointless. You’re doing, you’re doing the jobs…but then the behaviour’s still happening, but it’s like, you’ve done the recommendations so everything should be fine… [Participant 2] (Pg 4 Theme 1: Expectation, paragraph 5) …you think the problem’s gonna be solved…I think, at first you’re looking for a quick fix aren’t you? You think someone’s here that can fix it all […] we’re always open to new ideas and that’s what you hope for I think when they come - new input. But it doesn’t seem to be the case… [Participant 1] (Pg 4 Theme 1: Expectation, paragraph 6) |
|  | McKenna et al., 2022 | Benefit gained(understanding) from the intervention or confidence (U) | …so yeah, I understand a little bit, but now I actually know that I can, I can do that, and have the confidence in doing it or saying, it’s alright I’ll have a go, I’ll try… [Participant 5] (Pg 6 Theme 3: Understanding, paragraph 2) |
|  | Pieper et al., 2018 | Seeing results motivated them to utilise the intervention (U) | Nurse: “Well, the moment of getting her out of bed was always… how shall I say…. Well, most of the time we thought: we’ll help her after our coffee break, around 11 o’clock–11.30. But then I noticed, when we helped her to get out of bed, say, around 8 o’clock–8.30, that she came singing out of bed, went to breakfast, and was quite relaxed.” (Pg 9 Facilitating and impeding factors associated with the level of the individual resident/professional, paragraph 2) Nurse: “It’s actually easier now to try out pain medication. Elderly care physicians were often reluctant – but with this stepwise intervention we have more evidence to support our request for treatment.” (Pg 9 Facilitating and impeding factors associated with the level of the individual resident/professional, paragraph 2) |
|  | Ducak et al., 2018 | Seeing Results is Believing (U) | there and seeing it work and seeing the residents get engaged and smiling and taking part in an activity, especially residents when they don’t think that they’re capable really of doing much of anything ... it’s a bit of a shocker when they hear a resident being the first to call out the answer to something when they thought all they could do was repeat the same sentence or phrase over and over again and actually to see that those memories are there ... we just have to know how to get them to surface. consultant (C1) (Pg 18, enabling factors, paragraph 7) The reading program I would have to say is the biggest win that we’ve had not only for the resident themselves but to actually promote our department, like activation with nursing staff and families, and it’s like, ‘‘Ah, she reads! Oh my!’’ ... it really is truly amazing to listen to someone who may not have put two words together in such a long time and then be able to actually concentrate and read those words. recreation manager (R7) (Pg 18, enabling factors, paragraph 8) ...there’s always people that are kind of set in their ways and the naysayers... in particular the doll therapy where they thought it was infantilizing somebody to give them a doll, but again, when they see how they react to the doll and that it brings them joy ...they’re more likely to come on board with it. consultant (C1) (Pg 18, enabling factors, paragraph 9) |
|  | Kwak et al., 2021 | Calming effect of music (U) | One respondent stated that “it triggered memory and seemed to calm behaviors during times of sun downing.” (Pg 8 What residents liked about M&M, paragraph 2) Another respondent stated that “they seem more alert. Some residents will often sing along or hum to the music they hear, others will listen and often brings a smile to their face,” noting how residents seemed more engaged with their social environment and expressive. (Pg 8 What residents liked about M&M, paragraph 2) |
|  | Kwak et al., 2021 | Seeing the positive effects of M&M (U) | Seeing the positive effects of M&M on residents and residents’ characteristics (e.g., being calm, enjoyment, residents wanting to listen to music) was another facilitator (n = 43); so too was, as one respondent said, “CNA staff observing success.” (Pg 9 Facilitators of Providing M&M., paragraph 1) |
|  | Janzen et al., 2013 | Perception that NPI application was based on trial and error  (N) | Multiple factors (e.g., time of day, personality of the staff or resident, and environment) made the outcome of the NPI use unpredictable. |
|  | Van Der Ploeg et al., 2012 | Staff perceived value of volunteers (U) | Two-thirds of facility staff reported they considered volunteers as a needed extra pair of hands, an additional resource. They are invaluable, volunteers are invaluable. Dementia specific facilities need a high staff ratio and whilst we can cope with the care side of things, the lifestyle, the quality-of-life activities would be desperately lacking if we didn’t have volunteers, especially for people with dementia, because they need more one to one, they need small group activities. (#14) (Pg 4 Perceived benefits and difficulties, paragraph 2) |
|  | Van Der Ploeg et al., 2012 | Increased well-being for resident as perceived by volunteers (U) | When one of them is really depressed and down, the lifestyle coordinator lets me know to begin with so I can spend more time, but when they put the dog on their knee ... just the look on their face and they profusely say “thank you for bringing him.” (#30) And there’s so many out there that feel so alone, and missing their home and their own belongings and just being able to express what they feel, I feel, is very beneficial for them. (#22) (Pg 5 Perceived benefits and difficulties, paragraph 5) |
|  | Backhouse et al., 2016 | View that activities or NPIs are extras (U) | At times of staff shortages, the activity staff would often be reallocated to their other roles, meaning no activities occurred on those days. a lot of people just see the activities side as a bolt-on’ (Susan, Manager, CH2) ) (Pg 5 Barriers to including residents in activities, paragraph 7) |
|  | McKenna et al., 2022 | Being pessimistic (U) | …not everyone with dementia can be fixed… [Participant 1] …[following the intervention] there was nothing more we could possibly do with her […] we basically can’t try anymore… [Participant 9] (Pg 4 Theme 1: Expectation, paragraph 8) |
|  | Garrido et al., 2021 | Not seeing the value of music (U) | “It’s actually quite a challenging space to provide the evidence, because a lot of the time we’re talking about qualitative perceptions and observations where staff are just saying ‘Oh well Mr Smith seemed happier’, or ‘she was singing’ and things like that … Sometimes collecting that evidence then turns into a barrier. We used to ask people to fill in a little survey after they had listened to music with someone and then I found that people just stopped listening to the music because they couldn’t be bothered to fill in the evaluation. (P3 Group 1)” (P8, Challenges to Implementing Music Programs in Aged Care, paragraph 5) |
| **Synthesised Finding 22**: The care staff's concern about the consequences of NPI use could impair their implementation. | Forget et al., 2021 | Concern about animal hygiene (U) | "There could be a concern about hygiene, dog hair, if ‘he’ licks things"; "families could say that it was not very hygienic,"; "the downside is in terms of cleaning"; "our management isn’t very animal-friendly in our structure, it’s a matter of hygiene." (ASG01, PSY02, ANIM03, PSY04) (P3 hygiene, paragraph 1) |
| The adverse effects (e.g., Namaste care) and the concern about the health risks (e.g., animal hygiene) from the NPIs may impair their implementation, Additionally, the fear of residents’ behavioural outbursts during the attempt to use NPIs may also deter their future implementation. | Forget et al., 2021 | Concern about animal’s quality of life (U) | "The disadvantage is that XXX is an animal and so it is a sensitive living being, it will feel tension and be afraid of certain residents"; "we can’t force it"; "the dog is exhausted at the end of the day because it is called upon a lot”. (PSY 02, AMP05, PSY07) (P3 Animal’s quality of life paragraph 1) |
|  | Kaasalainen et al., 2019 | Adverse events such as incidence of skin breakdown (U) | When we initially started putting residents in there and they went for the four hours a day. Those residents who had maybe previous ulcers, re opened. (Site 1, director of care, page 2) Well it doesn’t hurt them, the residents. Except there are some that can’t go twice a day because they have skin break down, I mean they just can’t handle it body wise. (Site 2, PSW/CA, page 3) (Pg 9 Barriers to implementing ‘Namaste Care, paragraph 4) |
|  | Kong & Kim, 2022 | Staff's hurtful experiences (U) | In my case, I treat residents as my family, but some residents with dementia become violent. They often bite, hit, and spit at me. (Participant 18, care worker) (Pg 6 Staff's hurtful experiences, paragraph 1) Because residents present dementia-related behaviors to care workers who provide the most care … many negative feelings build up, so some care workers treat residents badly in return. (Participant 9, RN) (Pg 6 Staff's hurtful experiences, paragraph 1) Sometimes, familyes are upset, angry, and out of control. When they behave like that, we are so shocked. Some families even say swear words to care workers. (Participant 24, NA) (Pg 7 Staff's hurtful experiences, paragraph 2 Some familyes observe us carefully to find our mistakes and then accuse us. In that case, frankly speaking, we feel bad and become less kind to them. (Participant 7, care worker) (Pg 7 Staff's hurtful experiences, paragraph 2) |
| **COM-B Category: Motivation-Reflective motivation** |  |  |  |
| **Social or professional identity or role** |  |  |  |
| **Synthesised Finding 23**: The lack of intervention ownership among the staff and role mismatching may impair the implementation of NPIs. | Ervin et al., 2014 | Not viewing as nursing responsibility (U) | ‘‘Diversional Therapy (DT) is responsible for these therapies as nursing staff are too busy with personal care. Behaviours increase in severity on weekends when DT is absent’’ ‘‘Diversional therapy are involved with this not nursing staff’’ (Pg 4, Cognitive oriented strategies in dementia care, paragraph 2) ‘‘No time for nurses to do this strategy, the domain of DT’s. Evenings are worse as nurses are busy and many residents are sundowners’’ (Pg 4, Stimulation oriented strategies in dementia care, paragraph 3 |
| When nursing staff is not taking ownership of behaviour management, jobs are not assigned in line with the scope of practice for caregivers and volunteers, it could deter the uptake of NPIs. Additionally, if caregivers don’t consider NPI as their job and families leave their loved ones to the care staff, the implementation of NPIs may be slowed. | Nunez et al., 2018 | Nurse burden and responsibilities (U) | ‘And simply because they have to air their feelings, you know? They get frustrated. Sometimes they may feel that they are delegating their duties to the best of their ability, and the staff … So it’s unfortunate sometimes that the night nurse feel that they have not got that assertiveness to be rude to—they do not like the word, instruct. You know? Tell, the staff, “This is what is required to do.” Because they should know anyway. But there is that kind of an issue sometimes’ CSFG 2 (Pg 7 table 1 Nurse burden and responsibilities) ‘They have to make decisions. You know, really life-threatening decision. And sometimes you think you are doing the right thing and sometimes you might not, so you always need to have a second opinion. But if you have had a fairly regular training and you are supported by the management team... but it’s always handy, reassuring when someone is actually working together with you, I think. Then you feel that whatever is going to happen, you are going to have a reflective practice, and to learn from that. Because everybody is a human being and you make mistakes.’ CSFG 2 (Pg 7 table 1 Nurse burden and responsibilities) |
|  | Hussin et al., 2021 | Responsibility outside the job scope (U) | “They don’t want to bring the mother to the hospital and refuse medication even though we give the options, so it’s like, ‘as long as we pay the monthly fee, then that’s it’….” (Shuhada, caregiver) “Complicated task, such as putting in the catheter, I don’t really know how to do it.” (Taripnan, caregiver) (Pg 12 Barrier: Responsibility outside the job scope paragraph 1) |
|  | Lawrence et al., 2016 | Concern about engaging all staff in intervention (U) | What will happen is they will talk, smile and pretend to understand and then after it will be a different thing. Some of them have the attitude, ‘It’s not my job, I am just here to clean him, feed him, that’s it, I don’t need to do anything else, it’s not my job’. (2002) (Pg 4 table 1, (2c) Relationships within the team) |
|  | Miller et al., 2021 | Designating a single staff member responsible for filling and turning on the diffuser each day (N) | None |
|  | Van Der Ploeg et al., 2012 | Being asked to perform jobs that only staff members are trained to do (U) | Yes sometimes they ask me to take them to the toilet and I say “Sorry, I can’t do it.” (#41) |
|  | Miller et al., 2021 | Championing by activity director (N) | None |
|  | Griffiths et al., 2019 | Mapper status and leadership skills: respect held for the mappers within the home (U) | “It’s people that you know and peer-led, it’s, you know, it’s not like somebody from outside coming and talking with them, it engages the staff.” (Manager) Pg 8 |
|  | Pieper et al., 2018 | Presence of a person with a motivational leadership style (N) | None |
|  | Tasseron-Dries et al., 2021 | Reluctance of family caregiver to take extra obligation (U) | “Yes, I have taken advantage of it in the sense of: oh, there is singing here this afternoon and she really enjoys that. She’ll be willing to go. And then I can do something else. And I will come in tomorrow”. (Family caregiver, son) (Pg 6 ‘Activities’: Preferences of family caregivers for activities with their relative living with dementia, paragraph 8) |
|  | Tasseron-Dries et al., 2021 | The culture of leaving everything to the nursing home (U) | “Family could do more, but we - as care professionals - should also encourage that. Now we say: you can’t continue like this, you need to go to the nursing home and they can take care of everything there. And then we don’t have to do anything anymore. That is the shift we need to make. We do tend to take over completely and are very much hospitalized in that sense. In this shift the family would also be allowed more and do more if they want to. This is still too far away. First there is this whole other step that needs to be realized” (manager) (Pg 6 ‘Activities’: Preferences of family caregivers for activities with their relative living with dementia, paragraph 7) |
| **COM-B Category: Motivation-Reflective motivation** |  |  |  |
| **Belief about capability** |  |  |  |
| **Synthesised Finding 24**: The lack of trust by care staff in psychologists and the loss of hope among families regarding their loved one’s participation in activities could deter NPI implementation. | Tasseron-Dries et al., 2021 | Perceived difficulty to participate (U) | “You can tell on all sides that it generates feelings of helplessness. People are willing, but they don’t really know how. It’s only few hours. I manage that pretty well now, although it can be sad sometimes, I can more or less accept how far gone she is. But I have seen a group of family members who attended twice, it is so painful every time to see your wife no longer able to do anything. Then you won’t participate in this kind of program.” (family caregiver, daughter) (Pg 6 ‘Personal circumstances’: Personal context of family caregivers, paragraph 3) |
| When the care staff lacks motivation because of their mistrust in the abilities of psychologists to provide innovative ideas for managing residents' behaviour and family caregivers hesitate to participate in activities with their loved ones, NPI implementation may be slowed. | McKenna et al., 2022 | lack originality of the psychologist suggestions (U) | …you do understand what they’re saying, but you think, well, we knew that anyway (laughs). I know it’s a bit awful to say that, but we always say, well we’ve known that, that’s what we’ve done… [Participant 3] …she’s only told us what we know. She’s not told us any other ways to deal with him… [Participant 4] (Pg 7 Theme 3: Understanding, paragraph 5) |
|  | Janzen et al., 2013 | Need little training to implement (N) | None |

## U=unequivocal; C=Credible; N= not supported by illustration

## 
